# Supplementary material for: Rapid incidence estimation from SARS-CoV-2 genomes reveals decreased case detection in Europe during summer 2020
Source: Nat Commun. 2021 Oct 14;12:6009. doi: 10.1038/s41467-021-26267-y (PMC8517019; doi:10.1038/s41467-021-26267-y)
Supplement: Supplementary file 1 — Supplementary Information [file 41467_2021_26267_MOESM1_ESM.pdf]

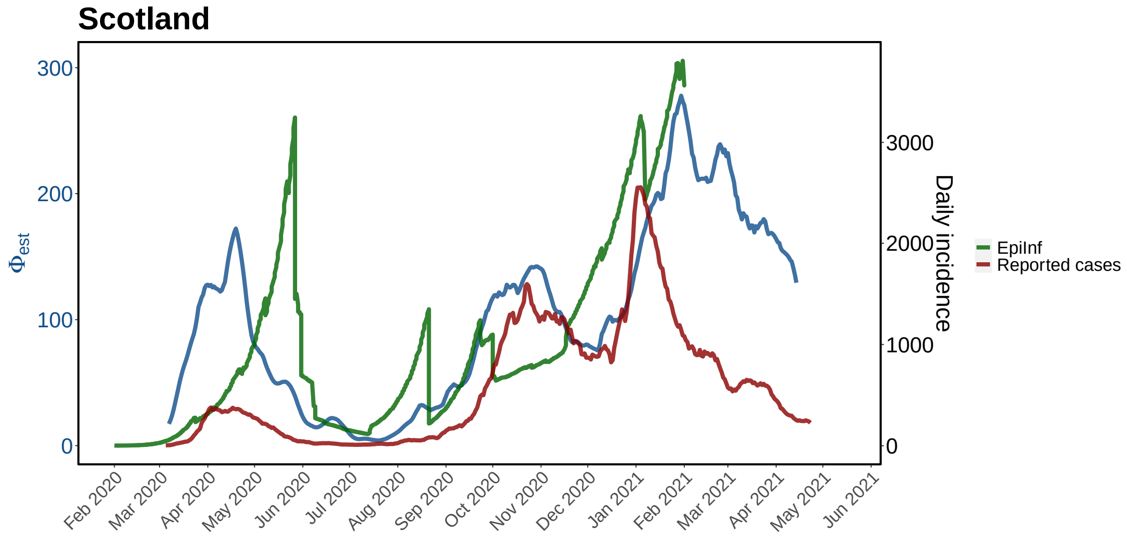

Supplementary Figure 1: Comparison of daily incidence estimates for Scotland using the incidence correlate derived from GInPipe (blue line, median estimates) and EpiInf (green line, median estimates) vs. the reported cases (red line, seven day rolling averages).

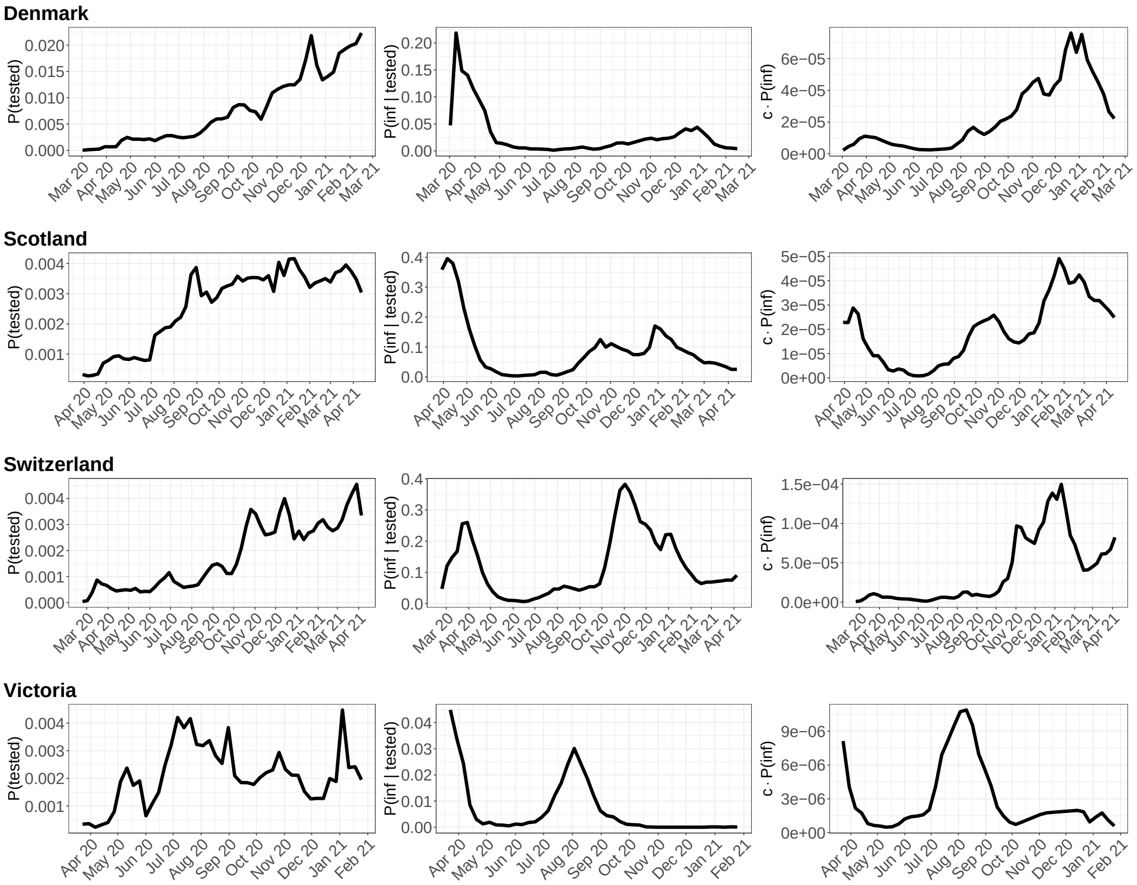

Supplementary Figure 2: From left to right: Probability of being tested, conditional probability that a tested individual is infected, and probability of being infected over time. From top to bottom: Denmark, Scotland, Switzerland, and Victoria (Australia).

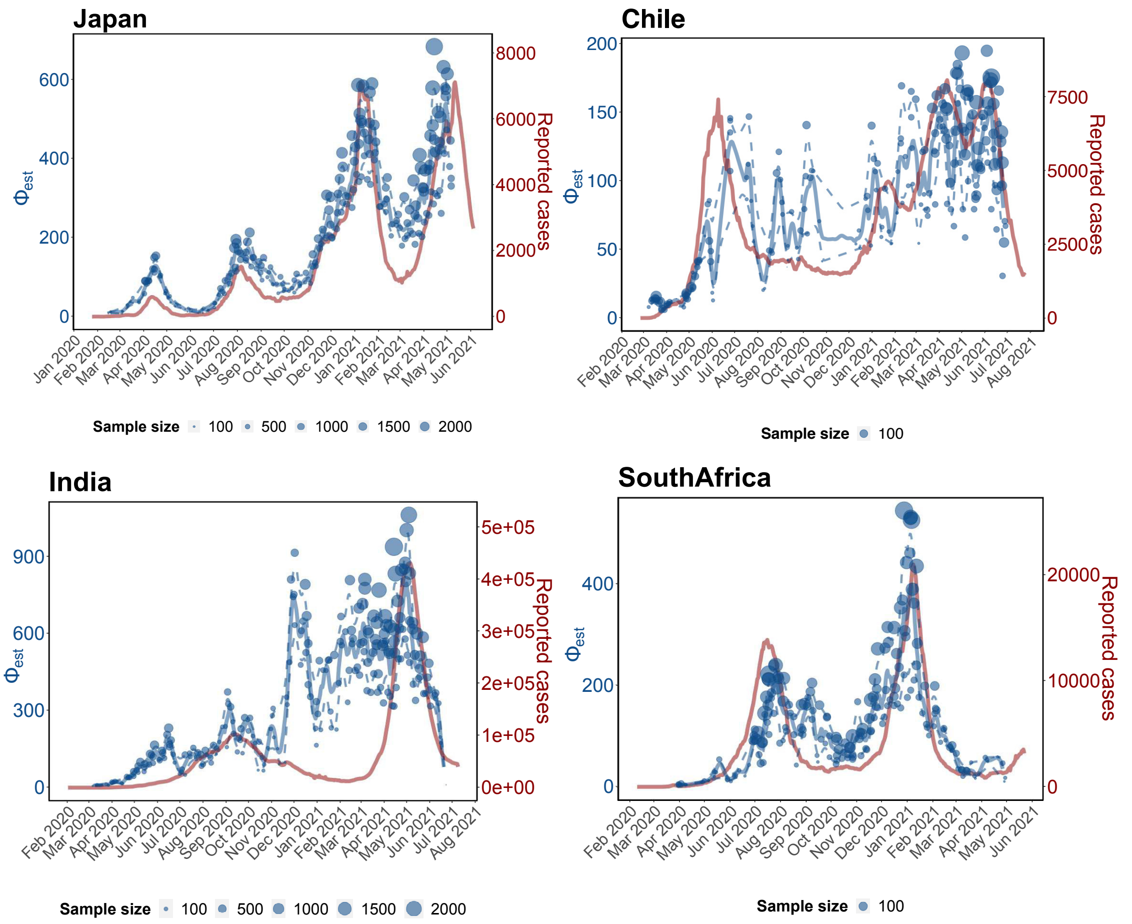

Supplementary Figure 3: Incidence reconstruction for additional countries. The graphic depicts the genome-based incidence reconstruction (in blue) using the proposed method (left axis) vs. the 7 days rolling average of newly reported cases in red (right axis). Blue dots depict ϕ_b point estimates of the incidence correlate, where the size of the dot is related to the number of sequences used to infer ϕ_b. The solid and dashed blue lines denote the median smoothed trajectories and their 5th and 95th percentiles. **Upper left.** Japan (n = 49.986 sequences) **Upper right.** Chile (n = 3.261 sequences) **Lower left.** India (n = 19.979 sequences) **Lower right.** South Africa (n = 3.347 sequences)

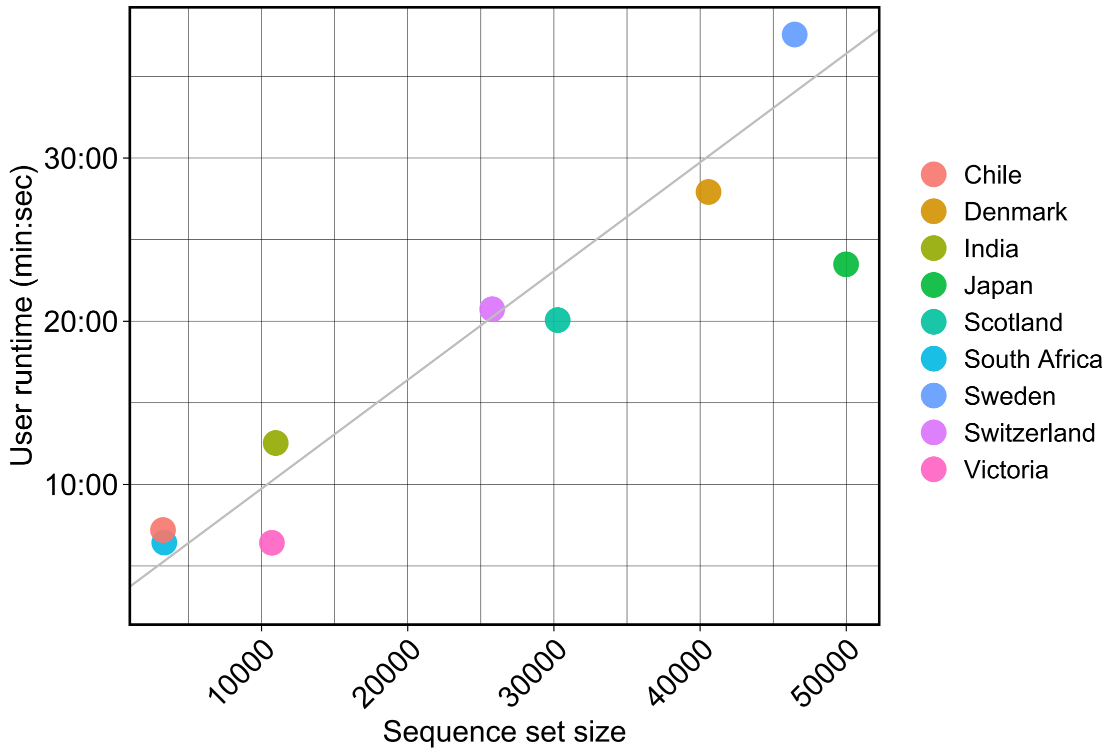

Supplementary Figure 4: Runtime of GInPipe scales linear with the number of sequences. The graphic depicts the runtime of executing the entire GInPipe pipeline on a standard laptop (2.3 Ghz, 2 cores) for different data sets.

Supplementary Note 1

The aim of Supplementary Note 1 is to evaluate, based on *in silico* simulated outbreaks, whether GInPipe can infer the number of infected individuals solely from viral genetic data. Moreover, we want to study the robustness of the method to missing data, unbalanced, time-dependent or biased sampling, introduction of unrelated sequence variants, its ability to reconstruct non-smooth dynamics, as well as its sensitivity to changes in the pathogen mutation rate and selective pressure.

SN.1 *In Silico* evaluation of GInPipe

As a proof of concept, we set up a simulation of a virtual outbreak: We formulate a stochastic epidemiological process by which a population of infectious agents progress with a time-dependent replication rate. This generates the number of infected individuals at each time step ('ground truth'). In our simulations, each infected individual can contribute to the pandemic and is associated with one viral genome. These genomes can be transmitted and randomly mutated in every time step. The set of genomes constitutes the data source for GInPipe. More precisely, we use the *in silico* generated viral genomes to compute the incidence correlate $\phi(t)$ using GInPipe. The derived $\phi(t)$ estimates are then compared with the number of infected individuals $N(t)$ at each time step.

SN.1.1 Summary and Limitations

We thoroughly tested the performance of our method and probed its weaknesses based on the *in silico* simulations. We found that the method's ϕ estimates linearly correlate with the infectious population size N_{true}. Different binning strategies, which are used to reconstruct the temporal incidence profiles yield complementary results. However, ϕ estimates can become inaccurate when the population of infectious individuals is small. 'Outliers' are however eliminated through application of a convolutional filter (smoothing). The ϕ estimates are unbiased in the case of missing data. However, the method becomes less accurate when too little viral sequences are available and when the "evolutionary signal" vanishes. Time-dependent changes in the sampling proportion have no effect on GInPipe. Evolutionary biased sampling (e.g. over representation of closely related sequences) decreases the evolutionary signal. This could, in principle, be problematic, if drastic changes, e.g. from a representative- to a biased sampling occur.
The importation of variants (introductions) that subsequently are community transmitted, do not affect the performance of the method. The same applies to introductions which are not community transmitted. In the extreme case of persistently large frequencies of non-transmitted introductions, however, the slope of the linear correlation function may change. Yet, a linear relationship between the incidence estimate ϕ and the infectious population size N_{true} is retained and the reconstruction of the pandemic trajectory is still possible.
GInPipe is able to reconstruct very steep increases and decreases in the population dynamic. Selective pressure has no effect on GInPipe's incidence reconstructions. With regards to applying GInPipe to other respiratory viruses, low mutation rates decrease the "evolutionary signal", but may be compensated by increasing the bin sizes, at the expense of temporal resolution.

SN.1.2 Outbreak simulation

For the evaluation of the method, we simulate the evolutionary dynamics of a viral outbreak. We simulate a time-discrete (generation-wise) population growth, where each simulation starts at time t_0 and ends at $t_{\text{final}} = 120$ (below, we also evaluate the corresponding continuous-time process). At t_0, the outbreak starts with $N_{\text{init}} = 50$ identical sequences of length $L = 1000$.
The nucleotides of the initial sequence are chosen randomly. In each of the t_{final} generations, the current population of size $N(t)$ replicates with replication rate ρ and mutates with mutation rate μ. To simulate non-monotonic outbreak trajectories, we chose a sinusoidal rate, depending on time t:

$$\rho(t) = \frac{\sin(t * 0.11)}{15} + 1.02 \tag{SN.1}$$

The number of sequences in the new generation $t + 1$ is sampled from the Poisson distribution with

$$N(t+1) \sim \mathcal{P}\big(N(t) \cdot \rho(t)\big) \tag{SN.2}$$

The sequences for the next generation are chosen randomly with replacement.

The sampled sequences of the new generation are mutated with mutation rate $\mu = 0.0001$. Analogous to the previous step, the total number of positions to be mutated is sampled from a Poisson distribution:

$$n_{\text{mut}} \sim \mathcal{P}\big(\mu \cdot N(t+1) \cdot L\big) \tag{SN.3}$$

These mutations are randomly chosen across all sequences and positions without replacement (below, we also simulate selection pressure). Each nucleotide is mutated according to the Kimura substitution type model [1] with fixed rates for transitions ($A \leftrightarrow G$, $C \leftrightarrow T$ with $\alpha = 0.6$) and both types of transversions ($A \leftrightarrow C$, $G \leftrightarrow T$ with $\beta = 0.2$, as well as $A \leftrightarrow T$, $C \leftrightarrow G$ with $\gamma = 0.2$). During the course of simulation, each position could in principle be mutated several times.

This procedure results in a sequence set for each generation, which is written into a fasta file. The header of each sequence contains an ID and a date (arbitrary start date + t days) to conform with the format required for the computational pipeline (`https://github.com/KleistLab/GInPipe`). We ran 10 simulations with the settings stated above. The trajectory of the population dynamics are depicted in Figure SN.5.

Moreover, we sub-sampled the the generation-wise data set in different scenarios (section SN.1.7) and created fasta files for the respective scenarios.

SN.1.3 Execution of the method

For each of the generated (sub-)samples we ran the whole pipeline as described in the *Methods* section (main manuscript). ‘Temporal bins’ were spanning 2, 4, 6, 8, and 10 days (here days correspond to generations) and ‘equal size bins’ were created comprising 2, 5, and 7 percent of all sequences. We evaluated the normalised ϕ_b estimates for each bin b according to eq. (1) in the main manuscript.

SN.1.4 Incidence estimate ϕ_b correlates linearly with infectious population size N_{true}

As depicted in Figure SN.6 (left), the bin-wise ϕ_b estimates show a strong linear relationship to the ground truth N_{true} with high significance (Pearson correlation coefficient $r = 0.98$, $p < 10^{-16}$). More specifically, we observe a linear scaling c of the incidence estimate ϕ with N_{true} of the form

$$N_{\text{true}}(t) = c \cdot \phi(t) + \text{error}. \tag{SN.4}$$

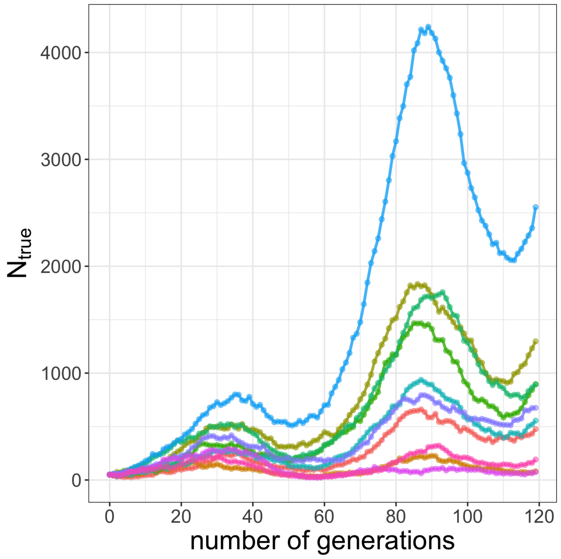

Figure SN.5: Trajectories for 10 simulations of population growth showing $N_{\text{true}}(t)$.

The deviation grows for larger values, because the considered evolutionary model is a Poisson process with error $= \sqrt{\phi(t)} \cdot \epsilon$, where ϵ is a zero-centered, symmetric, additive error.
In Figure SN.6 (right), we compare the percent deviation from the linear regression $\hat{\phi}(t)$:

$$\%\text{deviation} = \frac{\phi_b(t_b) - \hat{\phi}(t_b)}{\hat{\phi}(t_b)} \cdot 100, \tag{SN.5}$$

with t_b denoting the *mean* date of all sequences in bin b. The deviation measure allows to center the approximation errors and henceforth analyse how distinct parameters may affect the method's performance. However, below, we also assess differences in the true vs. GInPipe reconstructed effective population sizes R_e.

We can see that the majority of the point estimates are quite accurately approximated with low percent deviation. Moreover, the estimation seems unbiased (symmetrically dispersed around 0% deviation) and large deviations are rare. Outliers are most prominent when we bin sequences by equal amounts of days, scattering in both directions, hence under- and overestimating. Creating bins of equal size, however, is slightly more prone to underestimating of $c \cdot N_{\text{true}}$.

SN.1.5 Effect of binning strategies

Next, we investigated the sources of outliers. In Figure SN.7, we show violin plots that colour-code the spanned days per bin (left) and the number of sequences per bin (right). Large deviations from the linear fit are particularly occurring for very small infectious population sizes N_{true}, which is indicated by the smaller markers in Fig. SN.7.
Moreover, overestimation for the 'equal size' binning is caused by bins spanning large temporal windows, around 20 days (see red dots in SN.7 (left), third violin plot). The outliers that occur for binning with equal amounts of days, to the contrary, are mainly caused by bins containing only very few sequences (red dots in the central violin plot in Figure SN.7 (left)). In these cases, the true population size is already small (small dots) and hence the amount of sequences used to infer the parameter are also low. Therefore, it may happen that the time until the next mutational event lies outside the particular bin, and not enough information is given to infer the (very low) population size.
We can conclude, that the choice of the binning strategy is important for accurate results. On the one hand, the bin has to be large enough in order to contain mutational information, but on

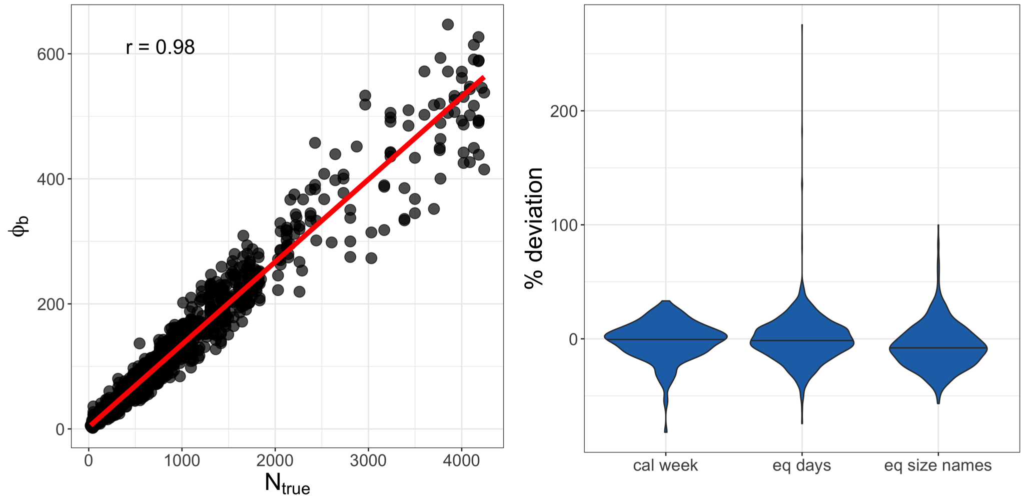

Figure SN.6: Linear relation between $N_{\mathbf{true}}$ and ϕ. **Left:** Dotplot showing N_{true} versus the estimated point estimates ϕ_b per bin (full data set). The red line shows the linear fit. **Right:** Violin plot of the percent deviation of ϕ_b from the linear fit for the different binning types (i) calendar week, (ii) equal amount of days and (iii) equal number of sequences per bin.

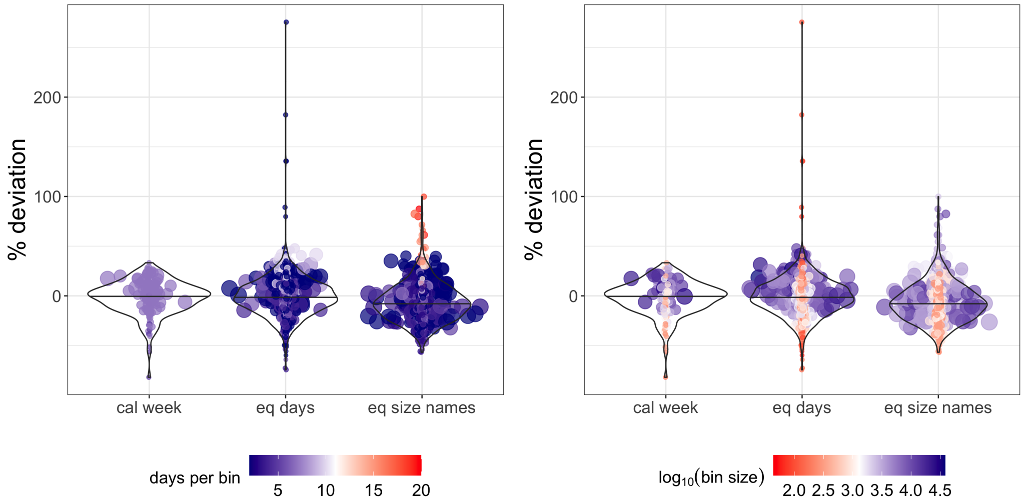

Figure SN.7: Deviation of ϕ from the linear correlation to $N_{\mathbf{true}}$. Incidence correlate ϕ was calculated for the different binning types, i.e. by calendar week, equal days and equal bin size. The size and dispersion of data points indicate the value of N_{true}. **Left:** Colour and transparency indicate the days spanned per bin, with blue and transparent for low values, red and solid for large values. **Right:** Colour and transparency indicate the number of sequences per bin (in $\log_{10}$), with blue and transparent for large bin sizes, red and solid for small bin sizes.

the other hand not too large such that the population dynamics can still be temporarily resolved. Moreover, prediction accuracy declines with decreasing population size N_{true}.

SN.1.6 Smoothing corrects for outliers

The point estimates are smoothed using a convolution filter with a window size of 7 (days). To infer statistical uncertainty in ϕ profiles, we conducted a sub-sampling procedure of the ϕ point estimates (details in the *Methods* section, main article).

In Figure SN.8 (left), we show the smoothed reconstruction ϕ_{est} of the simulated dynamics of N_{true}, which we saw in Figure SN.5. Figure SN.8 (right) also shows, in a representative simulation, that the incidence correlates ϕ (blue dots and line) yield a proportional estimate of N_{true} (red line) and reflects the overall epidemiological dynamic quite accurately.

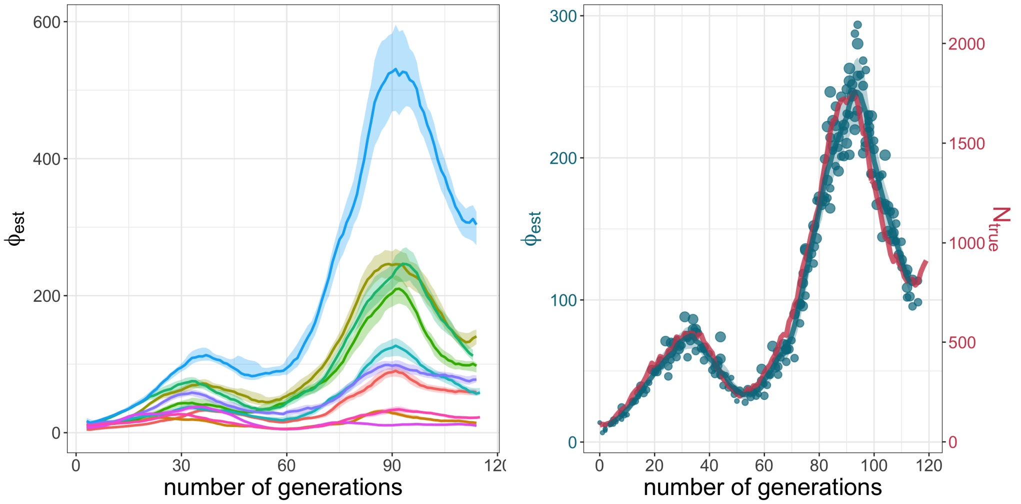

Figure SN.8: Reconstruction of outbreak dynamics. Left: The solid lines denote the smoothed median of the ϕ estimates for the 10 stochastic simulations of population growth. The coloured shades present the area between the 5th and 95th percentile derived from sub-sampling over ϕ_b point estimates. **Right:** The blue line shows the smoothed median of the sub-sampled ϕ_b estimates (points) for one of the simulations. The red line shows true incidence per generation N_{true}.

SN.1.7 Missing data does not affect incidence reconstructions

In the evaluation above, we have used all sequences that emerge in the evolutionary process. For a more realistic setting, we evaluated the estimation of ϕ by taking sub-samples of the total sequence set: We sub-sampled a certain fraction of all sequences (WSREL = proportional sub-sampling), and drew the same amount of sequences per generation (WSABS = unbalanced sub-sampling). For the sub-sampling schemes, we tested the following parameters:

- WSREL: fraction of sequences used for inference $r_{\text{sub}} \in \{0.9, 0.7, 0.5, 0.3, 0.1\}$
- WSABS: fixed amount of sequences at each generation (up to) $a_{\text{sub}} \in \{500, 350, 150, 50\}$

The sequences are randomly chosen without replacement at every time point (below we also evaluate other sampling schemes).

In Figure SN.9, we show violin plots of the percent deviation, akin to Eq. (SN.5) for the raw point estimates (yellow) and the smoothed estimates (green) for different sub-sampling schemes. We can generally observe that ϕ is strongly correlated with the population size N_{true} ($r \geq 0.94$), even in scenarios with significant under-sampling. Moreover, ϕ is an unbiased predictor of the incidence (median deviation centered around 0). The deviation from the linear correlation line slightly increases with more severe under-sampling, i.e. the ϕ estimates are still linearly correlated with N_{true}, but the ϕ estimates become less accurate.

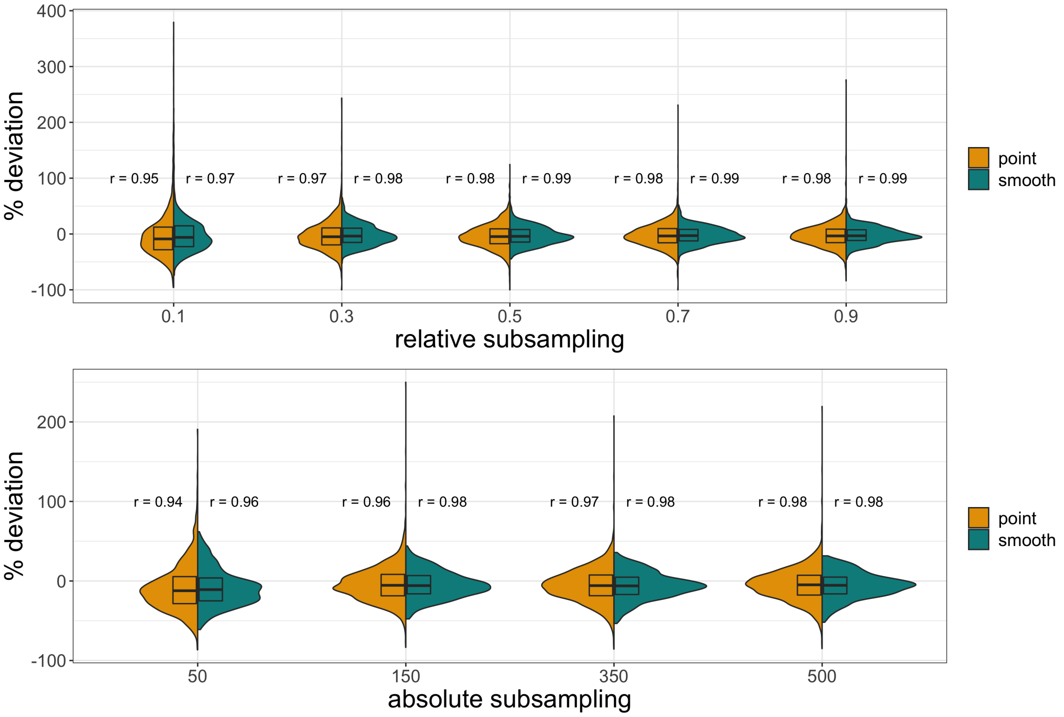

Figure SN.9: Split violin plot showing the percent deviation of the linear fit for ϕ estimates for respective levels of subsampling. The box indicates the interquartile range (IQR) with the median. The left, yellow part shows the deviation for the point estimates, the right, green part depicts the deviation for the smoothed estimates. On each side of the violin plot, the r value indicates the respective level of linear relationship of ϕ and N_{true}. **Top:** Proportional sub-sampling. **Bottom:** Absolute sub-sampling.

SN.1.8 Effects of time-dependent sampling rate on incidence reconstruction

In order to further investigate whether there are any effects of the sampling proportion on incidence reconstruction, we assessed the prediction for a quasi-constant population size with different scenarios of changing subsampling over time. For this, we chose the growth rate $\rho(t) = 1$, i.e. the population of infected individuals will stochastically fluctuate around its initial value, Fig. SN.10, upper left.
First, we inspect the incidence reconstructions without temporal changes in sub-sampling. In Fig. SN.10 (upper right), we show the incidence reconstructions for which 100% of the data is available for GInPipe. In Fig. SN.10 (lower left and right), the median reconstructions are shown for sub-sampled data.

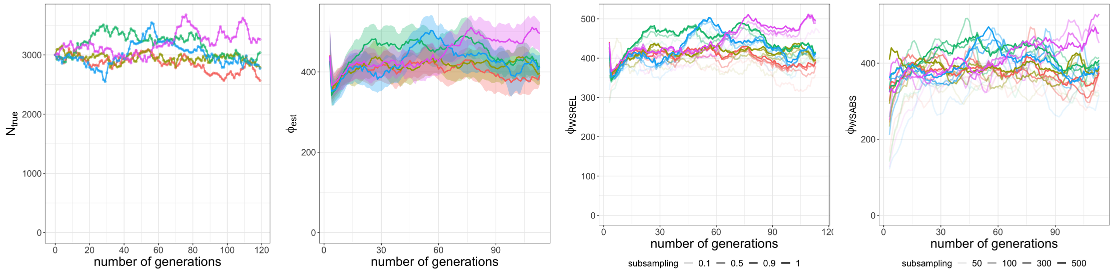

Figure SN.10: Simulations for pandemic dynamics with no apparent trend. Left: True population size over time for 5 simulations starting with 3000 sequences and no increase/decrease in the population of infected individuals other than stochastic noise. **Center left:** The solid lines denote the smoothed median of the ϕ estimates for the stochastic simulations. The shaded areas denote the 90% confidence interval. **Center right:** Smoothed median of the ϕ estimates with relative sub-sampling of sequences with proportions indicated below the graphic. **Right:** Smoothed median of the ϕ estimates with absolute sub-sampling with number of sequences as indicated below the graphic.

We observe that the sub-sampling does not affect our estimates drastically. However, the sub-sampling is not time-dependent in these simulations.
Next, we inspect the effect of drastic changes in the sampling rate. For this, we evaluate oscillating changes in the sampling rate, Fig. SN.11 (upper right panels), as well as a sudden increase from a sampling rate of 0.1 to 1.0 Fig. SN.11 (lower left panels) and from 0.5 to 1.0 Fig. SN.11 (lower right panels).

In essence, we observe no systematic effect of temporal changes in the sampling proportion on the incidence reconstructions. This suggests that sudden increases of genomic surveillance of SARS-CoV-2, as observed in many countries after the appearance of the alpha variant (B.1.1.7), do not impact incidence reconstructions using GInPipe. For the extremely low sampling rates in the oscillating case (around generation 45 and 105) we observe inaccuracies and large variance. Therefore, we conclude that temporal changes in the sampling rate do not introduce systematic biases for incidence reconstruction. In line with previous observations (Fig. SN.4), we can however conclude that very low amounts of data increase uncertainty in the incidence reconstructions.

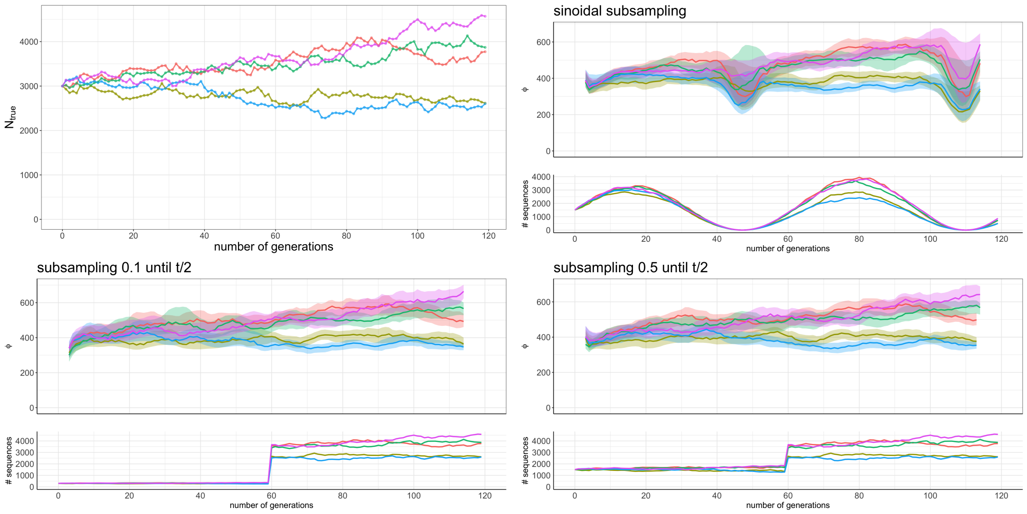

Figure SN.11: Incidence reconstructions with steady population size and time-dependent sub-sampling. Incidence reconstruction shown as smoothed median of the ϕ estimates (solid lines). The coloured shades present the area between the 5th and 95th percentile derived from sub-sampling over the point estimates. The panels below the incidence reconstruction show the number of sampled sequences for the prediction. **Top left:** Simulated population dynamics (5 simulations). **Top Right:** Reconstructed incidence history when the sampling proportion is oscillating. **Bottom left:** Reconstructed incidence dynamics when the sampling proportion abruptly increases from 0.1 to 1.0. **Bottom right:** Incidence reconstructions when the sampling proportion abruptly changed from 0.5 to 1.0.

SN.1.9 Incidence reconstruction when sequences are sampled by similarity

In the previous sections, we assumed that sequence sampling is representative, i.e. that the sub-sampled sequences are randomly selected. In this section, we evaluate if GInPipe is able to reconstruct incidence histories when sequences are sampled with an similarity-bias. This is supposed to emulate extreme events when sequencing is entirely unbalanced, e.g. there is a large under-detection and detection is driven *entirely* by contact tracing. For example, after an infected individual is detected, any *forward* and *backward* contacts are investigated and positive cases are sequenced. At the same time, there would be very little diagnosis without a known contact. The sequenced samples would then be highly similar to the sequence from the first detected case, if they are linked by transmission events.
For the sequence sub-sampling process, instead of choosing the sequences randomly with the same probability, we assign a similarity value $d_t(s)$ for each sequence s to the previous generation, which is used as a probability measure for being sampled in the next generation. The similarity measure is given by the hamming distance (number of differing nucleotides) to the preceding sequence. If the previous generation contains various sequence types, the minimum distance is chosen:

$$d_t(s_i) = \min_{s_j} \left(d(s_i, s_j) \right), \forall s_i \in S_t,\ s_j \in S_{t-1}, \tag{SN.6}$$

with S_t being the sequence population at time t, and S_{t-1} the sequence population in the preceding generation $t-1$.
We chose to half the chance of being sampled with every nucleotide difference. The probability p_t of each sequence s_i to be chosen at time t is given by

$$p_t(s_i) = \frac{1}{D} \cdot 2^{-d_t(s_i)}, \tag{SN.7}$$

with $D = \sum_{j \in S_t} \frac{1}{2^{d_t(s_j)}}$ being a normalising factor.

Figure SN.12 (top left) shows the simulated population dynamics. Fig. SN.12 (top center) shows the respective incidence reconstructions without sub-sampling (full data set), i.e. the sequences are not sampled by similarity yet. As expected, the incidence estimations with the full data set yields good results ($r = 0.98$ for the smoothed estimates).
When the sequence set is sub-sampled by similarity as described above, we can see a scaling effect, Fig. SN.12 (upper right). I.e., the smaller the sequence set (in terms of the proportion of the entire sequence set), the lower the ϕ values. Interestingly, the profiles are maintained Fig. SN.12 (upper right, Pearson correlations $r \geq 0.95$), while the slope between the populations size and the smoothed incidence estimate decreases with a decreasing proportions of sub-sampled sequences. Therefore, although a strong linear relationship can be maintained with proportional sub-sampling, the scaling factor is affected, Fig. SN.12 (top right and bottom).
This observation is explained by a decrease in the 'evolutionary signal' when sequences are sampled by similarity. This effect is most pronounced if the sampled sequence set becomes small.

Sampling non-proportional subsets over time, i.e. taking the same amount of sequences independent of the population size at each generation (50–500 sequences), leads to a irregular rescaling of the underlying dynamic. The linear relationship of the incidence correlate and the true population size is debilitated (Pearson correlation ranging from 0.85 to 0.95 for the smoothed ϕ estimates). This effect is depicted in Fig. SN.13, where the smoothed ϕ estimates for different levels of similarity-biased sub-sampling for one of the simulations are shown. On the left, the different shades of purple indicate the proportion of sub-sampling. As can be seen, the scaling factor decreases with the amount of sequences, but the dynamic can be retained. However, when non-proportional amounts of sequences are drawn, indicated by the shades of green in the right panel, the sub-sampling-driven scaling effect is uneven over time and the dynamic of the incidence correlate can become distorted. For the case of 500 and 350 sequences per generation, the estimates reach similar values for the first wave (around $t = 30$) compared with the entire data set

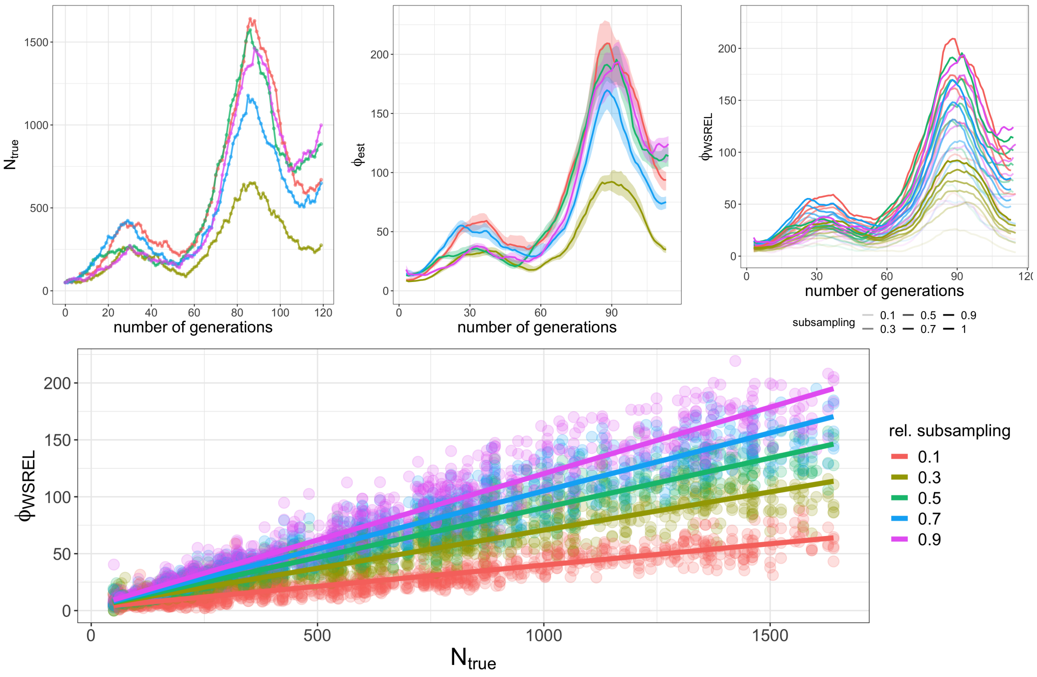

Figure SN.12: Incidence reconstruction when sequences are sampled by similarity. Top left: Simulated population dynamics. **Top center:** The solid lines denote the smoothed median of the incidence reconstructions with GInPipe using all available genetic data (no biased sub-sampling). The coloured shades present the area between the 5th and 95th percentile. **Top right:** Smoothed median of the re-sampled ϕ estimates with relative sub-sampling biased by similarity. **Bottom** Population size N_{true} vs. incidence correlate ϕ for different sub-samplings from the top right panel.

(the black dashed line). However, the second wave (around $t = 90$) is down-scaled and does not reflect the different magnitudes of the two waves.

In conclusion, when sequences are sampled by similarity, the 'evolutionary signal' decreases and the scaling between the true population size and the incidence estimate decreases. If this biased sampling is not changing through time, incidence histories can still be reconstructed. If the sampling strategy would change over the time course of the pandemic (e.g. from biased to representative sampling), difficulties with GInPipe-based incidence reconstruction may arise. Most other methods (e.g. phylodynamics) are likely to encounter similar difficulties when the data is systematically perturbed. For GInPipe, and likewise for any other method that utilizes pathogen genome data, a representative sampling of the population is important to guarantee that meaningful results are obtained.

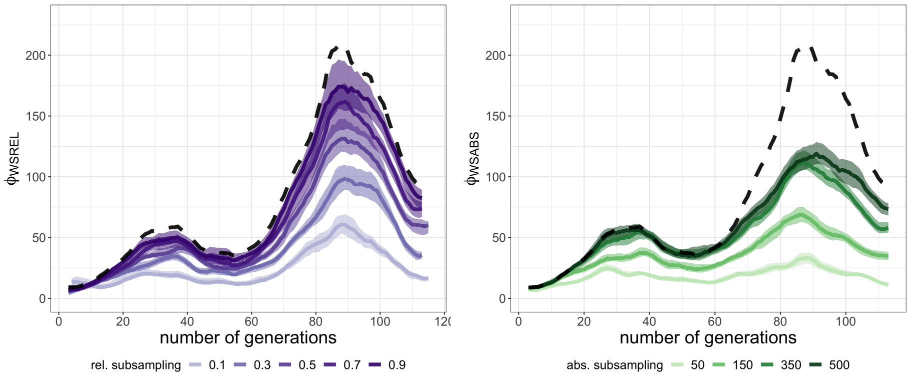

Figure SN.13: Incidence reconstruction with similarity-biased sampling for different levels of sub-sampling for one of the simulations. The shades indicate the level of sub-sampling. The black dashed line shows the estimates with all data. **Left:** Proportional sub-sampling. **Right:** Absolute sub-sampling.

SN.1.10 Incidence reconstructions for a continuous-time population dynamic

In the previous sections, we used a minimalistic approach for simulating the outbreak dynamics. In particular, we performed a time-discretisation, i.e. for each discrete time $t \in (0, 1, ..., 120)$, the population size was estimated by sampling from a Poisson distribution, $N(t+1) \sim \mathcal{P}\big(N(t) \cdot \rho(t)\big)$. Here, to verify that the time discretisation has absolutely no effect on the *in silico* assessment of the GInPipe method, we also provide simulations using the time-continuous birth-death process. We introduce the two reactions:

$$R_1 : \; I \xrightarrow{r_1(t)} I + I \qquad ; \qquad R_2 : \; I \xrightarrow{r_2(t)} \star \qquad \text{(SN.8)}$$

where R_1 denotes the 'birth' reaction, where an infected individual infects another individual. Reaction R_2 denotes the 'death' reaction, where an infected individual becomes non-infectious.
The reaction rates of the model above are given as $r_1(t) = \beta(t) \cdot I(t)$ and $r_2(t) = I(t) \cdot \delta$. Furthermore, we set $\delta = 0.5$ (1/day) and then derive $\beta(t) = \rho(t) \cdot \delta$, where $\rho(t)$ is given in eq. (SN.1).
We then simulate the time-continuous model using the Gillespie algorithm, which draws the time Δt to the next event (firing of reaction R_1 or R_2) from an exponential distribution. After each population dynamic event, we update the number of sequences (either cloning or deleting one sequence) and place $n_{\text{mut}} \sim \mathcal{P}\big(\mu \cdot N(t + \Delta t) \cdot L \cdot \Delta t\big)$ mutations onto the set of sequences.
After simulating a few outbreaks, Fig. SN.14 (left), we use GInPipe to reconstruct the incidence dynamics using the standard setting, as shown in Fig. SN.14 (middle and right). As can be seen, the choice of modelling approach (discrete time vs. continuous time) has no effect of GInPipe's ability to reconstruct incidence dynamics.
While the population dynamics may also be modelled by more complex S(E)IR-type models, the model above (eq. (SN.8)) can be easily deduced from such models by assuming that a sufficient number of susceptibles is available. However, the choice of structual model does not affect any of the analyses made in Supplementary Note 1.

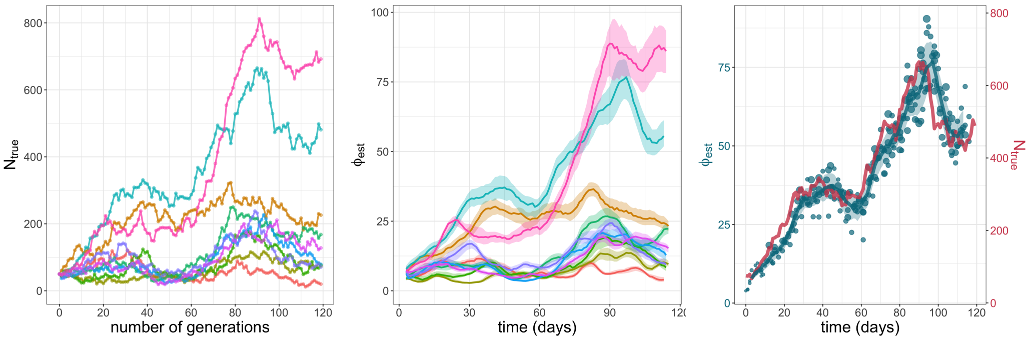

Figure SN.14: Simulations with birth-death-process. Left: True population size. **Middle:** The solid lines denote the smoothed median of the ϕ estimates for the stochastic simulations. The shaded areas denote the 90% confidence interval. **Right:** An example simulation of the epidemic (red curve) vs. the reconstructed incidence history.

SN.1.11 Comparison of R_e estimates

GInPipe allows to infer the effective reproduction number R_e, based on the Wallinga-Teunis method, as described in the *Methods* section in the main manuscript. In the *Results* section, we assess the accuracy of GInPipe by comparing the R_e values inferred from smoothed ϕ estimates with R_e values calculated from the simulated pandemic N_{true}. In Figure SN.15, we see the individual $\log(R_e)$ profiles for the 10 simulations from Section SN.1.2 inferred with N_{true} (bold

cyan lines) and the according smoothed ϕ estimates (red lines). The identity plot in Fig. 1 in the main manuscript shows the $\log(R_e)$ values inferred with N_{true} vs. ϕ, with the respective proportion of qualitatively agreeing or disagreeing predictions in the four quadrants. The accuracy is given by the proportion of qualitatively agreeing R_e values, yielding a value of 0.92 using the complete data set.

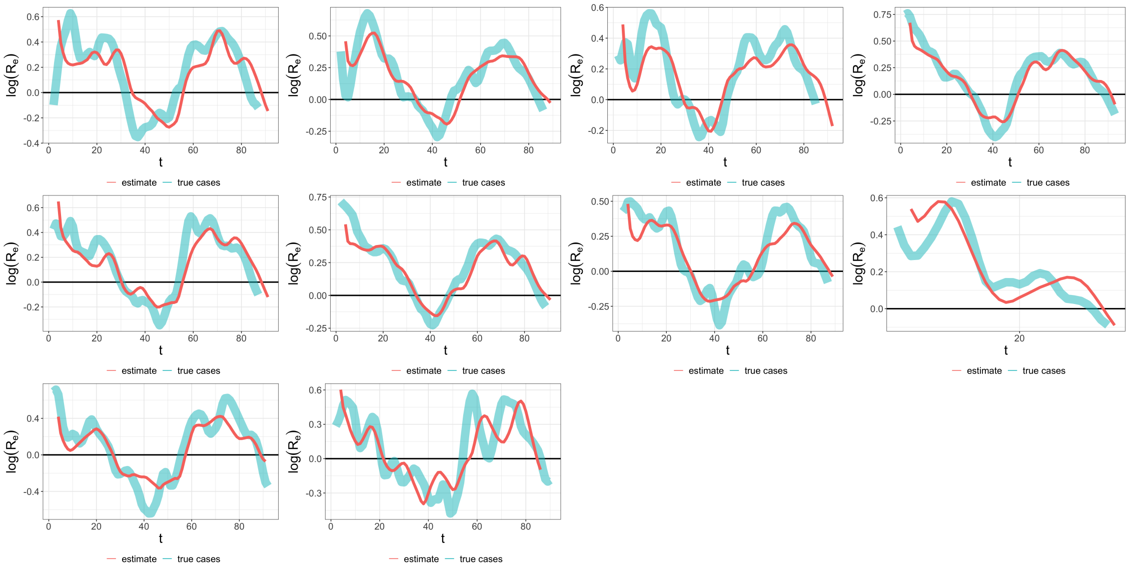

Figure SN.15: Individual R_e profiles. Individual R_e profiles for 10 simulations estimated from the true population sizes (thick cyan lines) and from ϕ estimates inferred with GInPipe (thin red lines).

Complementing the evaluations in the main manuscript, we additionally assess the accuracy for the sub-sampled data (Fig. SN.16). Here, we also observe good results, achieving accuracies between 0.89 and 0.92. In the case of very low sub-sampling proportions (10%), however, the accuracy declines to 78%, due to noisy ("wiggly") dynamics.

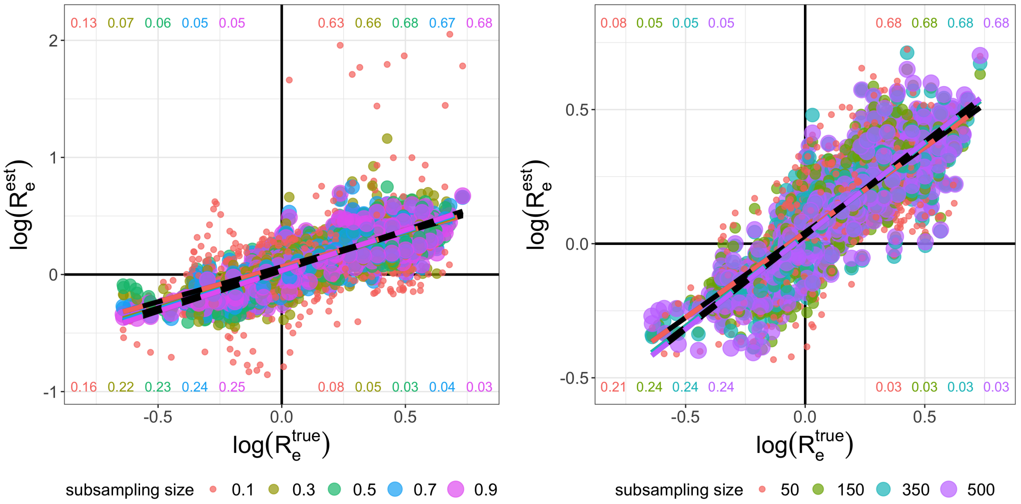

Figure SN.16: Dotplot showing the true $\log(R_e)$ values (x-axis) vs. the R_e values estimated using GInPipe for different levels of sub-sampling. Left: Relative sub-sampling (10, 30, 50, 70 or 90% of the data sampled.) **Right:** Absolute sub-sampling (a fixed number of either 50, 150, 350 or 500 sequences are sampled per time point, irrespective of the actual population size).

SN.1.12 Introductions of new sequences do not affect prediction accuracy

Above, we evaluated an isolated outbreak, where the diversity of mutants is emerging solely through evolution, i.e. reproduction and mutation. However, in case of a widespread epidemic, several outbreaks at different locations could lead to populations which evolve in parallel, causing different strains to emerge. Foreign strains may then be introduced into a local epidemic at any point in time.
To test whether the introduction of foreign strains affects the proposed method, we added $x_i \in \{50, 100, 500, 1000\}$ 'introduction sequences' at random time points to the initial outbreak. In all simulations, the 'introduction sequences' have 3% of the genome randomly mutated with regards to the founder sequence of the local outbreak. We performed 5 simulations that began with 1–10 start sequences. All sequences were considered to evolve with the same replication and mutation rates.
For a representative simulation, Figure SN.17 (left) shows the simulated epidemiological dynamics for different numbers of introductions. On the right, we see the corresponding correlations of the incidence estimate ϕ with the true population size N_{true}. The inferred ϕ estimates show a strong linear correlation with N_{true} across all simulated scenarios ($r = 0.99$, $p < 10^{-16}$).

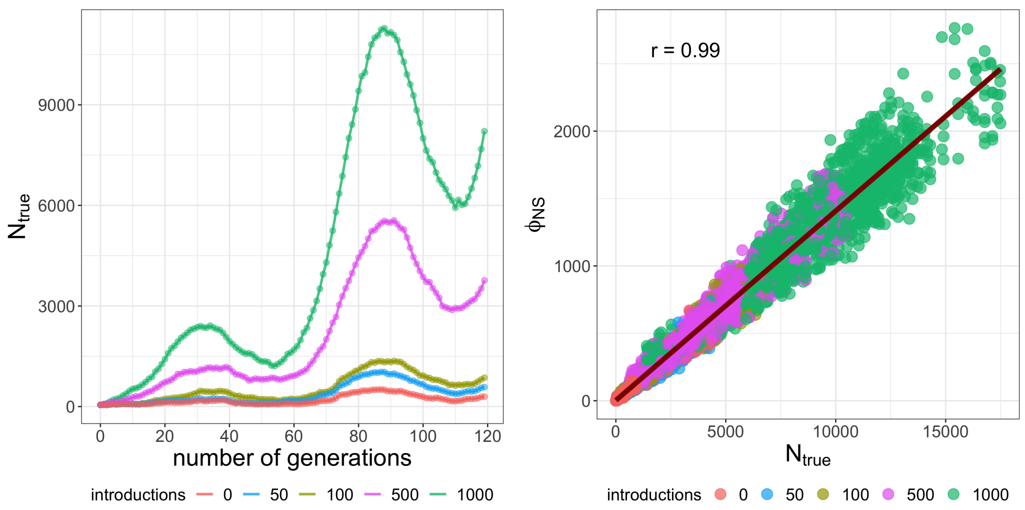

Figure SN.17: Simulations with foreign sequence introductions. **Left:** True population dynamics for the original outbreak (0 introductions) and after introduction of new sequences, each in a representative simulation. The total amount of introduced sequences at random time points is colour-coded. **Right:** Dotplot showing N_{true} versus the point estimates ϕ using 5 simulations per 'number of introductions' scenario. The colours indicate the number of introduced foreign sequences to the original outbreak. The black line shows the linear fit.

In Figure SN.18 we evaluate how the relative amount of introductions affects the percent deviation, akin to Eq. (SN.5). We can see that for the majority of the simulations the deviation is around zero with no apparent trend of increasing dispersion (prediction inaccuracy), when large numbers of foreign variants become introduced.
In summary, the predictions are not perturbed by introductions of foreign sequences. Potential biases from single introductions are compensated by ongoing local evolution (community transmission) of the imported sequences.

To investigate the effect of introductions without the compensating effect of community transmissions, we simulated an extreme scenario, where the introduced sequences are only added to the local outbreak and do not replicate or mutate. They simply occur as additional haplotypes in the population. In reality this would be the case, if infected people from a different region get tested,

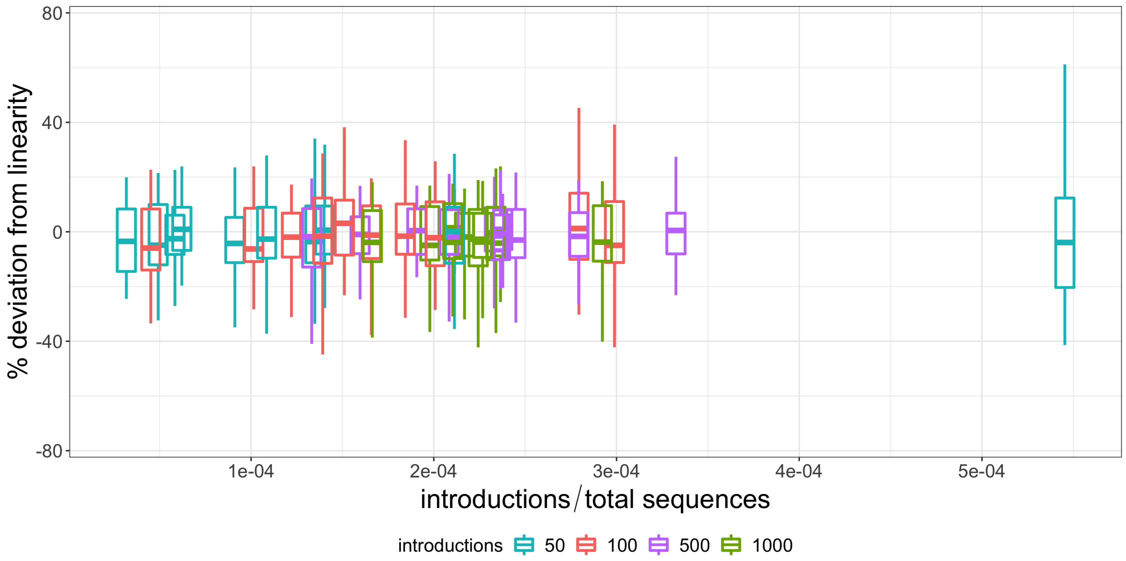

Figure SN.18: Deviation from linear correlation for smoothed ϕ with introduced sequences. Boxplots showing for each simulation the percent deviation of smoothed ϕ from the linear fit to N_{true} in comparison to the ratio of introductions and total amount of emerging sequences in the evolutionary process. The box includes the interquartile range (IQR) with median, together with the lower and upper $1.5 \times$ IQR whiskers. The colour indicates the number of introductions.

their virus sequenced and subsequent quarantine would completely prevent further transmission of imported variants.
For illustration, Figure SN.19 (top left) shows a simulation where introduced variants are not onwards transmitted. Unlike in Fig. SN.17 (left), where imported variants spread, we can see that the outbreak dynamics are not affected by the non-replicating introductions.
The corresponding ϕ estimates in Figure SN.19 (top right) show the following: In simulations, where 50 or 100 variants where introduced, the corresponding ϕ estimate remain quantitatively unchanged in comparison to a 'zero-introduction' simulation. In the extreme case, where 500, and even 1000 variants were introduced, (pink and green lines in Figure SN.19, top right), the dynamical profile of the number of infected individuals is still very closely captured (highlighting the 2.5 waves of infection). However, quantitatively the ϕ estimates are slightly increased in comparison to the 'zero-introduction' simulation. Thus, if one would like to compare a setting, where there are many non-replicating introductions, to a setting with replicating- or 'zero-' introductions, the quantitative interpretation of the ϕ estimates with regards to the true number of infected individuals N_{true} might differ to some extend. There may be a slightly different slope for the linear correlation function of ϕ vs. N_{true}.
In Figure SN.19 (bottom left), we systematically assess whether large relative amounts of non-replicating introductions decrease prediction accuracy, i.e. increase deviation from the linear regression function for the specific simulation. We do not discover a systematic trend, even if there were more non-replicating introductions than regionally transmitting viruses. We monitor the relative amount of introductions over time for the corresponding smoothed ϕ estimates in the panel above (Fig. SN.19, bottom right). Looking at the ratios of introductions vs. the effective population size per generation for the case of 1000 introductions (green line), we observe that the method is still able to reconstruct the dynamic and attain a linear relationship, despite having consecutively more than 15% introductions during the first wave and partly almost reaching the number of evolving sequences.

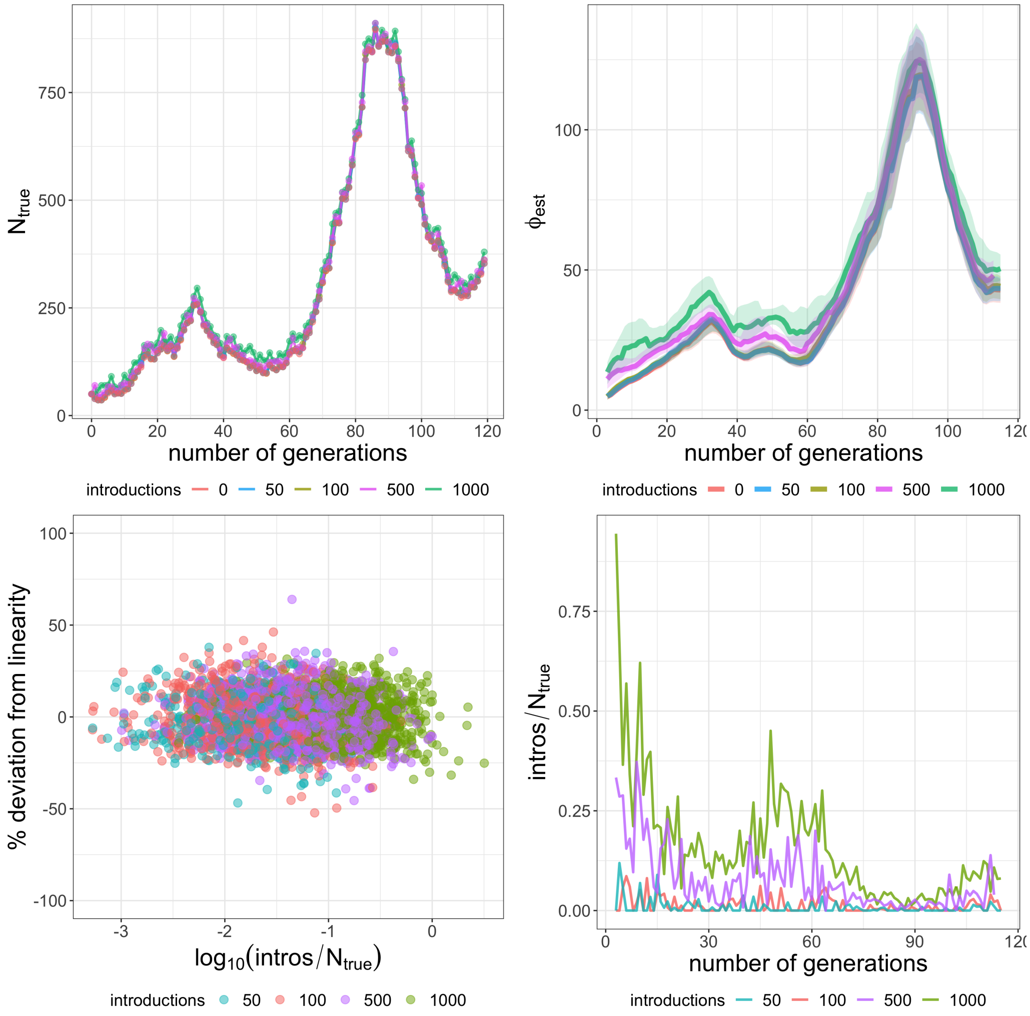

Figure SN.19: Simulations for introduced variants that do not spread in the population. The total amount of introduced sequences at random time points is colour coded. **Top left:** True population size over time for the original outbreak (0 introductions) and after introduction of new sequences for a representative simulation. **Top right:** The solid lines denote the smoothed median of the ϕ estimates for one stochastic simulation with different amounts of introductions. The shaded areas denote the 90% confidence interval. **Bottom left:** Dotplot showing the percent deviation of the smoothed ϕ estimates from the respective linear fit to N_{true} in relation to the relative frequency ($\log_{10}$) of introductions per time point. **Bottom right:** Relative amount of new introductions over time, corresponding to the simulated trajectories in the top right figure.

SN.1.13 Reconstruction of extreme (un-smooth) dynamics

To further test the limits of GInPipe, we set up two experiments with population dynamics that resemble step-functions. Obviously, these dynamics are quite unlikely to happen in reality and are therefore considered as extreme cases to push the pipeline to the limit.
In the first experiment, the population of infected individuals stochastically fluctuates around its initial value of ≈ 2000 for 60 generations, after which the population suddenly decreases by a factor 10 ($N(t = 61) = \mathcal{P}(N(t = 60) \cdot 0.1)$, Fig. SN.20) and henceforth remains around around a value of ≈ 200 for the remaining time points. In the second experiment, the population of infected individuals stochastically fluctuates around its initial value of ≈ 100 for 60 generations, after which it increases by a factor five ($N(t = 61) = \mathcal{P}(N(t = 60) \cdot 5)$, Fig. SN.21).
Fig. SN.20 (upper left) shows 10 simulations where the population sizes suddenly decrease by a factor 10. Fig. SN.20 (upper right) shows the corresponding smoothed median ϕ estimates and Fig. SN.20 (lower panels) shows two individual simulations and the corresponding incidence reconstructions.
Notably, while GInPipe generally reconstructs the population dynamics quite well, we do observe a "smearing out" when the population size changes drastically at generation 60, Fig. SN.20 (upper left vs. upper right). This "smearing out" is likely due to the binning of temporally adjacent sequences, and may be overcome by defining bins that do not span the region of abrupt change (if the latter was known).

Likewise, for the case of the step-function that increases the population size, Fig. SN.21 (upper left), we observe reasonable good incidence reconstructions, Fig. SN.21 (upper right). However, while many reconstructed incidence histories map the population dynamics reliably (example in Fig. SN.21, lower left), we can also observe more drastic delays in some simulations Fig. SN.21, lower right). This effect appears to be stronger for the step function increasing the populations size (Fig. SN.21), than for the step function decreasing the population size (Fig. SN.20). We believe that this effect is mainly due to a sampling bias with regards to the number of new mutations (i.e. in some simulations the new mutations appear later than the population size increases). In fact, such extreme dynamics could in theory appear if a single super-spreading event is responsible for the vast majority of new infections. In view of the spatial scales we analysed (countries or federal states) it is however unlikely that a single event dominates the pandemic. Our assessments of GInPipe for non-smooth population dynamics (step-wise changes of the number of infected individuals) revealed that the pipeline, with default setting may "smear out" abrupt changes in the number of infected individuals. For sudden changes that increase the number of infected individuals, temporal delays may be encountered. However, we have to note that this technical test may have limited relevance to a real-world application where the population of infected individuals changes rather smoothly over time.

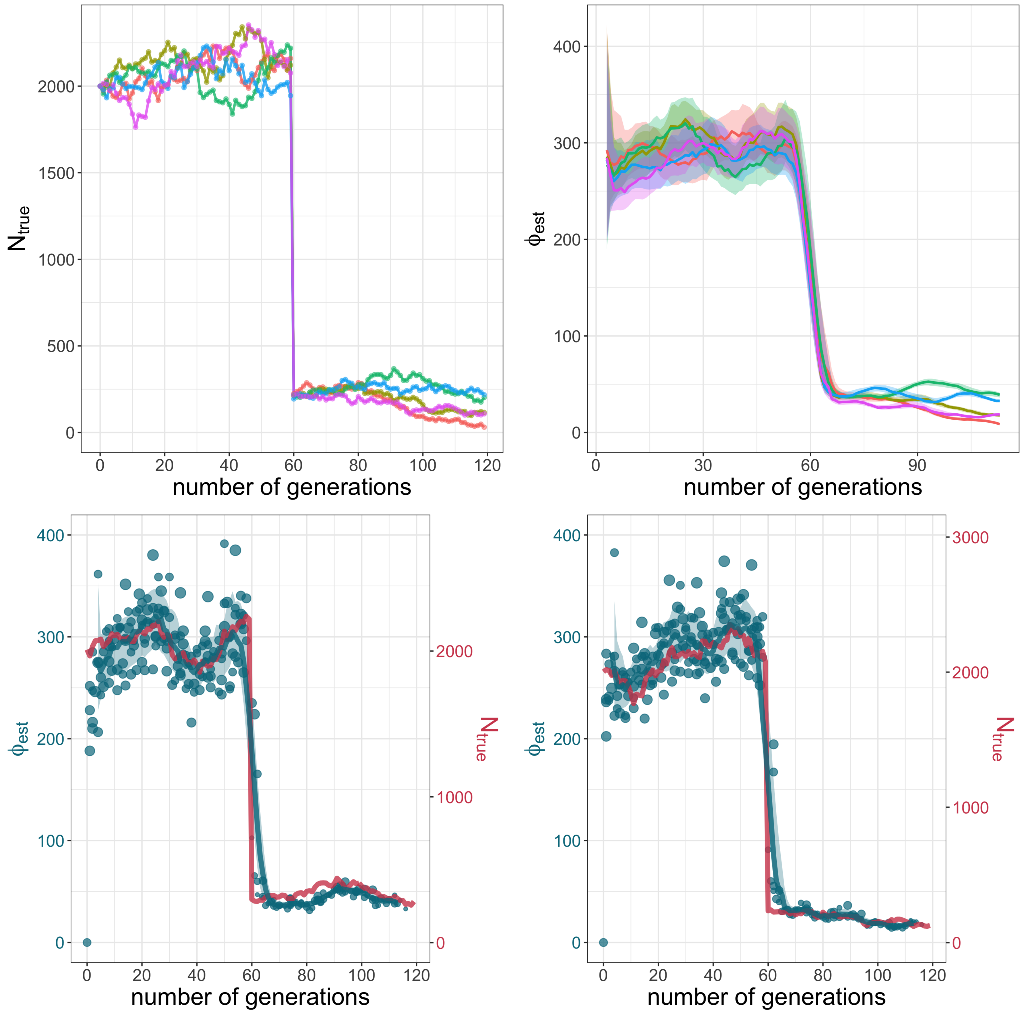

Figure SN.20: Simulations for a drastic decline of infected individuals. Top left: Simulated pandemic trajectories (5 simulations) starting with 3000 infected individuals that drop by a factor of ≈ 10 after 60 generations. **Top right:** The solid lines denote the smoothed median of the ϕ estimates for the respective stochastic simulations. The shaded areas denote the 90% confidence interval. **Bottom left and right:** Two individual simulations (red lines) and the corresponding incidence reconstructions (blue dots, lines (median) and shaded areas (90% confidence interval))

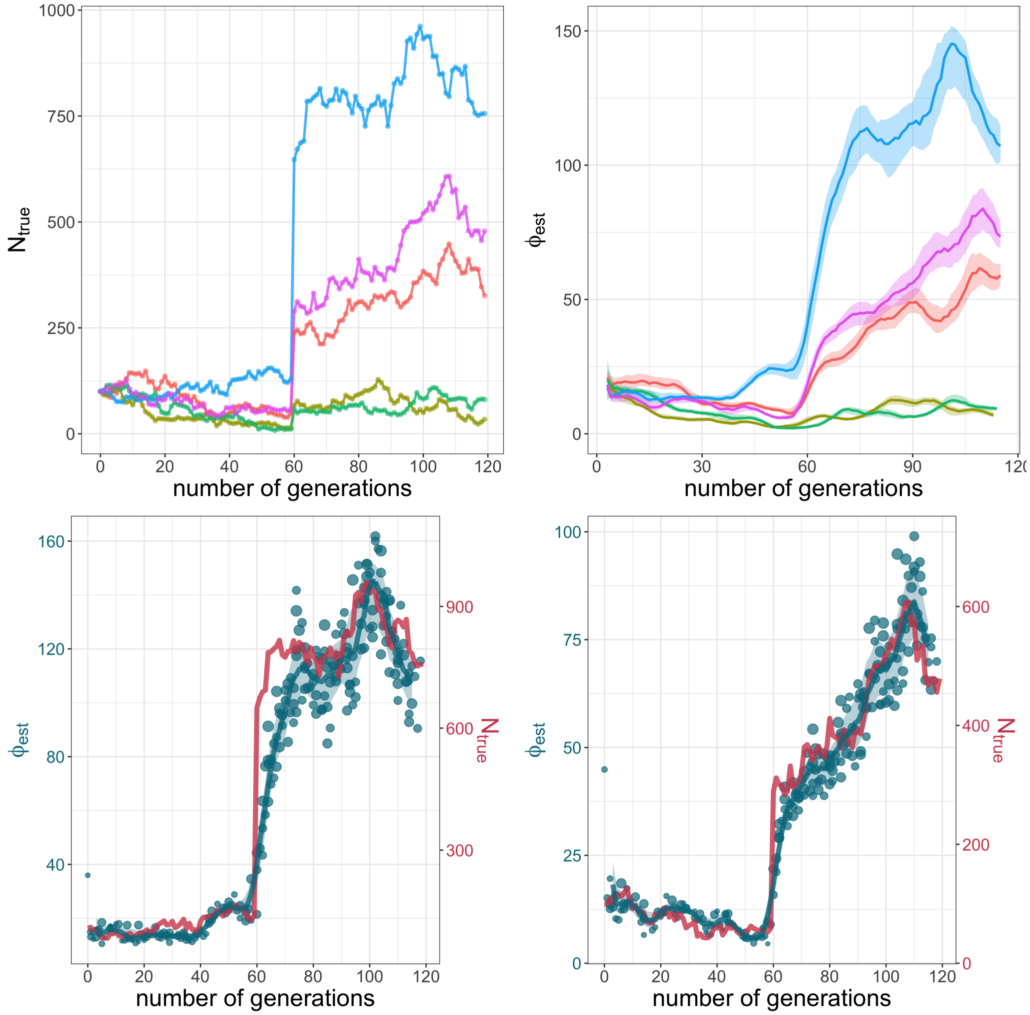

Figure SN.21: Simulations for a drastic increase of infected individuals. Top left: Simulated pandemic trajectories (5 simulations) starting with 100 infected individuals that increase by a factor of ≈ 5 after 60 generations. **Top right:** The solid lines denote the smoothed median of the ϕ estimates for the respective stochastic simulations. The shaded areas denote the 90% confidence interval. **Bottom left and right:** Two individual simulations (red lines) and the corresponding incidence reconstructions (blue dots, lines (median) and shaded areas (90% confidence interval))

SN.1.14 Effect of selective pressure on incidence reconstruction

In next scenario, we wanted to analyse if selection pressure affects GInPipe's ability to reconstruct the incidence history. To this end, we assign a fitness value $f(i, m_i)$ to each mutation m_i at position i. We then compute the fitness of a sequence f_s as the product of the fitness of all mutations contained in that sequence. Sequences s are then chosen to replicate based on their fitness, i.e. with probability $f_s / \sum_r f_r$. The mutations can either be beneficial (fitter than the wild type) or deleterious (lower survival chance than the wild type).

Individual fitness values were drawn from a log-normal distribution. To assess different levels of stochasticity and yet derive comparable simulations, we considered $\ell \in N_f \subset L$ positions to affect fitness (with $f(\ell, m_\ell) \neq 1$). The remaining positions $k \in L \setminus N_f$ were considered to be neutral ($f(k, m_k) = 1 =$ wild type fitness). We chose the single fitness values according to

$$f(\ell, m_\ell) = e^X, \text{ with } X \sim \mathcal{N}(0, \sigma^2) \tag{SN.9}$$

The fitness of a sequence s is given by the product the position-wise fitness values $f(i, m_i)$ in the respective nucleotide state m_i.

$$f_s = \prod_{i=1}^{L} f_s(i, m_i) \tag{SN.10}$$

$$= \prod_{i=1}^{L} \exp(X_i) \tag{SN.11}$$

$$= \exp(\sum_{i=1}^{L} X_i) \tag{SN.12}$$

Our total variance, which denotes the convolution of all mutation-wise variances, was set to $\sigma^2_{\text{tot}} = 0.2$. This setting was chosen, such that the overall change in fitness remains within a realistic range (e.g. the fitness advantage of the SARS-CoV-2 Delta variant over the wild type is assumed to be ≈ 2). Consequently, the variances of the individual fitness values $f(\ell, m_\ell)$ were set to $\sigma^2 = \frac{\sigma^2_{\text{tot}}}{N_f}$. For

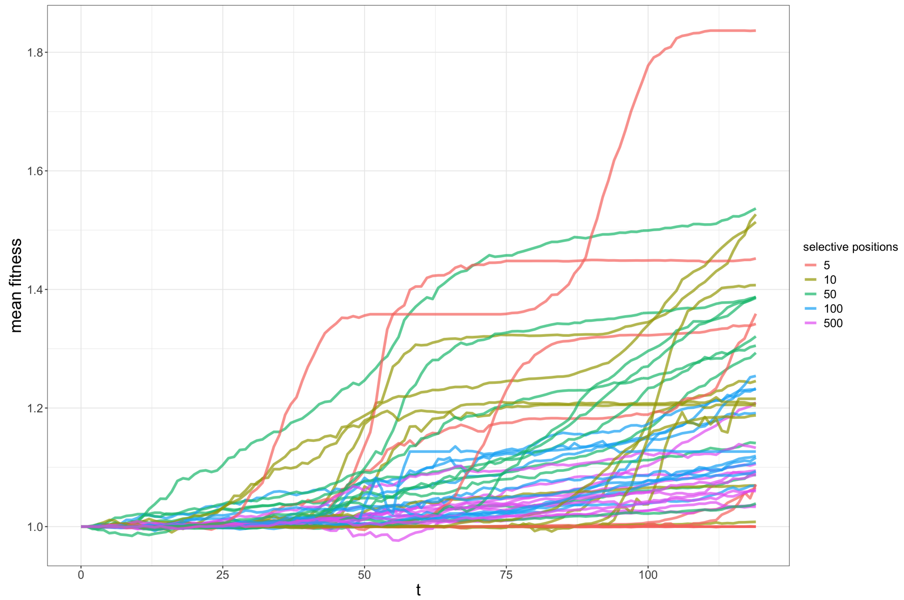

Figure SN.22: Population-average fitness for 10 simulations respectively with $N_f \in \{5, 10, 50, 100, 500\}$ selective sites.

illustration purposes, Figure SN.22 shows the *population average* fitness for 10 simulations each, using the evolutionary setting as above, with $N_f = \{5, 10, 50, 100, 500\}$ non-neutral sites. With the choice of a normalised variance, smaller numbers of non-neutral sites correspond to larger fitness changes per site, making the simulations with e.g. $N_f = 5$ more stochastic. On the contrary, with $N_f = 500$, each non-neutral mutation confers very small changes with regards to fitness, making the evolutionary adaptation more transient. The plot shows that the composition of the sequence set is driven by selective pressure: I.e. the sequence composition is changed such that the average fitness increases over time.

Fig. SN.23(top left) shows the simulated population dynamics using the evolutionary setting with $N_f = 50$ non-neutral sites. Fig. SN.23(top right) shows the GInPipe reconstructed incidence histories and Fig. SN.23(lower panels) shows the reconstructed incidence histories with different sub-sampling schemes (relative vs. absolute sub-sampling). As can be seen in Fig. SN.23, GInPipe can reconstruct incidence histories very well when particular positions are under selective pressure.

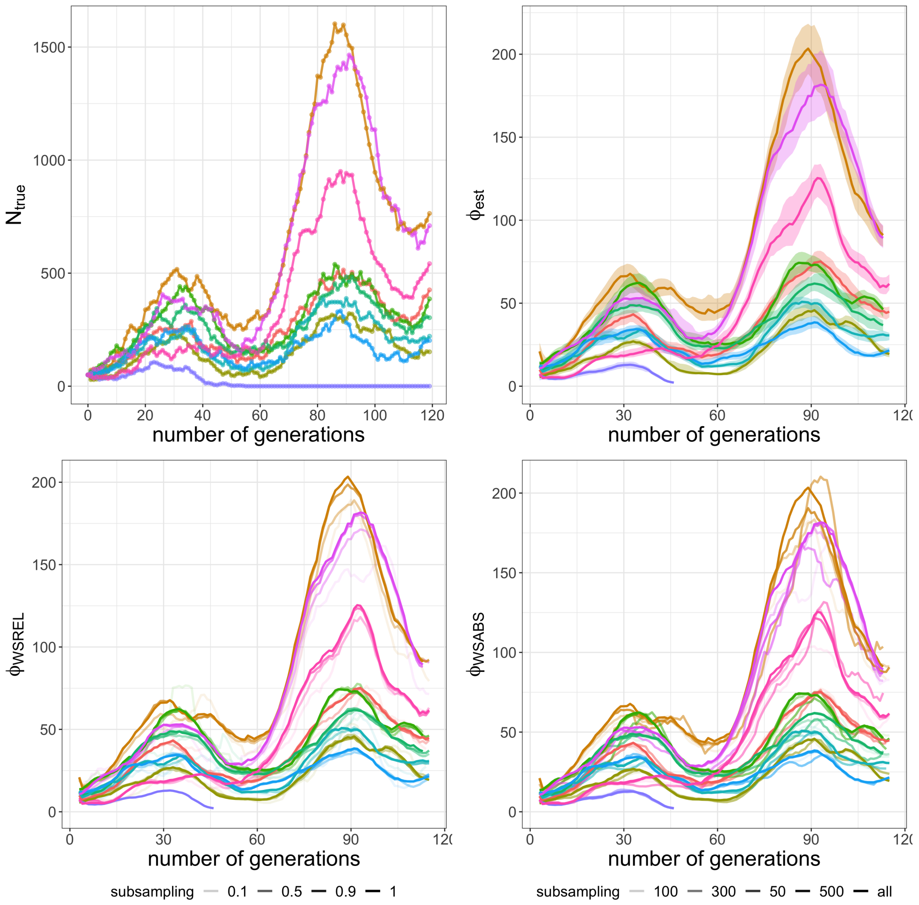

Figure SN.23: Simulations with selective pressure. Top left: Population dynamics with selection pressure. **Top right:** The solid lines denote the smoothed ϕ estimates using all sequence data. The shaded areas denote the 90% confidence interval. **Bottom left:** Smoothed ϕ estimates with relative sub-sampling. **Bottom right:** Smoothed ϕ estimates with absolute sub-sampling.

SN.1.15 Effect of mutation rate on incidence reconstruction

In this section we assess how changes in the mutation rate affect the incidence reconstruction with GInPipe. Note that a severely lowered mutation rate would decrease the evolutionary information contained in any sequence set and increase stochasticity, by turning evolutionary events into rare events. We performed simulations with the same setting as in section SN.1.3, but this time increased or decreased the mutation rate 10-fold.

10-fold lowed mutation rate. Simulations using a low mutation rate of $\mu = 0.00001$ are depicted in Fig. SN.24 (upper row, left most panel). Reconstructed incidence dynamics using GInPipe with default settings are shown in Fig. SN.24 using the full data set (upper row, center left panel), as well as sub-sampled data sets (upper row, the two panels on the right). For the full data set, GInPipe can still reconstruct the dynamics (Pearson correlation of $r = 0.9$), but for the sub-sampled data sets the reconstructions worsen (Pearson correlation coefficients of $r = 0.7-0.9$).

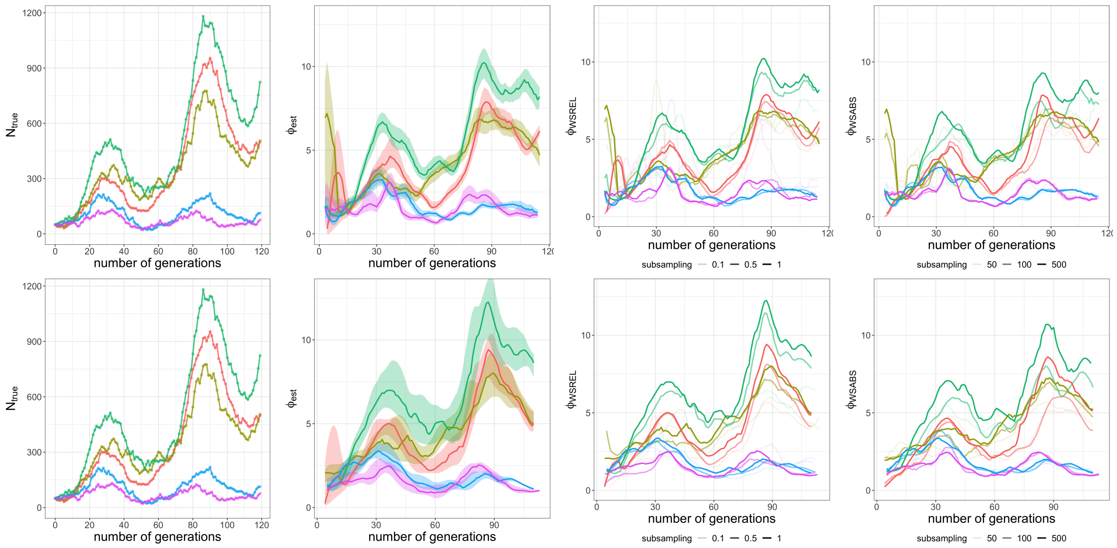

Figure SN.24: Incidence reconstructions with a 10-fold lowered mutation rate ($\mu = 0.00001$). Top row: Original binning strategy. **Bottom row:** Adjusted binning strategy. **Left:** Simulated pandemic trajectories (5 simulations). **Center left:** The solid lines denote the smoothed median of the ϕ estimates for the respective stochastic simulations using all sequences. The shaded areas denote the 90% confidence interval. **Center right and rightmost panel:** Smoothed median of the ϕ estimates for the respective stochastic simulations using subsets of the sequences (center right: relative subsampling, far right: uneven subsampling). The transparency of the curve indicates the level of subsampling.

Work-around: Increasing bin sizes. As mentioned above, for very low mutation rates, the binning strategy may produce sequence sets that contain no evolutionary information. Consequently, GInPipe may not robustly infer incidence estimates on these data sets. However, increasing the bin sizes may revert this situation. To demonstrate this presumption, we changed the binning strategy (compare to *Methods* section in the main manuscript) such that the bin sizes increase. In particular, we set: (i) the days per bin to $\{10, 15, 20, 25, 30\}$, (ii) the sequences per bin to $\{5, 10, 15, 20\}$ percent of the entire sequence set and adjusted the (iii) constraints, such that each bin must span at least 5 days and not more than 30 days and contain at least 20 sequences.

As can be seen in Fig. SN.24, this helped to recover the original dynamics (Pearson correlation of $r = 0.95$ (full data set), and 0.87–0.94 for the sub-sampled data sets), however at the cost of temporal resolution (e.g. the final increase in incidence cannot be recovered).

A comparison of the original binning strategy with the adjusted binning strategy (larger bins) is shown in Fig. SN.25 for one simulation. As can be seen therein (top left panel vs. bottom left panel), the original binning strategy has a much finer time resolution (number of blue dots).

As mentioned before, lowering the mutation rate increases stochasticity, and consequently decreases the signal-to-noise ratio. When sub-sampling the data using the original binning strategy Fig. SN.25 (top center and right panel), this can lead to scenarios where certain bins produce outlier dynamics (large shaded areas). When increasing the bin sizes Fig. SN.25 (bottom center and right panel), these large fluctuations due to extreme outliers could be eliminated.

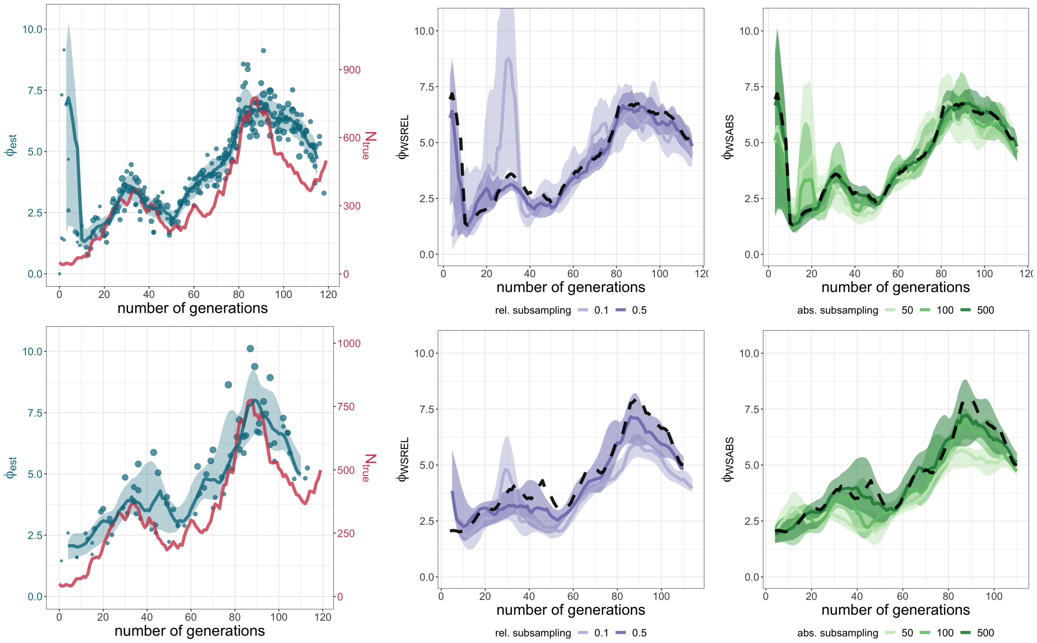

Figure SN.25: Example of incidence reconstructions for one simulation with a 10-fold decreased mutation rate (μ = 0.00001) using different binning strategies. Top row: Original binning strategy as stated in Section SN.1.3. **Bottom row:** Adapted binning strategy (larger bins). **Left**: Simulated population dynamics (red line) and GInPipe reconstructed incidence dynamics using all available data (blue dots: ϕ estimates, blue line: smoothed estimate). **Center**: Incidence reconstructions using relative sub-sampling. The transparency of the lines indicate the level of sub-sampling. **Right**: Incidence reconstructions using absolute sub-sampling. The transparency of the lines indicate the level of sub-sampling.

10-fold increased mutation rate. Simulations with a 10-fold increased mutation rate of $\mu = 0.001$ are shown in Fig. SN.26 (left) together with GInPipe's incidence reconstructions using the full data set (center left), as well as sub-sampled data sets (the two panels on the right). For a large mutation rate GInPipe can reconstruct the incidence very well (Pearson correlation of $r = 0.996$). For relative sub-sampling the incidence reconstructions are similarly good ($r > 0.95$), Fig. SN.26 (center right). For absolute sub-sampling Fig. SN.26 (rightmost panel) reconstructions are still very good ($r > 0.92$), except for the lowest absolute sub-sampling (50 sequences), where we observed $r = 0.81$.

Observations and Outlook

In summary, our simulations show that, particularly for low mutation rates, the 'evolutionary signal' decreases and that the emergence of novel haplotypes becomes more stochastic. In essence, this means that the signal-to-noise ratio decreases. The loss in evolutionary information can be circumvented by increasing the bin sizes, at the cost of temporal resolution. While the binning strategy presented in the *Methods* is well adapted to SARS-CoV-2, application of GInPipe to other respiratory infections would likely require to adapt the binning strategy to the data. As an outlook, we plan to build filters that automatically adapt binning strategies to the amount of

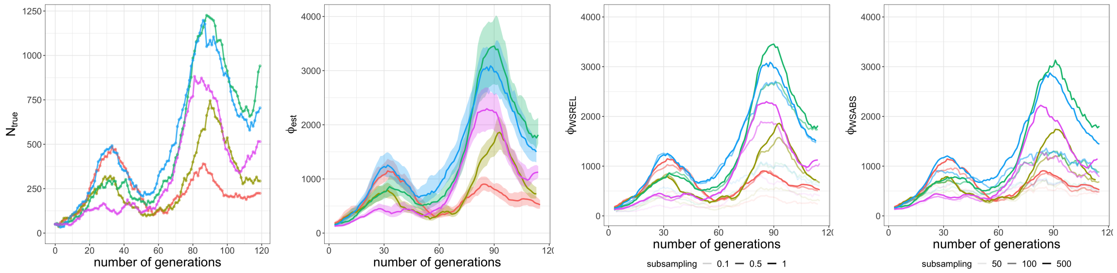

Figure SN.26: Incidence reconstructions with a 10-fold increased mutation rate ($\mu = 0.001$). Leftmost panel: Simulated pandemic trajectories (5 simulations). **Center left:** The solid lines denote the smoothed median incidence reconstructions using all sequences. The shaded areas denote the 90% confidence interval. **Center right and rightmost panel:** Smoothed median incidence reconstructions using subsets of the the data (center right: relative sub-sampling; right most: absolute sub-sampling). The transparency of the curve indicates the level of sub-sampling.

evolutionary information in a given data set, and which mark temporal periods where insufficient information is available.

Supplementary Note 2

In this Supplementary Note all parameters used in the phylodynamic inference are stated, such as (i) change dates where major non-pharmaceutical measures were introduced or lifted (these intervals are used to calculate the piece-wise constant $R_e(\tau)$ values); (ii) All final parameter estimates, as well as (iii) 'lineage-through-time' plots for phylodynamic reconstruction.

Birth-death-sampling rate change dates

**Date**	**Non-pharmaceutical interventions**
28 April 2020	Start of easing of restrictions: Opening of primary schools, later public places
15 June 2020	Further easing of restrictions: bigger private and mass gatherings allowed, home office partially recommended
22 August 2020	Protective masks: Face masks mandatory in closed public spaces
26 October 2020	Reimplementation of light restrictions: private gatherings
26 December 2020	Start of lockdown: closure of schools, restaurants and entertainment venues

Table SN.1: Denmark: Intervention measures used to set the birth-death-sampling rate change dates

Date	Non-pharmaceutical interventions
24 March 2020	Start of national lockdown: Closure of shops, restaurants etc, stay-at-home order
29 May 2020	First lifting of restrictions: Phase 1 of route map out of lockdown
15 July 2020	Phase 3 of route map out of lockdown: Opening restaurants, hairdressers etc.
23 September 2020	Reimplementation of restrictive measures: Restrictions on household visits and national curfew
17 November 2020	Further restrictions: Update on COVID-19 protection levels and travel regulations
05 January 2021	Start of second lockdown for mainland Scotland: stay-at-home order

Table SN.2: Scotland: Intervention measures used to set the birth-death-sampling rate change dates

Date	Non-pharmaceutical interventions
16 March 2020	Start of national lockdown: Closure of shops, museums, restaurants, other gathering places and borders
11 May 2020	Lifting of stricter lockdown measures: Opening of i.a. schools, shops and museums
15 June 2020	Second step in reopening the borders
20 August 2020	Unspecified
19 October 2020	Reimplementation of restrictions: home office recommendation, restrictions on private gatherings and for restaurants

Table SN.3: Switzerland: Intervention measures used to set the birth-death-sampling rate change dates

Date	Non-pharmaceutical interventions
16 March 2020	State of Emergency and first restrictions
11 May 2020	First easing of restrictions
19 July 2020	Reimplementation of restrictions, especially around Melbourne
13 September 2020	First step in roadmap for reopening
26 October 2020	Zero new cases recorded

Table SN.4: Victoria, Australia: Intervention measures used to set the birth-death-sampling rate change dates

Phylogenetic and epidemiological parameter estimates

	median	95% HPDI
become non−infectious rate δ_1	2.5625e+01	[2.3626e+01, 2.7618e+01]
become non−infectious rate δ_2	4.8634e+01	[4.6664e+01, 5.0612e+01]
become non−infectious rate δ_3	4.9078e+01	[4.7126e+01, 5.1035e+01]
become non−infectious rate δ_4	5.0413e+01	[4.8636e+01, 5.2326e+01]
become non−infectious rate δ_5	5.7918e+01	[5.6189e+01, 5.9674e+01]
become non−infectious rate δ_6	5.6963e+01	[5.5201e+01, 5.8822e+01]
reproductive number $R_{e,1}$	1.9810e+00	[1.7938e+00, 2.1807e+00]
reproductive number $R_{e,2}$	7.8879e−01	[6.4151e−01, 9.2349e−01]
reproductive number $R_{e,3}$	9.8603e−01	[9.2555e−01, 1.0466e+00]
reproductive number $R_{e,4}$	1.0571e+00	[1.0197e+00, 1.0928e+00]
reproductive number $R_{e,5}$	1.1692e+00	[1.1397e+00, 1.2008e+00]
reproductive number $R_{e,6}$	1.0611e+00	[1.0354e+00, 1.0884e+00]
sampling proportion s_1	0.0000e+00	[0.0000e+00, 0.0000e+00]
sampling proportion s_2	2.6598e−03	[1.0540e−03, 4.8091e−03]
sampling proportion s_3	3.2960e−04	[1.4211e−04, 5.7886e−04]
sampling proportion s_4	2.0136e−03	[1.3163e−03, 2.8224e−03]
sampling proportion s_5	8.8009e−03	[8.2494e−03, 9.0000e−03]
sampling proportion s_6	7.9828e−03	[7.9259e−03, 8.0000e−03]
sampling proportion s_7	1.2953e−02	[1.2798e−02, 1.3000e−02]
frequency parameter π_A	2.9857e−01	[2.9799e−01, 2.9912e−01]
frequency parameter π_C	1.8344e−01	[1.8295e−01, 1.8389e−01]
frequency parameter π_G	1.9603e−01	[1.9552e−01, 1.9649e−01]
frequency parameter π_T	3.2197e−01	[3.2140e−01, 3.2255e−01]
transition/transversion bias	6.0220e+00	[5.5656e+00, 6.5410e+00]
gamma shape parameter	6.5450e−01	[1.3687e−03, 3.0386e+00]
clock rate	8.0000e−04	[8.0000e−04, 8.0000e−04]

Table SN.5: Denmark: Median and 95% highest posterior density interval (HPDI) of the posterior sample of the model-specific phylogenetic and epidemiological parameters. Subscript of the latter indicates the interval for which it is inferred. Intervals are labelled numerically from the start of the process to the date of the last sample with boundaries as specified in Table SN.1
.

	median	95% HPDI
become non−infectious rate δ_1	2.6001e+01	[2.4104e+01, 2.8018e+01]
become non−infectious rate δ_2	4.9892e+01	[4.8056e+01, 5.1859e+01]
become non−infectious rate δ_3	4.8036e+01	[4.6171e+01, 5.0075e+01]
become non−infectious rate δ_4	5.0577e+01	[4.8539e+01, 5.2410e+01]
become non−infectious rate δ_5	5.7387e+01	[5.5567e+01, 5.9139e+01]
become non−infectious rate δ_6	5.4086e+01	[5.2269e+01, 5.6012e+01]
become non−infectious rate δ_7	4.9587e+01	[4.7647e+01, 5.1547e+01]
reproductive number $R_{e,1}$	2.3968e+00	[2.1690e+00, 2.6398e+00]
reproductive number $R_{e,2}$	9.6825e−01	[9.3264e−01, 1.0074e+00]
reproductive number $R_{e,3}$	8.8451e−01	[8.0327e−01, 9.6796e−01]
reproductive number $R_{e,4}$	1.4277e+00	[1.3730e+00, 1.4844e+00]
reproductive number $R_{e,5}$	9.5832e−01	[9.2419e−01, 9.9162e−01]
reproductive number $R_{e,6}$	1.2506e+00	[1.2013e+00, 1.2968e+00]
reproductive number $R_{e,7}$	1.2581e+00	[1.1723e+00, 1.3468e+00]
sampling proportion s_1	0.0000e+00	[0.0000e+00, 0.0000e+00]
sampling proportion s_2	4.3825e−02	[3.1846e−02, 5.7429e−02]
sampling proportion s_3	4.2829e−02	[3.6254e−02, 5.0000e−02]
sampling proportion s_4	7.2367e−03	[5.6379e−03, 7.9998e−03]
sampling proportion s_5	3.9707e−02	[3.3169e−02, 4.6317e−02]
sampling proportion s_6	9.9777e−03	[9.9019e−03, 1.0000e−02]
sampling proportion s_7	5.4729e−03	[5.3869e−03, 5.5000e−03]
sampling proportion s_8	7.3337e−03	[6.5973e−03, 7.6000e−03]
frequency parameter π_A	2.9858e−01	[2.9802e−01, 2.9907e−01]
frequency parameter π_C	1.8353e−01	[1.8309e−01, 1.8396e−01]
frequency parameter π_G	1.9605e−01	[1.9564e−01, 1.9659e−01]
frequency parameter π_T	3.2184e−01	[3.2130e−01, 3.2241e−01]
transition/transversion bias	6.0886e+00	[5.6013e+00, 6.6120e+00]
gamma shape parameter	7.1777e−01	[1.5681e−03, 3.0007e+00]
clock rate	8.0000e−04	[8.0000e−04, 8.0000e−04]

Table SN.6: Scotland: Median and 95% highest posterior density interval (HPDI) of the posterior sample of the model-specific phylogenetic and epidemiological parameters. Subscript of the latter indicates the interval for which it is inferred. Intervals are labelled numerically from the start of the process to the date of the last sample with boundaries as specified in Table SN.2
.

	median	95% HPDI
become non−infectious rate δ_1	2.6673e+01	[2.4635e+01, 2.8638e+01]
become non−infectious rate δ_2	4.9550e+01	[4.7621e+01, 5.1421e+01]
become non−infectious rate δ_3	4.8491e+01	[4.6607e+01, 5.0447e+01]
become non−infectious rate δ_4	4.9093e+01	[4.7222e+01, 5.1156e+01]
become non−infectious rate δ_5	5.4159e+01	[5.2311e+01, 5.5945e+01]
become non−infectious rate δ_6	5.3518e+01	[5.1617e+01, 5.5328e+01]
reproductive number $R_{e,1}$	3.7731e+00	[3.4827e+00, 4.0733e+00]
reproductive number $R_{e,2}$	6.0013e−01	[5.4912e−01, 6.5069e−01]
reproductive number $R_{e,3}$	1.0066e+00	[9.1301e−01, 1.0976e+00]
reproductive number $R_{e,4}$	1.0697e+00	[1.0268e+00, 1.1102e+00]
reproductive number $R_{e,5}$	1.2218e+00	[1.1859e+00, 1.2583e+00]
reproductive number $R_{e,6}$	1.2443e+00	[1.2194e+00, 1.2701e+00]
sampling proportion s_1	0.0000e+00	[0.0000e+00, 0.0000e+00]
sampling proportion s_2	2.8901e−02	[2.2041e−02, 3.6251e−02]
sampling proportion s_3	1.2186e−02	[1.0287e−02, 1.3000e−02]
sampling proportion s_4	5.6926e−03	[3.3451e−03, 8.4517e−03]
sampling proportion s_5	2.1886e−02	[1.7928e−02, 2.5921e−02]
sampling proportion s_6	1.0948e−02	[1.0777e−02, 1.1000e−02]
sampling proportion s_7	1.9905e−03	[1.9590e−03, 2.0000e−03]
frequency parameter π_A	2.9853e−01	[2.9787e−01, 2.9914e−01]
frequency parameter π_C	1.8344e−01	[1.8286e−01, 1.8392e−01]
frequency parameter π_G	1.9598e−01	[1.9550e−01, 1.9656e−01]
frequency parameter π_T	3.2206e−01	[3.2144e−01, 3.2270e−01]
transition/transversion bias	5.1855e+00	[4.8250e+00, 5.5949e+00]
gamma shape parameter	7.7207e−01	[1.0269e−03, 3.1043e+00]
clock rate	8.0000e−04	[8.0000e−04, 8.0000e−04]

Table SN.7: Switzerland: Median and 95% highest posterior density interval (HPDI) of the posterior sample of the model-specific phylogenetic and epidemiological parameters. Subscript of the latter indicates the interval for which it is inferred. Intervals are labelled numerically from the start of the process to the date of the last sample with boundaries as specified in Table SN.3
.

	median	95% HPDI
become non−infectious rate δ_1	2.6169e+01	[2.4199e+01, 2.8134e+01]
become non−infectious rate δ_2	4.9617e+01	[4.7653e+01, 5.1587e+01]
become non−infectious rate δ_3	5.1130e+01	[4.9280e+01, 5.3008e+01]
become non−infectious rate δ_4	5.3928e+01	[5.2086e+01, 5.5703e+01]
become non−infectious rate δ_5	4.8734e+01	[4.6809e+01, 5.0700e+01]
become non−infectious rate δ_6	4.8756e+01	[4.6749e+01, 5.0668e+01]
reproductive number $R_{e,1}$	1.4985e+00	[1.3534e+00, 1.6480e+00]
reproductive number $R_{e,2}$	8.5330e−01	[7.5130e−01, 9.2257e−01]
reproductive number $R_{e,3}$	1.5831e+00	[1.5003e+00, 1.6653e+00]
reproductive number $R_{e,4}$	8.2566e−01	[7.7671e−01, 8.7320e−01]
reproductive number $R_{e,5}$	2.3464e−01	[3.3521e−07, 5.6699e−01]
reproductive number $R_{e,6}$	2.0824e+00	[1.3328e+00, 2.9249e+00]
sampling proportion $s_{g,1}$	0.0000e+00	[0.0000e+00, 0.0000e+00]
sampling proportion $s_{g,2}$	2.9634e−02	[1.9242e−02, 4.1241e−02]
sampling proportion $s_{g,3}$	1.7328e−01	[1.3147e−01, 2.1603e−01]
sampling proportion $s_{g,4}$	6.5030e−02	[4.3376e−02, 9.1276e−02]
sampling proportion $s_{g,5}$	2.2637e−01	[8.7877e−02, 4.1711e−01]
sampling proportion $s_{g,6}$	1.6848e−01	[8.4786e−03, 4.3823e−01]
sampling proportion $s_{g,7}$	1.7360e−02	[2.1027e−08, 1.0043e−01]
sampling proportion $s_{D.2,1}$	0.0000e+00	[0.0000e+00, 0.0000e+00]
sampling proportion $s_{D.2,2}$	5.0018e−02	[4.0377e−03, 9.8919e−02]
sampling proportion $s_{D.2,3}$	2.5750e−02	[1.6234e−07, 8.5113e−02]
sampling proportion $s_{D.2,4}$	9.9060e−02	[9.6127e−02, 1.0000e−01]
sampling proportion $s_{D.2,5}$	9.9453e−02	[9.7659e−02, 1.0000e−01]
sampling proportion $s_{D.2,6}$	8.5009e−02	[5.6538e−02, 9.9999e−02]
sampling proportion $s_{D.2,7}$	4.9863e−02	[1.6285e−06, 9.4899e−02]
frequency parameter π_A	2.9860e−01	[2.9781e−01, 2.9947e−01]
frequency parameter π_C	1.8357e−01	[1.8284e−01, 1.8423e−01]
frequency parameter π_G	1.9609e−01	[1.9536e−01, 1.9677e−01]
frequency parameter π_T	3.2173e−01	[3.2082e−01, 3.2255e−01]
transition/transversion bias	6.3017e+00	[5.4814e+00, 7.2111e+00]
gamma shape parameter	7.1495e−01	[1.0263e−03, 3.0786e+00]
clock rate	8.0000e−04	[8.0000e−04, 8.0000e−04]

Table SN.8: Victoria: Median and 95% highest posterior density interval (HPDI) of the posterior sample of the model-specific phylogenetic and epidemiological parameters. Subscript of the latter indicates the interval for which it is inferred. Intervals are labelled numerically from the start of the process to the date of the last sample with boundaries as specified in Table SN.4
.

Identified Pango lineages and tree reconstructions

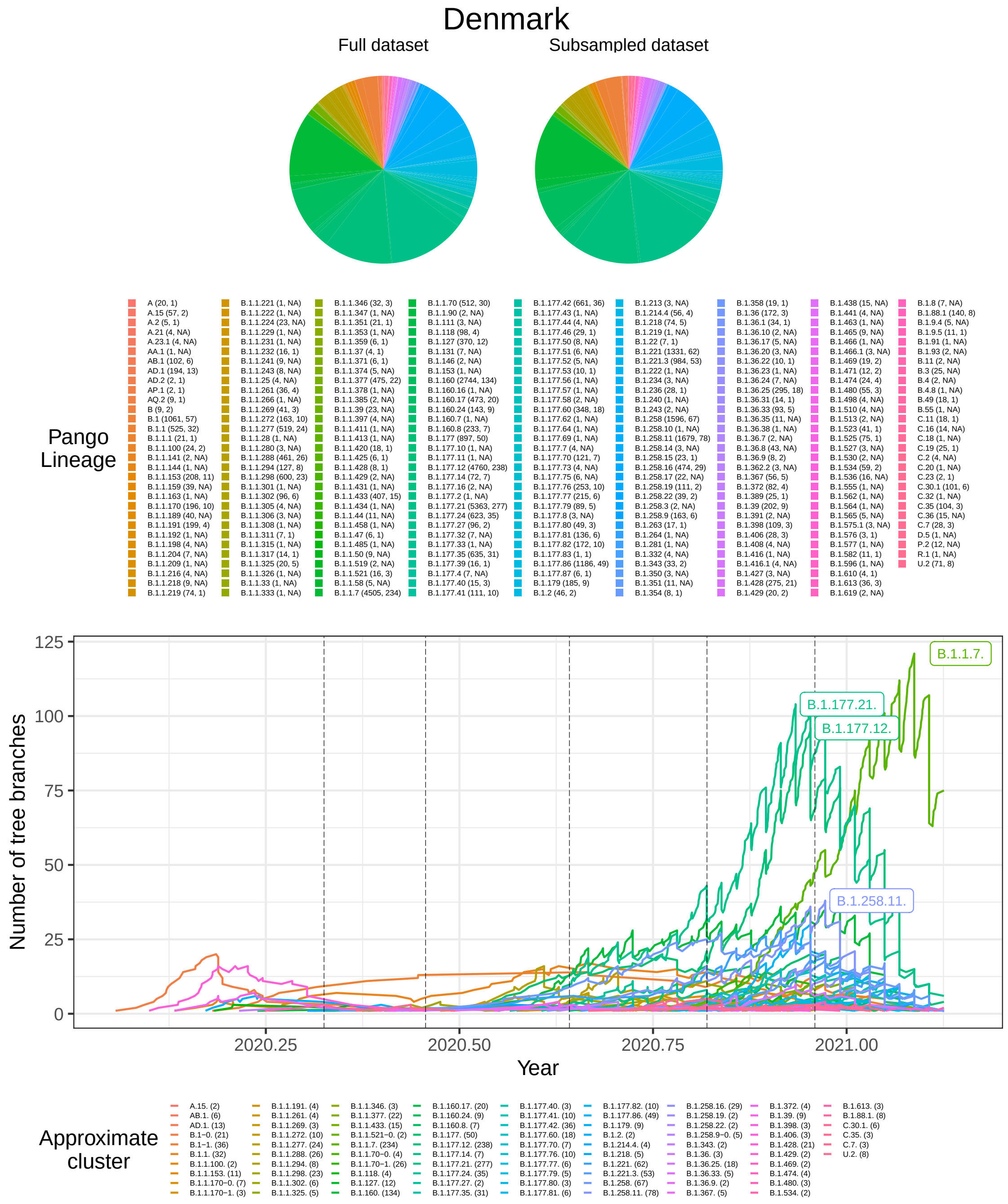

Figure SN.27: Denmark: Top: Pango lineages identified in the full (left) and subsampled (right) data set with the coloured area being proportional to the size of the specific lineage. Values in brackets next to the lineage name in the legend represent the number of sequences identified in the full (left) and subsampled (right) data set. Bottom: Lineage-through-time plot, counting the number of phylogenetic tree branches over time, of maximum clade credibility tree for all identified approximate clusters. Pango lineages that were split due to a longer period without any sample are denoted numerically using a dash separator, e.g. B.1-0 and B.1-1. Vertical dotted lines represent the dates at which birth, death and sampling rate were allowed to change. The number of sequences included for each approximate cluster in the analysis are given in brackets next to the cluster name in the legend.

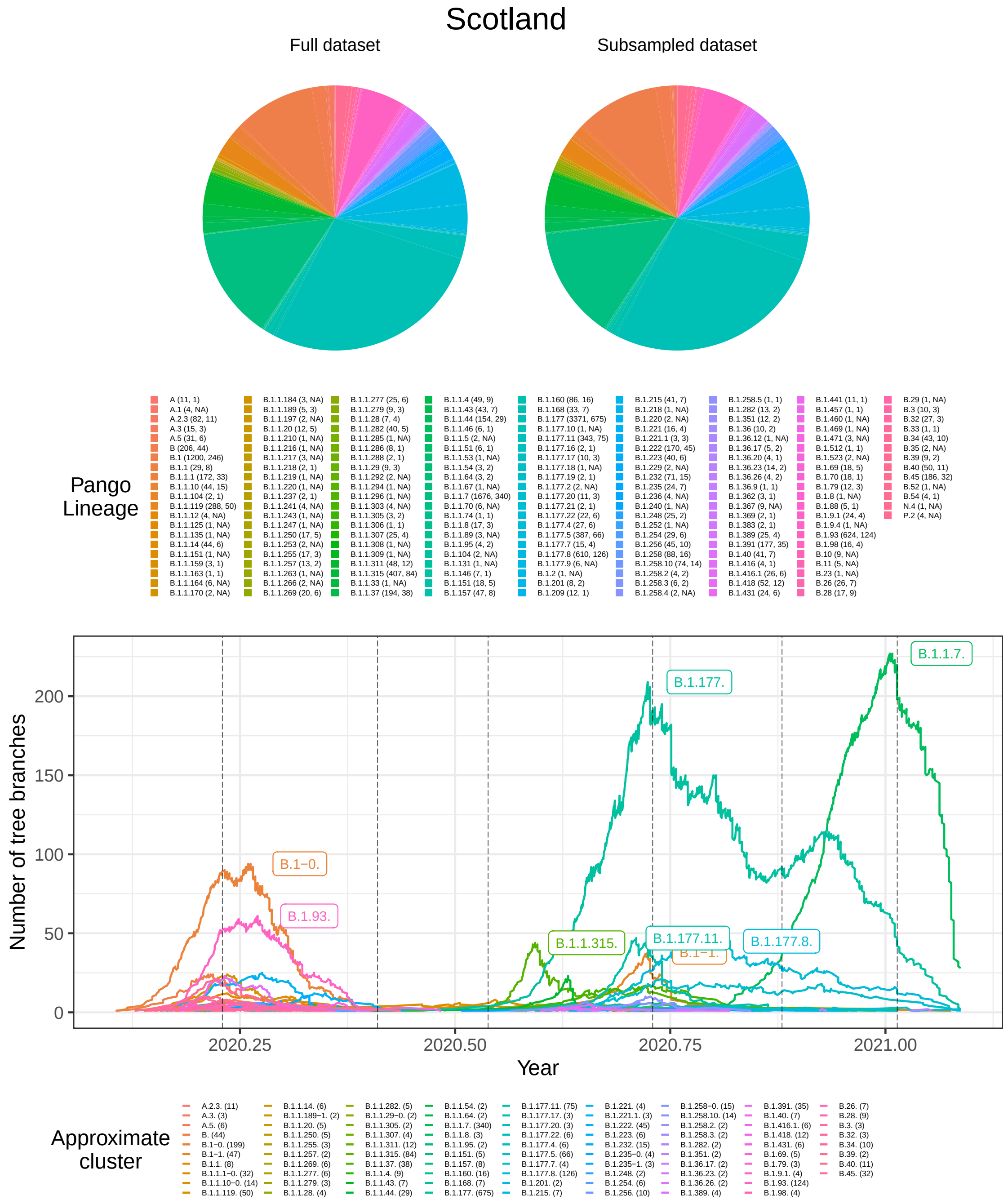

Figure SN.28: Scotland: Top: Pango lineages identified in the full (left) and subsampled (right) data set with the coloured area being proportional to the size of the specific lineage. Values in brackets next to the lineage name in the legend represent the number of sequences identified in the full (left) and subsampled (right) data set. Bottom: Lineage-through-time plot, counting the number of phylogenetic tree branches over time, of maximum clade credibility tree for all identified approximate clusters. Pango lineages that were split due to a longer period without any sample are denoted numerically using a dash separator, e.g. B.1-0 and B.1-1. Vertical dotted lines represent the dates at which birth, death and sampling rate were allowed to change. The number of sequences included for each approximate cluster in the analysis are given in brackets next to the cluster name in the legend.

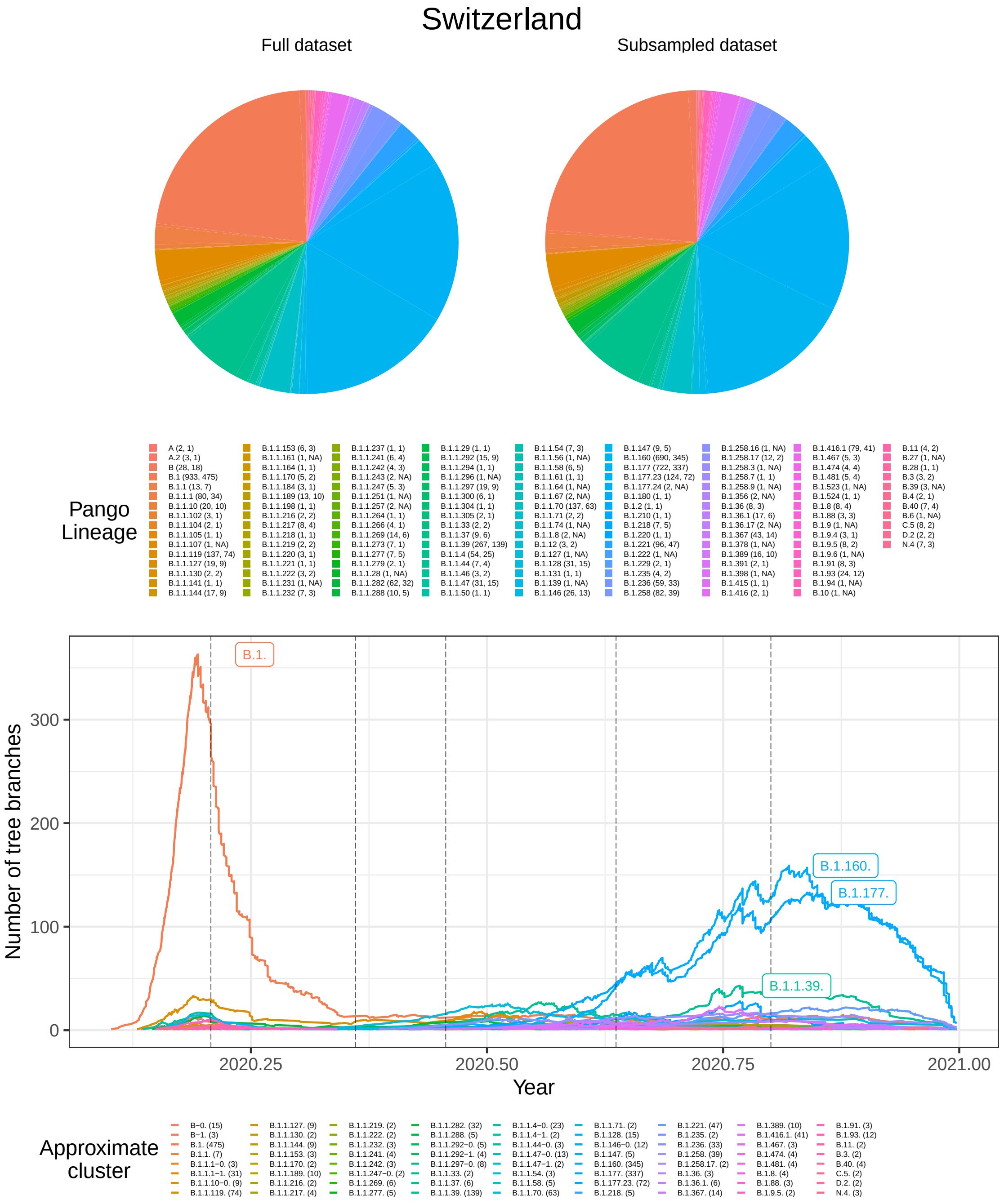

Figure SN.29: Switzerland: Top: Pango lineages identified in the full (left) and subsampled (right) data set with the coloured area being proportional to the size of the specific lineage. Values in brackets next to the lineage name in the legend represent the number of sequences identified in the full (left) and subsampled (right) data set. Bottom: Lineage-through-time plot, counting the number of phylogenetic tree branches over time, of maximum clade credibility tree for all identified approximate clusters. Pango lineages that were split due to a longer period without any sample are denoted numerically using a dash separator, e.g. B-0 and B-1. Vertical dotted lines represent the dates at which birth, death and sampling rate were allowed to change. The number of sequences included for each approximate cluster in the analysis are given in brackets next to the cluster name in the legend.

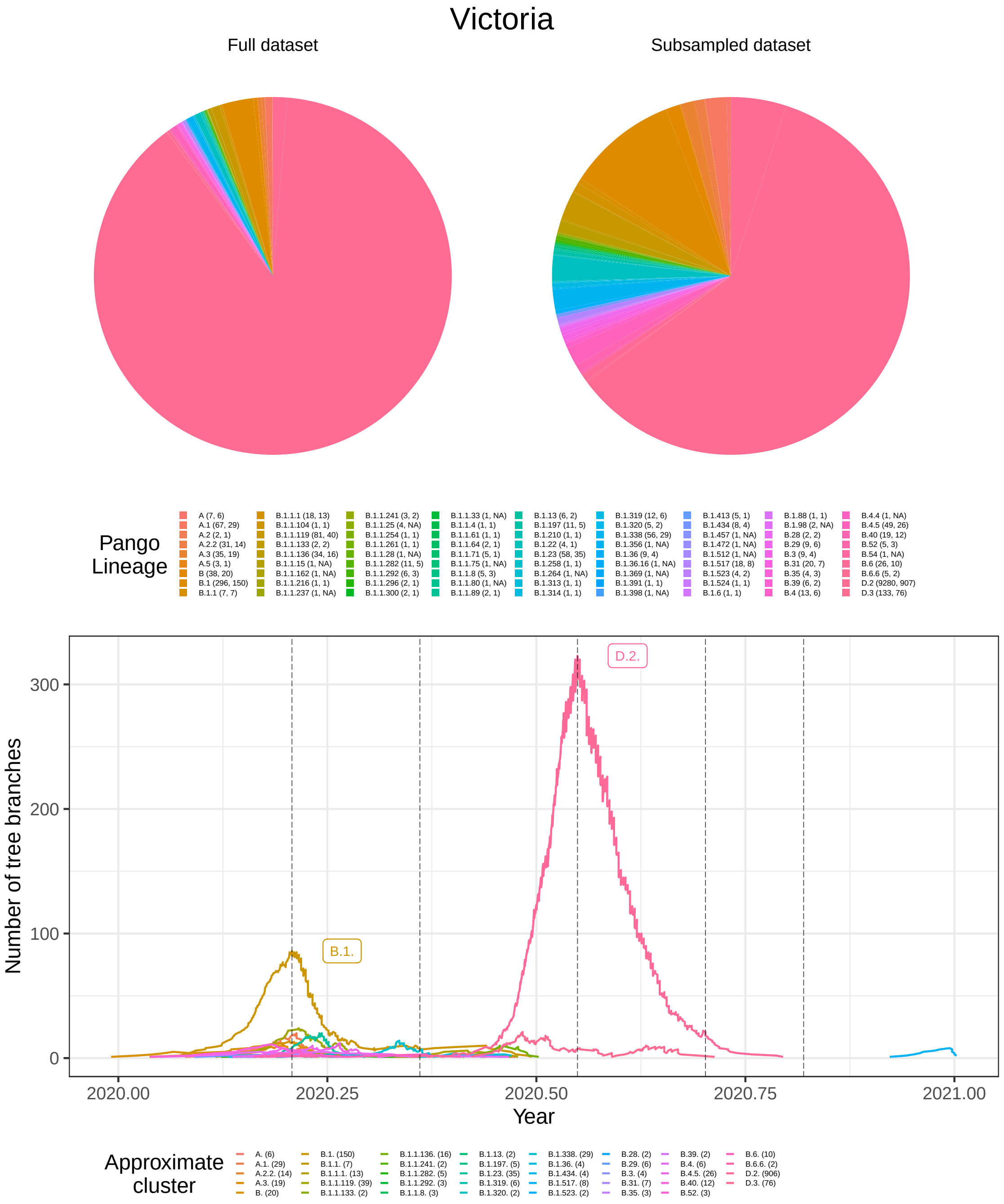

Figure SN.30: Victoria, Australia: Top: Pango lineages identified in the full (left) and subsampled (right) data set with the coloured area being proportional to the size of the specific lineage. Values in brackets next to the lineage name in the legend represent the number of sequences identified in the full (left) and subsampled (right) data set. Due to the lineage-specific subsampling procedure, the relative lineage compostion is different between the full and the subsampled dataset. Bottom: Lineage-through-time plot, counting the number of phylogenetic tree branches over time, of maximum clade credibility tree for all identified approximate clusters. Vertical dotted lines represent the dates at which birth, death and sampling rate were allowed to change. The number of sequences included for each approximate cluster in the analysis are given in brackets next to the cluster name in the legend.

Supplementary Note 3

This Supplementary Note contains all weblinks that point to the primary data depicted or discussed in the main manuscript. For example, the data related to (i) changes in non-pharmaceutical interventions, which are used in BEAST2; (ii) case reporting data which is depicted in Fig. 3 (main article); (iii) the number of tests performed and the proportion of positive tests, which are used to calculate the proportion of detected cases; and (iv) changes in testing strategies, which are depicted in Fig. 4 (main article).

Supplementary Links

Changes in non-pharmaceutical interventions

The data sources stated herein are used to determine change dates for phylodynamic reconstruction. The latter is used to compute R_e values that are depicted in Fig. 2 (main manuscript).

Denmark and Switzerland (Phylodynamics). WHO Coronavirus (COVID-19) Dashboard. Link: `https://covid19.who.int/table`, (accessed: 2021-03-10)

Victoria (Phylodynamics). Victorian coronavirus (COVID-19) data. Link: `https://www.dhhs.vic.gov.au/victorian-coronavirus-covid-19-data`, accessed: 2021-03-10)

Scotland (Phylodynamics). Coronavirus (COVID-19): trends in daily data. Link: `https://www.gov.scot/publications/coronavirus-covid-19-trends-in-daily-data`, (accessed: 2021-03-10)

Case reports

Reported SARS-CoV-2 infections are depicted in Fig. 3 (main manuscript) alongside the reconstructed incidence histories using GInPipe.

Denmark and Switzerland. Daily new reported cases. Link: `https://github.com/owid/covid-19-data/tree/master/public/data`, (accessed: 2021-04-27)

Scotland. New reported cases by date. Link: `https://coronavirus.data.gov.uk/details/download`, (accessed: 2021-04-27)

Victoria (AU). Daily new reported cases by state. Link: `https://public.tableau.com/profile/vicdhhs#!/vizhome/Cases_15982342702770/DashCasesGSG`, (accessed: 2021-04-27)

Number of tests performed and the test positivity

This data is used in Fig. 4 (main manuscript) to compute the case detection rate as outlined in the *Methods* section.

Denmark. A cross-country database of COVID-19 testing. Link: `https://covid.ourworldindata.org/data/owid-covid-data.csv`, (accessed: 2021-03-11)

Scotland. Coronavirus (COVID-19): trends in daily data. Link: `https://www.gov.scot/publications/coronavirus-covid-19-trends-in-daily-data`, (accessed: 2021-03-11)

Switzerland. A cross-country database of COVID-19 testing . Link: `https://covid.ourworldindata.org/data/owid-covid-data.csv`, (accessed: 2021-03-11)

Victoria. Testing for COVID-19. Link: https://www.covid19data.com.au/testing, (accessed: 2021-03-19)

Testing Strategy

Data on major changes in testing strategies are depicted in Fig. 4 as vertical dashed lines.
Denmark. Policy responses for Denmark. Link: https://www.covid19healthsystem.org/countries/denmark/livinghit.aspx?Section=1.5%20Testing&Type=Section, (accessed: 2021-03-11)
Scotland. Timeline of Coronavirus (COVID-19) in Scotland. Link: https://spice-spotlight.scot/2021/03/12/timeline-of-coronavirus-covid-19-in-scotland, (accessed: 2021-03-11)
Switzerland. Policy responses for Switzerland (Covid-19 Health System Response Monitor). Link: https://www.covid19healthsystem.org/countries/switzerland/livinghit.aspx?Section=1.5%20Testing&Type=Section, (accessed: 2021-03-18)
Victoria. Updates about the outbreak of the coronavirus disease (COVID-19). Link: https://www.dhhs.vic.gov.au/coronavirus/updates, (accessed: 2021-03-11)

Supplementary Note 4

GISAID acknowledgement tables

Denmark

We gratefully acknowledge the following Authors from the Originating laboratories responsible for obtaining the specimens, as well as the Submitting laboratories where the genome data were generated and shared via GISAID, on which this research is based.

All Submitters of data may be contacted directly via www.gisaid.org

Authors are sorted alphabetically.

Accession ID	Originating Laboratory	Submitting Laboratory	Authors
EPI_ISL_1065158, EPI_ISL_1065476, EPI_ISL_1065490, EPI_ISL_1065547, EPI_ISL_1065586, EPI_ISL_1065725, EPI_ISL_1065843, EPI_ISL_1065879, EPI_ISL_1066055, EPI_ISL_1066329, EPI_ISL_1066354, EPI_ISL_1066434, EPI_ISL_1066442, EPI_ISL_1066894, EPI_ISL_1066900, EPI_ISL_1066966, EPI_ISL_1067042, EPI_ISL_1067536			
see above	Department of Virus and Microbiological Special Diagnostics, Statens Serum Institut, Copenhagen, Denmark	Aalborg University	Danish Covid-19 Genome Consortium
EPI_ISL_415646	Department of Virus and Microbiological Special diagnostics, Statens Serum Institut, Copenhagen, Denmark.	ViFU	Morten Rasmussen, Maiken Worsoe Rosenstierne , Anders Fomsgaard
EPI_ISL_415647	Department of Virus and Microbiological Special diagnostics, Statens Serum Institut, Copenhagen, Denmark.	Statens Serum Institute	Morten Rasmussen, Maiken Worsoe Rosenstierne , Anders Fomsgaard
EPI_ISL_415648	Department of Virus and Microbiological Special diagnostics, Statens Serum Institut, Copenhagen, Denmark.	ViFU	Morten Rasmussen, Maiken Worsoe Rosenstierne , Anders Fomsgaard
EPI_ISL_416140, EPI_ISL_416141, EPI_ISL_416142	Department of Virus and Microbiological Special diagnostics, Statens Serum Institut, Copenhagen, Denmark.	Statens Serum Institute	Morten Rasmussen, Maiken Worsoe Rosenstierne , Anders Fomsgaard
EPI_ISL_416143, EPI_ISL_416144, EPI_ISL_416153	Department of Virus and Microbiological Special diagnostics, Statens Serum Institut, Copenhagen, Denmark.	ViFU	Morten Rasmussen, Maiken Worsoe Rosenstierne , Anders Fomsgaard
EPI_ISL_429262, EPI_ISL_429263, EPI_ISL_429264, EPI_ISL_429265, EPI_ISL_429266, EPI_ISL_429267, EPI_ISL_429268, EPI_ISL_429269, EPI_ISL_429270, EPI_ISL_429271, EPI_ISL_429272, EPI_ISL_429273, EPI_ISL_429274, EPI_ISL_429275, EPI_ISL_429276, EPI_ISL_429277, EPI_ISL_429278, EPI_ISL_429279, EPI_ISL_429280, EPI_ISL_429281, EPI_ISL_429282, EPI_ISL_429283, EPI_ISL_429284, EPI_ISL_429285, EPI_ISL_429286, EPI_ISL_429287, EPI_ISL_429288, EPI_ISL_429289, EPI_ISL_429290, EPI_ISL_429291, EPI_ISL_429292, EPI_ISL_429293, EPI_ISL_429294, EPI_ISL_429295, EPI_ISL_429296, EPI_ISL_429297, EPI_ISL_429298, EPI_ISL_429299, EPI_ISL_429300, EPI_ISL_429301, EPI_ISL_429302, EPI_ISL_429303, EPI_ISL_429304, EPI_ISL_429305, EPI_ISL_429306, EPI_ISL_429307, EPI_ISL_429308, EPI_ISL_429309, EPI_ISL_429310, EPI_ISL_429311, EPI_ISL_429312, EPI_ISL_429313, EPI_ISL_429314, EPI_ISL_429315, EPI_ISL_429316, EPI_ISL_429317, EPI_ISL_429318, EPI_ISL_429320, EPI_ISL_429321, EPI_ISL_429322, EPI_ISL_429323, EPI_ISL_429324, EPI_ISL_429325, EPI_ISL_429326, EPI_ISL_429327, EPI_ISL_429328, EPI_ISL_429329, EPI_ISL_429330, EPI_ISL_429331, EPI_ISL_429332			
see above	Department of Clinical Microbiology, Copenhagen University Hospital, Hvidovre, Kettegaard Alle 30, 2650 Hvidovre.	Albertsen lab, Department of Chemistry and Bioscience, Aalborg University, Denmark	Rasmus Kirkegaard
EPI_ISL_429333, EPI_ISL_429334, EPI_ISL_429335, EPI_ISL_429336, EPI_ISL_429337, EPI_ISL_429338, EPI_ISL_429339, EPI_ISL_429340, EPI_ISL_429341, EPI_ISL_429342, EPI_ISL_429343, EPI_ISL_429344, EPI_ISL_429345, EPI_ISL_429346, EPI_ISL_429347, EPI_ISL_429348, EPI_ISL_429349, EPI_ISL_429350, EPI_ISL_429351, EPI_ISL_429352, EPI_ISL_429353, EPI_ISL_429354, EPI_ISL_429355, EPI_ISL_429356, EPI_ISL_429357, EPI_ISL_429358, EPI_ISL_429359, EPI_ISL_429360, EPI_ISL_429361, EPI_ISL_429362, EPI_ISL_429363, EPI_ISL_429364, EPI_ISL_429365, EPI_ISL_429366, EPI_ISL_429367, EPI_ISL_429368, EPI_ISL_429369, EPI_ISL_429370, EPI_ISL_429371, EPI_ISL_429372, EPI_ISL_429373, EPI_ISL_429374, EPI_ISL_429375, EPI_ISL_429376, EPI_ISL_429377, EPI_ISL_429378, EPI_ISL_429379, EPI_ISL_429380, EPI_ISL_429381, EPI_ISL_429382, EPI_ISL_429383, EPI_ISL_429384, EPI_ISL_429385, EPI_ISL_429386, EPI_ISL_429387, EPI_ISL_429388, EPI_ISL_429389, EPI_ISL_429390, EPI_ISL_429391, EPI_ISL_429392, EPI_ISL_429393, EPI_ISL_429394, EPI_ISL_429395, EPI_ISL_429396, EPI_ISL_429397, EPI_ISL_429398, EPI_ISL_429399, EPI_ISL_429400, EPI_ISL_429401, EPI_ISL_429402, EPI_ISL_429403, EPI_ISL_429404, EPI_ISL_429405, EPI_ISL_429406, EPI_ISL_429407, EPI_ISL_429408, EPI_ISL_429409, EPI_ISL_429410, EPI_ISL_429411, EPI_ISL_429412, EPI_ISL_429413, EPI_ISL_429414, EPI_ISL_429415, EPI_ISL_429416, EPI_ISL_429417, EPI_ISL_429418, EPI_ISL_429419, EPI_ISL_429420, EPI_ISL_429421, EPI_ISL_429422, EPI_ISL_429423, EPI_ISL_429424, EPI_ISL_429425, EPI_ISL_429426, EPI_ISL_429427, EPI_ISL_429428, EPI_ISL_429429, EPI_ISL_429430, EPI_ISL_429431, EPI_ISL_429432, EPI_ISL_429433, EPI_ISL_429434, EPI_ISL_429435, EPI_ISL_429436, EPI_ISL_429437, EPI_ISL_429438, EPI_ISL_429439, EPI_ISL_429440, EPI_ISL_429441, EPI_ISL_429442, EPI_ISL_429443, EPI_ISL_429444, EPI_ISL_429445, EPI_ISL_429446, EPI_ISL_429447, EPI_ISL_429448, EPI_ISL_429449, EPI_ISL_429450, EPI_ISL_429451, EPI_ISL_429452, EPI_ISL_429453, EPI_ISL_429454, EPI_ISL_429455, EPI_ISL_429456, EPI_ISL_429457, EPI_ISL_429458, EPI_ISL_429459, EPI_ISL_429460, EPI_ISL_429461, EPI_ISL_429462, EPI_ISL_429463, EPI_ISL_429464, EPI_ISL_429465, EPI_ISL_429466, EPI_ISL_429467, EPI_ISL_429468, EPI_ISL_429469, EPI_ISL_429470, EPI_ISL_429471, EPI_ISL_429472, EPI_ISL_429473, EPI_ISL_429474, EPI_ISL_429475, EPI_ISL_429476, EPI_ISL_429477, EPI_ISL_429478, EPI_ISL_429479, EPI_ISL_429480, EPI_ISL_429481, EPI_ISL_429482, EPI_ISL_429483, EPI_ISL_429484, EPI_ISL_429485, EPI_ISL_429486, EPI_ISL_429487, EPI_ISL_429488, EPI_ISL_429489, EPI_ISL_429490, EPI_ISL_429491, EPI_ISL_429492, EPI_ISL_429493, EPI_ISL_429494, EPI_ISL_429495, EPI_ISL_429496, EPI_ISL_429497, EPI_ISL_429498, EPI_ISL_429499, EPI_ISL_429500, EPI_ISL_429501, EPI_ISL_429502, EPI_ISL_429503, EPI_ISL_429504, EPI_ISL_429505, EPI_ISL_429506, EPI_ISL_429507, EPI_ISL_429508, EPI_ISL_429509, EPI_ISL_429510, EPI_ISL_429511, EPI_ISL_429512, EPI_ISL_429513, EPI_ISL_429514, EPI_ISL_429515, EPI_ISL_429516, EPI_ISL_429517, EPI_ISL_429518, EPI_ISL_429519, EPI_ISL_429520, EPI_ISL_429521, EPI_ISL_429522, EPI_ISL_429523, EPI_ISL_429524, EPI_ISL_429525, EPI_ISL_429526, EPI_ISL_429527, EPI_ISL_429528, EPI_ISL_429529, EPI_ISL_429530, EPI_ISL_429531, EPI_ISL_429532, EPI_ISL_429533, EPI_ISL_429534, EPI_ISL_429535, EPI_ISL_429536, EPI_ISL_429537, EPI_ISL_429538, EPI_ISL_429539, EPI_ISL_429540, EPI_ISL_429541, EPI_ISL_429542, EPI_ISL_429543, EPI_ISL_429544, EPI_ISL_429545, EPI_ISL_429546, EPI_ISL_429547, EPI_ISL_429548, EPI_ISL_429549, EPI_ISL_429550, EPI_ISL_429551, EPI_ISL_429552, EPI_ISL_429553, EPI_ISL_429554, EPI_ISL_429555, EPI_ISL_429556, EPI_ISL_429557, EPI_ISL_429558, EPI_ISL_429559, EPI_ISL_429560, EPI_ISL_429561, EPI_ISL_429562, EPI_ISL_429563, EPI_ISL_429564, EPI_ISL_429565, EPI_ISL_429566, EPI_ISL_429567, EPI_ISL_429568, EPI_ISL_429569, EPI_ISL_429570, EPI_ISL_429571, EPI_ISL_429572, EPI_ISL_429573, EPI_ISL_429574, EPI_ISL_429575, EPI_ISL_429576, EPI_ISL_429577, EPI_ISL_429578, EPI_ISL_429579, EPI_ISL_429580, EPI_ISL_429581, EPI_ISL_429582, EPI_ISL_429583, EPI_ISL_429584, EPI_ISL_429585, EPI_ISL_429586, EPI_ISL_429587, EPI_ISL_429588, EPI_ISL_429589, EPI_ISL_429590, EPI_ISL_436962, EPI_ISL_436963, EPI_ISL_436964, EPI_ISL_436965, EPI_ISL_436966, EPI_ISL_436967, EPI_ISL_436968, EPI_ISL_436969, EPI_ISL_436970, EPI_ISL_436971, EPI_ISL_436972, EPI_ISL_436973, EPI_ISL_436974, EPI_ISL_436975, EPI_ISL_436976, EPI_ISL_436977, EPI_ISL_436978, EPI_ISL_436979, EPI_ISL_436980, EPI_ISL_436981, EPI_ISL_436982, EPI_ISL_436983, EPI_ISL_436984, EPI_ISL_436985, EPI_ISL_436986, EPI_ISL_436987, EPI_ISL_436988, EPI_ISL_436989, EPI_ISL_436990, EPI_ISL_436991, EPI_ISL_436992, EPI_ISL_436993, EPI_ISL_436994, EPI_ISL_436995, EPI_ISL_436996, EPI_ISL_436997, EPI_ISL_436998, EPI_ISL_436999, EPI_ISL_437000, EPI_ISL_437001, EPI_ISL_437002, EPI_ISL_437003, EPI_ISL_437004, EPI_ISL_437005, EPI_ISL_437006, EPI_ISL_437007, EPI_ISL_437008, EPI_ISL_437009, EPI_ISL_437010, EPI_ISL_437011, EPI_ISL_437012, EPI_ISL_437013, EPI_ISL_437014, EPI_ISL_437015, EPI_ISL_437016, EPI_ISL_437017, EPI_ISL_437018, EPI_ISL_437019, EPI_ISL_437020, EPI_ISL_437021, EPI_ISL_437022, EPI_ISL_437023, EPI_ISL_437024, EPI_ISL_437025, EPI_ISL_437026, EPI_ISL_437027, EPI_ISL_437028, EPI_ISL_437029, EPI_ISL_437030, EPI_ISL_437031, EPI_ISL_437032, EPI_ISL_437033, EPI_ISL_437034, EPI_ISL_437035, EPI_ISL_437036, EPI_ISL_437037, EPI_ISL_437038, EPI_ISL_437039, EPI_ISL_437040, EPI_ISL_437041, EPI_ISL_437042, EPI_ISL_437627, EPI_ISL_437628, EPI_ISL_437629, EPI_ISL_437630, EPI_ISL_437631, EPI_ISL_437632, EPI_ISL_437633, EPI_ISL_437634, EPI_ISL_437635, EPI_ISL_437636, EPI_ISL_437637, EPI_ISL_437638, EPI_ISL_437639, EPI_ISL_437640, EPI_ISL_437641, EPI_ISL_437642, EPI_ISL_437643, EPI_ISL_437644, EPI_ISL_437645, EPI_ISL_437646, EPI_ISL_437647, EPI_ISL_437648, EPI_ISL_437649, EPI_ISL_437650, EPI_ISL_437651, EPI_ISL_437652, EPI_ISL_437653, EPI_ISL_437654, EPI_ISL_437655, EPI_ISL_437656, EPI_ISL_437657, EPI_ISL_437658, EPI_ISL_437659, EPI_ISL_437660, EPI_ISL_437661, EPI_ISL_437662, EPI_ISL_437663, EPI_ISL_437664, EPI_ISL_437665, EPI_ISL_437666, EPI_ISL_437667, EPI_ISL_437668, EPI_ISL_437669, EPI_ISL_437670, EPI_ISL_437671, EPI_ISL_437672, EPI_ISL_437673, EPI_ISL_437674, EPI_ISL_437675, EPI_ISL_437676, EPI_ISL_437677, EPI_ISL_437678, EPI_ISL_437679, EPI_ISL_437680, EPI_ISL_437681, EPI_ISL_437682, EPI_ISL_437683, EPI_ISL_444817, EPI_ISL_444818, EPI_ISL_444819, EPI_ISL_444820, EPI_ISL_444821, EPI_ISL_444822, EPI_ISL_444823, EPI_ISL_444824, EPI_ISL_444825, EPI_ISL_444826, EPI_ISL_444827, EPI_ISL_444828, EPI_ISL_444829, EPI_ISL_444830, EPI_ISL_444831, EPI_ISL_444832, EPI_ISL_444833, EPI_ISL_444834, EPI_ISL_444835, EPI_ISL_444836, EPI_ISL_444837, EPI_ISL_444838, EPI_ISL_444839, EPI_ISL_444840, EPI_ISL_444841, EPI_ISL_444842, EPI_ISL_444843, EPI_ISL_444844, EPI_ISL_444845, EPI_ISL_444846, EPI_ISL_444847, EPI_ISL_444848, EPI_ISL_444849, EPI_ISL_444850, EPI_ISL_444851, EPI_ISL_444852, EPI_ISL_444853, EPI_ISL_444854, EPI_ISL_444855, EPI_ISL_444856, EPI_ISL_444857, EPI_ISL_444858, EPI_ISL_444859, EPI_ISL_444860, EPI_ISL_444861, EPI_ISL_444862, EPI_ISL_444863, EPI_ISL_444864, EPI_ISL_444865, EPI_ISL_444866, EPI_ISL_444867, EPI_ISL_444868, EPI_ISL_444869, EPI_ISL_444870, EPI_ISL_444871, EPI_ISL_444872, EPI_ISL_444873, EPI_ISL_444874, EPI_ISL_444875, EPI_ISL_444876, EPI_ISL_444877, EPI_ISL_444878, EPI_ISL_444879, EPI_ISL_444880, EPI_ISL_444881, EPI_ISL_444882, EPI_ISL_444883, EPI_ISL_444884, EPI_ISL_444885, EPI_ISL_444886, EPI_ISL_444887, EPI_ISL_444888, EPI_ISL_444889, EPI_ISL_444890, EPI_ISL_444891, EPI_ISL_444892, EPI_ISL_444893, EPI_ISL_444894, EPI_ISL_444895, EPI_ISL_444896, EPI_ISL_444897, EPI_ISL_444898, EPI_ISL_444899, EPI_ISL_444900, EPI_ISL_444901, EPI_ISL_444902, EPI_ISL_444903, EPI_ISL_444904, EPI_ISL_444905, EPI_ISL_444906, EPI_ISL_444907, EPI_ISL_444908, EPI_ISL_444909, EPI_ISL_444910, EPI_ISL_444911, EPI_ISL_444912, EPI_ISL_444913, EPI_ISL_444914, EPI_ISL_444915, EPI_ISL_444916, EPI_ISL_444917, EPI_ISL_444918, EPI_ISL_444919, EPI_ISL_444920, EPI_ISL_444921, EPI_ISL_444922, EPI_ISL_444923, EPI_ISL_444924, EPI_ISL_444925, EPI_ISL_444926, EPI_ISL_444927, EPI_ISL_444928, EPI_ISL_444929, EPI_ISL_444930, EPI_ISL_444931, EPI_ISL_444932, EPI_ISL_444933, EPI_ISL_444934, EPI_ISL_444935, EPI_ISL_444936, EPI_ISL_444937, EPI_ISL_444938, EPI_ISL_444939, EPI_ISL_444940, EPI_ISL_444941, EPI_ISL_444942, EPI_ISL_444943, EPI_ISL_444944, EPI_ISL_444945, EPI_ISL_444946, EPI_ISL_444947, EPI_ISL_444948, EPI_ISL_444949, EPI_ISL_444950, EPI_ISL_444951, EPI_ISL_444952, EPI_ISL_444953, EPI_ISL_444954, EPI_ISL_444955, EPI_ISL_444956, EPI_ISL_444957, EPI_ISL_444958, EPI_ISL_444959, EPI_ISL_444960, EPI_ISL_444961, EPI_ISL_444962, EPI_ISL_444963, EPI_ISL_444964, EPI_ISL_444965, EPI_ISL_444966, EPI_ISL_444967, EPI_ISL_444968			
see above	Department of Virus and Microbiological Special Diagnostics, Statens Serum Institut, Copenhagen, Denmark, Artillerivej 5, 2300 Copenahgen S	Albertsen lab, Department of Chemistry and Bioscience, Aalborg University, Denmark	Rasmus Kirkegaard
EPI_ISL_451988, EPI_ISL_451989, EPI_ISL_451990, EPI_ISL_451991, EPI_ISL_451992, EPI_ISL_451993, EPI_ISL_451994, EPI_ISL_451995, EPI_ISL_451996, EPI_ISL_451997, EPI_ISL_451998, EPI_ISL_451999, EPI_ISL_452000, EPI_ISL_452001, EPI_ISL_452002, EPI_ISL_452003, EPI_ISL_452004, EPI_ISL_452005, EPI_ISL_452006, EPI_ISL_452007, EPI_ISL_452008, EPI_ISL_452009, EPI_ISL_452010, EPI_ISL_452011, EPI_ISL_452012, EPI_ISL_452013, EPI_ISL_452014, EPI_ISL_452015, EPI_ISL_452016, EPI_ISL_452017, EPI_ISL_452018, EPI_ISL_452019, EPI_ISL_452020, EPI_ISL_452021, EPI_ISL_452022, EPI_ISL_452023, EPI_ISL_452024, EPI_ISL_452025, EPI_ISL_452026, EPI_ISL_452027, EPI_ISL_452028, EPI_ISL_452029, EPI_ISL_452030, EPI_ISL_452031, EPI_ISL_452032, EPI_ISL_452033, EPI_ISL_452034, EPI_ISL_452035, EPI_ISL_452036, EPI_ISL_452037, EPI_ISL_452038, EPI_ISL_452039, EPI_ISL_452040, EPI_ISL_452041, EPI_ISL_452042, EPI_ISL_452043, EPI_ISL_452044, EPI_ISL_452045, EPI_ISL_452046, EPI_ISL_452047, EPI_ISL_452048, EPI_ISL_452049, EPI_ISL_452050, EPI_ISL_452051, EPI_ISL_452052, EPI_ISL_452053, EPI_ISL_452054, EPI_ISL_452055, EPI_ISL_452056, EPI_ISL_452057, EPI_ISL_452058, EPI_ISL_452059, EPI_ISL_452060, EPI_ISL_452061, EPI_ISL_452062, EPI_ISL_452063, EPI_ISL_452064, EPI_ISL_452065, EPI_ISL_452066, EPI_ISL_452067, EPI_ISL_452068, EPI_ISL_452069, EPI_ISL_452070, EPI_ISL_452071, EPI_ISL_452072, EPI_ISL_452073, EPI_ISL_452074, EPI_ISL_452075, EPI_ISL_452076, EPI_ISL_452077, EPI_ISL_452078, EPI_ISL_452079, EPI_ISL_452080, EPI_ISL_452081, EPI_ISL_452082, EPI_ISL_452083, EPI_ISL_452084, EPI_ISL_452085, EPI_ISL_452086, EPI_ISL_452087, EPI_ISL_452088, EPI_ISL_452089, EPI_ISL_452090, EPI_ISL_452091, EPI_ISL_452092, EPI_ISL_452093, EPI_ISL_452094, EPI_ISL_452095, EPI_ISL_452096, EPI_ISL_452097, EPI_ISL_452098, EPI_ISL_452099, EPI_ISL_452100			
see above	Department of Clinical Microbiology, Copenhagen University Hospital, Hvidovre, Kettegaard Alle 30, 2650 Hvidovre.	Albertsen lab, Department of Chemistry and Bioscience, Aalborg University, Denmark	Rasmus Kirkegaard
EPI_ISL_452101, EPI_ISL_452102	Department of Virus and Microbiological Special Diagnostics, Statens Serum Institut, Copenhagen, Denmark, Artillerivej 5, 2300 Copenahgen S	Albertsen lab, Department of Chemistry and Bioscience, Aalborg University, Denmark	Rasmus Kirkegaard
EPI_ISL_515974	Department of Virus and Microbiological Special Diagnostics, Statens Serum Institut	Albertsen lab, Department of Chemistry and Bioscience, Aalborg University, Denmark	Danish Corona Danica Consortia

We gratefully acknowledge the following Authors from the Originating laboratories responsible for obtaining the specimens, as well as the Submitting laboratories where the genome data were generated and shared via GISAID, on which this research is based.

All Submitters of data may be contacted directly via www.gisaid.org

Authors are sorted alphabetically.

Accession ID	Originating Laboratory	Submitting Laboratory	Authors
EPI_ISL_1025908, EPI_ISL_1025909, EPI_ISL_1025910, EPI_ISL_1025911, EPI_ISL_1025912, EPI_ISL_1025913, EPI_ISL_1025914, EPI_ISL_1025915, EPI_ISL_1025916, EPI_ISL_1025917, EPI_ISL_1025918, EPI_ISL_1025919, EPI_ISL_1025920, EPI_ISL_1025921, EPI_ISL_1025922, EPI_ISL_1025923, EPI_ISL_1025924, EPI_ISL_1025925, EPI_ISL_1025926, EPI_ISL_1025927, EPI_ISL_1025928, EPI_ISL_1025929, EPI_ISL_1025930, EPI_ISL_1025931, EPI_ISL_1025932, EPI_ISL_1025933, EPI_ISL_1025934, EPI_ISL_1025935, EPI_ISL_1025936, EPI_ISL_1025937, EPI_ISL_1025938, EPI_ISL_1025939, EPI_ISL_1025940, EPI_ISL_1025941, EPI_ISL_1025942, EPI_ISL_1025943, EPI_ISL_1025944, EPI_ISL_1025945, EPI_ISL_1025946, EPI_ISL_1025947, EPI_ISL_1025948, EPI_ISL_1025949, EPI_ISL_1025950, EPI_ISL_1025951, EPI_ISL_1025952, EPI_ISL_1025953, EPI_ISL_1025954, EPI_ISL_1025955, EPI_ISL_1025956, EPI_ISL_1025957, EPI_ISL_1025958, EPI_ISL_1025959, EPI_ISL_1025960, EPI_ISL_1025961, EPI_ISL_1025962, EPI_ISL_1065096, EPI_ISL_1065097, EPI_ISL_1065099, EPI_ISL_1065103, EPI_ISL_1065189, EPI_ISL_1065275, EPI_ISL_1065294, EPI_ISL_1065312, EPI_ISL_1065370, EPI_ISL_1065437, EPI_ISL_1065587, EPI_ISL_1065603, EPI_ISL_1065614, EPI_ISL_1065662, EPI_ISL_1065709, EPI_ISL_1065716, EPI_ISL_1065721, EPI_ISL_1065764, EPI_ISL_1065786, EPI_ISL_1065801, EPI_ISL_1065886, EPI_ISL_1065939, EPI_ISL_1066047, EPI_ISL_1066147, EPI_ISL_1066162, EPI_ISL_1066181, EPI_ISL_1066231, EPI_ISL_1066241, EPI_ISL_1066255, EPI_ISL_1066265, EPI_ISL_1066295, EPI_ISL_1066303, EPI_ISL_1066413, EPI_ISL_1066427, EPI_ISL_1066450, EPI_ISL_1066454, EPI_ISL_1066504, EPI_ISL_1066541, EPI_ISL_1066560, EPI_ISL_1066575, EPI_ISL_1066577, EPI_ISL_1066678, EPI_ISL_1066715, EPI_ISL_1066717, EPI_ISL_1066836, EPI_ISL_1066879, EPI_ISL_1066880, EPI_ISL_1066881, EPI_ISL_1066882, EPI_ISL_1066933, EPI_ISL_1066977, EPI_ISL_1067026, EPI_ISL_1067083, EPI_ISL_1067087, EPI_ISL_1067111, EPI_ISL_1067180, EPI_ISL_1067182, EPI_ISL_1067191, EPI_ISL_1067206, EPI_ISL_1067215, EPI_ISL_1067222, EPI_ISL_1067288, EPI_ISL_1067293, EPI_ISL_1067316, EPI_ISL_1067320, EPI_ISL_1067364, EPI_ISL_1067402, EPI_ISL_1067409, EPI_ISL_1067414, EPI_ISL_1067433, EPI_ISL_1067448, EPI_ISL_1067487, EPI_ISL_1067489, EPI_ISL_1067504, EPI_ISL_1067518, EPI_ISL_1067524, EPI_ISL_1067535, EPI_ISL_1067571			
see above	Department of Virus and Microbiological Special Diagnostics, Statens Serum Institut, Copenhagen, Denmark	Aalborg University	Danish Covid-19 Genome Consortium
EPI_ISL_625695, EPI_ISL_625698, EPI_ISL_625706, EPI_ISL_625709, EPI_ISL_625742, EPI_ISL_625822, EPI_ISL_625823, EPI_ISL_625864, EPI_ISL_625883, EPI_ISL_625890, EPI_ISL_626124, EPI_ISL_626127, EPI_ISL_626129, EPI_ISL_626132, EPI_ISL_626133, EPI_ISL_626134, EPI_ISL_626137, EPI_ISL_626138, EPI_ISL_626139, EPI_ISL_626144, EPI_ISL_626146, EPI_ISL_626148, EPI_ISL_626149, EPI_ISL_626157, EPI_ISL_626162, EPI_ISL_626164			
see above	Department of Virus and Microbiological Special Diagnostics, Statens Serum Institut, Denmark	Albertsen lab, Department of Chemistry and Bioscience, Aalborg University, Denmark	Danish Covid-19 Genome Consortia
EPI_ISL_641514, EPI_ISL_641515, EPI_ISL_641516	Department of Virus and Microbiological Special Diagnostics, Statens Serum Institut, Copenhagen, Denmark	Albertsen lab, Department of Chemistry and Bioscience, Aalborg University, Denmark	Thomas Bruun Rasmussen, Jannik Fonager, Morten Rasmussen
EPI_ISL_668458, EPI_ISL_668459, EPI_ISL_668460, EPI_ISL_668461, EPI_ISL_668462, EPI_ISL_668463, EPI_ISL_668464, EPI_ISL_668465, EPI_ISL_668466, EPI_ISL_668467, EPI_ISL_668468, EPI_ISL_668469, EPI_ISL_668470, EPI_ISL_668471, EPI_ISL_668472, EPI_ISL_668473, EPI_ISL_668474, EPI_ISL_668475, EPI_ISL_668476, EPI_ISL_668477, EPI_ISL_668478, EPI_ISL_668479, EPI_ISL_668480, EPI_ISL_668481, EPI_ISL_668482, EPI_ISL_668483, EPI_ISL_668484, EPI_ISL_668485, EPI_ISL_668486, EPI_ISL_668487, EPI_ISL_668488, EPI_ISL_668489, EPI_ISL_668490, EPI_ISL_668491, EPI_ISL_668492, EPI_ISL_668493, EPI_ISL_668494, EPI_ISL_668495, EPI_ISL_668496, EPI_ISL_668497, EPI_ISL_668498, EPI_ISL_668499, EPI_ISL_668500, EPI_ISL_668501, EPI_ISL_668502, EPI_ISL_668503, EPI_ISL_668504, EPI_ISL_668505, EPI_ISL_668506, EPI_ISL_668507, EPI_ISL_668508, EPI_ISL_668509, EPI_ISL_668510, EPI_ISL_668511, EPI_ISL_668512, EPI_ISL_668513, EPI_ISL_668514, EPI_ISL_668515, EPI_ISL_668516, EPI_ISL_668517, EPI_ISL_668518, EPI_ISL_668519, EPI_ISL_668520, EPI_ISL_668521, EPI_ISL_668522, EPI_ISL_668523, EPI_ISL_668524, EPI_ISL_668525, EPI_ISL_668526, EPI_ISL_668527, EPI_ISL_668528, EPI_ISL_668529, EPI_ISL_668530, EPI_ISL_668531, EPI_ISL_668532, EPI_ISL_668533, EPI_ISL_668534, EPI_ISL_668535, EPI_ISL_668536, EPI_ISL_668537, EPI_ISL_668538, EPI_ISL_668539, EPI_ISL_668540, EPI_ISL_668541, EPI_ISL_668542, EPI_ISL_668543, EPI_ISL_668544, EPI_ISL_668545, EPI_ISL_668546, EPI_ISL_668547, EPI_ISL_668548, EPI_ISL_668549, EPI_ISL_668550, EPI_ISL_668551, EPI_ISL_668552, EPI_ISL_668553, EPI_ISL_668554, EPI_ISL_668555, EPI_ISL_668556, EPI_ISL_668557, EPI_ISL_668558, EPI_ISL_668559, EPI_ISL_668560, EPI_ISL_668561, EPI_ISL_668562, EPI_ISL_668563, EPI_ISL_668564, EPI_ISL_668565, EPI_ISL_668566, EPI_ISL_668567, EPI_ISL_668568, EPI_ISL_668569, EPI_ISL_668570, EPI_ISL_668571, EPI_ISL_668572, EPI_ISL_668573, EPI_ISL_668574, EPI_ISL_668575, EPI_ISL_668576, EPI_ISL_668577, EPI_ISL_668578, EPI_ISL_668579, EPI_ISL_668580, EPI_ISL_668581, EPI_ISL_668582, EPI_ISL_668583, EPI_ISL_668584, EPI_ISL_668585, EPI_ISL_668586, EPI_ISL_668587, EPI_ISL_668588, EPI_ISL_668589, EPI_ISL_668590, EPI_ISL_668591, EPI_ISL_668592, EPI_ISL_668593, EPI_ISL_668594, EPI_ISL_668595, EPI_ISL_668596, EPI_ISL_668597, EPI_ISL_668598, EPI_ISL_668599, EPI_ISL_668600, EPI_ISL_668601, EPI_ISL_668602, EPI_ISL_668603, EPI_ISL_668604, EPI_ISL_668605, EPI_ISL_668606, EPI_ISL_668607, EPI_ISL_668608, EPI_ISL_668609, EPI_ISL_668610, EPI_ISL_668611, EPI_ISL_668612, EPI_ISL_668613, EPI_ISL_668614, EPI_ISL_668615, EPI_ISL_668616, EPI_ISL_668617, EPI_ISL_668618, EPI_ISL_668619, EPI_ISL_668620, EPI_ISL_668621, EPI_ISL_668622, EPI_ISL_668623, EPI_ISL_668624, EPI_ISL_668625, EPI_ISL_668626, EPI_ISL_668627, EPI_ISL_668628, EPI_ISL_668629, EPI_ISL_668630, EPI_ISL_668631, EPI_ISL_668632, EPI_ISL_668633, EPI_ISL_668634, EPI_ISL_668635, EPI_ISL_668636, EPI_ISL_668637, EPI_ISL_668638, EPI_ISL_668639, EPI_ISL_668640, EPI_ISL_668641, EPI_ISL_668642, EPI_ISL_668643, EPI_ISL_668644, EPI_ISL_668645, EPI_ISL_668646, EPI_ISL_668647, EPI_ISL_668648, EPI_ISL_668649, EPI_ISL_668650, EPI_ISL_668651, EPI_ISL_668652, EPI_ISL_668653, EPI_ISL_668654, EPI_ISL_668655, EPI_ISL_668656, EPI_ISL_668657, EPI_ISL_668658, EPI_ISL_668659, EPI_ISL_668660, EPI_ISL_668661, EPI_ISL_668662, EPI_ISL_668663, EPI_ISL_668664, EPI_ISL_668665, EPI_ISL_668666, EPI_ISL_668667, EPI_ISL_668668, EPI_ISL_668669, EPI_ISL_668670, EPI_ISL_668671, EPI_ISL_668672, EPI_ISL_668673, EPI_ISL_668674, EPI_ISL_668675, EPI_ISL_668676, EPI_ISL_668677, EPI_ISL_668678, EPI_ISL_668679, EPI_ISL_668680, EPI_ISL_668681, EPI_ISL_668682, EPI_ISL_668683, EPI_ISL_668684, EPI_ISL_668685, EPI_ISL_668686, EPI_ISL_668687, EPI_ISL_668688, EPI_ISL_668689, EPI_ISL_668690, EPI_ISL_668691, EPI_ISL_668692, EPI_ISL_668693, EPI_ISL_668694, EPI_ISL_668695, EPI_ISL_668696, EPI_ISL_668697, EPI_ISL_668698, EPI_ISL_668699, EPI_ISL_668700, EPI_ISL_668701, EPI_ISL_668702, EPI_ISL_668703, EPI_ISL_668704, EPI_ISL_668705, EPI_ISL_668706, EPI_ISL_668707, EPI_ISL_668708, EPI_ISL_668709, EPI_ISL_668710, EPI_ISL_668711, EPI_ISL_668712, EPI_ISL_668713, EPI_ISL_668714, EPI_ISL_668715, EPI_ISL_668716, EPI_ISL_668717, EPI_ISL_668718, EPI_ISL_668719, EPI_ISL_668720, EPI_ISL_668721, EPI_ISL_668722, EPI_ISL_668723, EPI_ISL_668724, EPI_ISL_668725, EPI_ISL_668726, EPI_ISL_668727, EPI_ISL_668728, EPI_ISL_668729, EPI_ISL_668730, EPI_ISL_668731, EPI_ISL_668732, EPI_ISL_668733, EPI_ISL_668734, EPI_ISL_668735, EPI_ISL_668736, EPI_ISL_668737, EPI_ISL_668738, EPI_ISL_668739, EPI_ISL_668740, EPI_ISL_668741, EPI_ISL_668742, EPI_ISL_668743, EPI_ISL_668744, EPI_ISL_668745, EPI_ISL_668746, EPI_ISL_668747, EPI_ISL_668748, EPI_ISL_668749, EPI_ISL_668750, EPI_ISL_668751, EPI_ISL_668752, EPI_ISL_668753, EPI_ISL_668754, EPI_ISL_668755, EPI_ISL_668756, EPI_ISL_668757, EPI_ISL_668758, EPI_ISL_668759, EPI_ISL_668760, EPI_ISL_668761, EPI_ISL_668762, EPI_ISL_668763, EPI_ISL_668764, EPI_ISL_668765, EPI_ISL_668766, EPI_ISL_668767, EPI_ISL_668768, EPI_ISL_668769, EPI_ISL_668770, EPI_ISL_668771, EPI_ISL_668772, EPI_ISL_668773, EPI_ISL_668774, EPI_ISL_668775, EPI_ISL_668776, EPI_ISL_668777, EPI_ISL_668778, EPI_ISL_668779, EPI_ISL_668780, EPI_ISL_668781, EPI_ISL_668782, EPI_ISL_668783, EPI_ISL_668784, EPI_ISL_668785, EPI_ISL_668786, EPI_ISL_668787, EPI_ISL_668788, EPI_ISL_668789, EPI_ISL_668790, EPI_ISL_668791, EPI_ISL_668792, EPI_ISL_668793, EPI_ISL_668794, EPI_ISL_668795, EPI_ISL_668796, EPI_ISL_668797, EPI_ISL_668798, EPI_ISL_668799, EPI_ISL_668800, EPI_ISL_668801, EPI_ISL_668802, EPI_ISL_668803, EPI_ISL_668804, EPI_ISL_668805, EPI_ISL_668806, EPI_ISL_668807, EPI_ISL_668808, EPI_ISL_668809, EPI_ISL_668810, EPI_ISL_668811, EPI_ISL_668812, EPI_ISL_668813, EPI_ISL_668814, EPI_ISL_668815, EPI_ISL_668816, EPI_ISL_668817, EPI_ISL_668818, EPI_ISL_668819, EPI_ISL_668820, EPI_ISL_668821, EPI_ISL_668822, EPI_ISL_668823, EPI_ISL_668824, EPI_ISL_668825, EPI_ISL_668826, EPI_ISL_668827, EPI_ISL_668828, EPI_ISL_668829, EPI_ISL_668830, EPI_ISL_668831, EPI_ISL_668832, EPI_ISL_668833, EPI_ISL_668834, EPI_ISL_668835, EPI_ISL_668836, EPI_ISL_668837, EPI_ISL_668838, EPI_ISL_668839, EPI_ISL_668840, EPI_ISL_668841, EPI_ISL_668842, EPI_ISL_668843, EPI_ISL_668844, EPI_ISL_668845, EPI_ISL_668846, EPI_ISL_668847, EPI_ISL_668848, EPI_ISL_668849, EPI_ISL_668850, EPI_ISL_668851, EPI_ISL_668852, EPI_ISL_668853, EPI_ISL_668854, EPI_ISL_668855, EPI_ISL_668856, EPI_ISL_668857, EPI_ISL_668858, EPI_ISL_668859, EPI_ISL_668860, EPI_ISL_668861, EPI_ISL_668862, EPI_ISL_668863, EPI_ISL_668864, EPI_ISL_668865, EPI_ISL_668866, EPI_ISL_668867, EPI_ISL_668868, EPI_ISL_668869, EPI_ISL_668870, EPI_ISL_668871, EPI_ISL_668872, EPI_ISL_668873, EPI_ISL_668874, EPI_ISL_668875, EPI_ISL_668876, EPI_ISL_668877, EPI_ISL_668878, EPI_ISL_668879, EPI_ISL_668880, EPI_ISL_668881, EPI_ISL_668882, EPI_ISL_668883, EPI_ISL_668884, EPI_ISL_668885, EPI_ISL_668886, EPI_ISL_668887, EPI_ISL_668888, EPI_ISL_668889, EPI_ISL_668890, EPI_ISL_668891, EPI_ISL_668892, EPI_ISL_668893, EPI_ISL_668894, EPI_ISL_668895, EPI_ISL_668896, EPI_ISL_668897, EPI_ISL_668898, EPI_ISL_668899, EPI_ISL_668900, EPI_ISL_668901, EPI_ISL_668902, EPI_ISL_668903, EPI_ISL_668904, EPI_ISL_668905, EPI_ISL_668906, EPI_ISL_668907, EPI_ISL_668908, EPI_ISL_668909, EPI_ISL_668910, EPI_ISL_668911, EPI_ISL_668912, EPI_ISL_668913, EPI_ISL_668914, EPI_ISL_668915, EPI_ISL_668916, EPI_ISL_668917, EPI_ISL_668918, EPI_ISL_668919, EPI_ISL_668920, EPI_ISL_668921, EPI_ISL_668922, EPI_ISL_668923, EPI_ISL_668924, EPI_ISL_668925, EPI_ISL_668926, EPI_ISL_668927, EPI_ISL_668928, EPI_ISL_668929, EPI_ISL_668930, EPI_ISL_668931, EPI_ISL_668932, EPI_ISL_668933, EPI_ISL_668934, EPI_ISL_668935, EPI_ISL_668936, EPI_ISL_668937, EPI_ISL_668938, EPI_ISL_668939, EPI_ISL_668940, EPI_ISL_668941, EPI_ISL_668942, EPI_ISL_668943, EPI_ISL_668944, EPI_ISL_668945, EPI_ISL_668946, EPI_ISL_668947, EPI_ISL_668948, EPI_ISL_668949, EPI_ISL_668950, EPI_ISL_668951, EPI_ISL_668952, EPI_ISL_668953, EPI_ISL_668954, EPI_ISL_668955, EPI_ISL_668956, EPI_ISL_668957, EPI_ISL_668958, EPI_ISL_668959, EPI_ISL_668960, EPI_ISL_668961, EPI_ISL_668962, EPI_ISL_668963, EPI_ISL_668964, EPI_ISL_668965, EPI_ISL_668966, EPI_ISL_668967, EPI_ISL_668968, EPI_ISL_668969, EPI_ISL_668970, EPI_ISL_668971, EPI_ISL_668972, EPI_ISL_668973, EPI_ISL_668974, EPI_ISL_668975, EPI_ISL_668976, EPI_ISL_668977, EPI_ISL_668978, EPI_ISL_668979, EPI_ISL_668980, EPI_ISL_668981, EPI_ISL_668982, EPI_ISL_668983, EPI_ISL_668984, EPI_ISL_668985, EPI_ISL_668986, EPI_ISL_668987, EPI_ISL_668988, EPI_ISL_668989, EPI_ISL_668990, EPI_ISL_668991, EPI_ISL_668992, EPI_ISL_668993, EPI_ISL_668994, EPI_ISL_668995, EPI_ISL_668996, EPI_ISL_668997, EPI_ISL_668998, EPI_ISL_668999, EPI_ISL_669000, EPI_ISL_669001, EPI_ISL_669002, EPI_ISL_669003, EPI_ISL_669004, EPI_ISL_669005, EPI_ISL_669006, EPI_ISL_669007, EPI_ISL_669008, EPI_ISL_669009, EPI_ISL_669010, EPI_ISL_669011, EPI_ISL_669012, EPI_ISL_669013, EPI_ISL_669014, EPI_ISL_669015, EPI_ISL_669016, EPI_ISL_669017, EPI_ISL_669018, EPI_ISL_669019, EPI_ISL_669020, EPI_ISL_669021, EPI_ISL_669022, EPI_ISL_669023, EPI_ISL_669024, EPI_ISL_669025, EPI_ISL_669026, EPI_ISL_669027, EPI_ISL_669028, EPI_ISL_669029, EPI_ISL_669030, EPI_ISL_669031, EPI_ISL_669032, EPI_ISL_669033, EPI_ISL_669034, EPI_ISL_669035, EPI_ISL_669036, EPI_ISL_669037, EPI_ISL_669038, EPI_ISL_669039, EPI_ISL_669040, EPI_ISL_669041, EPI_ISL_669042, EPI_ISL_669043, EPI_ISL_669044, EPI_ISL_669045, EPI_ISL_669046, EPI_ISL_669047, EPI_ISL_669048, EPI_ISL_669049, EPI_ISL_669050, EPI_ISL_669051, EPI_ISL_669052, EPI_ISL_669053, EPI_ISL_669054, EPI_ISL_669055, EPI_ISL_669056, EPI_ISL_669057, EPI_ISL_669058, EPI_ISL_669059, EPI_ISL_669060, EPI_ISL_669061, EPI_ISL_669062, EPI_ISL_669063, EPI_ISL_669064, EPI_ISL_669065, EPI_ISL_669066, EPI_ISL_669067, EPI_ISL_669068, EPI_ISL_669069, EPI_ISL_669070, EPI_ISL_669071, EPI_ISL_669072, EPI_ISL_669073, EPI_ISL_669074, EPI_ISL_669075, EPI_ISL_669076, EPI_ISL_669077, EPI_ISL_669078, EPI_ISL_669079, EPI_ISL_669080, EPI_ISL_669081, EPI_ISL_669082, EPI_ISL_669083, EPI_ISL_669084, EPI_ISL_669085, EPI_ISL_669086, EPI_ISL_669087, EPI_ISL_669088, EPI_ISL_669089, EPI_ISL_669090, EPI_ISL_669091, EPI_ISL_669092, EPI_ISL_669093, EPI_ISL_669094, EPI_ISL_669095, EPI_ISL_669096, EPI_ISL_669097, EPI_ISL_669098, EPI_ISL_669099, EPI_ISL_669100, EPI_ISL_669101, EPI_ISL_669102, EPI_ISL_669103, EPI_ISL_669104, EPI_ISL_669105, EPI_ISL_669106, EPI_ISL_669107, EPI_ISL_669108, EPI_ISL_669109, EPI_ISL_669110, EPI_ISL_669111, EPI_ISL_669112, EPI_ISL_669113, EPI_ISL_669114, EPI_ISL_669115, EPI_ISL_669116, EPI_ISL_669117, EPI_ISL_669118, EPI_ISL_669119, EPI_ISL_669120, EPI_ISL_669121, EPI_ISL_669122, EPI_ISL_669123, EPI_ISL_669124, EPI_ISL_669125, EPI_ISL_669126, EPI_ISL_669127, EPI_ISL_669128, EPI_ISL_669129, EPI_ISL_669130, EPI_ISL_669131, EPI_ISL_669132, EPI_ISL_669133, EPI_ISL_669134, EPI_ISL_669135, EPI_ISL_669136, EPI_ISL_669137, EPI_ISL_669138, EPI_ISL_669139, EPI_ISL_669140, EPI_ISL_669141, EPI_ISL_669142, EPI_ISL_669143, EPI_ISL_669144, EPI_ISL_669145, EPI_ISL_669146, EPI_ISL_669147, EPI_ISL_669148, EPI_ISL_669149, EPI_ISL_669150, EPI_ISL_669151, EPI_ISL_669152, EPI_ISL_669153, EPI_ISL_669154, EPI_ISL_669155, EPI_ISL_669156, EPI_ISL_669157, EPI_ISL_669158, EPI_ISL_669159, EPI_ISL_669160, EPI_ISL_669161, EPI_ISL_669162, EPI_ISL_669163, EPI_ISL_669164, EPI_ISL_669165, EPI_ISL_669166, EPI_ISL_669167, EPI_ISL_669168, EPI_ISL_669169, EPI_ISL_669170, EPI_ISL_669171, EPI_ISL_669172, EPI_ISL_669173, EPI_ISL_669174, EPI_ISL_669175, EPI_ISL_669176, EPI_ISL_669177, EPI_ISL_669178, EPI_ISL_669179, EPI_ISL_669180, EPI_ISL_669181, EPI_ISL_669182, EPI_ISL_669183, EPI_ISL_669184, EPI_ISL_669185, EPI_ISL_669186, EPI_ISL_669187, EPI_ISL_669188, EPI_ISL_669189, EPI_ISL_669190, EPI_ISL_669191, EPI_ISL_669192, EPI_ISL_669193, EPI_ISL_669194, EPI_ISL_669195, EPI_ISL_669196, EPI_ISL_669197, EPI_ISL_669198, EPI_ISL_669199, EPI_ISL_669200, EPI_ISL_669201, EPI_ISL_669202, EPI_ISL_669203, EPI_ISL_669204, EPI_ISL_669205, EPI_ISL_669206, EPI_ISL_669207, EPI_ISL_669208, EPI_ISL_669209, EPI_ISL_669210, EPI_ISL_669211, EPI_ISL_669212, EPI_ISL_669213, EPI_ISL_669214, EPI_ISL_669215, EPI_ISL_669216, EPI_ISL_669217, EPI_ISL_669218, EPI_ISL_669219, EPI_ISL_669220, EPI_ISL_669221, EPI_ISL_669222, EPI_ISL_669223, EPI_ISL_669224, EPI_ISL_669225, EPI_ISL_669226, EPI_ISL_669227, EPI_ISL_669228, EPI_ISL_669229, EPI_ISL_669230, EPI_ISL_669231, EPI_ISL_669232, EPI_ISL_669233, EPI_ISL_669234, EPI_ISL_669235, EPI_ISL_669236, EPI_ISL_669237, EPI_ISL_669238, EPI_ISL_669239, EPI_ISL_669240, EPI_ISL_669241, EPI_ISL_669242, EPI_ISL_669243, EPI_ISL_669244, EPI_ISL_669245, EPI_ISL_669246, EPI_ISL_669247, EPI_ISL_669248, EPI_ISL_669249, EPI_ISL_669250, EPI_ISL_669251, EPI_ISL_669252, EPI_ISL_669253, EPI_ISL_669254, EPI_ISL_669255, EPI_ISL_669256, EPI_ISL_669257, EPI_ISL_669258, EPI_ISL_669259, EPI_ISL_669260, EPI_ISL_669261, EPI_ISL_669262, EPI_ISL_669263, EPI_ISL_669264, EPI_ISL_669265, EPI_ISL_669266, EPI_ISL_669267, EPI_ISL_669268, EPI_ISL_669269, EPI_ISL_669270, EPI_ISL_669271, EPI_ISL_669272, EPI_ISL_669273, EPI_ISL_669274, EPI_ISL_669275, EPI_ISL_669276, EPI_ISL_669277, EPI_ISL_669278, EPI_ISL_669279, EPI_ISL_669280, EPI_ISL_669281, EPI_ISL_669282, EPI_ISL_669283, EPI_ISL_669284, EPI_ISL_669285, EPI_ISL_669286, EPI_ISL_669287, EPI_ISL_669288, EPI_ISL_669289, EPI_ISL_669290, EPI_ISL_669291, EPI_ISL_669292, EPI_ISL_669293, EPI_ISL_669294, EPI_ISL_669295, EPI_ISL_669296, EPI_ISL_669297, EPI_ISL_669298, EPI_ISL_669299, EPI_ISL_669300, EPI_ISL_669301, EPI_ISL_669302, EPI_ISL_669303, EPI_ISL_669304, EPI_ISL_669305, EPI_ISL_669306, EPI_ISL_669307, EPI_ISL_669308, EPI_ISL_669309, EPI_ISL_669310, EPI_ISL_669311, EPI_ISL_669312, EPI_ISL_669313, EPI_ISL_669314, EPI_ISL_669315, EPI_ISL_669316, EPI_ISL_669317, EPI_ISL_669318, EPI_ISL_669319, EPI_ISL_669320, EPI_ISL_669321, EPI_ISL_669322, EPI_ISL_669323, EPI_ISL_669324, EPI_ISL_669325, EPI_ISL_669326, EPI_ISL_669327, EPI_ISL_669328, EPI_ISL_669329, EPI_ISL_669330, EPI_ISL_669331, EPI_ISL_669332, EPI_ISL_669333, EPI_ISL_669334, EPI_ISL_669335, EPI_ISL_669336, EPI_ISL_669337, EPI_ISL_669338, EPI_ISL_669339, EPI_ISL_669340, EPI_ISL_669341, EPI_ISL_669342, EPI_ISL_669343, EPI_ISL_669344, EPI_ISL_669345, EPI_ISL_669346, EPI_ISL_669347, EPI_ISL_669348, EPI_ISL_669349, EPI_ISL_669350, EPI_ISL_669351, EPI_ISL_669352, EPI_ISL_669353, EPI_ISL_669354, EPI_ISL_669355, EPI_ISL_669356, EPI_ISL_669357, EPI_ISL_669358, EPI_ISL_669359, EPI_ISL_669360, EPI_ISL_669361, EPI_ISL_669362, EPI_ISL_669363, EPI_ISL_669364, EPI_ISL_669365, EPI_ISL_669366, EPI_ISL_669367, EPI_ISL_669368, EPI_ISL_669369, EPI_ISL_669370, EPI_ISL_669371, EPI_ISL_669372, EPI_ISL_669373, EPI_ISL_669374, EPI_ISL_669375, EPI_ISL_669376, EPI_ISL_669377, EPI_ISL_669378, EPI_ISL_669379, EPI_ISL_669380, EPI_ISL_669381, EPI_ISL_669382, EPI_ISL_669383, EPI_ISL_669384, EPI_ISL_669385, EPI_ISL_669386, EPI_ISL_669387, EPI_ISL_669388, EPI_ISL_669389, EPI_ISL_669390, EPI_ISL_669391, EPI_ISL_669392, EPI_ISL_669393, EPI_ISL_669394, EPI_ISL_669395, EPI_ISL_669396, EPI_ISL_669397, EPI_ISL_669398, EPI_ISL_669399, EPI_ISL_669400, EPI_ISL_669401, EPI_ISL_669402, EPI_ISL_669403, EPI_ISL_669404, EPI_ISL_669405, EPI_ISL_669406, EPI_ISL_669407, EPI_ISL_669408, EPI_ISL_669409, EPI_ISL_669410, EPI_ISL_669411, EPI_ISL_669412, EPI_ISL_669413, EPI_ISL_669414, EPI_ISL_669415, EPI_ISL_669416, EPI_ISL_669417, EPI_ISL_669418, EPI_ISL_669419, EPI_ISL_669420, EPI_ISL_669421, EPI_ISL_669422, EPI_ISL_669423, EPI_ISL_669424, EPI_ISL_669425, EPI_ISL_669426, EPI_ISL_669427, EPI_ISL_669428, EPI_ISL_669429, EPI_ISL_669430, EPI_ISL_669431, EPI_ISL_669432, EPI_ISL_669433, EPI_ISL_669434, EPI_ISL_669435, EPI_ISL_669436, EPI_ISL_669437, EPI_ISL_669438, EPI_ISL_669439, EPI_ISL_669440, EPI_ISL_669441, EPI_ISL_669442, EPI_ISL_669443, EPI_ISL_669444, EPI_ISL_669445, EPI_ISL_669446, EPI_ISL_669447, EPI_ISL_669448, EPI_ISL_669449, EPI_ISL_669450, EPI_ISL_669451, EPI_ISL_669452, EPI_ISL_669453, EPI_ISL_669454, EPI_ISL_669455, EPI_ISL_669456, EPI_ISL_669457, EPI_ISL_669458, EPI_ISL_669459, EPI_ISL_669460, EPI_ISL_669461, EPI_ISL_669462, EPI_ISL_669463, EPI_ISL_669464, EPI_ISL_669465, EPI_ISL_669466, EPI_ISL_669467, EPI_ISL_669468, EPI_ISL_669469, EPI_ISL_669470, EPI_ISL_669471, EPI_ISL_669472, EPI_ISL_669473, EPI_ISL_669474, EPI_ISL_669475, EPI_ISL_669476, EPI_ISL_669477, EPI_ISL_669478, EPI_ISL_669479, EPI_ISL_669480, EPI_ISL_669481, EPI_ISL_669482, EPI_ISL_669483,			

We gratefully acknowledge the following Authors from the Originating laboratories responsible for obtaining the specimens, as well as the Submitting laboratories where the genome data were generated and shared via GISAID, on which this research is based.

All Submitters of data may be contacted directly via www.gisaid.org

Authors are sorted alphabetically.

Accession ID	Originating Laboratory	Submitting Laboratory	Authors
EPI_ISL_1025670, EPI_ISL_1025671, EPI_ISL_1025672, EPI_ISL_1025673, EPI_ISL_1025674, EPI_ISL_1025675, EPI_ISL_1025676, EPI_ISL_1025677, EPI_ISL_1025678, EPI_ISL_1025679, EPI_ISL_1025680, EPI_ISL_1025681, EPI_ISL_1025682, EPI_ISL_1025683, EPI_ISL_1025684, EPI_ISL_1025685, EPI_ISL_1025686, EPI_ISL_1025687, EPI_ISL_1025688, EPI_ISL_1025689, EPI_ISL_1025690, EPI_ISL_1025691, EPI_ISL_1025692, EPI_ISL_1025693, EPI_ISL_1025694, EPI_ISL_1025695, EPI_ISL_1025696, EPI_ISL_1025697, EPI_ISL_1025698, EPI_ISL_1025699, EPI_ISL_1025700, EPI_ISL_1025701, EPI_ISL_1025702, EPI_ISL_1025703, EPI_ISL_1025704, EPI_ISL_1025705, EPI_ISL_1025706, EPI_ISL_1025707, EPI_ISL_1025708, EPI_ISL_1025709, EPI_ISL_1025710, EPI_ISL_1025711, EPI_ISL_1025712, EPI_ISL_1025713, EPI_ISL_1025714, EPI_ISL_1025715, EPI_ISL_1025716, EPI_ISL_1025717, EPI_ISL_1025718, EPI_ISL_1025719, EPI_ISL_1025720, EPI_ISL_1025721, EPI_ISL_1025722, EPI_ISL_1025723, EPI_ISL_1025724, EPI_ISL_1025725, EPI_ISL_1025726, EPI_ISL_1025727, EPI_ISL_1025728, EPI_ISL_1025729, EPI_ISL_1025730, EPI_ISL_1025731, EPI_ISL_1025732, EPI_ISL_1025733, EPI_ISL_1025734, EPI_ISL_1025735, EPI_ISL_1025736, EPI_ISL_1025737, EPI_ISL_1025738, EPI_ISL_1025739, EPI_ISL_1025740, EPI_ISL_1025741, EPI_ISL_1025742, EPI_ISL_1025743, EPI_ISL_1025744, EPI_ISL_1025745, EPI_ISL_1025746, EPI_ISL_1025747, EPI_ISL_1025748, EPI_ISL_1025749, EPI_ISL_1025750, EPI_ISL_1025751, EPI_ISL_1025752, EPI_ISL_1025753, EPI_ISL_1025754, EPI_ISL_1025755, EPI_ISL_1025756, EPI_ISL_1025757, EPI_ISL_1025758, EPI_ISL_1025759, EPI_ISL_1025760, EPI_ISL_1025761, EPI_ISL_1025762, EPI_ISL_1025763, EPI_ISL_1025764, EPI_ISL_1025765, EPI_ISL_1025766, EPI_ISL_1025767, EPI_ISL_1025768, EPI_ISL_1025769, EPI_ISL_1025770, EPI_ISL_1025771, EPI_ISL_1025772, EPI_ISL_1025773, EPI_ISL_1025774, EPI_ISL_1025775, EPI_ISL_1025776, EPI_ISL_1025777, EPI_ISL_1025778, EPI_ISL_1025779, EPI_ISL_1025780, EPI_ISL_1025781, EPI_ISL_1025782, EPI_ISL_1025783, EPI_ISL_1025784, EPI_ISL_1025785, EPI_ISL_1025786, EPI_ISL_1025787, EPI_ISL_1025788, EPI_ISL_1025789, EPI_ISL_1025790, EPI_ISL_1025791, EPI_ISL_1025792, EPI_ISL_1025793, EPI_ISL_1025794, EPI_ISL_1025795, EPI_ISL_1025796, EPI_ISL_1025797, EPI_ISL_1025798, EPI_ISL_1025799, EPI_ISL_1025800, EPI_ISL_1025801, EPI_ISL_1025802, EPI_ISL_1025803, EPI_ISL_1025804, EPI_ISL_1025805, EPI_ISL_1025806, EPI_ISL_1025807, EPI_ISL_1025808, EPI_ISL_1025809, EPI_ISL_1025810, EPI_ISL_1025811, EPI_ISL_1025812, EPI_ISL_1025813, EPI_ISL_1025814, EPI_ISL_1025815, EPI_ISL_1025816, EPI_ISL_1025817, EPI_ISL_1025818, EPI_ISL_1025819, EPI_ISL_1025820, EPI_ISL_1025821, EPI_ISL_1025822, EPI_ISL_1025823, EPI_ISL_1025824, EPI_ISL_1025825, EPI_ISL_1025826, EPI_ISL_1025827, EPI_ISL_1025828, EPI_ISL_1025829, EPI_ISL_1025830, EPI_ISL_1025831, EPI_ISL_1025832, EPI_ISL_1025833, EPI_ISL_1025834, EPI_ISL_1025835, EPI_ISL_1025836, EPI_ISL_1025837, EPI_ISL_1025838, EPI_ISL_1025839, EPI_ISL_1025840, EPI_ISL_1025841, EPI_ISL_1025842, EPI_ISL_1025843, EPI_ISL_1025844, EPI_ISL_1025845, EPI_ISL_1025846, EPI_ISL_1025847, EPI_ISL_1025848, EPI_ISL_1025849, EPI_ISL_1025850, EPI_ISL_1025851, EPI_ISL_1025852, EPI_ISL_1025853, EPI_ISL_1025854, EPI_ISL_1025855, EPI_ISL_1025856, EPI_ISL_1025857, EPI_ISL_1025858, EPI_ISL_1025859, EPI_ISL_1025860, EPI_ISL_1025861, EPI_ISL_1025862, EPI_ISL_1025863, EPI_ISL_1025864, EPI_ISL_1025865, EPI_ISL_1025866, EPI_ISL_1025867, EPI_ISL_1025868, EPI_ISL_1025869, EPI_ISL_1025870, EPI_ISL_1025871, EPI_ISL_1025872, EPI_ISL_1025873, EPI_ISL_1025874, EPI_ISL_1025875, EPI_ISL_1025876, EPI_ISL_1025877, EPI_ISL_1025878, EPI_ISL_1025879, EPI_ISL_1025880, EPI_ISL_1025881, EPI_ISL_1025882, EPI_ISL_1025883, EPI_ISL_1025884, EPI_ISL_1025885, EPI_ISL_1025886, EPI_ISL_1025887, EPI_ISL_1025888, EPI_ISL_1025889, EPI_ISL_1025890, EPI_ISL_1025891, EPI_ISL_1025892, EPI_ISL_1025893, EPI_ISL_1025894, EPI_ISL_1025895, EPI_ISL_1025896, EPI_ISL_1025897, EPI_ISL_1025898, EPI_ISL_1025899, EPI_ISL_1025900, EPI_ISL_1025901, EPI_ISL_1025902, EPI_ISL_1025903, EPI_ISL_1025904, EPI_ISL_1025905, EPI_ISL_1025906, EPI_ISL_1025907, EPI_ISL_1065100, EPI_ISL_1065101, EPI_ISL_1065112, EPI_ISL_1065114, EPI_ISL_1065115, EPI_ISL_1065116, EPI_ISL_1065117, EPI_ISL_1065119, EPI_ISL_1065126, EPI_ISL_1065128, EPI_ISL_1065132, EPI_ISL_1065134, EPI_ISL_1065145, EPI_ISL_1065147, EPI_ISL_1065149, EPI_ISL_1065153, EPI_ISL_1065162, EPI_ISL_1065169, EPI_ISL_1065171, EPI_ISL_1065187, EPI_ISL_1065188, EPI_ISL_1065194, EPI_ISL_1065196, EPI_ISL_1065202, EPI_ISL_1065206, EPI_ISL_1065208, EPI_ISL_1065213, EPI_ISL_1065217, EPI_ISL_1065218, EPI_ISL_1065233, EPI_ISL_1065234, EPI_ISL_1065242, EPI_ISL_1065245, EPI_ISL_1065248, EPI_ISL_1065250, EPI_ISL_1065251, EPI_ISL_1065258, EPI_ISL_1065259, EPI_ISL_1065267, EPI_ISL_1065272, EPI_ISL_1065273, EPI_ISL_1065279, EPI_ISL_1065282, EPI_ISL_1065284, EPI_ISL_1065285, EPI_ISL_1065286, EPI_ISL_1065290, EPI_ISL_1065291, EPI_ISL_1065296, EPI_ISL_1065301, EPI_ISL_1065302, EPI_ISL_1065304, EPI_ISL_1065309, EPI_ISL_1065311, EPI_ISL_1065313, EPI_ISL_1065314, EPI_ISL_1065318, EPI_ISL_1065322, EPI_ISL_1065326, EPI_ISL_1065327, EPI_ISL_1065328, EPI_ISL_1065329, EPI_ISL_1065340, EPI_ISL_1065343, EPI_ISL_1065347, EPI_ISL_1065348, EPI_ISL_1065352, EPI_ISL_1065359, EPI_ISL_1065363, EPI_ISL_1065366, EPI_ISL_1065371, EPI_ISL_1065374, EPI_ISL_1065376, EPI_ISL_1065377, EPI_ISL_1065378, EPI_ISL_1065387, EPI_ISL_1065388, EPI_ISL_1065393, EPI_ISL_1065395, EPI_ISL_1065397, EPI_ISL_1065399, EPI_ISL_1065402, EPI_ISL_1065407, EPI_ISL_1065421, EPI_ISL_1065422, EPI_ISL_1065428, EPI_ISL_1065431, EPI_ISL_1065438, EPI_ISL_1065444, EPI_ISL_1065446, EPI_ISL_1065452, EPI_ISL_1065458, EPI_ISL_1065460, EPI_ISL_1065461, EPI_ISL_1065462, EPI_ISL_1065463, EPI_ISL_1065464, EPI_ISL_1065465, EPI_ISL_1065466, EPI_ISL_1065468, EPI_ISL_1065474, EPI_ISL_1065475, EPI_ISL_1065487, EPI_ISL_1065491, EPI_ISL_1065497, EPI_ISL_1065509, EPI_ISL_1065510, EPI_ISL_1065515, EPI_ISL_1065517, EPI_ISL_1065521, EPI_ISL_1065522, EPI_ISL_1065523, EPI_ISL_1065528, EPI_ISL_1065529, EPI_ISL_1065531, EPI_ISL_1065533, EPI_ISL_1065534, EPI_ISL_1065541, EPI_ISL_1065544, EPI_ISL_1065545, EPI_ISL_1065550, EPI_ISL_1065551, EPI_ISL_1065553, EPI_ISL_1065556, EPI_ISL_1065562, EPI_ISL_1065564, EPI_ISL_1065566, EPI_ISL_1065567, EPI_ISL_1065571, EPI_ISL_1065576, EPI_ISL_1065577, EPI_ISL_1065579, EPI_ISL_1065580, EPI_ISL_1065581, EPI_ISL_1065582, EPI_ISL_1065593, EPI_ISL_1065595, EPI_ISL_1065597, EPI_ISL_1065601, EPI_ISL_1065602, EPI_ISL_1065604, EPI_ISL_1065610, EPI_ISL_1065611, EPI_ISL_1065616, EPI_ISL_1065618, EPI_ISL_1065620, EPI_ISL_1065621, EPI_ISL_1065625, EPI_ISL_1065626, EPI_ISL_1065636, EPI_ISL_1065638, EPI_ISL_1065640, EPI_ISL_1065642, EPI_ISL_1065643, EPI_ISL_1065651, EPI_ISL_1065655, EPI_ISL_1065658, EPI_ISL_1065659, EPI_ISL_1065660, EPI_ISL_1065666, EPI_ISL_1065671, EPI_ISL_1065674, EPI_ISL_1065675, EPI_ISL_1065677, EPI_ISL_1065678, EPI_ISL_1065679, EPI_ISL_1065685, EPI_ISL_1065687, EPI_ISL_1065696, EPI_ISL_1065699, EPI_ISL_1065700, EPI_ISL_1065702, EPI_ISL_1065707, EPI_ISL_1065708, EPI_ISL_1065710, EPI_ISL_1065715, EPI_ISL_1065717, EPI_ISL_1065719, EPI_ISL_1065720, EPI_ISL_1065723, EPI_ISL_1065724, EPI_ISL_1065728, EPI_ISL_1065729, EPI_ISL_1065732, EPI_ISL_1065733, EPI_ISL_1065735, EPI_ISL_1065744, EPI_ISL_1065745, EPI_ISL_1065748, EPI_ISL_1065753, EPI_ISL_1065757, EPI_ISL_1065760, EPI_ISL_1065761, EPI_ISL_1065762, EPI_ISL_1065770, EPI_ISL_1065774, EPI_ISL_1065777, EPI_ISL_1065778, EPI_ISL_1065780, EPI_ISL_1065781, EPI_ISL_1065787, EPI_ISL_1065788, EPI_ISL_1065789, EPI_ISL_1065790, EPI_ISL_1065795, EPI_ISL_1065796, EPI_ISL_1065799, EPI_ISL_1065803, EPI_ISL_1065805, EPI_ISL_1065806, EPI_ISL_1065807, EPI_ISL_1065810, EPI_ISL_1065814, EPI_ISL_1065816, EPI_ISL_1065817, EPI_ISL_1065820, EPI_ISL_1065825, EPI_ISL_1065829, EPI_ISL_1065831, EPI_ISL_1065833, EPI_ISL_1065835, EPI_ISL_1065837, EPI_ISL_1065838, EPI_ISL_1065839, EPI_ISL_1065847, EPI_ISL_1065849, EPI_ISL_1065851, EPI_ISL_1065852, EPI_ISL_1065853, EPI_ISL_1065856, EPI_ISL_1065868, EPI_ISL_1065869, EPI_ISL_1065870, EPI_ISL_1065883, EPI_ISL_1065884, EPI_ISL_1065890, EPI_ISL_1065893, EPI_ISL_1065896, EPI_ISL_1065901, EPI_ISL_1065902, EPI_ISL_1065909, EPI_ISL_1065921, EPI_ISL_1065923, EPI_ISL_1065925, EPI_ISL_1065930, EPI_ISL_1065932, EPI_ISL_1065941, EPI_ISL_1065942, EPI_ISL_1065947, EPI_ISL_1065956, EPI_ISL_1065959, EPI_ISL_1065969, EPI_ISL_1065971, EPI_ISL_1065975, EPI_ISL_1065978, EPI_ISL_1065984, EPI_ISL_1065985, EPI_ISL_1065988, EPI_ISL_1065992, EPI_ISL_1065994, EPI_ISL_1065998, EPI_ISL_1066003, EPI_ISL_1066006, EPI_ISL_1066008, EPI_ISL_1066017, EPI_ISL_1066023, EPI_ISL_1066028, EPI_ISL_1066029, EPI_ISL_1066030, EPI_ISL_1066032, EPI_ISL_1066034, EPI_ISL_1066036, EPI_ISL_1066038, EPI_ISL_1066043, EPI_ISL_1066052, EPI_ISL_1066057, EPI_ISL_1066058, EPI_ISL_1066059, EPI_ISL_1066060, EPI_ISL_1066072, EPI_ISL_1066073, EPI_ISL_1066075, EPI_ISL_1066083, EPI_ISL_1066084, EPI_ISL_1066085, EPI_ISL_1066091, EPI_ISL_1066096, EPI_ISL_1066098, EPI_ISL_1066101, EPI_ISL_1066103, EPI_ISL_1066107, EPI_ISL_1066109, EPI_ISL_1066111, EPI_ISL_1066113, EPI_ISL_1066115, EPI_ISL_1066119, EPI_ISL_1066120, EPI_ISL_1066121, EPI_ISL_1066124, EPI_ISL_1066125, EPI_ISL_1066126, EPI_ISL_1066135, EPI_ISL_1066137, EPI_ISL_1066139, EPI_ISL_1066149, EPI_ISL_1066151, EPI_ISL_1066172, EPI_ISL_1066185, EPI_ISL_1066187, EPI_ISL_1066203, EPI_ISL_1066211, EPI_ISL_1066213, EPI_ISL_1066217, EPI_ISL_1066219, EPI_ISL_1066221, EPI_ISL_1066223, EPI_ISL_1066225, EPI_ISL_1066226, EPI_ISL_1066238, EPI_ISL_1066240, EPI_ISL_1066244, EPI_ISL_1066246, EPI_ISL_1066247, EPI_ISL_1066251, EPI_ISL_1066260, EPI_ISL_1066268, EPI_ISL_1066271, EPI_ISL_1066274, EPI_ISL_1066276, EPI_ISL_1066296, EPI_ISL_1066298, EPI_ISL_1066300, EPI_ISL_1066306, EPI_ISL_1066317, EPI_ISL_1066319, EPI_ISL_1066320, EPI_ISL_1066327, EPI_ISL_1066334, EPI_ISL_1066341, EPI_ISL_1066343, EPI_ISL_1066347, EPI_ISL_1066348, EPI_ISL_1066358, EPI_ISL_1066360, EPI_ISL_1066364, EPI_ISL_1066372, EPI_ISL_1066378, EPI_ISL_1066381, EPI_ISL_1066386, EPI_ISL_1066387, EPI_ISL_1066389, EPI_ISL_1066393, EPI_ISL_1066416, EPI_ISL_1066419, EPI_ISL_1066421, EPI_ISL_1066424, EPI_ISL_1066430, EPI_ISL_1066435, EPI_ISL_1066440, EPI_ISL_1066447, EPI_ISL_1066451, EPI_ISL_1066452, EPI_ISL_1066453, EPI_ISL_1066462, EPI_ISL_1066463, EPI_ISL_1066468, EPI_ISL_1066469, EPI_ISL_1066470, EPI_ISL_1066475, EPI_ISL_1066479, EPI_ISL_1066481, EPI_ISL_1066482, EPI_ISL_1066485, EPI_ISL_1066486, EPI_ISL_1066488, EPI_ISL_1066493, EPI_ISL_1066495, EPI_ISL_1066499, EPI_ISL_1066503, EPI_ISL_1066511, EPI_ISL_1066513, EPI_ISL_1066514, EPI_ISL_1066520, EPI_ISL_1066521, EPI_ISL_1066523, EPI_ISL_1066527, EPI_ISL_1066529, EPI_ISL_1066531, EPI_ISL_1066535, EPI_ISL_1066537, EPI_ISL_1066548, EPI_ISL_1066549, EPI_ISL_1066554, EPI_ISL_1066555, EPI_ISL_1066556, EPI_ISL_1066557, EPI_ISL_1066561, EPI_ISL_1066562, EPI_ISL_1066563, EPI_ISL_1066564, EPI_ISL_1066566, EPI_ISL_1066568, EPI_ISL_1066569, EPI_ISL_1066573, EPI_ISL_1066574, EPI_ISL_1066580, EPI_ISL_1066582, EPI_ISL_1066589, EPI_ISL_1066598, EPI_ISL_1066600, EPI_ISL_1066601, EPI_ISL_1066611, EPI_ISL_1066615, EPI_ISL_1066618, EPI_ISL_1066621, EPI_ISL_1066622, EPI_ISL_1066623, EPI_ISL_1066624, EPI_ISL_1066625, EPI_ISL_1066626, EPI_ISL_1066627, EPI_ISL_1066632, EPI_ISL_1066634, EPI_ISL_1066636, EPI_ISL_1066643, EPI_ISL_1066644, EPI_ISL_1066645, EPI_ISL_1066648, EPI_ISL_1066650, EPI_ISL_1066651, EPI_ISL_1066652, EPI_ISL_1066654, EPI_ISL_1066664, EPI_ISL_1066671, EPI_ISL_1066674, EPI_ISL_1066680, EPI_ISL_1066682, EPI_ISL_1066688, EPI_ISL_1066689, EPI_ISL_1066690, EPI_ISL_1066695, EPI_ISL_1066702, EPI_ISL_1066704, EPI_ISL_1066706, EPI_ISL_1066707, EPI_ISL_1066709, EPI_ISL_1066711, EPI_ISL_1066713, EPI_ISL_1066718, EPI_ISL_1066720, EPI_ISL_1066725, EPI_ISL_1066733, EPI_ISL_1066735, EPI_ISL_1066737, EPI_ISL_1066738, EPI_ISL_1066745, EPI_ISL_1066746, EPI_ISL_1066749, EPI_ISL_1066752, EPI_ISL_1066755, EPI_ISL_1066779, EPI_ISL_1066785, EPI_ISL_1066791, EPI_ISL_1066809, EPI_ISL_1066817, EPI_ISL_1066820, EPI_ISL_1066825, EPI_ISL_1066826, EPI_ISL_1066827, EPI_ISL_1066834, EPI_ISL_1066839, EPI_ISL_1066841, EPI_ISL_1066842, EPI_ISL_1066844, EPI_ISL_1066850, EPI_ISL_1066852, EPI_ISL_1066853, EPI_ISL_1066857, EPI_ISL_1066858, EPI_ISL_1066859, EPI_ISL_1066860, EPI_ISL_1066862, EPI_ISL_1066864, EPI_ISL_1066869, EPI_ISL_1066873, EPI_ISL_1066878, EPI_ISL_1066887, EPI_ISL_1066891, EPI_ISL_1066897, EPI_ISL_1066903, EPI_ISL_1066904, EPI_ISL_1066906, EPI_ISL_1066912, EPI_ISL_1066921, EPI_ISL_1066923, EPI_ISL_1066926, EPI_ISL_1066931, EPI_ISL_1066935, EPI_ISL_1066937, EPI_ISL_1066941, EPI_ISL_1066943, EPI_ISL_1066946, EPI_ISL_1066948, EPI_ISL_1066950, EPI_ISL_1066952, EPI_ISL_1066955, EPI_ISL_1066957, EPI_ISL_1066963, EPI_ISL_1066964, EPI_ISL_1066965, EPI_ISL_1066968, EPI_ISL_1066969, EPI_ISL_1066970, EPI_ISL_1066973, EPI_ISL_1066974, EPI_ISL_1066975, EPI_ISL_1066979, EPI_ISL_1066980, EPI_ISL_1066985, EPI_ISL_1066986, EPI_ISL_1066987, EPI_ISL_1066995, EPI_ISL_1066996, EPI_ISL_1066999, EPI_ISL_1067003, EPI_ISL_1067006, EPI_ISL_1067007, EPI_ISL_1067013, EPI_ISL_1067014, EPI_ISL_1067015, EPI_ISL_1067016, EPI_ISL_1067018, EPI_ISL_1067020, EPI_ISL_1067021, EPI_ISL_1067022, EPI_ISL_1067029, EPI_ISL_1067036, EPI_ISL_1067038, EPI_ISL_1067044, EPI_ISL_1067045, EPI_ISL_1067046, EPI_ISL_1067052, EPI_ISL_1067054, EPI_ISL_1067066, EPI_ISL_1067068, EPI_ISL_1067072, EPI_ISL_1067075, EPI_ISL_1067080, EPI_ISL_1067090, EPI_ISL_1067091, EPI_ISL_1067094, EPI_ISL_1067110, EPI_ISL_1067124, EPI_ISL_1067125, EPI_ISL_1067132, EPI_ISL_1067133, EPI_ISL_1067137, EPI_ISL_1067141, EPI_ISL_1067142, EPI_ISL_1067143, EPI_ISL_1067145, EPI_ISL_1067148, EPI_ISL_1067152, EPI_ISL_1067156, EPI_ISL_1067158, EPI_ISL_1067161, EPI_ISL_1067163, EPI_ISL_1067164, EPI_ISL_1067168, EPI_ISL_1067172, EPI_ISL_1067176, EPI_ISL_1067177, EPI_ISL_1067184, EPI_ISL_1067188, EPI_ISL_1067193, EPI_ISL_1067197, EPI_ISL_1067199, EPI_ISL_1067204, EPI_ISL_1067205, EPI_ISL_1067210, EPI_ISL_1067211, EPI_ISL_1067212, EPI_ISL_1067216, EPI_ISL_1067217, EPI_ISL_1067224, EPI_ISL_1067229, EPI_ISL_1067235, EPI_ISL_1067237, EPI_ISL_1067241, EPI_ISL_1067244, EPI_ISL_1067246, EPI_ISL_1067249, EPI_ISL_1067253, EPI_ISL_1067256, EPI_ISL_1067257, EPI_ISL_1067258, EPI_ISL_1067264, EPI_ISL_1067267, EPI_ISL_1067268, EPI_ISL_1067274, EPI_ISL_1067277, EPI_ISL_1067279, EPI_ISL_1067280, EPI_ISL_1067281, EPI_ISL_1067284, EPI_ISL_1067285, EPI_ISL_1067286, EPI_ISL_1067289, EPI_ISL_1067294, EPI_ISL_1067296, EPI_ISL_1067302, EPI_ISL_1067307, EPI_ISL_1067309, EPI_ISL_1067313, EPI_ISL_1067315, EPI_ISL_1067318, EPI_ISL_1067321, EPI_ISL_1067323, EPI_ISL_1067325, EPI_ISL_1067332, EPI_ISL_1067339, EPI_ISL_1067342, EPI_ISL_1067350, EPI_ISL_1067352, EPI_ISL_1067355, EPI_ISL_1067357, EPI_ISL_1067358, EPI_ISL_1067359, EPI_ISL_1067365, EPI_ISL_1067368, EPI_ISL_1067374, EPI_ISL_1067380, EPI_ISL_1067381, EPI_ISL_1067382, EPI_ISL_1067383, EPI_ISL_1067384, EPI_ISL_1067385, EPI_ISL_1067386, EPI_ISL_1067395, EPI_ISL_1067396, EPI_ISL_1067401, EPI_ISL_1067405, EPI_ISL_1067407, EPI_ISL_1067410, EPI_ISL_1067411, EPI_ISL_1067415, EPI_ISL_1067425, EPI_ISL_1067437, EPI_ISL_1067438, EPI_ISL_1067440, EPI_ISL_1067441, EPI_ISL_1067444, EPI_ISL_1067445, EPI_ISL_1067446, EPI_ISL_1067450, EPI_ISL_1067462, EPI_ISL_1067469, EPI_ISL_1067473, EPI_ISL_1067483, EPI_ISL_1067490, EPI_ISL_1067491, EPI_ISL_1067493, EPI_ISL_1067495, EPI_ISL_1067500, EPI_ISL_1067501, EPI_ISL_1067506, EPI_ISL_1067512, EPI_ISL_1067513, EPI_ISL_1067519, EPI_ISL_1067523, EPI_ISL_1067525, EPI_ISL_1067530, EPI_ISL_1067531, EPI_ISL_1067533, EPI_ISL_1067537, EPI_ISL_1067538, EPI_ISL_1067543, EPI_ISL_1067549, EPI_ISL_1067555, EPI_ISL_1067558, EPI_ISL_1067559, EPI_ISL_1067561, EPI_ISL_1067568, EPI_ISL_1067569, EPI_ISL_1067574, EPI_ISL_1067578, EPI_ISL_1067579, EPI_ISL_1067582			
see above	Department of Virus and Microbiological Special Diagnostics, Statens Serum Institut, Copenhagen, Denmark	Aalborg University	Danish Covid-19 Genome Consortium
EPI_ISL_712099, EPI_ISL_712100, EPI_ISL_712119, EPI_ISL_712120, EPI_ISL_712121, EPI_ISL_712122, EPI_ISL_712123, EPI_ISL_712124, EPI_ISL_712125, EPI_ISL_712126, EPI_ISL_712127, EPI_ISL_712128, EPI_ISL_712250, EPI_ISL_712251, EPI_ISL_712252, EPI_ISL_712253, EPI_ISL_712295, EPI_ISL_712321, EPI_ISL_712340, EPI_ISL_712341, EPI_ISL_712342, EPI_ISL_712343, EPI_ISL_712344, EPI_ISL_712345, EPI_ISL_712346, EPI_ISL_712417, EPI_ISL_712431, EPI_ISL_712439, EPI_ISL_712446, EPI_ISL_712447, EPI_ISL_712448, EPI_ISL_712455, EPI_ISL_712456, EPI_ISL_712457, EPI_ISL_712458, EPI_ISL_712459, EPI_ISL_712460, EPI_ISL_712461, EPI_ISL_712462, EPI_ISL_712463, EPI_ISL_712464, EPI_ISL_712465, EPI_ISL_712466, EPI_ISL_712467, EPI_ISL_712468, EPI_ISL_712469, EPI_ISL_712470, EPI_ISL_712471, EPI_ISL_712472, EPI_ISL_712473, EPI_ISL_712474, EPI_ISL_712475, EPI_ISL_712476, EPI_ISL_712477, EPI_ISL_712478, EPI_ISL_712479, EPI_ISL_712480, EPI_ISL_712481, EPI_ISL_712482, EPI_ISL_712483, EPI_ISL_712484, EPI_ISL_712485, EPI_ISL_712486, EPI_ISL_712487, EPI_ISL_712488, EPI_ISL_712489, EPI_ISL_712490, EPI_ISL_712491, EPI_ISL_712492, EPI_ISL_712493, EPI_ISL_712494, EPI_ISL_712495, EPI_ISL_712496, EPI_ISL_712497, EPI_ISL_712498, EPI_ISL_712499, EPI_ISL_712500, EPI_ISL_712501, EPI_ISL_712502, EPI_ISL_712503, EPI_ISL_712504, EPI_ISL_712505, EPI_ISL_712506, EPI_ISL_712507, EPI_ISL_712508, EPI_ISL_712509, EPI_ISL_712510, EPI_ISL_712511, EPI_ISL_712512, EPI_ISL_712513, EPI_ISL_712514, EPI_ISL_712515, EPI_ISL_712516, EPI_ISL_712517, EPI_ISL_712518, EPI_ISL_712519, EPI_ISL_712520, EPI_ISL_712521, EPI_ISL_712522, EPI_ISL_712523, EPI_ISL_712524, EPI_ISL_712525, EPI_ISL_712526, EPI_ISL_712527, EPI_ISL_712528, EPI_ISL_712529, EPI_ISL_712530, EPI_ISL_747527, EPI_ISL_747528, EPI_ISL_747529, EPI_ISL_747533, EPI_ISL_747535, EPI_ISL_747536, EPI_ISL_747540, EPI_ISL_747541, EPI_ISL_747542, EPI_ISL_747543, EPI_ISL_747544, EPI_ISL_747545, EPI_ISL_747546, EPI_ISL_747547, EPI_ISL_747548, EPI_ISL_747549, EPI_ISL_747550, EPI_ISL_747551, EPI_ISL_747552, EPI_ISL_747553, EPI_ISL_747554, EPI_ISL_747555, EPI_ISL_747556, EPI_ISL_747557, EPI_ISL_747558, EPI_ISL_747559, EPI_ISL_747560, EPI_ISL_747561, EPI_ISL_747562, EPI_ISL_747563, EPI_ISL_747564, EPI_ISL_747565, EPI_ISL_747566, EPI_ISL_747567, EPI_ISL_747568, EPI_ISL_747569, EPI_ISL_747570, EPI_ISL_747571, EPI_ISL_747572, EPI_ISL_747573, EPI_ISL_747574, EPI_ISL_747575, EPI_ISL_747576, EPI_ISL_747589, EPI_ISL_747590, EPI_ISL_747591, EPI_ISL_747592, EPI_ISL_747593, EPI_ISL_747594, EPI_ISL_747595, EPI_ISL_747599, EPI_ISL_747601, EPI_ISL_747602, EPI_ISL_747607, EPI_ISL_747608, EPI_ISL_747610, EPI_ISL_747611, EPI_ISL_747612, EPI_ISL_747613, EPI_ISL_747614, EPI_ISL_747616, EPI_ISL_747617, EPI_ISL_747618, EPI_ISL_747619, EPI_ISL_747620, EPI_ISL_747621, EPI_ISL_747622, EPI_ISL_747623, EPI_ISL_747632, EPI_ISL_747636, EPI_ISL_747637, EPI_ISL_747638, EPI_ISL_747639, EPI_ISL_747640, EPI_ISL_747642, EPI_ISL_747643, EPI_ISL_747644, EPI_ISL_747645, EPI_ISL_747648, EPI_ISL_747652, EPI_ISL_747655, EPI_ISL_747656, EPI_ISL_747658, EPI_ISL_747659, EPI_ISL_747660, EPI_ISL_747661, EPI_ISL_747662, EPI_ISL_747663, EPI_ISL_747664, EPI_ISL_747665, EPI_ISL_747666, EPI_ISL_747667, EPI_ISL_747668, EPI_ISL_747669, EPI_ISL_747670, EPI_ISL_747671, EPI_ISL_747672, EPI_ISL_747673, EPI_ISL_747674, EPI_ISL_747681, EPI_ISL_747682, EPI_ISL_747683, EPI_ISL_747684, EPI_ISL_747685, EPI_ISL_747686, EPI_ISL_747687, EPI_ISL_747688, EPI_ISL_747689, EPI_ISL_747690, EPI_ISL_747691, EPI_ISL_747692, EPI_ISL_747693, EPI_ISL_747694, EPI_ISL_747695, EPI_ISL_747696, EPI_ISL_747697, EPI_ISL_747698, EPI_ISL_747699, EPI_ISL_747700, EPI_ISL_747701, EPI_ISL_747702, EPI_ISL_747703, EPI_ISL_747704, EPI_ISL_747705, EPI_ISL_747706, EPI_ISL_747707, EPI_ISL_747708, EPI_ISL_747721, EPI_ISL_747722, EPI_ISL_747723, EPI_ISL_747728, EPI_ISL_747729, EPI_ISL_747731, EPI_ISL_747732, EPI_ISL_747733, EPI_ISL_747734, EPI_ISL_747739, EPI_ISL_747740, EPI_ISL_747741, EPI_ISL_747742, EPI_ISL_747743, EPI_ISL_747745, EPI_ISL_747746, EPI_ISL_747748, EPI_ISL_747750, EPI_ISL_747752, EPI_ISL_747753, EPI_ISL_747754, EPI_ISL_747756, EPI_ISL_747757, EPI_ISL_747758, EPI_ISL_747759, EPI_ISL_747760, EPI_ISL_747761, EPI_ISL_747762, EPI_ISL_747763, EPI_ISL_747764, EPI_ISL_747765, EPI_ISL_747766, EPI_ISL_747767, EPI_ISL_747768, EPI_ISL_747769, EPI_ISL_747770, EPI_ISL_747771, EPI_ISL_747772, EPI_ISL_747773, EPI_ISL_747774, EPI_ISL_747775, EPI_ISL_747776, EPI_ISL_747777, EPI_ISL_747778, EPI_ISL_747779, EPI_ISL_747780, EPI_ISL_747781, EPI_ISL_747782, EPI_ISL_747783, EPI_ISL_747784, EPI_ISL_747785, EPI_ISL_747786, EPI_ISL_747787, EPI_ISL_747788, EPI_ISL_747789, EPI_ISL_747790, EPI_ISL_747791, EPI_ISL_747792, EPI_ISL_747793, EPI_ISL_747794, EPI_ISL_747795, EPI_ISL_747796, EPI_ISL_747797, EPI_ISL_747798, EPI_ISL_747799, EPI_ISL_747800, EPI_ISL_747801, EPI_ISL_747802, EPI_ISL_747803, EPI_ISL_747804, EPI_ISL_747805, EPI_ISL_747806,			

We gratefully acknowledge the following Authors from the Originating laboratories responsible for obtaining the specimens, as well as the Submitting laboratories where the genome data were generated and shared via GISAID, on which this research is based.

All Submitters of data may be contacted directly via www.gisaid.org

Authors are sorted alphabetically.

Accession ID	Originating Laboratory	Submitting Laboratory	Authors

EPI_ISL_1025345, EPI_ISL_1025346, EPI_ISL_1025347, EPI_ISL_1025348, EPI_ISL_1025349, EPI_ISL_1025350, EPI_ISL_1025351, EPI_ISL_1025352, EPI_ISL_1025353, EPI_ISL_1025354, EPI_ISL_1025355, EPI_ISL_1025356, EPI_ISL_1025357, EPI_ISL_1025358, EPI_ISL_1025359, EPI_ISL_1025360, EPI_ISL_1025361, EPI_ISL_1025362, EPI_ISL_1025363, EPI_ISL_1025364, EPI_ISL_1025365, EPI_ISL_1025366, EPI_ISL_1025367, EPI_ISL_1025368, EPI_ISL_1025369, EPI_ISL_1025370, EPI_ISL_1025371, EPI_ISL_1025372, EPI_ISL_1025373, EPI_ISL_1025374, EPI_ISL_1025375, EPI_ISL_1025376, EPI_ISL_1025377, EPI_ISL_1025378, EPI_ISL_1025379, EPI_ISL_1025380, EPI_ISL_1025381, EPI_ISL_1025382, EPI_ISL_1025383, EPI_ISL_1025384, EPI_ISL_1025385, EPI_ISL_1025386, EPI_ISL_1025387, EPI_ISL_1025388, EPI_ISL_1025389, EPI_ISL_1025390, EPI_ISL_1025391, EPI_ISL_1025392, EPI_ISL_1025393, EPI_ISL_1025394, EPI_ISL_1025395, EPI_ISL_1025396, EPI_ISL_1025397, EPI_ISL_1025398, EPI_ISL_1025399, EPI_ISL_1025400, EPI_ISL_1025401, EPI_ISL_1025402, EPI_ISL_1025403, EPI_ISL_1025404, EPI_ISL_1025405, EPI_ISL_1025406, EPI_ISL_1025407, EPI_ISL_1025408, EPI_ISL_1025409, EPI_ISL_1025410, EPI_ISL_1025411, EPI_ISL_1025412, EPI_ISL_1025413, EPI_ISL_1025414, EPI_ISL_1025415, EPI_ISL_1025416, EPI_ISL_1025417, EPI_ISL_1025418, EPI_ISL_1025419, EPI_ISL_1025420, EPI_ISL_1025421, EPI_ISL_1025422, EPI_ISL_1025423, EPI_ISL_1025424, EPI_ISL_1025425, EPI_ISL_1025426, EPI_ISL_1025427, EPI_ISL_1025428, EPI_ISL_1025429, EPI_ISL_1025430, EPI_ISL_1025431, EPI_ISL_1025432, EPI_ISL_1025433, EPI_ISL_1025434, EPI_ISL_1025435, EPI_ISL_1025436, EPI_ISL_1025437, EPI_ISL_1025438, EPI_ISL_1025439, EPI_ISL_1025440, EPI_ISL_1025441, EPI_ISL_1025442, EPI_ISL_1025443, EPI_ISL_1025444, EPI_ISL_1025445, EPI_ISL_1025446, EPI_ISL_1025447, EPI_ISL_1025448, EPI_ISL_1025449, EPI_ISL_1025450, EPI_ISL_1025451, EPI_ISL_1025452, EPI_ISL_1025453, EPI_ISL_1025454, EPI_ISL_1025455, EPI_ISL_1025456, EPI_ISL_1025457, EPI_ISL_1025458, EPI_ISL_1025459, EPI_ISL_1025460, EPI_ISL_1025461, EPI_ISL_1025462, EPI_ISL_1025463, EPI_ISL_1025464, EPI_ISL_1025465, EPI_ISL_1025466, EPI_ISL_1025467, EPI_ISL_1025468, EPI_ISL_1025469, EPI_ISL_1025470, EPI_ISL_1025471, EPI_ISL_1025472, EPI_ISL_1025473, EPI_ISL_1025474, EPI_ISL_1025475, EPI_ISL_1025476, EPI_ISL_1025477, EPI_ISL_1025478, EPI_ISL_1025479, EPI_ISL_1025480, EPI_ISL_1025481, EPI_ISL_1025482, EPI_ISL_1025483, EPI_ISL_1025484, EPI_ISL_1025485, EPI_ISL_1025486, EPI_ISL_1025487, EPI_ISL_1025488, EPI_ISL_1025489, EPI_ISL_1025490, EPI_ISL_1025491, EPI_ISL_1025492, EPI_ISL_1025493, EPI_ISL_1025494, EPI_ISL_1025495, EPI_ISL_1025496, EPI_ISL_1025497, EPI_ISL_1025498, EPI_ISL_1025499, EPI_ISL_1025500, EPI_ISL_1025501, EPI_ISL_1025502, EPI_ISL_1025503, EPI_ISL_1025504, EPI_ISL_1025505, EPI_ISL_1025506, EPI_ISL_1025507, EPI_ISL_1025508, EPI_ISL_1025509, EPI_ISL_1025510, EPI_ISL_1025511, EPI_ISL_1025512, EPI_ISL_1025513, EPI_ISL_1025514, EPI_ISL_1025515, EPI_ISL_1025516, EPI_ISL_1025517, EPI_ISL_1025518, EPI_ISL_1025519, EPI_ISL_1025520, EPI_ISL_1025521, EPI_ISL_1025522, EPI_ISL_1025523, EPI_ISL_1025524, EPI_ISL_1025525, EPI_ISL_1025526, EPI_ISL_1025527, EPI_ISL_1025528, EPI_ISL_1025529, EPI_ISL_1025530, EPI_ISL_1025531, EPI_ISL_1025532, EPI_ISL_1025533, EPI_ISL_1025534, EPI_ISL_1025535, EPI_ISL_1025536, EPI_ISL_1025537, EPI_ISL_1025538, EPI_ISL_1025539, EPI_ISL_1025540, EPI_ISL_1025541, EPI_ISL_1025542, EPI_ISL_1025543, EPI_ISL_1025544, EPI_ISL_1025545, EPI_ISL_1025546, EPI_ISL_1025547, EPI_ISL_1025548, EPI_ISL_1025549, EPI_ISL_1025550, EPI_ISL_1025551, EPI_ISL_1025552, EPI_ISL_1025553, EPI_ISL_1025554, EPI_ISL_1025555, EPI_ISL_1025556, EPI_ISL_1025557, EPI_ISL_1025558, EPI_ISL_1025559, EPI_ISL_1025560, EPI_ISL_1025561, EPI_ISL_1025562, EPI_ISL_1025563, EPI_ISL_1025564, EPI_ISL_1025565, EPI_ISL_1025566, EPI_ISL_1025567, EPI_ISL_1025568, EPI_ISL_1025569, EPI_ISL_1025570, EPI_ISL_1025571, EPI_ISL_1025572, EPI_ISL_1025573, EPI_ISL_1025574, EPI_ISL_1025575, EPI_ISL_1025576, EPI_ISL_1025577, EPI_ISL_1025578, EPI_ISL_1025579, EPI_ISL_1025580, EPI_ISL_1025581, EPI_ISL_1025582, EPI_ISL_1025583, EPI_ISL_1025584, EPI_ISL_1025585, EPI_ISL_1025586, EPI_ISL_1025587, EPI_ISL_1025588, EPI_ISL_1025589, EPI_ISL_1025590, EPI_ISL_1025591, EPI_ISL_1025592, EPI_ISL_1025593, EPI_ISL_1025594, EPI_ISL_1025595, EPI_ISL_1025596, EPI_ISL_1025597, EPI_ISL_1025598, EPI_ISL_1025599, EPI_ISL_1025600, EPI_ISL_1025601, EPI_ISL_1025602, EPI_ISL_1025603, EPI_ISL_1025604, EPI_ISL_1025605, EPI_ISL_1025606, EPI_ISL_1025607, EPI_ISL_1025608, EPI_ISL_1025609, EPI_ISL_1025610, EPI_ISL_1025611, EPI_ISL_1025612, EPI_ISL_1025613, EPI_ISL_1025614, EPI_ISL_1025615, EPI_ISL_1025616, EPI_ISL_1025617, EPI_ISL_1025618, EPI_ISL_1025619, EPI_ISL_1025620, EPI_ISL_1025621, EPI_ISL_1025622, EPI_ISL_1025623, EPI_ISL_1025624, EPI_ISL_1025625, EPI_ISL_1025626, EPI_ISL_1025627, EPI_ISL_1025628, EPI_ISL_1025629, EPI_ISL_1025630, EPI_ISL_1025631, EPI_ISL_1025632, EPI_ISL_1025633, EPI_ISL_1025634, EPI_ISL_1025635, EPI_ISL_1025636, EPI_ISL_1025637, EPI_ISL_1025638, EPI_ISL_1025639, EPI_ISL_1025640, EPI_ISL_1025641, EPI_ISL_1025642, EPI_ISL_1025643, EPI_ISL_1025644, EPI_ISL_1025645, EPI_ISL_1025646, EPI_ISL_1025647, EPI_ISL_1025648, EPI_ISL_1025649, EPI_ISL_1025650, EPI_ISL_1025651, EPI_ISL_1025652, EPI_ISL_1025653, EPI_ISL_1025654, EPI_ISL_1025655, EPI_ISL_1025656, EPI_ISL_1025657, EPI_ISL_1025658, EPI_ISL_1025659, EPI_ISL_1025660, EPI_ISL_1025661, EPI_ISL_1025662, EPI_ISL_1025663, EPI_ISL_1025664, EPI_ISL_1025665, EPI_ISL_1025666, EPI_ISL_1025667, EPI_ISL_1025668, EPI_ISL_1025669, EPI_ISL_1065105, EPI_ISL_1065111, EPI_ISL_1065156, EPI_ISL_1065207, EPI_ISL_1065219, EPI_ISL_1065229, EPI_ISL_1065232, EPI_ISL_1065240, EPI_ISL_1065265, EPI_ISL_1065293, EPI_ISL_1065300, EPI_ISL_1065306, EPI_ISL_1065317, EPI_ISL_1065330, EPI_ISL_1065341, EPI_ISL_1065382, EPI_ISL_1065383, EPI_ISL_1065386, EPI_ISL_1065417, EPI_ISL_1065425, EPI_ISL_1065479, EPI_ISL_1065488, EPI_ISL_1065502, EPI_ISL_1065514, EPI_ISL_1065516, EPI_ISL_1065535, EPI_ISL_1065542, EPI_ISL_1065613, EPI_ISL_1065615, EPI_ISL_1065622, EPI_ISL_1065652, EPI_ISL_1065681, EPI_ISL_1065697, EPI_ISL_1065698, EPI_ISL_1065738, EPI_ISL_1065741, EPI_ISL_1065751, EPI_ISL_1065754, EPI_ISL_1065769, EPI_ISL_1065783, EPI_ISL_1065804, EPI_ISL_1065809, EPI_ISL_1065828, EPI_ISL_1065830, EPI_ISL_1065841, EPI_ISL_1065866, EPI_ISL_1065878, EPI_ISL_1065906, EPI_ISL_1065915, EPI_ISL_1065919, EPI_ISL_1065931, EPI_ISL_1065934, EPI_ISL_1065935, EPI_ISL_1065949, EPI_ISL_1065952, EPI_ISL_1065972, EPI_ISL_1065999, EPI_ISL_1066024, EPI_ISL_1066026, EPI_ISL_1066033, EPI_ISL_1066049, EPI_ISL_1066061, EPI_ISL_1066069, EPI_ISL_1066071, EPI_ISL_1066074, EPI_ISL_1066078, EPI_ISL_1066080, EPI_ISL_1066089, EPI_ISL_1066090, EPI_ISL_1066157, EPI_ISL_1066164, EPI_ISL_1066180, EPI_ISL_1066190, EPI_ISL_1066202, EPI_ISL_1066210, EPI_ISL_1066239, EPI_ISL_1066242, EPI_ISL_1066243, EPI_ISL_1066250, EPI_ISL_1066256, EPI_ISL_1066266, EPI_ISL_1066279, EPI_ISL_1066314, EPI_ISL_1066333, EPI_ISL_1066338, EPI_ISL_1066344, EPI_ISL_1066373, EPI_ISL_1066382, EPI_ISL_1066388, EPI_ISL_1066406, EPI_ISL_1066429, EPI_ISL_1066431, EPI_ISL_1066433, EPI_ISL_1066436, EPI_ISL_1066448, EPI_ISL_1066456, EPI_ISL_1066459, EPI_ISL_1066461, EPI_ISL_1066467, EPI_ISL_1066478, EPI_ISL_1066480, EPI_ISL_1066492, EPI_ISL_1066512, EPI_ISL_1066528, EPI_ISL_1066533, EPI_ISL_1066546, EPI_ISL_1066571, EPI_ISL_1066583, EPI_ISL_1066609, EPI_ISL_1066629, EPI_ISL_1066631, EPI_ISL_1066633, EPI_ISL_1066653, EPI_ISL_1066663, EPI_ISL_1066676, EPI_ISL_1066712, EPI_ISL_1066716, EPI_ISL_1066730, EPI_ISL_1066740, EPI_ISL_1066751, EPI_ISL_1066772, EPI_ISL_1066800, EPI_ISL_1066803, EPI_ISL_1066807, EPI_ISL_1066821, EPI_ISL_1066840, EPI_ISL_1066843, EPI_ISL_1066848, EPI_ISL_1066851, EPI_ISL_1066854, EPI_ISL_1066874, EPI_ISL_1066896, EPI_ISL_1066898, EPI_ISL_1066901, EPI_ISL_1066918, EPI_ISL_1066929, EPI_ISL_1066930, EPI_ISL_1066942, EPI_ISL_1066959, EPI_ISL_1066978, EPI_ISL_1066992, EPI_ISL_1066998, EPI_ISL_1067010, EPI_ISL_1067030, EPI_ISL_1067057, EPI_ISL_1067058, EPI_ISL_1067063, EPI_ISL_1067088, EPI_ISL_1067095, EPI_ISL_1067103, EPI_ISL_1067115, EPI_ISL_1067117, EPI_ISL_1067119, EPI_ISL_1067128, EPI_ISL_1067135, EPI_ISL_1067136, EPI_ISL_1067138, EPI_ISL_1067139, EPI_ISL_1067159, EPI_ISL_1067162, EPI_ISL_1067170, EPI_ISL_1067175, EPI_ISL_1067179, EPI_ISL_1067187, EPI_ISL_1067190, EPI_ISL_1067192, EPI_ISL_1067194, EPI_ISL_1067195, EPI_ISL_1067201, EPI_ISL_1067221, EPI_ISL_1067230, EPI_ISL_1067243, EPI_ISL_1067248, EPI_ISL_1067252, EPI_ISL_1067271, EPI_ISL_1067276, EPI_ISL_1067278, EPI_ISL_1067335, EPI_ISL_1067348, EPI_ISL_1067363, EPI_ISL_1067372, EPI_ISL_1067375, EPI_ISL_1067394, EPI_ISL_1067397, EPI_ISL_1067398, EPI_ISL_1067431, EPI_ISL_1067432, EPI_ISL_1067434, EPI_ISL_1067442, EPI_ISL_1067453, EPI_ISL_1067458, EPI_ISL_1067459, EPI_ISL_1067481, EPI_ISL_1067485, EPI_ISL_1067492, EPI_ISL_1067503, EPI_ISL_1067510, EPI_ISL_1067514, EPI_ISL_1067526, EPI_ISL_1067550, EPI_ISL_1067552, EPI_ISL_1067562, EPI_ISL_1067566

see above	Department of Virus and Microbiological Special Diagnostics, Statens Serum Institut, Copenhagen, Denmark	Aalborg University	Danish Covid-19 Genome Consortium

EPI_ISL_793030, EPI_ISL_793031, EPI_ISL_793032, EPI_ISL_793033, EPI_ISL_793034, EPI_ISL_793035, EPI_ISL_793036, EPI_ISL_793037, EPI_ISL_793038, EPI_ISL_793039, EPI_ISL_793040, EPI_ISL_793041, EPI_ISL_793042, EPI_ISL_793043, EPI_ISL_793044, EPI_ISL_793045, EPI_ISL_793046, EPI_ISL_793047, EPI_ISL_793048, EPI_ISL_793049, EPI_ISL_793050, EPI_ISL_793116, EPI_ISL_793117, EPI_ISL_793120, EPI_ISL_793130, EPI_ISL_793134, EPI_ISL_793142, EPI_ISL_793145, EPI_ISL_793150, EPI_ISL_793151, EPI_ISL_793159, EPI_ISL_793163, EPI_ISL_793164, EPI_ISL_793171, EPI_ISL_793179, EPI_ISL_793180, EPI_ISL_793181, EPI_ISL_793182, EPI_ISL_793183, EPI_ISL_793184, EPI_ISL_793185, EPI_ISL_793186, EPI_ISL_793187, EPI_ISL_793188, EPI_ISL_793189, EPI_ISL_793190, EPI_ISL_793191, EPI_ISL_793192, EPI_ISL_793193, EPI_ISL_793205, EPI_ISL_793206, EPI_ISL_793207, EPI_ISL_793208, EPI_ISL_793209, EPI_ISL_793210, EPI_ISL_793211, EPI_ISL_793212, EPI_ISL_793213, EPI_ISL_793214, EPI_ISL_793215, EPI_ISL_793216, EPI_ISL_793217, EPI_ISL_793296, EPI_ISL_793299, EPI_ISL_793301, EPI_ISL_793316, EPI_ISL_793317, EPI_ISL_793318, EPI_ISL_793319, EPI_ISL_793320, EPI_ISL_793321, EPI_ISL_793322, EPI_ISL_793323, EPI_ISL_793324, EPI_ISL_793325, EPI_ISL_793326, EPI_ISL_793327, EPI_ISL_793328, EPI_ISL_793329, EPI_ISL_793330, EPI_ISL_793331, EPI_ISL_793371, EPI_ISL_793374, EPI_ISL_793394, EPI_ISL_793396, EPI_ISL_793399, EPI_ISL_793400, EPI_ISL_793401, EPI_ISL_793402, EPI_ISL_793403, EPI_ISL_793406, EPI_ISL_793417, EPI_ISL_793421, EPI_ISL_793422, EPI_ISL_793423, EPI_ISL_793424, EPI_ISL_793425, EPI_ISL_793426, EPI_ISL_793427, EPI_ISL_793428, EPI_ISL_793429, EPI_ISL_793430, EPI_ISL_793431, EPI_ISL_793432, EPI_ISL_793433, EPI_ISL_793434, EPI_ISL_793435, EPI_ISL_793436, EPI_ISL_793437, EPI_ISL_793438, EPI_ISL_793439, EPI_ISL_793440, EPI_ISL_793441, EPI_ISL_793442, EPI_ISL_793443, EPI_ISL_793444, EPI_ISL_793445, EPI_ISL_793446, EPI_ISL_793447, EPI_ISL_793448, EPI_ISL_793449, EPI_ISL_793450, EPI_ISL_793451, EPI_ISL_793452, EPI_ISL_793453, EPI_ISL_793454, EPI_ISL_793455, EPI_ISL_793456, EPI_ISL_793457, EPI_ISL_793458, EPI_ISL_793459, EPI_ISL_793460, EPI_ISL_793461, EPI_ISL_793462, EPI_ISL_793463, EPI_ISL_793464, EPI_ISL_793465, EPI_ISL_793466, EPI_ISL_793467, EPI_ISL_793468, EPI_ISL_793469, EPI_ISL_793470, EPI_ISL_793471, EPI_ISL_793472, EPI_ISL_793473, EPI_ISL_793474, EPI_ISL_793475, EPI_ISL_793476, EPI_ISL_793477, EPI_ISL_793478, EPI_ISL_793479, EPI_ISL_793480, EPI_ISL_793481, EPI_ISL_793482, EPI_ISL_793483, EPI_ISL_793484, EPI_ISL_793485, EPI_ISL_793486, EPI_ISL_793487, EPI_ISL_793488, EPI_ISL_793489, EPI_ISL_793490, EPI_ISL_793491, EPI_ISL_793492, EPI_ISL_793493, EPI_ISL_793494, EPI_ISL_793495, EPI_ISL_793496, EPI_ISL_793497, EPI_ISL_793498, EPI_ISL_793499, EPI_ISL_793500, EPI_ISL_793501, EPI_ISL_793502, EPI_ISL_793503, EPI_ISL_793504, EPI_ISL_793505, EPI_ISL_793506, EPI_ISL_793507, EPI_ISL_793508, EPI_ISL_793509, EPI_ISL_793510, EPI_ISL_793511, EPI_ISL_793512, EPI_ISL_793513, EPI_ISL_793514, EPI_ISL_793515, EPI_ISL_793516, EPI_ISL_793517, EPI_ISL_793518, EPI_ISL_793519, EPI_ISL_793520, EPI_ISL_793521, EPI_ISL_793522, EPI_ISL_793523, EPI_ISL_793524, EPI_ISL_793525, EPI_ISL_793526, EPI_ISL_793527, EPI_ISL_793528, EPI_ISL_793529, EPI_ISL_793530, EPI_ISL_793531, EPI_ISL_793532, EPI_ISL_793533, EPI_ISL_793534, EPI_ISL_793535, EPI_ISL_793536, EPI_ISL_793537, EPI_ISL_793538, EPI_ISL_793539, EPI_ISL_793540, EPI_ISL_793541, EPI_ISL_793542, EPI_ISL_793543, EPI_ISL_793544, EPI_ISL_793545, EPI_ISL_793546, EPI_ISL_793547, EPI_ISL_793548, EPI_ISL_793549, EPI_ISL_793550, EPI_ISL_793551, EPI_ISL_793552, EPI_ISL_793553, EPI_ISL_793554, EPI_ISL_793555, EPI_ISL_793556, EPI_ISL_793557, EPI_ISL_793558, EPI_ISL_793559, EPI_ISL_793560, EPI_ISL_793561, EPI_ISL_793562, EPI_ISL_793563, EPI_ISL_793564, EPI_ISL_793565, EPI_ISL_793566, EPI_ISL_793567, EPI_ISL_793568, EPI_ISL_793569, EPI_ISL_793570, EPI_ISL_793571, EPI_ISL_793572, EPI_ISL_793573, EPI_ISL_793574, EPI_ISL_793575, EPI_ISL_793576, EPI_ISL_793577, EPI_ISL_793578, EPI_ISL_793579, EPI_ISL_793580, EPI_ISL_793581, EPI_ISL_793582, EPI_ISL_793583, EPI_ISL_793584, EPI_ISL_793585, EPI_ISL_793586, EPI_ISL_793587, EPI_ISL_793588, EPI_ISL_793589, EPI_ISL_793590, EPI_ISL_793591, EPI_ISL_793592, EPI_ISL_793593, EPI_ISL_793594, EPI_ISL_793595, EPI_ISL_793596, EPI_ISL_793597, EPI_ISL_793598, EPI_ISL_793599, EPI_ISL_793600, EPI_ISL_793601, EPI_ISL_793602, EPI_ISL_793603, EPI_ISL_793604, EPI_ISL_793605, EPI_ISL_793606, EPI_ISL_793607, EPI_ISL_793608, EPI_ISL_793609, EPI_ISL_793610, EPI_ISL_793611, EPI_ISL_793612, EPI_ISL_793613, EPI_ISL_793614, EPI_ISL_793615, EPI_ISL_793616, EPI_ISL_793617, EPI_ISL_793618, EPI_ISL_793619, EPI_ISL_793620, EPI_ISL_793621, EPI_ISL_793622, EPI_ISL_793623, EPI_ISL_793624, EPI_ISL_793625, EPI_ISL_793626, EPI_ISL_793627, EPI_ISL_793628, EPI_ISL_793629, EPI_ISL_793630, EPI_ISL_793631, EPI_ISL_793632, EPI_ISL_793633, EPI_ISL_793634, EPI_ISL_793635, EPI_ISL_793636, EPI_ISL_793637, EPI_ISL_793638, EPI_ISL_793639, EPI_ISL_793640, EPI_ISL_793641, EPI_ISL_793642, EPI_ISL_793643, EPI_ISL_793644, EPI_ISL_793645, EPI_ISL_793646, EPI_ISL_793647, EPI_ISL_793648, EPI_ISL_793649, EPI_ISL_795011, EPI_ISL_795012, EPI_ISL_795013, EPI_ISL_795014, EPI_ISL_795015, EPI_ISL_795016, EPI_ISL_795017, EPI_ISL_795018, EPI_ISL_795019, EPI_ISL_795021, EPI_ISL_795022, EPI_ISL_795023, EPI_ISL_795024, EPI_ISL_795025, EPI_ISL_795026, EPI_ISL_795027, EPI_ISL_795028, EPI_ISL_795029, EPI_ISL_795030, EPI_ISL_795031, EPI_ISL_795032, EPI_ISL_795033, EPI_ISL_795034, EPI_ISL_795035, EPI_ISL_795036, EPI_ISL_795037, EPI_ISL_795038, EPI_ISL_795040, EPI_ISL_795041, EPI_ISL_795042, EPI_ISL_795043, EPI_ISL_795044, EPI_ISL_795045, EPI_ISL_795046, EPI_ISL_795047, EPI_ISL_795048, EPI_ISL_795049, EPI_ISL_795050, EPI_ISL_795051, EPI_ISL_795052, EPI_ISL_795053, EPI_ISL_795054, EPI_ISL_795055, EPI_ISL_795056, EPI_ISL_795057, EPI_ISL_795058, EPI_ISL_795059, EPI_ISL_795060, EPI_ISL_795061, EPI_ISL_795062, EPI_ISL_795063, EPI_ISL_795064, EPI_ISL_795065, EPI_ISL_795066, EPI_ISL_795067, EPI_ISL_795068, EPI_ISL_795069, EPI_ISL_795070, EPI_ISL_795071, EPI_ISL_795072, EPI_ISL_795073, EPI_ISL_795074, EPI_ISL_795075, EPI_ISL_795076, EPI_ISL_795077, EPI_ISL_795078, EPI_ISL_795079, EPI_ISL_795080, EPI_ISL_795081, EPI_ISL_795082, EPI_ISL_795083, EPI_ISL_795084, EPI_ISL_795085, EPI_ISL_795086, EPI_ISL_795087, EPI_ISL_795088, EPI_ISL_795089, EPI_ISL_795090, EPI_ISL_795091, EPI_ISL_795092, EPI_ISL_795093, EPI_ISL_795094, EPI_ISL_795095, EPI_ISL_795096, EPI_ISL_795097, EPI_ISL_795098, EPI_ISL_795099, EPI_ISL_795100, EPI_ISL_795101, EPI_ISL_795102, EPI_ISL_795103, EPI_ISL_795104, EPI_ISL_795105, EPI_ISL_795106, EPI_ISL_795107, EPI_ISL_795108, EPI_ISL_795109, EPI_ISL_795110, EPI_ISL_795111, EPI_ISL_795112, EPI_ISL_795113, EPI_ISL_795114, EPI_ISL_795115, EPI_ISL_795116, EPI_ISL_795117, EPI_ISL_795118, EPI_ISL_795119, EPI_ISL_795120, EPI_ISL_795121, EPI_ISL_795122, EPI_ISL_795123, EPI_ISL_795124, EPI_ISL_795125, EPI_ISL_795126, EPI_ISL_795127, EPI_ISL_795128, EPI_ISL_795129, EPI_ISL_795130, EPI_ISL_795131, EPI_ISL_795132, EPI_ISL_795133, EPI_ISL_795134, EPI_ISL_795135, EPI_ISL_795136, EPI_ISL_795137, EPI_ISL_795138, EPI_ISL_795139, EPI_ISL_795140, EPI_ISL_795141, EPI_ISL_795142, EPI_ISL_795143, EPI_ISL_795144, EPI_ISL_795145, EPI_ISL_795146, EPI_ISL_795147, EPI_ISL_795148, EPI_ISL_795154, EPI_ISL_795155, EPI_ISL_795156, EPI_ISL_795157, EPI_ISL_795158, EPI_ISL_795159, EPI_ISL_795160, EPI_ISL_795161, EPI_ISL_795162, EPI_ISL_795163, EPI_ISL_795164, EPI_ISL_795165, EPI_ISL_795166, EPI_ISL_795167, EPI_ISL_795168, EPI_ISL_795169, EPI_ISL_795171, EPI_ISL_795172, EPI_ISL_795173, EPI_ISL_795175, EPI_ISL_795176, EPI_ISL_795177, EPI_ISL_795178, EPI_ISL_795179, EPI_ISL_795180, EPI_ISL_795181, EPI_ISL_795182, EPI_ISL_795183, EPI_ISL_795184, EPI_ISL_795185, EPI_ISL_795186, EPI_ISL_795187, EPI_ISL_795188, EPI_ISL_795189, EPI_ISL_795190, EPI_ISL_795191, EPI_ISL_795192, EPI_ISL_795193, EPI_ISL_795194, EPI_ISL_795195, EPI_ISL_795196, EPI_ISL_795197, EPI_ISL_795198, EPI_ISL_795199, EPI_ISL_795200, EPI_ISL_795201, EPI_ISL_795202, EPI_ISL_795203, EPI_ISL_795204, EPI_ISL_795205, EPI_ISL_795206, EPI_ISL_795207, EPI_ISL_795208, EPI_ISL_795209, EPI_ISL_795210, EPI_ISL_795211, EPI_ISL_795212, EPI_ISL_795213, EPI_ISL_795214, EPI_ISL_795215, EPI_ISL_795216, EPI_ISL_795217, EPI_ISL_795218, EPI_ISL_795219, EPI_ISL_795220, EPI_ISL_795221, EPI_ISL_795222, EPI_ISL_795223, EPI_ISL_795224, EPI_ISL_795225, EPI_ISL_795226, EPI_ISL_795227, EPI_ISL_795228, EPI_ISL_795229, EPI_ISL_795230, EPI_ISL_795231, EPI_ISL_795232, EPI_ISL_795233, EPI_ISL_795234, EPI_ISL_795235, EPI_ISL_795236, EPI_ISL_795237, EPI_ISL_795238, EPI_ISL_795242, EPI_ISL_795244, EPI_ISL_795245, EPI_ISL_795246, EPI_ISL_795247, EPI_ISL_795248, EPI_ISL_795249, EPI_ISL_795250, EPI_ISL_795252, EPI_ISL_795253, EPI_ISL_795254, EPI_ISL_795255, EPI_ISL_795256, EPI_ISL_795257, EPI_ISL_795258, EPI_ISL_795259, EPI_ISL_795260, EPI_ISL_795261, EPI_ISL_795262, EPI_ISL_795263, EPI_ISL_795264, EPI_ISL_795265, EPI_ISL_795266, EPI_ISL_795267, EPI_ISL_795268, EPI_ISL_795269, EPI_ISL_795270, EPI_ISL_795271, EPI_ISL_795272, EPI_ISL_795273, EPI_ISL_795274, EPI_ISL_795275, EPI_ISL_795276, EPI_ISL_795277, EPI_ISL_795281, EPI_ISL_795282, EPI_ISL_795283, EPI_ISL_795284, EPI_ISL_795285, EPI_ISL_795286, EPI_ISL_795287, EPI_ISL_795288, EPI_ISL_795289, EPI_ISL_795290, EPI_ISL_795291, EPI_ISL_795292, EPI_ISL_795293, EPI_ISL_795294, EPI_ISL_795295, EPI_ISL_795296, EPI_ISL_795297, EPI_ISL_795298, EPI_ISL_795299, EPI_ISL_795300, EPI_ISL_795301, EPI_ISL_795302, EPI_ISL_795303, EPI_ISL_795304, EPI_ISL_795305, EPI_ISL_795306, EPI_ISL_795307, EPI_ISL_795308, EPI_ISL_795309, EPI_ISL_795310, EPI_ISL_795311, EPI_ISL_795312, EPI_ISL_795313, EPI_ISL_795314, EPI_ISL_795315, EPI_ISL_795316, EPI_ISL_795317, EPI_ISL_795318, EPI_ISL_795319, EPI_ISL_795320, EPI_ISL_795321, EPI_ISL_795322, EPI_ISL_795323, EPI_ISL_795324, EPI_ISL_795325, EPI_ISL_795326, EPI_ISL_795327, EPI_ISL_795328, EPI_ISL_795329, EPI_ISL_795330, EPI_ISL_795331, EPI_ISL_795332, EPI_ISL_795333, EPI_ISL_795334, EPI_ISL_795335, EPI_ISL_795336, EPI_ISL_795337, EPI_ISL_795338, EPI_ISL_795339, EPI_ISL_795340, EPI_ISL_795341, EPI_ISL_795342, EPI_ISL_795343, EPI_ISL_795344, EPI_ISL_795345, EPI_ISL_795346, EPI_ISL_795347, EPI_ISL_795348, EPI_ISL_795349, EPI_ISL_795350, EPI_ISL_795351, EPI_ISL_795352, EPI_ISL_795353, EPI_ISL_795354, EPI_ISL_795355, EPI_ISL_795356, EPI_ISL_795357, EPI_ISL_795358, EPI_ISL_795359, EPI_ISL_795360, EPI_ISL_795361, EPI_ISL_795368, EPI_ISL_795369, EPI_ISL_795370, EPI_ISL_795371, EPI_ISL_795372, EPI_ISL_795373, EPI_ISL_795374, EPI_ISL_795375, EPI_ISL_795376, EPI_ISL_795377, EPI_ISL_795378, EPI_ISL_795379, EPI_ISL_795380, EPI_ISL_795381, EPI_ISL_795382, EPI_ISL_795384, EPI_ISL_795385, EPI_ISL_795386, EPI_ISL_795387, EPI_ISL_795388, EPI_ISL_795389, EPI_ISL_795390, EPI_ISL_795391, EPI_ISL_795392, EPI_ISL_795393, EPI_ISL_795394, EPI_ISL_795395, EPI_ISL_795396, EPI_ISL_795397, EPI_ISL_795398, EPI_ISL_795399, EPI_ISL_795400, EPI_ISL_795401, EPI_ISL_795402, EPI_ISL_795403, EPI_ISL_795404, EPI_ISL_795405, EPI_ISL_795406, EPI_ISL_795407, EPI_ISL_795408, EPI_ISL_795409, EPI_ISL_795410, EPI_ISL_795411, EPI_ISL_795412, EPI_ISL_795414, EPI_ISL_795415, EPI_ISL_795416, EPI_ISL_795417, EPI_ISL_795418, EPI_ISL_795419, EPI_ISL_795420, EPI_ISL_795421, EPI_ISL_795422, EPI_ISL_795423, EPI_ISL_795424, EPI_ISL_795426, EPI_ISL_795427, EPI_ISL_795428, EPI_ISL_795429, EPI_ISL_795430, EPI_ISL_795431, EPI_ISL_795432, EPI_ISL_795433, EPI_ISL_795434, EPI_ISL_795435,

We gratefully acknowledge the following Authors from the Originating laboratories responsible for obtaining the specimens, as well as the Submitting laboratories where the genome data were generated and shared via GISAID, on which this research is based.

All Submitters of data may be contacted directly via www.gisaid.org

Authors are sorted alphabetically.

Accession ID	Originating Laboratory	Submitting Laboratory	Authors

EPI_ISL_1024983, EPI_ISL_1024984, EPI_ISL_1024985, EPI_ISL_1024986, EPI_ISL_1024987, EPI_ISL_1024988, EPI_ISL_1024989, EPI_ISL_1024990, EPI_ISL_1024991, EPI_ISL_1024992, EPI_ISL_1024993, EPI_ISL_1024994, EPI_ISL_1024995, EPI_ISL_1024996, EPI_ISL_1024997, EPI_ISL_1024998, EPI_ISL_1024999, EPI_ISL_1025000, EPI_ISL_1025001, EPI_ISL_1025002, EPI_ISL_1025003, EPI_ISL_1025004, EPI_ISL_1025005, EPI_ISL_1025006, EPI_ISL_1025007, EPI_ISL_1025008, EPI_ISL_1025009, EPI_ISL_1025010, EPI_ISL_1025011, EPI_ISL_1025012, EPI_ISL_1025013, EPI_ISL_1025014, EPI_ISL_1025015, EPI_ISL_1025016, EPI_ISL_1025017, EPI_ISL_1025018, EPI_ISL_1025019, EPI_ISL_1025020, EPI_ISL_1025021, EPI_ISL_1025022, EPI_ISL_1025023, EPI_ISL_1025024, EPI_ISL_1025025, EPI_ISL_1025026, EPI_ISL_1025027, EPI_ISL_1025028, EPI_ISL_1025029, EPI_ISL_1025030, EPI_ISL_1025031, EPI_ISL_1025032, EPI_ISL_1025033, EPI_ISL_1025034, EPI_ISL_1025035, EPI_ISL_1025036, EPI_ISL_1025037, EPI_ISL_1025038, EPI_ISL_1025039, EPI_ISL_1025040, EPI_ISL_1025041, EPI_ISL_1025042, EPI_ISL_1025043, EPI_ISL_1025044, EPI_ISL_1025045, EPI_ISL_1025046, EPI_ISL_1025047, EPI_ISL_1025048, EPI_ISL_1025049, EPI_ISL_1025050, EPI_ISL_1025051, EPI_ISL_1025052, EPI_ISL_1025053, EPI_ISL_1025054, EPI_ISL_1025055, EPI_ISL_1025056, EPI_ISL_1025057, EPI_ISL_1025058, EPI_ISL_1025059, EPI_ISL_1025060, EPI_ISL_1025061, EPI_ISL_1025062, EPI_ISL_1025063, EPI_ISL_1025064, EPI_ISL_1025065, EPI_ISL_1025066, EPI_ISL_1025067, EPI_ISL_1025068, EPI_ISL_1025069, EPI_ISL_1025070, EPI_ISL_1025071, EPI_ISL_1025072, EPI_ISL_1025073, EPI_ISL_1025074, EPI_ISL_1025075, EPI_ISL_1025076, EPI_ISL_1025077, EPI_ISL_1025078, EPI_ISL_1025079, EPI_ISL_1025080, EPI_ISL_1025081, EPI_ISL_1025082, EPI_ISL_1025083, EPI_ISL_1025084, EPI_ISL_1025085, EPI_ISL_1025086, EPI_ISL_1025087, EPI_ISL_1025088, EPI_ISL_1025089, EPI_ISL_1025090, EPI_ISL_1025091, EPI_ISL_1025092, EPI_ISL_1025093, EPI_ISL_1025094, EPI_ISL_1025095, EPI_ISL_1025096, EPI_ISL_1025097, EPI_ISL_1025098, EPI_ISL_1025099, EPI_ISL_1025100, EPI_ISL_1025101, EPI_ISL_1025102, EPI_ISL_1025103, EPI_ISL_1025104, EPI_ISL_1025105, EPI_ISL_1025106, EPI_ISL_1025107, EPI_ISL_1025108, EPI_ISL_1025109, EPI_ISL_1025110, EPI_ISL_1025111, EPI_ISL_1025112, EPI_ISL_1025113, EPI_ISL_1025114, EPI_ISL_1025115, EPI_ISL_1025116, EPI_ISL_1025117, EPI_ISL_1025118, EPI_ISL_1025119, EPI_ISL_1025120, EPI_ISL_1025121, EPI_ISL_1025122, EPI_ISL_1025123, EPI_ISL_1025124, EPI_ISL_1025125, EPI_ISL_1025126, EPI_ISL_1025127, EPI_ISL_1025128, EPI_ISL_1025129, EPI_ISL_1025130, EPI_ISL_1025131, EPI_ISL_1025132, EPI_ISL_1025133, EPI_ISL_1025134, EPI_ISL_1025135, EPI_ISL_1025136, EPI_ISL_1025137, EPI_ISL_1025138, EPI_ISL_1025139, EPI_ISL_1025140, EPI_ISL_1025141, EPI_ISL_1025142, EPI_ISL_1025143, EPI_ISL_1025144, EPI_ISL_1025145, EPI_ISL_1025146, EPI_ISL_1025147, EPI_ISL_1025148, EPI_ISL_1025149, EPI_ISL_1025150, EPI_ISL_1025151, EPI_ISL_1025152, EPI_ISL_1025153, EPI_ISL_1025154, EPI_ISL_1025155, EPI_ISL_1025156, EPI_ISL_1025157, EPI_ISL_1025158, EPI_ISL_1025159, EPI_ISL_1025160, EPI_ISL_1025161, EPI_ISL_1025162, EPI_ISL_1025163, EPI_ISL_1025164, EPI_ISL_1025165, EPI_ISL_1025166, EPI_ISL_1025167, EPI_ISL_1025168, EPI_ISL_1025169, EPI_ISL_1025170, EPI_ISL_1025171, EPI_ISL_1025172, EPI_ISL_1025173, EPI_ISL_1025174, EPI_ISL_1025175, EPI_ISL_1025176, EPI_ISL_1025177, EPI_ISL_1025178, EPI_ISL_1025179, EPI_ISL_1025180, EPI_ISL_1025181, EPI_ISL_1025182, EPI_ISL_1025183, EPI_ISL_1025184, EPI_ISL_1025185, EPI_ISL_1025186, EPI_ISL_1025187, EPI_ISL_1025188, EPI_ISL_1025189, EPI_ISL_1025190, EPI_ISL_1025191, EPI_ISL_1025192, EPI_ISL_1025193, EPI_ISL_1025194, EPI_ISL_1025195, EPI_ISL_1025196, EPI_ISL_1025197, EPI_ISL_1025198, EPI_ISL_1025199, EPI_ISL_1025200, EPI_ISL_1025201, EPI_ISL_1025202, EPI_ISL_1025203, EPI_ISL_1025204, EPI_ISL_1025205, EPI_ISL_1025206, EPI_ISL_1025207, EPI_ISL_1025208, EPI_ISL_1025209, EPI_ISL_1025210, EPI_ISL_1025211, EPI_ISL_1025212, EPI_ISL_1025213, EPI_ISL_1025214, EPI_ISL_1025215, EPI_ISL_1025216, EPI_ISL_1025217, EPI_ISL_1025218, EPI_ISL_1025219, EPI_ISL_1025220, EPI_ISL_1025221, EPI_ISL_1025222, EPI_ISL_1025223, EPI_ISL_1025224, EPI_ISL_1025225, EPI_ISL_1025226, EPI_ISL_1025227, EPI_ISL_1025228, EPI_ISL_1025229, EPI_ISL_1025230, EPI_ISL_1025231, EPI_ISL_1025232, EPI_ISL_1025233, EPI_ISL_1025234, EPI_ISL_1025235, EPI_ISL_1025236, EPI_ISL_1025237, EPI_ISL_1025238, EPI_ISL_1025239, EPI_ISL_1025240, EPI_ISL_1025241, EPI_ISL_1025242, EPI_ISL_1025243, EPI_ISL_1025244, EPI_ISL_1025245, EPI_ISL_1025246, EPI_ISL_1025247, EPI_ISL_1025248, EPI_ISL_1025249, EPI_ISL_1025250, EPI_ISL_1025251, EPI_ISL_1025252, EPI_ISL_1025253, EPI_ISL_1025254, EPI_ISL_1025255, EPI_ISL_1025256, EPI_ISL_1025257, EPI_ISL_1025258, EPI_ISL_1025259, EPI_ISL_1025260, EPI_ISL_1025261, EPI_ISL_1025262, EPI_ISL_1025263, EPI_ISL_1025264, EPI_ISL_1025265, EPI_ISL_1025266, EPI_ISL_1025267, EPI_ISL_1025268, EPI_ISL_1025269, EPI_ISL_1025270, EPI_ISL_1025271, EPI_ISL_1025272, EPI_ISL_1025273, EPI_ISL_1025274, EPI_ISL_1025275, EPI_ISL_1025276, EPI_ISL_1025277, EPI_ISL_1025278, EPI_ISL_1025279, EPI_ISL_1025280, EPI_ISL_1025281, EPI_ISL_1025282, EPI_ISL_1025283, EPI_ISL_1025284, EPI_ISL_1025285, EPI_ISL_1025286, EPI_ISL_1025287, EPI_ISL_1025288, EPI_ISL_1025289, EPI_ISL_1025290, EPI_ISL_1025291, EPI_ISL_1025292, EPI_ISL_1025293, EPI_ISL_1025294, EPI_ISL_1025295, EPI_ISL_1025296, EPI_ISL_1025297, EPI_ISL_1025298, EPI_ISL_1025299, EPI_ISL_1025300, EPI_ISL_1025301, EPI_ISL_1025302, EPI_ISL_1025303, EPI_ISL_1025304, EPI_ISL_1025305, EPI_ISL_1025306, EPI_ISL_1025307, EPI_ISL_1025308, EPI_ISL_1025309, EPI_ISL_1025310, EPI_ISL_1025311, EPI_ISL_1025312, EPI_ISL_1025313, EPI_ISL_1025314, EPI_ISL_1025315, EPI_ISL_1025316, EPI_ISL_1025317, EPI_ISL_1025318, EPI_ISL_1025319, EPI_ISL_1025320, EPI_ISL_1025321, EPI_ISL_1025322, EPI_ISL_1025323, EPI_ISL_1025324, EPI_ISL_1025325, EPI_ISL_1025326, EPI_ISL_1025327, EPI_ISL_1025328, EPI_ISL_1025329, EPI_ISL_1025330, EPI_ISL_1025331, EPI_ISL_1025332, EPI_ISL_1025333, EPI_ISL_1025334, EPI_ISL_1025335, EPI_ISL_1025336, EPI_ISL_1025337, EPI_ISL_1025338, EPI_ISL_1025339, EPI_ISL_1025340, EPI_ISL_1025341, EPI_ISL_1025342, EPI_ISL_1025343, EPI_ISL_1025344, EPI_ISL_1065095, EPI_ISL_1065113, EPI_ISL_1065127, EPI_ISL_1065135, EPI_ISL_1065137, EPI_ISL_1065143, EPI_ISL_1065161, EPI_ISL_1065170, EPI_ISL_1065172, EPI_ISL_1065185, EPI_ISL_1065227, EPI_ISL_1065237, EPI_ISL_1065238, EPI_ISL_1065241, EPI_ISL_1065268, EPI_ISL_1065269, EPI_ISL_1065274, EPI_ISL_1065276, EPI_ISL_1065283, EPI_ISL_1065292, EPI_ISL_1065299, EPI_ISL_1065303, EPI_ISL_1065305, EPI_ISL_1065316, EPI_ISL_1065320, EPI_ISL_1065321, EPI_ISL_1065333, EPI_ISL_1065338, EPI_ISL_1065408, EPI_ISL_1065410, EPI_ISL_1065457, EPI_ISL_1065472, EPI_ISL_1065480, EPI_ISL_1065498, EPI_ISL_1065499, EPI_ISL_1065511, EPI_ISL_1065513, EPI_ISL_1065518, EPI_ISL_1065524, EPI_ISL_1065525, EPI_ISL_1065526, EPI_ISL_1065536, EPI_ISL_1065563, EPI_ISL_1065565, EPI_ISL_1065570, EPI_ISL_1065588, EPI_ISL_1065596, EPI_ISL_1065628, EPI_ISL_1065637, EPI_ISL_1065645, EPI_ISL_1065654, EPI_ISL_1065663, EPI_ISL_1065688, EPI_ISL_1065703, EPI_ISL_1065734, EPI_ISL_1065743, EPI_ISL_1065767, EPI_ISL_1065802, EPI_ISL_1065808, EPI_ISL_1065811, EPI_ISL_1065859, EPI_ISL_1065871, EPI_ISL_1065875, EPI_ISL_1065885, EPI_ISL_1065888, EPI_ISL_1065898, EPI_ISL_1065908, EPI_ISL_1065937, EPI_ISL_1065950, EPI_ISL_1065957, EPI_ISL_1065960, EPI_ISL_1065974, EPI_ISL_1066002, EPI_ISL_1066009, EPI_ISL_1066010, EPI_ISL_1066012, EPI_ISL_1066019, EPI_ISL_1066025, EPI_ISL_1066040, EPI_ISL_1066048, EPI_ISL_1066066, EPI_ISL_1066087, EPI_ISL_1066099, EPI_ISL_1066127, EPI_ISL_1066129, EPI_ISL_1066140, EPI_ISL_1066141, EPI_ISL_1066158, EPI_ISL_1066159, EPI_ISL_1066183, EPI_ISL_1066184, EPI_ISL_1066188, EPI_ISL_1066191, EPI_ISL_1066192, EPI_ISL_1066194, EPI_ISL_1066195, EPI_ISL_1066199, EPI_ISL_1066205, EPI_ISL_1066208, EPI_ISL_1066232, EPI_ISL_1066233, EPI_ISL_1066245, EPI_ISL_1066285, EPI_ISL_1066304, EPI_ISL_1066324, EPI_ISL_1066339, EPI_ISL_1066346, EPI_ISL_1066359, EPI_ISL_1066370, EPI_ISL_1066371, EPI_ISL_1066374, EPI_ISL_1066375, EPI_ISL_1066403, EPI_ISL_1066412, EPI_ISL_1066415, EPI_ISL_1066432, EPI_ISL_1066437, EPI_ISL_1066444, EPI_ISL_1066494, EPI_ISL_1066496, EPI_ISL_1066506, EPI_ISL_1066508, EPI_ISL_1066515, EPI_ISL_1066516, EPI_ISL_1066545, EPI_ISL_1066572, EPI_ISL_1066576, EPI_ISL_1066591, EPI_ISL_1066606, EPI_ISL_1066610, EPI_ISL_1066630, EPI_ISL_1066659, EPI_ISL_1066666, EPI_ISL_1066685, EPI_ISL_1066697, EPI_ISL_1066698, EPI_ISL_1066708, EPI_ISL_1066710, EPI_ISL_1066726, EPI_ISL_1066727, EPI_ISL_1066753, EPI_ISL_1066754, EPI_ISL_1066757, EPI_ISL_1066761, EPI_ISL_1066812, EPI_ISL_1066813, EPI_ISL_1066814, EPI_ISL_1066832, EPI_ISL_1066866, EPI_ISL_1066867, EPI_ISL_1066875, EPI_ISL_1066876, EPI_ISL_1066909, EPI_ISL_1066938, EPI_ISL_1066944, EPI_ISL_1066954, EPI_ISL_1066988, EPI_ISL_1066997, EPI_ISL_1067011, EPI_ISL_1067012, EPI_ISL_1067037, EPI_ISL_1067048, EPI_ISL_1067074, EPI_ISL_1067092, EPI_ISL_1067098, EPI_ISL_1067105, EPI_ISL_1067112, EPI_ISL_1067166, EPI_ISL_1067203, EPI_ISL_1067214, EPI_ISL_1067238, EPI_ISL_1067242, EPI_ISL_1067254, EPI_ISL_1067259, EPI_ISL_1067303, EPI_ISL_1067306, EPI_ISL_1067353, EPI_ISL_1067361, EPI_ISL_1067362, EPI_ISL_1067376, EPI_ISL_1067387, EPI_ISL_1067389, EPI_ISL_1067393, EPI_ISL_1067413, EPI_ISL_1067422, EPI_ISL_1067436, EPI_ISL_1067452, EPI_ISL_1067466, EPI_ISL_1067468, EPI_ISL_1067482, EPI_ISL_1067488, EPI_ISL_1067520, EPI_ISL_1067539

see above	Department of Virus and Microbiological Special Diagnostics, Statens Serum Institut, Copenhagen, Denmark	Aalborg University	Danish Covid-19 Genome Consortium

EPI_ISL_844543, EPI_ISL_844545, EPI_ISL_844552, EPI_ISL_844553, EPI_ISL_844556, EPI_ISL_844557, EPI_ISL_844558, EPI_ISL_844559, EPI_ISL_844560, EPI_ISL_844561, EPI_ISL_844562, EPI_ISL_844563, EPI_ISL_844564, EPI_ISL_844565, EPI_ISL_844566, EPI_ISL_844567, EPI_ISL_844568, EPI_ISL_844569, EPI_ISL_844570, EPI_ISL_844571, EPI_ISL_844572, EPI_ISL_844573, EPI_ISL_844574, EPI_ISL_844575, EPI_ISL_844576, EPI_ISL_844577, EPI_ISL_844635, EPI_ISL_844636, EPI_ISL_844637, EPI_ISL_844638, EPI_ISL_844639, EPI_ISL_844653, EPI_ISL_844654, EPI_ISL_844655, EPI_ISL_844666, EPI_ISL_844667, EPI_ISL_844669, EPI_ISL_844670, EPI_ISL_844676, EPI_ISL_844680, EPI_ISL_844681, EPI_ISL_844693, EPI_ISL_844694, EPI_ISL_844702, EPI_ISL_844706, EPI_ISL_844707, EPI_ISL_844708, EPI_ISL_844716, EPI_ISL_844717, EPI_ISL_844718, EPI_ISL_844730, EPI_ISL_844731, EPI_ISL_844732, EPI_ISL_844733, EPI_ISL_844734, EPI_ISL_844735, EPI_ISL_844736, EPI_ISL_844737, EPI_ISL_844738, EPI_ISL_844747, EPI_ISL_844758, EPI_ISL_844759, EPI_ISL_844763, EPI_ISL_844766, EPI_ISL_844767, EPI_ISL_844768, EPI_ISL_844788, EPI_ISL_844805, EPI_ISL_844807, EPI_ISL_844808, EPI_ISL_844818, EPI_ISL_844821, EPI_ISL_844824, EPI_ISL_844834, EPI_ISL_844836, EPI_ISL_844837, EPI_ISL_844842, EPI_ISL_844843, EPI_ISL_844844, EPI_ISL_844845, EPI_ISL_844846, EPI_ISL_844847, EPI_ISL_844848, EPI_ISL_844849, EPI_ISL_844860, EPI_ISL_844861, EPI_ISL_844862, EPI_ISL_844863, EPI_ISL_844864, EPI_ISL_844865, EPI_ISL_844911, EPI_ISL_844912, EPI_ISL_844931, EPI_ISL_844932, EPI_ISL_844948, EPI_ISL_844951, EPI_ISL_844953, EPI_ISL_844954, EPI_ISL_844955, EPI_ISL_844956, EPI_ISL_844957, EPI_ISL_844958, EPI_ISL_844978, EPI_ISL_844981, EPI_ISL_844988, EPI_ISL_844991, EPI_ISL_844992, EPI_ISL_844993, EPI_ISL_844998, EPI_ISL_844999, EPI_ISL_845000, EPI_ISL_845001, EPI_ISL_845002, EPI_ISL_845003, EPI_ISL_845004, EPI_ISL_845005, EPI_ISL_845006, EPI_ISL_845007, EPI_ISL_845008, EPI_ISL_845009, EPI_ISL_845010, EPI_ISL_845011, EPI_ISL_845012, EPI_ISL_845013, EPI_ISL_845014, EPI_ISL_845015, EPI_ISL_845016, EPI_ISL_845017, EPI_ISL_845018, EPI_ISL_845019, EPI_ISL_845020, EPI_ISL_845021, EPI_ISL_845022, EPI_ISL_845023, EPI_ISL_845024, EPI_ISL_845025, EPI_ISL_845026, EPI_ISL_845027, EPI_ISL_845028, EPI_ISL_845029, EPI_ISL_845030, EPI_ISL_845031, EPI_ISL_845032, EPI_ISL_845033, EPI_ISL_845034, EPI_ISL_845035, EPI_ISL_845036, EPI_ISL_845037, EPI_ISL_845038, EPI_ISL_845039, EPI_ISL_845040, EPI_ISL_845041, EPI_ISL_845042, EPI_ISL_845043, EPI_ISL_845044, EPI_ISL_845045, EPI_ISL_845046, EPI_ISL_845047, EPI_ISL_845048, EPI_ISL_845049, EPI_ISL_845050, EPI_ISL_845051, EPI_ISL_845052, EPI_ISL_845053, EPI_ISL_845054, EPI_ISL_845055, EPI_ISL_845056, EPI_ISL_845057, EPI_ISL_845058, EPI_ISL_845059, EPI_ISL_845060, EPI_ISL_845061, EPI_ISL_845062, EPI_ISL_845063, EPI_ISL_845064, EPI_ISL_845065, EPI_ISL_845066, EPI_ISL_845067, EPI_ISL_845068, EPI_ISL_845069, EPI_ISL_845070, EPI_ISL_845071, EPI_ISL_845072, EPI_ISL_845073, EPI_ISL_845074, EPI_ISL_845075, EPI_ISL_845076, EPI_ISL_845077, EPI_ISL_845078, EPI_ISL_845079, EPI_ISL_845080, EPI_ISL_845275, EPI_ISL_845276, EPI_ISL_845277, EPI_ISL_845278, EPI_ISL_845279, EPI_ISL_845280, EPI_ISL_845377, EPI_ISL_845378, EPI_ISL_845379, EPI_ISL_845380, EPI_ISL_845381, EPI_ISL_845382, EPI_ISL_845383, EPI_ISL_845384, EPI_ISL_845385, EPI_ISL_845386, EPI_ISL_845387, EPI_ISL_845388, EPI_ISL_845389, EPI_ISL_845390, EPI_ISL_845391, EPI_ISL_845392, EPI_ISL_845393, EPI_ISL_845394, EPI_ISL_845395, EPI_ISL_845396, EPI_ISL_845397, EPI_ISL_845398, EPI_ISL_845399, EPI_ISL_845498, EPI_ISL_845499, EPI_ISL_845500, EPI_ISL_846775, EPI_ISL_846776, EPI_ISL_846777, EPI_ISL_846778, EPI_ISL_846780, EPI_ISL_846782, EPI_ISL_846783, EPI_ISL_846784, EPI_ISL_846785, EPI_ISL_846786, EPI_ISL_846787, EPI_ISL_846788, EPI_ISL_846789, EPI_ISL_846790, EPI_ISL_846791, EPI_ISL_846792, EPI_ISL_846793, EPI_ISL_846794, EPI_ISL_846795, EPI_ISL_846796, EPI_ISL_846797, EPI_ISL_846798, EPI_ISL_846799, EPI_ISL_846800, EPI_ISL_846801, EPI_ISL_846802, EPI_ISL_846803, EPI_ISL_846804, EPI_ISL_846805, EPI_ISL_846806, EPI_ISL_846807, EPI_ISL_846808, EPI_ISL_846809, EPI_ISL_846810, EPI_ISL_846811, EPI_ISL_846821, EPI_ISL_846822, EPI_ISL_846823, EPI_ISL_846824, EPI_ISL_846825, EPI_ISL_846826, EPI_ISL_846827, EPI_ISL_846828, EPI_ISL_846829, EPI_ISL_846830, EPI_ISL_846831, EPI_ISL_846832, EPI_ISL_846833, EPI_ISL_846834, EPI_ISL_846835, EPI_ISL_846836, EPI_ISL_846837, EPI_ISL_846838, EPI_ISL_846839, EPI_ISL_846840, EPI_ISL_846841, EPI_ISL_846842, EPI_ISL_846843, EPI_ISL_846844, EPI_ISL_846845, EPI_ISL_846846, EPI_ISL_846847, EPI_ISL_846848, EPI_ISL_846849, EPI_ISL_846850, EPI_ISL_846854, EPI_ISL_846855, EPI_ISL_846856, EPI_ISL_846857, EPI_ISL_846858, EPI_ISL_846859, EPI_ISL_846862, EPI_ISL_846863, EPI_ISL_846864, EPI_ISL_846865, EPI_ISL_846867, EPI_ISL_846868, EPI_ISL_846869, EPI_ISL_846870, EPI_ISL_846871, EPI_ISL_846872, EPI_ISL_846873, EPI_ISL_846874, EPI_ISL_846875, EPI_ISL_846876, EPI_ISL_846878, EPI_ISL_846879, EPI_ISL_846880, EPI_ISL_846881, EPI_ISL_846882, EPI_ISL_846883, EPI_ISL_846885, EPI_ISL_846887, EPI_ISL_846890, EPI_ISL_846891, EPI_ISL_846892, EPI_ISL_846896, EPI_ISL_846897, EPI_ISL_846899, EPI_ISL_846900, EPI_ISL_846901, EPI_ISL_846902, EPI_ISL_846903, EPI_ISL_846905, EPI_ISL_846906, EPI_ISL_846907, EPI_ISL_846908, EPI_ISL_846909, EPI_ISL_846911, EPI_ISL_846912, EPI_ISL_846914, EPI_ISL_846915, EPI_ISL_846916, EPI_ISL_846917, EPI_ISL_846918, EPI_ISL_846919, EPI_ISL_846920, EPI_ISL_846921, EPI_ISL_846922, EPI_ISL_846923, EPI_ISL_846924, EPI_ISL_846925, EPI_ISL_846926, EPI_ISL_846927, EPI_ISL_846928, EPI_ISL_846929, EPI_ISL_846930, EPI_ISL_846931, EPI_ISL_846932, EPI_ISL_846933, EPI_ISL_846934, EPI_ISL_846935, EPI_ISL_846936, EPI_ISL_846937, EPI_ISL_846938, EPI_ISL_846939, EPI_ISL_846940, EPI_ISL_846941, EPI_ISL_846942, EPI_ISL_846943, EPI_ISL_846944, EPI_ISL_846945, EPI_ISL_846946, EPI_ISL_846947, EPI_ISL_846948, EPI_ISL_846949, EPI_ISL_846950, EPI_ISL_846951, EPI_ISL_846952, EPI_ISL_846953, EPI_ISL_846954, EPI_ISL_846955, EPI_ISL_846956, EPI_ISL_846957, EPI_ISL_846958, EPI_ISL_846959, EPI_ISL_846960, EPI_ISL_846961, EPI_ISL_846962, EPI_ISL_846963, EPI_ISL_846964, EPI_ISL_846965, EPI_ISL_846966, EPI_ISL_846967, EPI_ISL_846968, EPI_ISL_846969, EPI_ISL_846970, EPI_ISL_846971, EPI_ISL_846972, EPI_ISL_846973, EPI_ISL_846974, EPI_ISL_846975, EPI_ISL_846976, EPI_ISL_846977, EPI_ISL_846978, EPI_ISL_846979, EPI_ISL_846980, EPI_ISL_846981, EPI_ISL_846982, EPI_ISL_846983, EPI_ISL_846984, EPI_ISL_846985, EPI_ISL_846986, EPI_ISL_846987, EPI_ISL_846988, EPI_ISL_846989, EPI_ISL_846990, EPI_ISL_846991, EPI_ISL_846992, EPI_ISL_846993, EPI_ISL_846994, EPI_ISL_846995, EPI_ISL_846996, EPI_ISL_846997, EPI_ISL_846998, EPI_ISL_846999, EPI_ISL_847000, EPI_ISL_847001, EPI_ISL_847002, EPI_ISL_847003, EPI_ISL_847004, EPI_ISL_847005, EPI_ISL_847006, EPI_ISL_847007, EPI_ISL_847008, EPI_ISL_847009, EPI_ISL_847010, EPI_ISL_847011, EPI_ISL_847012, EPI_ISL_847013, EPI_ISL_847014, EPI_ISL_847015, EPI_ISL_847016, EPI_ISL_847017, EPI_ISL_847018, EPI_ISL_847019, EPI_ISL_847020, EPI_ISL_847021, EPI_ISL_847022, EPI_ISL_847023, EPI_ISL_847024, EPI_ISL_847025, EPI_ISL_847026, EPI_ISL_847027, EPI_ISL_847028, EPI_ISL_847029, EPI_ISL_847030, EPI_ISL_847031, EPI_ISL_847032, EPI_ISL_847033, EPI_ISL_847034, EPI_ISL_847035, EPI_ISL_847036, EPI_ISL_847037, EPI_ISL_847038, EPI_ISL_847039, EPI_ISL_847040, EPI_ISL_847041, EPI_ISL_847042, EPI_ISL_847043, EPI_ISL_847044, EPI_ISL_847045, EPI_ISL_847046, EPI_ISL_847047, EPI_ISL_847048, EPI_ISL_847049, EPI_ISL_847050, EPI_ISL_847051, EPI_ISL_847052, EPI_ISL_847053, EPI_ISL_847054, EPI_ISL_847055, EPI_ISL_847056, EPI_ISL_847057, EPI_ISL_847058, EPI_ISL_847059, EPI_ISL_847060, EPI_ISL_847061, EPI_ISL_847062, EPI_ISL_847063, EPI_ISL_847064, EPI_ISL_847065, EPI_ISL_847066, EPI_ISL_847067, EPI_ISL_847068, EPI_ISL_847069, EPI_ISL_847070, EPI_ISL_847071, EPI_ISL_847072, EPI_ISL_847073, EPI_ISL_847074, EPI_ISL_847075, EPI_ISL_847076, EPI_ISL_847077, EPI_ISL_847078, EPI_ISL_847079, EPI_ISL_847080, EPI_ISL_847081, EPI_ISL_847082, EPI_ISL_847083, EPI_ISL_847084, EPI_ISL_847085, EPI_ISL_847086, EPI_ISL_847087, EPI_ISL_847088, EPI_ISL_847089, EPI_ISL_847090, EPI_ISL_847091, EPI_ISL_847092, EPI_ISL_847093, EPI_ISL_847094, EPI_ISL_847095, EPI_ISL_847096, EPI_ISL_847097, EPI_ISL_847098, EPI_ISL_847099, EPI_ISL_847100, EPI_ISL_847101, EPI_ISL_847102, EPI_ISL_847103, EPI_ISL_847104, EPI_ISL_847105, EPI_ISL_847106, EPI_ISL_847107, EPI_ISL_847108, EPI_ISL_847109, EPI_ISL_847110, EPI_ISL_847111, EPI_ISL_847112, EPI_ISL_847113, EPI_ISL_847114, EPI_ISL_847115, EPI_ISL_847116, EPI_ISL_847117, EPI_ISL_847118, EPI_ISL_847119, EPI_ISL_847120, EPI_ISL_847121, EPI_ISL_847122, EPI_ISL_847123, EPI_ISL_847124, EPI_ISL_847180, EPI_ISL_847181, EPI_ISL_847182, EPI_ISL_847183, EPI_ISL_847184, EPI_ISL_847185, EPI_ISL_847186, EPI_ISL_847187, EPI_ISL_847188, EPI_ISL_847189, EPI_ISL_847190, EPI_ISL_847191, EPI_ISL_847192, EPI_ISL_847193, EPI_ISL_847194, EPI_ISL_847195, EPI_ISL_847196, EPI_ISL_847197, EPI_ISL_847198, EPI_ISL_847206, EPI_ISL_847207, EPI_ISL_847208, EPI_ISL_847209, EPI_ISL_847210, EPI_ISL_847211, EPI_ISL_847212, EPI_ISL_847213, EPI_ISL_847214, EPI_ISL_847215, EPI_ISL_847216, EPI_ISL_847217, EPI_ISL_847218, EPI_ISL_847219, EPI_ISL_847220, EPI_ISL_847221, EPI_ISL_847222, EPI_ISL_847223, EPI_ISL_847224, EPI_ISL_847225, EPI_ISL_847226, EPI_ISL_847227, EPI_ISL_847228, EPI_ISL_847229, EPI_ISL_847230, EPI_ISL_847231, EPI_ISL_847232, EPI_ISL_847238, EPI_ISL_847239, EPI_ISL_847240, EPI_ISL_847241, EPI_ISL_847242, EPI_ISL_847243, EPI_ISL_847250, EPI_ISL_847251, EPI_ISL_847257, EPI_ISL_847258, EPI_ISL_847259, EPI_ISL_847261, EPI_ISL_847265, EPI_ISL_847267, EPI_ISL_847268, EPI_ISL_847269, EPI_ISL_847271, EPI_ISL_847272, EPI_ISL_847273, EPI_ISL_847275, EPI_ISL_847276, EPI_ISL_847277, EPI_ISL_847278, EPI_ISL_847279, EPI_ISL_847281, EPI_ISL_847282, EPI_ISL_847283, EPI_ISL_847284, EPI_ISL_847285, EPI_ISL_847286, EPI_ISL_847287, EPI_ISL_847288, EPI_ISL_847289, EPI_ISL_847290, EPI_ISL_847291, EPI_ISL_847292, EPI_ISL_847293, EPI_ISL_847294, EPI_ISL_847295, EPI_ISL_847296, EPI_ISL_847297, EPI_ISL_847298, EPI_ISL_847299, EPI_ISL_847300, EPI_ISL_847301, EPI_ISL_847302, EPI_ISL_847303, EPI_ISL_847304, EPI_ISL_847305, EPI_ISL_847306, EPI_ISL_847307, EPI_ISL_847308, EPI_ISL_847309, EPI_ISL_847310, EPI_ISL_847311, EPI_ISL_847312, EPI_ISL_847313, EPI_ISL_847314, EPI_ISL_847315, EPI_ISL_847316, EPI_ISL_847317, EPI_ISL_847318, EPI_ISL_847319, EPI_ISL_847321, EPI_ISL_847322, EPI_ISL_847323, EPI_ISL_847324, EPI_ISL_847325, EPI_ISL_847326, EPI_ISL_847327, EPI_ISL_847328, EPI_ISL_847329, EPI_ISL_847330, EPI_ISL_847331, EPI_ISL_847333, EPI_ISL_847334, EPI_ISL_847336, EPI_ISL_847338, EPI_ISL_847339, EPI_ISL_847340, EPI_ISL_847341, EPI_ISL_847342, EPI_ISL_847343, EPI_ISL_847344, EPI_ISL_847345, EPI_ISL_847346, EPI_ISL_847347, EPI_ISL_847350, EPI_ISL_847354, EPI_ISL_847355, EPI_ISL_847356, EPI_ISL_847357, EPI_ISL_847359, EPI_ISL_847360, EPI_ISL_847361, EPI_ISL_847362, EPI_ISL_847363, EPI_ISL_847364, EPI_ISL_847365, EPI_ISL_847367, EPI_ISL_847371, EPI_ISL_847372, EPI_ISL_847373, EPI_ISL_847374, EPI_ISL_847375, EPI_ISL_847376, EPI_ISL_847378, EPI_ISL_847379, EPI_ISL_847380, EPI_ISL_847381, EPI_ISL_847382, EPI_ISL_847383,

We gratefully acknowledge the following Authors from the Originating laboratories responsible for obtaining the specimens, as well as the Submitting laboratories where the genome data were generated and shared via GISAID, on which this research is based.

All Submitters of data may be contacted directly via www.gisaid.org

Authors are sorted alphabetically.

Accession ID	Originating Laboratory	Submitting Laboratory	Authors
EPI_ISL_1022420, EPI_ISL_1022421, EPI_ISL_1022422, EPI_ISL_1022423, EPI_ISL_1022424, EPI_ISL_1022425, EPI_ISL_1022426, EPI_ISL_1022427, EPI_ISL_1022428, EPI_ISL_1022429, EPI_ISL_1022430, EPI_ISL_1022431, EPI_ISL_1022432, EPI_ISL_1022433, EPI_ISL_1022434, EPI_ISL_1022435, EPI_ISL_1022436, EPI_ISL_1022437, EPI_ISL_1022438, EPI_ISL_1022439, EPI_ISL_1022440, EPI_ISL_1022441, EPI_ISL_1022442, EPI_ISL_1022443, EPI_ISL_1022444, EPI_ISL_1022445, EPI_ISL_1022446, EPI_ISL_1022447, EPI_ISL_1022448, EPI_ISL_1022449, EPI_ISL_1022450, EPI_ISL_1022451, EPI_ISL_1022452, EPI_ISL_1022453, EPI_ISL_1022454, EPI_ISL_1022455, EPI_ISL_1022456, EPI_ISL_1022457, EPI_ISL_1022458, EPI_ISL_1022459, EPI_ISL_1022460, EPI_ISL_1022461, EPI_ISL_1022462, EPI_ISL_1022463, EPI_ISL_1022464, EPI_ISL_1022465, EPI_ISL_1022466, EPI_ISL_1022467, EPI_ISL_1022468, EPI_ISL_1022469, EPI_ISL_1022470, EPI_ISL_1022471, EPI_ISL_1022472, EPI_ISL_1022473, EPI_ISL_1022474, EPI_ISL_1022475, EPI_ISL_1022476, EPI_ISL_1022477, EPI_ISL_1022478, EPI_ISL_1022479, EPI_ISL_1022480, EPI_ISL_1022481, EPI_ISL_1022482, EPI_ISL_1022483, EPI_ISL_1022484, EPI_ISL_1022485, EPI_ISL_1022486, EPI_ISL_1022487, EPI_ISL_1022488, EPI_ISL_1022489, EPI_ISL_1022490, EPI_ISL_1022491, EPI_ISL_1022492, EPI_ISL_1022493, EPI_ISL_1022494, EPI_ISL_1022495, EPI_ISL_1022496, EPI_ISL_1022497, EPI_ISL_1022498, EPI_ISL_1022499, EPI_ISL_1022500, EPI_ISL_1022501, EPI_ISL_1022502, EPI_ISL_1022503, EPI_ISL_1022504, EPI_ISL_1022505, EPI_ISL_1022506, EPI_ISL_1022507, EPI_ISL_1022508, EPI_ISL_1022509, EPI_ISL_1022510, EPI_ISL_1022511, EPI_ISL_1022512, EPI_ISL_1022513, EPI_ISL_1022514, EPI_ISL_1022515, EPI_ISL_1022516, EPI_ISL_1022517, EPI_ISL_1022518, EPI_ISL_1022519, EPI_ISL_1022520, EPI_ISL_1022521, EPI_ISL_1022522, EPI_ISL_1022523, EPI_ISL_1022524, EPI_ISL_1022525, EPI_ISL_1022526, EPI_ISL_1022527, EPI_ISL_1022528, EPI_ISL_1022529, EPI_ISL_1022530, EPI_ISL_1022531, EPI_ISL_1022532, EPI_ISL_1022533, EPI_ISL_1022534, EPI_ISL_1022535, EPI_ISL_1022536, EPI_ISL_1022537, EPI_ISL_1022538, EPI_ISL_1022539, EPI_ISL_1022540, EPI_ISL_1022541, EPI_ISL_1022542, EPI_ISL_1022543, EPI_ISL_1022544, EPI_ISL_1022545, EPI_ISL_1022546, EPI_ISL_1022547, EPI_ISL_1022548, EPI_ISL_1022549, EPI_ISL_1022550, EPI_ISL_1022551, EPI_ISL_1022552, EPI_ISL_1022553, EPI_ISL_1022554, EPI_ISL_1022555, EPI_ISL_1022556, EPI_ISL_1022557, EPI_ISL_1022558, EPI_ISL_1022559, EPI_ISL_1022560, EPI_ISL_1022561, EPI_ISL_1022562, EPI_ISL_1022563, EPI_ISL_1022564, EPI_ISL_1022565, EPI_ISL_1022566, EPI_ISL_1022567, EPI_ISL_1022568, EPI_ISL_1022569, EPI_ISL_1022570, EPI_ISL_1022571, EPI_ISL_1022572, EPI_ISL_1022573, EPI_ISL_1022574, EPI_ISL_1022575, EPI_ISL_1022576, EPI_ISL_1022577, EPI_ISL_1022578, EPI_ISL_1022579, EPI_ISL_1022580, EPI_ISL_1022581, EPI_ISL_1022582, EPI_ISL_1022583, EPI_ISL_1022584, EPI_ISL_1022585, EPI_ISL_1022586, EPI_ISL_1022587, EPI_ISL_1022588, EPI_ISL_1022589, EPI_ISL_1022590, EPI_ISL_1022591, EPI_ISL_1022592, EPI_ISL_1022593, EPI_ISL_1022594, EPI_ISL_1022595, EPI_ISL_1022596, EPI_ISL_1022597, EPI_ISL_1022598, EPI_ISL_1022599, EPI_ISL_1022600, EPI_ISL_1022601, EPI_ISL_1022602, EPI_ISL_1022603, EPI_ISL_1022604, EPI_ISL_1022605, EPI_ISL_1022606, EPI_ISL_1022607, EPI_ISL_1022608, EPI_ISL_1022609, EPI_ISL_1022610, EPI_ISL_1022611, EPI_ISL_1022612, EPI_ISL_1022613, EPI_ISL_1022614, EPI_ISL_1022615, EPI_ISL_1022616, EPI_ISL_1022617, EPI_ISL_1022618, EPI_ISL_1022619, EPI_ISL_1022620, EPI_ISL_1022621, EPI_ISL_1022622, EPI_ISL_1022623, EPI_ISL_1022624, EPI_ISL_1022625, EPI_ISL_1022626, EPI_ISL_1022627, EPI_ISL_1022628, EPI_ISL_1022629, EPI_ISL_1022630, EPI_ISL_1022631, EPI_ISL_1022632, EPI_ISL_1022633, EPI_ISL_1022634, EPI_ISL_1022635, EPI_ISL_1022636, EPI_ISL_1022637, EPI_ISL_1022638, EPI_ISL_1022639, EPI_ISL_1022640, EPI_ISL_1022641, EPI_ISL_1022642, EPI_ISL_1022643, EPI_ISL_1022644, EPI_ISL_1022645, EPI_ISL_1022646, EPI_ISL_1022647, EPI_ISL_1022648, EPI_ISL_1022649, EPI_ISL_1022650, EPI_ISL_1022651, EPI_ISL_1022652, EPI_ISL_1022653, EPI_ISL_1022654, EPI_ISL_1022655, EPI_ISL_1022656, EPI_ISL_1022657, EPI_ISL_1022658, EPI_ISL_1022659, EPI_ISL_1022660, EPI_ISL_1022661, EPI_ISL_1022662, EPI_ISL_1022663, EPI_ISL_1022664, EPI_ISL_1022665, EPI_ISL_1022666, EPI_ISL_1022667, EPI_ISL_1022668, EPI_ISL_1022669, EPI_ISL_1022670, EPI_ISL_1022671, EPI_ISL_1022672, EPI_ISL_1022673, EPI_ISL_1022674, EPI_ISL_1022675, EPI_ISL_1022676, EPI_ISL_1022677, EPI_ISL_1022678, EPI_ISL_1022679, EPI_ISL_1022680, EPI_ISL_1022681, EPI_ISL_1022682, EPI_ISL_1022683, EPI_ISL_1022684, EPI_ISL_1022685, EPI_ISL_1022686, EPI_ISL_1022687, EPI_ISL_1022688, EPI_ISL_1022689, EPI_ISL_1022690, EPI_ISL_1022691, EPI_ISL_1022692, EPI_ISL_1022693, EPI_ISL_1022694, EPI_ISL_1022695, EPI_ISL_1022696, EPI_ISL_1022697, EPI_ISL_1022698, EPI_ISL_1022699, EPI_ISL_1022700, EPI_ISL_1022701, EPI_ISL_1022702, EPI_ISL_1022703, EPI_ISL_1022704, EPI_ISL_1022705, EPI_ISL_1022706, EPI_ISL_1022707, EPI_ISL_1022708, EPI_ISL_1022709, EPI_ISL_1022710, EPI_ISL_1022711, EPI_ISL_1022712, EPI_ISL_1022713, EPI_ISL_1022714, EPI_ISL_1022715, EPI_ISL_1022716, EPI_ISL_1022717, EPI_ISL_1022718, EPI_ISL_1022719, EPI_ISL_1022720, EPI_ISL_1022721, EPI_ISL_1022722, EPI_ISL_1022723, EPI_ISL_1022724, EPI_ISL_1022725, EPI_ISL_1022726, EPI_ISL_1022727, EPI_ISL_1022728, EPI_ISL_1022729, EPI_ISL_1022730, EPI_ISL_1022731, EPI_ISL_1022732, EPI_ISL_1022733, EPI_ISL_1022734, EPI_ISL_1022735, EPI_ISL_1022736, EPI_ISL_1022737, EPI_ISL_1022738, EPI_ISL_1022739, EPI_ISL_1022740, EPI_ISL_1022741, EPI_ISL_1022742, EPI_ISL_1022743, EPI_ISL_1022744, EPI_ISL_1022745, EPI_ISL_1022746, EPI_ISL_1022747, EPI_ISL_1022748, EPI_ISL_1022749, EPI_ISL_1022750, EPI_ISL_1022751, EPI_ISL_1022752, EPI_ISL_1022753, EPI_ISL_1022754, EPI_ISL_1022755, EPI_ISL_1022756, EPI_ISL_1022757, EPI_ISL_1022758, EPI_ISL_1022759, EPI_ISL_1022760, EPI_ISL_1022761, EPI_ISL_1022762, EPI_ISL_1022763, EPI_ISL_1022764, EPI_ISL_1022765, EPI_ISL_1022766, EPI_ISL_1022767, EPI_ISL_1022768, EPI_ISL_1022769, EPI_ISL_1022770, EPI_ISL_1022771, EPI_ISL_1022772, EPI_ISL_1022773, EPI_ISL_1022774, EPI_ISL_1022775, EPI_ISL_1022776, EPI_ISL_1022777, EPI_ISL_1022778, EPI_ISL_1022779, EPI_ISL_1022780, EPI_ISL_1022781, EPI_ISL_1022782, EPI_ISL_1022783, EPI_ISL_1022784, EPI_ISL_1022785, EPI_ISL_1022786, EPI_ISL_1022787, EPI_ISL_1022788, EPI_ISL_1022789, EPI_ISL_1022790, EPI_ISL_1022791, EPI_ISL_1022792, EPI_ISL_1022793, EPI_ISL_1022794, EPI_ISL_1022795, EPI_ISL_1022796, EPI_ISL_1022797, EPI_ISL_1022798, EPI_ISL_1022799, EPI_ISL_1022800, EPI_ISL_1022801, EPI_ISL_1022802, EPI_ISL_1022803, EPI_ISL_1022804, EPI_ISL_1022805, EPI_ISL_1022806, EPI_ISL_1022807, EPI_ISL_1022808, EPI_ISL_1022809, EPI_ISL_1022810, EPI_ISL_1022811, EPI_ISL_1022812, EPI_ISL_1022813, EPI_ISL_1022814, EPI_ISL_1022815, EPI_ISL_1022816, EPI_ISL_1022817, EPI_ISL_1022818, EPI_ISL_1022819, EPI_ISL_1022820, EPI_ISL_1022821, EPI_ISL_1022822, EPI_ISL_1022823, EPI_ISL_1022824, EPI_ISL_1022825, EPI_ISL_1022826, EPI_ISL_1022827, EPI_ISL_1022828, EPI_ISL_1022829, EPI_ISL_1022830, EPI_ISL_1022831, EPI_ISL_1022832, EPI_ISL_1022833, EPI_ISL_1022834, EPI_ISL_1022835, EPI_ISL_1022836, EPI_ISL_1022837, EPI_ISL_1022838, EPI_ISL_1022839, EPI_ISL_1022840, EPI_ISL_1022841, EPI_ISL_1022842, EPI_ISL_1022843, EPI_ISL_1022844, EPI_ISL_1022845, EPI_ISL_1022846, EPI_ISL_1022847, EPI_ISL_1022848, EPI_ISL_1022849, EPI_ISL_1022850, EPI_ISL_1022851, EPI_ISL_1022852, EPI_ISL_1022853, EPI_ISL_1022854, EPI_ISL_1022855, EPI_ISL_1022856, EPI_ISL_1022857, EPI_ISL_1022858, EPI_ISL_1022859, EPI_ISL_1022860, EPI_ISL_1022861, EPI_ISL_1022862, EPI_ISL_1022863, EPI_ISL_1022864, EPI_ISL_1022865, EPI_ISL_1022866, EPI_ISL_1022867, EPI_ISL_1022868, EPI_ISL_1022869, EPI_ISL_1022870, EPI_ISL_1022871, EPI_ISL_1022872, EPI_ISL_1022873, EPI_ISL_1022874, EPI_ISL_1022875, EPI_ISL_1022876, EPI_ISL_1022877, EPI_ISL_1022878, EPI_ISL_1022879, EPI_ISL_1022880, EPI_ISL_1022881, EPI_ISL_1022882, EPI_ISL_1022883, EPI_ISL_1022884, EPI_ISL_1022885, EPI_ISL_1022886, EPI_ISL_1022887, EPI_ISL_1022888, EPI_ISL_1022889, EPI_ISL_1022890, EPI_ISL_1022891, EPI_ISL_1022892, EPI_ISL_1022893, EPI_ISL_1022894, EPI_ISL_1022895, EPI_ISL_1022896, EPI_ISL_1022897, EPI_ISL_1022898, EPI_ISL_1022899, EPI_ISL_1022900, EPI_ISL_1022901, EPI_ISL_1022902, EPI_ISL_1022903, EPI_ISL_1022904, EPI_ISL_1022905, EPI_ISL_1022906, EPI_ISL_1022907, EPI_ISL_1022908, EPI_ISL_1022909, EPI_ISL_1022910, EPI_ISL_1022911, EPI_ISL_1022912, EPI_ISL_1022913, EPI_ISL_1022914, EPI_ISL_1022915, EPI_ISL_1022916, EPI_ISL_1022917, EPI_ISL_1022918, EPI_ISL_1022919, EPI_ISL_1022920, EPI_ISL_1022921, EPI_ISL_1022922, EPI_ISL_1022923, EPI_ISL_1022924, EPI_ISL_1022925, EPI_ISL_1022926, EPI_ISL_1022927, EPI_ISL_1022928, EPI_ISL_1022929, EPI_ISL_1022930, EPI_ISL_1022931, EPI_ISL_1022932, EPI_ISL_1022933, EPI_ISL_1022934, EPI_ISL_1022935, EPI_ISL_1022936, EPI_ISL_1022937, EPI_ISL_1022938, EPI_ISL_1022939, EPI_ISL_1022940, EPI_ISL_1022941, EPI_ISL_1022942, EPI_ISL_1022943, EPI_ISL_1022944, EPI_ISL_1022945, EPI_ISL_1022946, EPI_ISL_1022947, EPI_ISL_1022948, EPI_ISL_1022949, EPI_ISL_1022950, EPI_ISL_1022951, EPI_ISL_1022952, EPI_ISL_1022953, EPI_ISL_1022954, EPI_ISL_1022955, EPI_ISL_1022956, EPI_ISL_1022957, EPI_ISL_1022958, EPI_ISL_1022959, EPI_ISL_1022960, EPI_ISL_1022961, EPI_ISL_1022962, EPI_ISL_1022963, EPI_ISL_1022964, EPI_ISL_1022965, EPI_ISL_1022966, EPI_ISL_1022967, EPI_ISL_1022968, EPI_ISL_1022969, EPI_ISL_1022970, EPI_ISL_1022971, EPI_ISL_1022972, EPI_ISL_1022973, EPI_ISL_1022974, EPI_ISL_1022975, EPI_ISL_1022976, EPI_ISL_1022977, EPI_ISL_1022978, EPI_ISL_1022979, EPI_ISL_1022980, EPI_ISL_1022981, EPI_ISL_1022982, EPI_ISL_1022983, EPI_ISL_1022984, EPI_ISL_1022985, EPI_ISL_1022986, EPI_ISL_1022987, EPI_ISL_1022988, EPI_ISL_1022989, EPI_ISL_1022990, EPI_ISL_1022991, EPI_ISL_1022992, EPI_ISL_1022993, EPI_ISL_1022994, EPI_ISL_1022995, EPI_ISL_1022996, EPI_ISL_1022997, EPI_ISL_1022998, EPI_ISL_1022999, EPI_ISL_1023000, EPI_ISL_1023001, EPI_ISL_1023002, EPI_ISL_1023003, EPI_ISL_1023004, EPI_ISL_1023005, EPI_ISL_1023006, EPI_ISL_1023007, EPI_ISL_1023008, EPI_ISL_1023009, EPI_ISL_1023010, EPI_ISL_1023011, EPI_ISL_1023012, EPI_ISL_1023013, EPI_ISL_1023014, EPI_ISL_1023015, EPI_ISL_1023016, EPI_ISL_1023017, EPI_ISL_1023018, EPI_ISL_1023019, EPI_ISL_1023020, EPI_ISL_1023021, EPI_ISL_1023022, EPI_ISL_1023023, EPI_ISL_1023024, EPI_ISL_1023025, EPI_ISL_1023026, EPI_ISL_1023027, EPI_ISL_1023028, EPI_ISL_1023029, EPI_ISL_1023030, EPI_ISL_1023031, EPI_ISL_1023032, EPI_ISL_1023033, EPI_ISL_1023034, EPI_ISL_1023035, EPI_ISL_1023036, EPI_ISL_1023037, EPI_ISL_1023038, EPI_ISL_1023039, EPI_ISL_1023040, EPI_ISL_1023041, EPI_ISL_1023042, EPI_ISL_1023043, EPI_ISL_1023044, EPI_ISL_1023045, EPI_ISL_1023046, EPI_ISL_1023047, EPI_ISL_1023048, EPI_ISL_1023049, EPI_ISL_1023050, EPI_ISL_1023051, EPI_ISL_1023052, EPI_ISL_1023053, EPI_ISL_1023054, EPI_ISL_1023055, EPI_ISL_1023056, EPI_ISL_1023057, EPI_ISL_1023058, EPI_ISL_1023059, EPI_ISL_1023060, EPI_ISL_1023061, EPI_ISL_1023062, EPI_ISL_1023063, EPI_ISL_1023064, EPI_ISL_1023065, EPI_ISL_1023066, EPI_ISL_1023067, EPI_ISL_1023068, EPI_ISL_1023069, EPI_ISL_1023070, EPI_ISL_1023071, EPI_ISL_1023072, EPI_ISL_1023073, EPI_ISL_1023074, EPI_ISL_1023075, EPI_ISL_1023076, EPI_ISL_1023077, EPI_ISL_1023078, EPI_ISL_1023079, EPI_ISL_1023080, EPI_ISL_1023081, EPI_ISL_1023082, EPI_ISL_1023083, EPI_ISL_1023084, EPI_ISL_1023085, EPI_ISL_1023086, EPI_ISL_1023087, EPI_ISL_1023088, EPI_ISL_1023089, EPI_ISL_1023090, EPI_ISL_1023091, EPI_ISL_1023092, EPI_ISL_1023093, EPI_ISL_1023094, EPI_ISL_1023095, EPI_ISL_1023096, EPI_ISL_1023097, EPI_ISL_1023098, EPI_ISL_1023099, EPI_ISL_1023100, EPI_ISL_1023101, EPI_ISL_1023102, EPI_ISL_1023103, EPI_ISL_1023104, EPI_ISL_1023105, EPI_ISL_1023106, EPI_ISL_1023107, EPI_ISL_1023108, EPI_ISL_1023109, EPI_ISL_1023110, EPI_ISL_1023111, EPI_ISL_1023112, EPI_ISL_1023113, EPI_ISL_1023114, EPI_ISL_1023115, EPI_ISL_1023116, EPI_ISL_1023117, EPI_ISL_1023118, EPI_ISL_1023119, EPI_ISL_1023120, EPI_ISL_1023121, EPI_ISL_1023122, EPI_ISL_1023123, EPI_ISL_1023124, EPI_ISL_1023125, EPI_ISL_1023126, EPI_ISL_1023127, EPI_ISL_1023128, EPI_ISL_1023129, EPI_ISL_1023130, EPI_ISL_1023131, EPI_ISL_1023132, EPI_ISL_1023133, EPI_ISL_1023134, EPI_ISL_1023135, EPI_ISL_1023136, EPI_ISL_1023137, EPI_ISL_1023138, EPI_ISL_1023139, EPI_ISL_1023140, EPI_ISL_1023141, EPI_ISL_1023142, EPI_ISL_1023143, EPI_ISL_1023144, EPI_ISL_1023145, EPI_ISL_1023146, EPI_ISL_1023147, EPI_ISL_1023148, EPI_ISL_1023149, EPI_ISL_1023150, EPI_ISL_1023151, EPI_ISL_1023152, EPI_ISL_1023153, EPI_ISL_1023154, EPI_ISL_1023155, EPI_ISL_1023156, EPI_ISL_1023157, EPI_ISL_1023158, EPI_ISL_1023159, EPI_ISL_1023160, EPI_ISL_1023161, EPI_ISL_1023162, EPI_ISL_1023163, EPI_ISL_1023164, EPI_ISL_1023165, EPI_ISL_1023166, EPI_ISL_1023167, EPI_ISL_1023168, EPI_ISL_1023169, EPI_ISL_1023170, EPI_ISL_1023171, EPI_ISL_1023172, EPI_ISL_1023173, EPI_ISL_1023174, EPI_ISL_1023175, EPI_ISL_1023176, EPI_ISL_1023177, EPI_ISL_1023178, EPI_ISL_1023179, EPI_ISL_1023180, EPI_ISL_1023181, EPI_ISL_1023182, EPI_ISL_1023183, EPI_ISL_1023184, EPI_ISL_1023185, EPI_ISL_1023186, EPI_ISL_1023187, EPI_ISL_1023188, EPI_ISL_1023189, EPI_ISL_1023190, EPI_ISL_1023191, EPI_ISL_1023192, EPI_ISL_1023193, EPI_ISL_1023194, EPI_ISL_1023195, EPI_ISL_1023196, EPI_ISL_1023197, EPI_ISL_1023198, EPI_ISL_1023199, EPI_ISL_1023200, EPI_ISL_1023201, EPI_ISL_1023202, EPI_ISL_1023203, EPI_ISL_1023204, EPI_ISL_1023205, EPI_ISL_1023206, EPI_ISL_1023207, EPI_ISL_1023208, EPI_ISL_1023209, EPI_ISL_1023210, EPI_ISL_1023211, EPI_ISL_1023212, EPI_ISL_1023213, EPI_ISL_1023214, EPI_ISL_1023215, EPI_ISL_1023216, EPI_ISL_1023217, EPI_ISL_1023218, EPI_ISL_1023219, EPI_ISL_1023220, EPI_ISL_1023221, EPI_ISL_1023222, EPI_ISL_1023223, EPI_ISL_1023224, EPI_ISL_1023225, EPI_ISL_1023226, EPI_ISL_1023227, EPI_ISL_1023228, EPI_ISL_1023229, EPI_ISL_1023230, EPI_ISL_1023231, EPI_ISL_1023232, EPI_ISL_1023233, EPI_ISL_1023234, EPI_ISL_1023235, EPI_ISL_1023236, EPI_ISL_1023237, EPI_ISL_1023238, EPI_ISL_1023239, EPI_ISL_1023240, EPI_ISL_1023241, EPI_ISL_1023242, EPI_ISL_1023243, EPI_ISL_1023244, EPI_ISL_1023245, EPI_ISL_1023246, EPI_ISL_1023247, EPI_ISL_1023248, EPI_ISL_1023249, EPI_ISL_1023250, EPI_ISL_1023251, EPI_ISL_1023252, EPI_ISL_1023253, EPI_ISL_1023254, EPI_ISL_1023255, EPI_ISL_1023256, EPI_ISL_1023257, EPI_ISL_1023258, EPI_ISL_1023259, EPI_ISL_1023260, EPI_ISL_1023261, EPI_ISL_1023262, EPI_ISL_1023263, EPI_ISL_1023264, EPI_ISL_1023265, EPI_ISL_1023266, EPI_ISL_1023267, EPI_ISL_1023268, EPI_ISL_1023269, EPI_ISL_1023270, EPI_ISL_1023271, EPI_ISL_1023272, EPI_ISL_1023273, EPI_ISL_1023274, EPI_ISL_1023275, EPI_ISL_1023276, EPI_ISL_1023277, EPI_ISL_1023278, EPI_ISL_1023279, EPI_ISL_1023280, EPI_ISL_1023281, EPI_ISL_1023282, EPI_ISL_1023283, EPI_ISL_1023284, EPI_ISL_1023285, EPI_ISL_1023286, EPI_ISL_1023287, EPI_ISL_1023288, EPI_ISL_1023289, EPI_ISL_1023290, EPI_ISL_1023291, EPI_ISL_1023292, EPI_ISL_1023293, EPI_ISL_1023294, EPI_ISL_1023295, EPI_ISL_1023296, EPI_ISL_1023297, EPI_ISL_1023298, EPI_ISL_1023299, EPI_ISL_1023300, EPI_ISL_1023301, EPI_ISL_1023302, EPI_ISL_1023303, EPI_ISL_1023304, EPI_ISL_1023305, EPI_ISL_1023306, EPI_ISL_1023307, EPI_ISL_1023308, EPI_ISL_1023309, EPI_ISL_1023310, EPI_ISL_1023311, EPI_ISL_1023312, EPI_ISL_1023313, EPI_ISL_1023314, EPI_ISL_1023315, EPI_ISL_1023316, EPI_ISL_1023317, EPI_ISL_1023318, EPI_ISL_1023319, EPI_ISL_1023320, EPI_ISL_1023321, EPI_ISL_1023322, EPI_ISL_1023323, EPI_ISL_1023324, EPI_ISL_1023325, EPI_ISL_1023326, EPI_ISL_1023327, EPI_ISL_1023328, EPI_ISL_1023329, EPI_ISL_1023330, EPI_ISL_1023331, EPI_ISL_1023332, EPI_ISL_1023333, EPI_ISL_1023334, EPI_ISL_1023335, EPI_ISL_1023336, EPI_ISL_1023337, EPI_ISL_1023338, EPI_ISL_1023339, EPI_ISL_1023340, EPI_ISL_1023341, EPI_ISL_1023342, EPI_ISL_1023343, EPI_ISL_1023344, EPI_ISL_1023345, EPI_ISL_1023346, EPI_ISL_1023347, EPI_ISL_1023348, EPI_ISL_1023349, EPI_ISL_1023350, EPI_ISL_1023351, EPI_ISL_1023352, EPI_ISL_1023353, EPI_ISL_1023354, EPI_ISL_1023355, EPI_ISL_1023356, EPI_ISL_1023357, EPI_ISL_1023358, EPI_ISL_1023359, EPI_ISL_1023360, EPI_ISL_1023361, EPI_ISL_1023362, EPI_ISL_1023363, EPI_ISL_1023364, EPI_ISL_1023365, EPI_ISL_1023366, EPI_ISL_1023367, EPI_ISL_1023368, EPI_ISL_1023369, EPI_ISL_1023370, EPI_ISL_1023371, EPI_ISL_1023372, EPI_ISL_1023373, EPI_ISL_1023374, EPI_ISL_1023375, EPI_ISL_1023376, EPI_ISL_1023377, EPI_ISL_1023378, EPI_ISL_1023379, EPI_ISL_1023380, EPI_ISL_1023381, EPI_ISL_1023382, EPI_ISL_1023383, EPI_ISL_1023384, EPI_ISL_1023385, EPI_ISL_1023386, EPI_ISL_1023387, EPI_ISL_1023388, EPI_ISL_1023389, EPI_ISL_1023390, EPI_ISL_1023391, EPI_ISL_1023392, EPI_ISL_1023393, EPI_ISL_1023394, EPI_ISL_1023395, EPI_ISL_1023396, EPI_ISL_1023397, EPI_ISL_1023398, EPI_ISL_1023399, EPI_ISL_1023400, EPI_ISL_1023401, EPI_ISL_1023402, EPI_ISL_1023403, EPI_ISL_1023404, EPI_ISL_1023405, EPI_ISL_1023406, EPI_ISL_1023407, EPI_ISL_1023408, EPI_ISL_1023409, EPI_ISL_1023410, EPI_ISL_1023411, EPI_ISL_1023412, EPI_ISL_1023413, EPI_ISL_1023414, EPI_ISL_1023415, EPI_ISL_1023416, EPI_ISL_1023417, EPI_ISL_1023418, EPI_ISL_1023419, EPI_ISL_1023420, EPI_ISL_1023421, EPI_ISL_1023422, EPI_ISL_1023423, EPI_ISL_1023424, EPI_ISL_1023425, EPI_ISL_1023426, EPI_ISL_1023427, EPI_ISL_1023428, EPI_ISL_1023429, EPI_ISL_1023430, EPI_ISL_1023431, EPI_ISL_1023432, EPI_ISL_1023433, EPI_ISL_1023434, EPI_ISL_1023435, EPI_ISL_1023436, EPI_ISL_1023437, EPI_ISL_1023438, EPI_ISL_1023439, EPI_ISL_1023440, EPI_ISL_1023441, EPI_ISL_1023442, EPI_ISL_1023443, EPI_ISL_1023444, EPI_ISL_1023445, EPI_ISL_1023446, EPI_ISL_1023447, EPI_ISL_1023449, EPI_ISL_1023451, EPI_ISL_1023453, EPI_ISL_1023455, EPI_ISL_1023458, EPI_ISL_1023460, EPI_ISL_1023462, EPI_ISL_1023464, EPI_ISL_1023466, EPI_ISL_1023469, EPI_ISL_1023471, EPI_ISL_1023472, EPI_ISL_1023473, EPI_ISL_1023474, EPI_ISL_1023475, EPI_ISL_1023476, EPI_ISL_1023477, EPI_ISL_1023478, EPI_ISL_1023479, EPI_ISL_1023480, EPI_ISL_1023481, EPI_ISL_1023482, EPI_ISL_1023483, EPI_ISL_1023484, EPI_ISL_1023485, EPI_ISL_1023486, EPI_ISL_1023487, EPI_ISL_1023488, EPI_ISL_1023489, EPI_ISL_1023490, EPI_ISL_1023491, EPI_ISL_1023492, EPI_ISL_1023493, EPI_ISL_1023494, EPI_ISL_1023495, EPI_ISL_1023496, EPI_ISL_1023497, EPI_ISL_1023498, EPI_ISL_1023499, EPI_ISL_1023500, EPI_ISL_1023501, EPI_ISL_1023502, EPI_ISL_1023503, EPI_ISL_1023504, EPI_ISL_1023505, EPI_ISL_1023506, EPI_ISL_1023507, EPI_ISL_1023508, EPI_ISL_1023509, EPI_ISL_1023510, EPI_ISL_1023511, EPI_ISL_1023512, EPI_ISL_1023513, EPI_ISL_1023514, EPI_ISL_1023515, EPI_ISL_1023516, EPI_ISL_1023517, EPI_ISL_1023518, EPI_ISL_1023519, EPI_ISL_1023520, EPI_ISL_1023521, EPI_ISL_1023522, EPI_ISL_1023523, EPI_ISL_1023525, EPI_ISL_1023526, EPI_ISL_1023527, EPI_ISL_1023528, EPI_ISL_1023529, EPI_ISL_1023530, EPI_ISL_1023531, EPI_ISL_1023532, EPI_ISL_1023533, EPI_ISL_1023534, EPI_ISL_1023535, EPI_ISL_1023536, EPI_ISL_1023537, EPI_ISL_1023538, EPI_ISL_1023539, EPI_ISL_1023540, EPI_ISL_1023541, EPI_ISL_1023542, EPI_ISL_1023543, EPI_ISL_1023544, EPI_ISL_1023545, EPI_ISL_1023546, EPI_ISL_1023547, EPI_ISL_1023548, EPI_ISL_1023549, EPI_ISL_1023550, EPI_ISL_1023551, EPI_ISL_1023552, EPI_ISL_1023553, EPI_ISL_1023554, EPI_ISL_1023555, EPI_ISL_1023556, EPI_ISL_1023557, EPI_ISL_1023558, EPI_ISL_1023559, EPI_ISL_1023560, EPI_ISL_1023561, EPI_ISL_1023562, EPI_ISL_1023563, EPI_ISL_1023564, EPI_ISL_1023565, EPI_ISL_1023566, EPI_ISL_1023567, EPI_ISL_1023568, EPI_ISL_1023569, EPI_ISL_1023570, EPI_ISL_1023571, EPI_ISL_1023572, EPI_ISL_1023573, EPI_ISL_1023574, EPI_ISL_1023575, EPI_ISL_1023576, EPI_ISL_1023577, EPI_ISL_1023578, EPI_ISL_1023579, EPI_ISL_1023580, EPI_ISL_1023581, EPI_ISL_1023583, EPI_ISL_1023585, EPI_ISL_1023587, EPI_ISL_1023589, EPI_ISL_1023591, EPI_ISL_1023593, EPI_ISL_1023596, EPI_ISL_1023598, EPI_ISL_1023600, EPI_ISL_1023602, EPI_ISL_1023604, EPI_ISL_1023607, EPI_ISL_1023609, EPI_ISL_1023611, EPI_ISL_1023613, EPI_ISL_1023616, EPI_ISL_1023618, EPI_ISL_1023619, EPI_ISL_1023620, EPI_ISL_1023621, EPI_ISL_1023622, EPI_ISL_1023623, EPI_ISL_1023624, EPI_ISL_1023625, EPI_ISL_1023626, EPI_ISL_1023627, EPI_ISL_1023628, EPI_ISL_1023629, EPI_ISL_1023630, EPI_ISL_1023631, EPI_ISL_1023632, EPI_ISL_1023633, EPI_ISL_1023634, EPI_ISL_1023635, EPI_ISL_1023636, EPI_ISL_1023637, EPI_ISL_1023638, EPI_ISL_1023639, EPI_ISL_1023640, EPI_ISL_1023641, EPI_ISL_1023642, EPI_ISL_1023643, EPI_ISL_1023644, EPI_ISL_1023645, EPI_ISL_1023646, EPI_ISL_1023647, EPI_ISL_1023648, EPI_ISL_1023649, EPI_ISL_1023650, EPI_ISL_1023651, EPI_ISL_1023652, EPI_ISL_1023653, EPI_ISL_1023654, EPI_ISL_1023655, EPI_ISL_1023656, EPI_ISL_1023657, EPI_ISL_1023658, EPI_ISL_1023659, EPI_ISL_1023660, EPI_ISL_1023661, EPI_ISL_1023662, EPI_ISL_1023663, EPI_ISL_1023664, EPI_ISL_1023665, EPI_ISL_1023666, EPI_ISL_1023667, EPI_ISL_1023668, EPI_ISL_1023669, EPI_ISL_1023670, EPI_ISL_1023672, EPI_ISL_1023674, EPI_ISL_1023676, EPI_ISL_1023678, EPI_ISL_1023680, EPI_ISL_1023682, EPI_ISL_1023684, EPI_ISL_1023687, EPI_ISL_1023689, EPI_ISL_1023691, EPI_ISL_1023694, EPI_ISL_1023696, EPI_ISL_1023698, EPI_ISL_1023700, EPI_ISL_1023702, EPI_ISL_1023705, EPI_ISL_1023707, EPI_ISL_1023709, EPI_ISL_1023711, EPI_ISL_1023713, EPI_ISL_1023715, EPI_ISL_1023717, EPI_ISL_1023719, EPI_ISL_1023722, EPI_ISL_1023724, EPI_ISL_1023726, EPI_ISL_1023728, EPI_ISL_1023730, EPI_ISL_1023731, EPI_ISL_1023732, EPI_ISL_1023733, EPI_ISL_1023734, EPI_ISL_1023735, EPI_ISL_1023736, EPI_ISL_1023737, EPI_ISL_1023738, EPI_ISL_1023739, EPI_ISL_1023740, EPI_ISL_1023741, EPI_ISL_1023742, EPI_ISL_1023743, EPI_ISL_1023744, EPI_ISL_1023745, EPI_ISL_1023746, EPI_ISL_1023747, EPI_ISL_1023748, EPI_ISL_1023749, EPI_ISL_1023750, EPI_ISL_1023751, EPI_ISL_1023752, EPI_ISL_1023753, EPI_ISL_1023754, EPI_ISL_1023755, EPI_ISL_1023756, EPI_ISL_1023757, EPI_ISL_1023758, EPI_ISL_1023759, EPI_ISL_1023760,			

Scotland

We gratefully acknowledge the following Authors from the Originating laboratories responsible for obtaining the specimens, as well as the Submitting laboratories where the genome data were generated and shared via GISAID, on which this research is based.

All Submitters of data may be contacted directly via www.gisaid.org

Authors are sorted alphabetically.

Accession ID	Originating Laboratory	Submitting Laboratory	Authors
EPI_ISL_1104230, EPI_ISL_1104233	Virology Department, Royal Infirmary of Edinburgh, NHS Lothian / School of Biological Sciences, University of Edinburgh	COVID-19 Genomics UK (COG-UK) Consortium	McHugh M, Dewar R, Cotton S, Rooke S, O'Toole Á, Scher E, Hill V, McCrone JT, Colquhoun R, Yu X, Jackson B, Rambaut A, Templeton K
EPI_ISL_1208395, EPI_ISL_1208396, EPI_ISL_1208397	Lighthouse Lab in Glasgow	Wellcome Sanger Institute for the COVID-19 Genomics UK (COG-UK) Consortium	Harper VanSteenhouse, Yumi Kasai, David Gray, Carol Clugston, Anna Dominiczak and Alex Alderton, Roberto Amato, Jeffrey Barrett, Sonia Goncalves, Ewan Harrison, David K. Jackson, Ian Johnston, Dominic Kwiatkowski, Cordelia Langford, John Sillitoe on behalf of the Wellcome Sanger Institute COVID-19 Surveillance Team
EPI_ISL_1730120, EPI_ISL_1730121	Virology Department, Royal Infirmary of Edinburgh, NHS Lothian / School of Biological Sciences, University of Edinburgh	COVID-19 Genomics UK (COG-UK) Consortium	McHugh M, Dewar R, Cotton S, Gallagher A, Maloney D, Rooke S, O'Toole Á, Scher E, Hill V, McCrone JT, Colquhoun R, Yu X, Jackson B, Rambaut A, Templeton K
EPI_ISL_413221	West of Scotland Specialist Virology Centre, NHSGGC	MRC-University of Glasgow Centre for Virus Research	Emma Thomson, Antonia Ho; James Shephard, Shirin Ashraf; Kathy Smollett, Daniel Mair, Stephen Carmichael, Ana da Silva Filipe; Richard Orton, Josh Singer, David L Robertson; Andrew Rambaut; Alasdair MacLean, Rory Gunson.
EPI_ISL_414027	West of Scotland Specialist Virology Centre, NHSGGC	MRC-University of Glasgow Centre for Virus Research	Emma Thomson, Antonia Ho; Kathy Smollett, Daniel Mair, Stephen Carmichael, Ana da Silva Filipe; Richard Orton, David L Robertson; Alasdair MacLean, Rory Gunson.
EPI_ISL_415629	Virology Department, Royal Infirmary of Edinburgh, NHS Lothian	Virology Department, Royal Infirmary of Edinburgh, NHS Lothian	McHugh M, Dewar R, O'Toole Á, Rambaut A, Williams TC, Templeton K
EPI_ISL_415630, EPI_ISL_415631	West of Scotland Specialist Virology Centre, NHSGGC	MRC-University of Glasgow Centre for Virus Research	Kathy Smollett, Daniel Mair, Stephen Carmichael, Ana da Silva Filipe; Richard Orton, David L Robertson; Alasdair MacLean, Rory Gunson; Natasha Jesudason, Kathy Li, Antonia Ho; Emma Thomson.
EPI_ISL_415640	Virology Department, Royal Infirmary of Edinburgh, NHS Lothian	Virology Department, Royal Infirmary of Edinburgh, NHS Lothian	McHugh M, Dewar R, O'Toole Á, Rambaut A, Williams TC, Templeton K
EPI_ISL_425647, EPI_ISL_425648, EPI_ISL_425650, EPI_ISL_425651, EPI_ISL_425652, EPI_ISL_425653, EPI_ISL_425654, EPI_ISL_425656, EPI_ISL_425657, EPI_ISL_425658, EPI_ISL_425659, EPI_ISL_425660, EPI_ISL_425662, EPI_ISL_425663, EPI_ISL_425668, EPI_ISL_425669, EPI_ISL_425670, EPI_ISL_425671, EPI_ISL_425672, EPI_ISL_425674, EPI_ISL_425675, EPI_ISL_425676, EPI_ISL_425677, EPI_ISL_425678, EPI_ISL_425679, EPI_ISL_425682, EPI_ISL_425684, EPI_ISL_425685, EPI_ISL_425687, EPI_ISL_425688, EPI_ISL_425692, EPI_ISL_425693, EPI_ISL_425695, EPI_ISL_425696, EPI_ISL_425697, EPI_ISL_425698, EPI_ISL_425699, EPI_ISL_425700, EPI_ISL_425701, EPI_ISL_425702, EPI_ISL_425703, EPI_ISL_425704, EPI_ISL_425705, EPI_ISL_425707, EPI_ISL_425708, EPI_ISL_425709, EPI_ISL_425711, EPI_ISL_425713, EPI_ISL_425715, EPI_ISL_425716, EPI_ISL_425717, EPI_ISL_425720, EPI_ISL_425721, EPI_ISL_425722, EPI_ISL_425724, EPI_ISL_425725, EPI_ISL_425726, EPI_ISL_425727, EPI_ISL_425728, EPI_ISL_425729, EPI_ISL_425730, EPI_ISL_425731, EPI_ISL_425732, EPI_ISL_425733, EPI_ISL_425734, EPI_ISL_425735, EPI_ISL_425736, EPI_ISL_425737, EPI_ISL_425739, EPI_ISL_425740, EPI_ISL_425741, EPI_ISL_425742, EPI_ISL_425743, EPI_ISL_425744, EPI_ISL_425745, EPI_ISL_425746, EPI_ISL_425747, EPI_ISL_425748, EPI_ISL_425749, EPI_ISL_425750, EPI_ISL_425751, EPI_ISL_425752, EPI_ISL_425754, EPI_ISL_425755, EPI_ISL_425756, EPI_ISL_425757, EPI_ISL_425758, EPI_ISL_425759, EPI_ISL_425760, EPI_ISL_425761, EPI_ISL_425762, EPI_ISL_425763, EPI_ISL_425764, EPI_ISL_425765, EPI_ISL_425766, EPI_ISL_425767, EPI_ISL_425768, EPI_ISL_425769, EPI_ISL_425770, EPI_ISL_425772, EPI_ISL_425773, EPI_ISL_425774, EPI_ISL_425775, EPI_ISL_425776, EPI_ISL_425777, EPI_ISL_425778, EPI_ISL_425779, EPI_ISL_425780, EPI_ISL_425782, EPI_ISL_425783, EPI_ISL_425784, EPI_ISL_425785, EPI_ISL_425786, EPI_ISL_425787, EPI_ISL_425788, EPI_ISL_425789, EPI_ISL_425790, EPI_ISL_425791, EPI_ISL_425792, EPI_ISL_425793, EPI_ISL_425794, EPI_ISL_425795, EPI_ISL_425796, EPI_ISL_425797, EPI_ISL_425798, EPI_ISL_425799, EPI_ISL_425800, EPI_ISL_425801, EPI_ISL_425802, EPI_ISL_425803, EPI_ISL_425804, EPI_ISL_425805, EPI_ISL_425806, EPI_ISL_425807, EPI_ISL_425808, EPI_ISL_425809, EPI_ISL_425810, EPI_ISL_425811, EPI_ISL_425812, EPI_ISL_425813, EPI_ISL_425815, EPI_ISL_425816, EPI_ISL_425817, EPI_ISL_425818			
see above	West of Scotland Specialist Virology Centre, NHSGGC / MRC-University of Glasgow Centre for Virus Research	COVID-19 Genomics UK (COG-UK) Consortium	Ana da Silva Filipe, Kathy Smollett, Stephen Carmichael, Natasha Johnson, Daniel Mair, Lily Tong, Jenna Nichols; Sarah McDonald; Richard Orton, Joseph Hughes, Sreenu Vattipally, David L Robertson; Kathy Li, Natasha Jesudason, Rajiv Shah, James Shepherd, Antonia Ho, Emma Thomson; Alasdair MacLean, Rory Gunson.
EPI_ISL_425819, EPI_ISL_425820, EPI_ISL_425821, EPI_ISL_425822, EPI_ISL_425823, EPI_ISL_425824, EPI_ISL_425825, EPI_ISL_425826, EPI_ISL_425827, EPI_ISL_425828, EPI_ISL_425829, EPI_ISL_425830, EPI_ISL_425834, EPI_ISL_425836, EPI_ISL_425839, EPI_ISL_425842, EPI_ISL_425843, EPI_ISL_425844, EPI_ISL_425845, EPI_ISL_425846, EPI_ISL_425847, EPI_ISL_425848, EPI_ISL_425849, EPI_ISL_425850, EPI_ISL_425851, EPI_ISL_425852, EPI_ISL_425855, EPI_ISL_425856, EPI_ISL_425860, EPI_ISL_425861, EPI_ISL_425862, EPI_ISL_425863, EPI_ISL_425864, EPI_ISL_425865, EPI_ISL_425866, EPI_ISL_425867, EPI_ISL_425868, EPI_ISL_425869, EPI_ISL_425870, EPI_ISL_425871, EPI_ISL_425872, EPI_ISL_425873, EPI_ISL_425874, EPI_ISL_425875, EPI_ISL_425876, EPI_ISL_425877, EPI_ISL_425878, EPI_ISL_425879, EPI_ISL_425880, EPI_ISL_425881, EPI_ISL_425882, EPI_ISL_425883, EPI_ISL_425884, EPI_ISL_425885, EPI_ISL_425886, EPI_ISL_425887, EPI_ISL_425888, EPI_ISL_425889, EPI_ISL_425890, EPI_ISL_425891, EPI_ISL_425892, EPI_ISL_425893, EPI_ISL_425894, EPI_ISL_425895, EPI_ISL_425896, EPI_ISL_425897, EPI_ISL_425898, EPI_ISL_425899, EPI_ISL_425900, EPI_ISL_425901, EPI_ISL_425902, EPI_ISL_425903, EPI_ISL_425904, EPI_ISL_425905, EPI_ISL_425906, EPI_ISL_425907, EPI_ISL_425908, EPI_ISL_425911, EPI_ISL_425912, EPI_ISL_425913, EPI_ISL_425915, EPI_ISL_425916, EPI_ISL_425917, EPI_ISL_425918, EPI_ISL_425919, EPI_ISL_425920, EPI_ISL_425921, EPI_ISL_425922, EPI_ISL_425923, EPI_ISL_425924, EPI_ISL_425925, EPI_ISL_425926, EPI_ISL_425927, EPI_ISL_425928, EPI_ISL_425929, EPI_ISL_425930, EPI_ISL_425931, EPI_ISL_425932, EPI_ISL_425933, EPI_ISL_425934, EPI_ISL_425935, EPI_ISL_425936, EPI_ISL_425937, EPI_ISL_425938, EPI_ISL_425939, EPI_ISL_425940, EPI_ISL_425941, EPI_ISL_425942, EPI_ISL_425943, EPI_ISL_425944, EPI_ISL_425945, EPI_ISL_425946, EPI_ISL_425947, EPI_ISL_425948, EPI_ISL_425949, EPI_ISL_425950, EPI_ISL_425951, EPI_ISL_425952, EPI_ISL_425953, EPI_ISL_425954, EPI_ISL_425955, EPI_ISL_425956, EPI_ISL_425958, EPI_ISL_425959, EPI_ISL_425961, EPI_ISL_425962, EPI_ISL_425963, EPI_ISL_425964, EPI_ISL_425965, EPI_ISL_425966, EPI_ISL_425967, EPI_ISL_425968, EPI_ISL_425969, EPI_ISL_425971, EPI_ISL_425972, EPI_ISL_425973, EPI_ISL_425975, EPI_ISL_425976, EPI_ISL_425978, EPI_ISL_425979, EPI_ISL_425980, EPI_ISL_425982, EPI_ISL_425984, EPI_ISL_425985, EPI_ISL_425986, EPI_ISL_425987, EPI_ISL_425988, EPI_ISL_425990, EPI_ISL_425991, EPI_ISL_425992, EPI_ISL_425993, EPI_ISL_425994, EPI_ISL_425995, EPI_ISL_425996, EPI_ISL_425997, EPI_ISL_425998, EPI_ISL_425999, EPI_ISL_426000, EPI_ISL_426001, EPI_ISL_426002, EPI_ISL_426003, EPI_ISL_426004, EPI_ISL_426005, EPI_ISL_426006, EPI_ISL_426007, EPI_ISL_426008, EPI_ISL_426009, EPI_ISL_426010, EPI_ISL_426011, EPI_ISL_426012, EPI_ISL_426013, EPI_ISL_426014, EPI_ISL_426015, EPI_ISL_426016, EPI_ISL_426017, EPI_ISL_426019, EPI_ISL_426020, EPI_ISL_426021, EPI_ISL_426022, EPI_ISL_426023, EPI_ISL_426024			
see above	Virology Department, Royal Infirmary of Edinburgh, NHS Lothian / School of Biological Sciences, University of Edinburgh / Institute of Genetics and Molecular Medicine, University of Edinburgh	COVID-19 Genomics UK (COG-UK) Consortium	McHugh M, Dewar R, Rooke S, Gallagher M, Balcaza C, O'Toole A, Hill V, McCrone JT, Colquhoun R, Yu X, Jackson B, Scher E, Rambaut A, Williams TC, Templeton K
EPI_ISL_432868, EPI_ISL_432869, EPI_ISL_432870, EPI_ISL_432871, EPI_ISL_432872, EPI_ISL_432873, EPI_ISL_432874, EPI_ISL_432875, EPI_ISL_432876, EPI_ISL_432877, EPI_ISL_432878, EPI_ISL_432880, EPI_ISL_432881, EPI_ISL_432883, EPI_ISL_432885, EPI_ISL_432886, EPI_ISL_432888, EPI_ISL_432889, EPI_ISL_432890, EPI_ISL_432891, EPI_ISL_432892, EPI_ISL_432893, EPI_ISL_432894, EPI_ISL_432895, EPI_ISL_432897, EPI_ISL_432898, EPI_ISL_432899, EPI_ISL_433068, EPI_ISL_433069, EPI_ISL_433070, EPI_ISL_433071, EPI_ISL_433072, EPI_ISL_433073, EPI_ISL_433075, EPI_ISL_433076, EPI_ISL_433077, EPI_ISL_433078, EPI_ISL_433079, EPI_ISL_433080, EPI_ISL_433081, EPI_ISL_433082, EPI_ISL_433083, EPI_ISL_433084, EPI_ISL_433085, EPI_ISL_433086, EPI_ISL_433087, EPI_ISL_433088, EPI_ISL_433089, EPI_ISL_433090, EPI_ISL_433091, EPI_ISL_433092, EPI_ISL_433093, EPI_ISL_433094, EPI_ISL_433095, EPI_ISL_433096, EPI_ISL_433097, EPI_ISL_433098, EPI_ISL_433099, EPI_ISL_433100, EPI_ISL_433101, EPI_ISL_433102, EPI_ISL_433104, EPI_ISL_433105, EPI_ISL_433107, EPI_ISL_433108, EPI_ISL_433109, EPI_ISL_433110, EPI_ISL_433111, EPI_ISL_433112, EPI_ISL_433113, EPI_ISL_433114, EPI_ISL_433115, EPI_ISL_433116, EPI_ISL_433117, EPI_ISL_433118, EPI_ISL_433119, EPI_ISL_433120, EPI_ISL_433121, EPI_ISL_433122, EPI_ISL_433123, EPI_ISL_433124, EPI_ISL_433125, EPI_ISL_433126, EPI_ISL_433127, EPI_ISL_433129, EPI_ISL_433130, EPI_ISL_433131, EPI_ISL_433132, EPI_ISL_433133, EPI_ISL_433135, EPI_ISL_433136, EPI_ISL_433137, EPI_ISL_433139, EPI_ISL_433140, EPI_ISL_433141, EPI_ISL_433142, EPI_ISL_433143, EPI_ISL_433145, EPI_ISL_433147, EPI_ISL_433148, EPI_ISL_433149, EPI_ISL_433150, EPI_ISL_433153, EPI_ISL_433154, EPI_ISL_433155, EPI_ISL_433156, EPI_ISL_433162, EPI_ISL_433164, EPI_ISL_433165, EPI_ISL_433166, EPI_ISL_433167, EPI_ISL_433168, EPI_ISL_433169, EPI_ISL_433170, EPI_ISL_433171, EPI_ISL_433172, EPI_ISL_433173, EPI_ISL_433174, EPI_ISL_433175, EPI_ISL_433176, EPI_ISL_433177, EPI_ISL_433178, EPI_ISL_433180, EPI_ISL_433181, EPI_ISL_433182, EPI_ISL_433183, EPI_ISL_433184, EPI_ISL_433185, EPI_ISL_433186, EPI_ISL_433187, EPI_ISL_433188, EPI_ISL_433190, EPI_ISL_433192, EPI_ISL_433193, EPI_ISL_433194, EPI_ISL_433195, EPI_ISL_433196, EPI_ISL_433197, EPI_ISL_433198, EPI_ISL_433200, EPI_ISL_433201, EPI_ISL_433202, EPI_ISL_433203, EPI_ISL_433204, EPI_ISL_433205, EPI_ISL_433206, EPI_ISL_433207, EPI_ISL_433208, EPI_ISL_433210, EPI_ISL_433211, EPI_ISL_433213, EPI_ISL_433214, EPI_ISL_433215, EPI_ISL_433216, EPI_ISL_433217, EPI_ISL_433218, EPI_ISL_433220, EPI_ISL_433221, EPI_ISL_433222, EPI_ISL_433223, EPI_ISL_433224, EPI_ISL_433225, EPI_ISL_433226, EPI_ISL_433227, EPI_ISL_433228, EPI_ISL_433229, EPI_ISL_433233, EPI_ISL_433234, EPI_ISL_433236, EPI_ISL_433237, EPI_ISL_433238, EPI_ISL_433240, EPI_ISL_433241, EPI_ISL_433242, EPI_ISL_433243, EPI_ISL_433244, EPI_ISL_433245, EPI_ISL_433246, EPI_ISL_433247, EPI_ISL_433248, EPI_ISL_433249, EPI_ISL_433250, EPI_ISL_433253, EPI_ISL_433254, EPI_ISL_433255, EPI_ISL_433256, EPI_ISL_433257, EPI_ISL_433260, EPI_ISL_433261, EPI_ISL_433262, EPI_ISL_433263, EPI_ISL_433265, EPI_ISL_433266, EPI_ISL_433267			
see above	Virology Department, Royal Infirmary of Edinburgh, NHS Lothian / School of Biological Sciences, University of Edinburgh / Institute of Genetics and Molecular Medicine, University of Edinburgh	COVID-19 Genomics UK (COG-UK) Consortium	McHugh M, Dewar R, Rooke S, Gallagher M, Balcaza C, O'Toole A, Hill V, McCrone JT, Colquhoun R, Yu X, Jackson B, Rambaut A, Williams TC, Templeton K
EPI_ISL_433270, EPI_ISL_433274, EPI_ISL_433276, EPI_ISL_433277, EPI_ISL_433278, EPI_ISL_433279, EPI_ISL_433280, EPI_ISL_433281, EPI_ISL_433282, EPI_ISL_433283, EPI_ISL_433284, EPI_ISL_433285, EPI_ISL_433286, EPI_ISL_433287, EPI_ISL_433288, EPI_ISL_433289, EPI_ISL_433291, EPI_ISL_433293, EPI_ISL_433294, EPI_ISL_433295, EPI_ISL_433298, EPI_ISL_433299, EPI_ISL_433302, EPI_ISL_433303, EPI_ISL_433304, EPI_ISL_433305, EPI_ISL_433307, EPI_ISL_433308, EPI_ISL_433313, EPI_ISL_433314, EPI_ISL_433316, EPI_ISL_433321, EPI_ISL_433323, EPI_ISL_433324, EPI_ISL_433326, EPI_ISL_433327, EPI_ISL_433329, EPI_ISL_433330, EPI_ISL_433333, EPI_ISL_433334, EPI_ISL_433335, EPI_ISL_433338, EPI_ISL_433339, EPI_ISL_433340, EPI_ISL_433341, EPI_ISL_433342, EPI_ISL_433344, EPI_ISL_433346, EPI_ISL_433347, EPI_ISL_433348, EPI_ISL_433350, EPI_ISL_433351, EPI_ISL_433353, EPI_ISL_433355, EPI_ISL_433356, EPI_ISL_433357, EPI_ISL_433358, EPI_ISL_433359, EPI_ISL_433360, EPI_ISL_433361, EPI_ISL_433362, EPI_ISL_433364, EPI_ISL_433366, EPI_ISL_433367, EPI_ISL_433368, EPI_ISL_433370, EPI_ISL_433371, EPI_ISL_433372			
see above	West of Scotland Specialist Virology Centre, NHSGGC / MRC-University of Glasgow Centre for Virus Research	COVID-19 Genomics UK (COG-UK) Consortium	Ana da Silva Filipe, Natasha Johnson, Kathy Smollett, Daniel Mair, Stephen Carmichael, Lily Tong, Jenna Nichols, Elihu Aranday-Cortes, Kirstyn Brunker, Yasmin Parr, Kyriaki Nomikou; Sarah McDonald, Marc Niebel, Patawee Asamaphan; Richard Orton, Joseph Hughes, Sreenu Vattipally, David L Robertson; Alasdair MacLean, Rory Gunson; Kathy Li, Natasha Jesudason, Rajiv Shah, James Shepherd, Antonia Ho, Emma Thomson

We gratefully acknowledge the following Authors from the Originating laboratories responsible for obtaining the specimens, as well as the Submitting laboratories where the genome data were generated and shared via GISAID, on which this research is based.

All Submitters of data may be contacted directly via www.gisaid.org

Authors are sorted alphabetically.

Accession ID	Originating Laboratory	Submitting Laboratory	Authors
EPI_ISL_1019507, EPI_ISL_1019508, EPI_ISL_1019509, EPI_ISL_1019510, EPI_ISL_1019511, EPI_ISL_1019512, EPI_ISL_1019513, EPI_ISL_1019514, EPI_ISL_1019516, EPI_ISL_1019518, EPI_ISL_1019519, EPI_ISL_1019520, EPI_ISL_1019521, EPI_ISL_1019522, EPI_ISL_1019523, EPI_ISL_1019524, EPI_ISL_1019525, EPI_ISL_1019526, EPI_ISL_1019527, EPI_ISL_1019528, EPI_ISL_1019529, EPI_ISL_1019530, EPI_ISL_1019531, EPI_ISL_1019532, EPI_ISL_1019533, EPI_ISL_1019534, EPI_ISL_1019535, EPI_ISL_1019536, EPI_ISL_1019537, EPI_ISL_1019538, EPI_ISL_1019539, EPI_ISL_1019540, EPI_ISL_1019541, EPI_ISL_1019542, EPI_ISL_1019543, EPI_ISL_1019544, EPI_ISL_1019545, EPI_ISL_1019546, EPI_ISL_1019547, EPI_ISL_1019548, EPI_ISL_1019549, EPI_ISL_1019550, EPI_ISL_1019551, EPI_ISL_1019552, EPI_ISL_1019553, EPI_ISL_1019554, EPI_ISL_1019555, EPI_ISL_1019556, EPI_ISL_1019557, EPI_ISL_1019558, EPI_ISL_1019559, EPI_ISL_1019560, EPI_ISL_1019561, EPI_ISL_1019562, EPI_ISL_1019563, EPI_ISL_1019564, EPI_ISL_1019565, EPI_ISL_1019566, EPI_ISL_1019567, EPI_ISL_1019568, EPI_ISL_1019569, EPI_ISL_1019570, EPI_ISL_1019571, EPI_ISL_1019572, EPI_ISL_1019573, EPI_ISL_1019574, EPI_ISL_1019575, EPI_ISL_1019577, EPI_ISL_1019578, EPI_ISL_1019579, EPI_ISL_1019580, EPI_ISL_1019581, EPI_ISL_1019582, EPI_ISL_1019583, EPI_ISL_1019584, EPI_ISL_1019585			
see above	Lighthouse Lab in Glasgow	Wellcome Sanger Institute for the COVID-19 Genomics UK (COG-UK) Consortium	Harper VanSteenhouse, Yumi Kasai, David Gray, Carol Clugston, Anna Dominiczak and Alex Alderton, Roberto Amato, Jeffrey Barrett, Sonia Goncalves, Ewan Harrison, David K. Jackson, Ian Johnston, Dominic Kwiatkowski, Cordelia Langford, John Sillitoe on behalf of the Wellcome Sanger Institute COVID-19 Surveillance Team
EPI_ISL_1019586	Lighthouse Lab in Milton Keynes	Wellcome Sanger Institute for the COVID-19 Genomics UK (COG-UK) Consortium	The Lighthouse Lab in Milton Keynes and Alex Alderton, Roberto Amato, Jeffrey Barrett, Sonia Goncalves, Ewan Harrison, David K. Jackson, Ian Johnston, Dominic Kwiatkowski, Cordelia Langford, John Sillitoe on behalf of the Wellcome Sanger Institute COVID-19 Surveillance Team
EPI_ISL_1019587, EPI_ISL_1019588, EPI_ISL_1019589, EPI_ISL_1019590, EPI_ISL_1019592, EPI_ISL_1019593, EPI_ISL_1019595, EPI_ISL_1019596, EPI_ISL_1019597, EPI_ISL_1019598, EPI_ISL_1019600, EPI_ISL_1019601, EPI_ISL_1019603, EPI_ISL_1019604, EPI_ISL_1019605, EPI_ISL_1019606, EPI_ISL_1019607, EPI_ISL_1019608, EPI_ISL_1019609, EPI_ISL_1019611, EPI_ISL_1019612, EPI_ISL_1019613, EPI_ISL_1019614, EPI_ISL_1019615, EPI_ISL_1019616, EPI_ISL_1019617, EPI_ISL_1019618, EPI_ISL_1019619, EPI_ISL_1019620, EPI_ISL_1019621, EPI_ISL_1019622, EPI_ISL_1019623, EPI_ISL_1019624, EPI_ISL_1019625, EPI_ISL_1019626, EPI_ISL_1019627, EPI_ISL_1019628, EPI_ISL_1019629, EPI_ISL_1019630, EPI_ISL_1019631, EPI_ISL_1019632, EPI_ISL_1019633, EPI_ISL_1019634, EPI_ISL_1019635, EPI_ISL_1019636, EPI_ISL_1019638, EPI_ISL_1019639, EPI_ISL_1019641, EPI_ISL_1019643, EPI_ISL_1019644, EPI_ISL_1019646, EPI_ISL_1019648, EPI_ISL_1019649, EPI_ISL_1019651, EPI_ISL_1019652, EPI_ISL_1019654, EPI_ISL_1019656, EPI_ISL_1019657, EPI_ISL_1019659, EPI_ISL_1019661, EPI_ISL_1019666, EPI_ISL_1019667, EPI_ISL_1019669, EPI_ISL_1019670, EPI_ISL_1019673, EPI_ISL_1019675, EPI_ISL_1019677, EPI_ISL_1019679, EPI_ISL_1019680, EPI_ISL_1019682, EPI_ISL_1019684, EPI_ISL_1019685, EPI_ISL_1019687, EPI_ISL_1019689, EPI_ISL_1019690, EPI_ISL_1019692, EPI_ISL_1019694, EPI_ISL_1019695, EPI_ISL_1019697, EPI_ISL_1019698, EPI_ISL_1019701, EPI_ISL_1019703, EPI_ISL_1019704, EPI_ISL_1019706, EPI_ISL_1019708, EPI_ISL_1019709, EPI_ISL_1019711, EPI_ISL_1019714, EPI_ISL_1019717, EPI_ISL_1019719, EPI_ISL_1019721, EPI_ISL_1019723, EPI_ISL_1019727, EPI_ISL_1019728, EPI_ISL_1019730, EPI_ISL_1019733, EPI_ISL_1019735, EPI_ISL_1019736, EPI_ISL_1019738, EPI_ISL_1019740, EPI_ISL_1019741, EPI_ISL_1019743, EPI_ISL_1019744, EPI_ISL_1019746, EPI_ISL_1019747, EPI_ISL_1019749, EPI_ISL_1019751, EPI_ISL_1019752, EPI_ISL_1019754, EPI_ISL_1019755, EPI_ISL_1019757, EPI_ISL_1019760, EPI_ISL_1019761, EPI_ISL_1019765, EPI_ISL_1019766, EPI_ISL_1019769, EPI_ISL_1019773, EPI_ISL_1019774, EPI_ISL_1019776, EPI_ISL_1019777, EPI_ISL_1019781, EPI_ISL_1019782, EPI_ISL_1019784, EPI_ISL_1019786, EPI_ISL_1019789, EPI_ISL_1019791, EPI_ISL_1019792, EPI_ISL_1019794, EPI_ISL_1019796, EPI_ISL_1019797, EPI_ISL_1019802, EPI_ISL_1019804, EPI_ISL_1019806, EPI_ISL_1019809, EPI_ISL_1019811, EPI_ISL_1019812, EPI_ISL_1019814, EPI_ISL_1019816, EPI_ISL_1019817, EPI_ISL_1019819, EPI_ISL_1019821, EPI_ISL_1019824, EPI_ISL_1019826, EPI_ISL_1019829, EPI_ISL_1019831, EPI_ISL_1019832, EPI_ISL_1019835, EPI_ISL_1019839, EPI_ISL_1019840, EPI_ISL_1019844, EPI_ISL_1019845, EPI_ISL_1019848, EPI_ISL_1019850, EPI_ISL_1019854, EPI_ISL_1019857, EPI_ISL_1019859, EPI_ISL_1019860, EPI_ISL_1019862, EPI_ISL_1019864, EPI_ISL_1019868, EPI_ISL_1019870, EPI_ISL_1019871, EPI_ISL_1019873, EPI_ISL_1019876, EPI_ISL_1019878, EPI_ISL_1019879, EPI_ISL_1019881, EPI_ISL_1019883, EPI_ISL_1019884, EPI_ISL_1019886, EPI_ISL_1019887, EPI_ISL_1019889, EPI_ISL_1019891, EPI_ISL_1019892, EPI_ISL_1019894, EPI_ISL_1019895, EPI_ISL_1019896, EPI_ISL_1019898, EPI_ISL_1019899, EPI_ISL_1019900, EPI_ISL_1019902, EPI_ISL_1019904, EPI_ISL_1019905, EPI_ISL_1019907, EPI_ISL_1019909, EPI_ISL_1019910, EPI_ISL_1019913, EPI_ISL_1019914, EPI_ISL_1019915, EPI_ISL_1019917, EPI_ISL_1019918, EPI_ISL_1019919, EPI_ISL_1019921, EPI_ISL_1019922, EPI_ISL_1019923, EPI_ISL_1019924, EPI_ISL_1019925, EPI_ISL_1019927, EPI_ISL_1019928, EPI_ISL_1019929, EPI_ISL_1019930, EPI_ISL_1019931, EPI_ISL_1019932, EPI_ISL_1019934, EPI_ISL_1019935, EPI_ISL_1019937, EPI_ISL_1019938, EPI_ISL_1019939, EPI_ISL_1019940, EPI_ISL_1019941, EPI_ISL_1019942, EPI_ISL_1019943, EPI_ISL_1019944, EPI_ISL_1019946, EPI_ISL_1019947, EPI_ISL_1019948, EPI_ISL_1019949, EPI_ISL_1019950, EPI_ISL_1019951, EPI_ISL_1019953, EPI_ISL_1019954, EPI_ISL_1019955, EPI_ISL_1019956, EPI_ISL_1019957, EPI_ISL_1019958, EPI_ISL_1019959, EPI_ISL_1019961, EPI_ISL_1019964, EPI_ISL_1019965, EPI_ISL_1019967, EPI_ISL_1019969, EPI_ISL_1019970, EPI_ISL_1019971, EPI_ISL_1019972, EPI_ISL_1019973, EPI_ISL_1019975, EPI_ISL_1019976, EPI_ISL_1019978, EPI_ISL_1019980, EPI_ISL_1019981, EPI_ISL_1019982, EPI_ISL_1019983, EPI_ISL_1019984, EPI_ISL_1019985, EPI_ISL_1019986, EPI_ISL_1019987, EPI_ISL_1019988, EPI_ISL_1019989, EPI_ISL_1019990, EPI_ISL_1019991, EPI_ISL_1019992, EPI_ISL_1019993, EPI_ISL_1019994, EPI_ISL_1019995, EPI_ISL_1019996, EPI_ISL_1019997, EPI_ISL_1019998, EPI_ISL_1019999, EPI_ISL_1020001, EPI_ISL_1020003, EPI_ISL_1020004, EPI_ISL_1020005, EPI_ISL_1020006, EPI_ISL_1020007, EPI_ISL_1020008, EPI_ISL_1020009, EPI_ISL_1020010, EPI_ISL_1020011, EPI_ISL_1020012, EPI_ISL_1020015, EPI_ISL_1042390, EPI_ISL_1042572			
see above	Lighthouse Lab in Glasgow	Wellcome Sanger Institute for the COVID-19 Genomics UK (COG-UK) Consortium	Harper VanSteenhouse, Yumi Kasai, David Gray, Carol Clugston, Anna Dominiczak and Alex Alderton, Roberto Amato, Jeffrey Barrett, Sonia Goncalves, Ewan Harrison, David K. Jackson, Ian Johnston, Dominic Kwiatkowski, Cordelia Langford, John Sillitoe on behalf of the Wellcome Sanger Institute COVID-19 Surveillance Team
EPI_ISL_1042822	Lighthouse Lab in Milton Keynes	Wellcome Sanger Institute for the COVID-19 Genomics UK (COG-UK) Consortium	The Lighthouse Lab in Milton Keynes and Alex Alderton, Roberto Amato, Jeffrey Barrett, Sonia Goncalves, Ewan Harrison, David K. Jackson, Ian Johnston, Dominic Kwiatkowski, Cordelia Langford, John Sillitoe on behalf of the Wellcome Sanger Institute COVID-19 Surveillance Team
EPI_ISL_1043461	Lighthouse Lab in Glasgow	Wellcome Sanger Institute for the COVID-19 Genomics UK (COG-UK) Consortium	Harper VanSteenhouse, Yumi Kasai, David Gray, Carol Clugston, Anna Dominiczak and Alex Alderton, Roberto Amato, Jeffrey Barrett, Sonia Goncalves, Ewan Harrison, David K. Jackson, Ian Johnston, Dominic Kwiatkowski, Cordelia Langford, John Sillitoe on behalf of the Wellcome Sanger Institute COVID-19 Surveillance Team
EPI_ISL_1047804, EPI_ISL_1047805, EPI_ISL_1047806, EPI_ISL_1047807, EPI_ISL_1047809, EPI_ISL_1047810, EPI_ISL_1047811, EPI_ISL_1047812, EPI_ISL_1047813, EPI_ISL_1047814, EPI_ISL_1047815, EPI_ISL_1047816, EPI_ISL_1047818, EPI_ISL_1047819, EPI_ISL_1047820, EPI_ISL_1047821, EPI_ISL_1047822, EPI_ISL_1047823, EPI_ISL_1047824, EPI_ISL_1047825, EPI_ISL_1047827, EPI_ISL_1047828, EPI_ISL_1047829, EPI_ISL_1047830, EPI_ISL_1047831, EPI_ISL_1047832, EPI_ISL_1047833, EPI_ISL_1047834, EPI_ISL_1047835, EPI_ISL_1047836, EPI_ISL_1047837, EPI_ISL_1047838, EPI_ISL_1047839, EPI_ISL_1047840, EPI_ISL_1047841, EPI_ISL_1047842, EPI_ISL_1047843, EPI_ISL_1047846, EPI_ISL_1047847, EPI_ISL_1047848, EPI_ISL_1047849, EPI_ISL_1047850, EPI_ISL_1047851, EPI_ISL_1047852, EPI_ISL_1047853, EPI_ISL_1047854			
see above	West of Scotland Specialist Virology Centre, NHSGGC / MRC-University of Glasgow Centre for Virus Research	COVID-19 Genomics UK (COG-UK) Consortium	Ana da Silva Filipe, Natasha Johnson, Kathy Smollett, Daniel Mair, Stephen Carmichael, Alice Broos, Lily Tong, Jenna Nichols, Kyriaki Nomikou; Sarah McDonald; Richard Orton, Joseph Hughes, Sreenu Vattipally, David L Robertson; Alasdair MacLean, Rory Gunson; Sharif Shaaban, Matthew Holden; Rachel Blacow, Guy Mollett, Kathy Li, James Shepherd, Antonia Ho, Emma Thomson
EPI_ISL_1047857, EPI_ISL_1047858, EPI_ISL_1047859, EPI_ISL_1047860, EPI_ISL_1047861, EPI_ISL_1047862, EPI_ISL_1047863, EPI_ISL_1047864, EPI_ISL_1047865, EPI_ISL_1047866, EPI_ISL_1047867, EPI_ISL_1047868, EPI_ISL_1047869, EPI_ISL_1047871, EPI_ISL_1047872, EPI_ISL_1047873, EPI_ISL_1047874, EPI_ISL_1047875, EPI_ISL_1047876, EPI_ISL_1047878, EPI_ISL_1047879, EPI_ISL_1047880, EPI_ISL_1047881, EPI_ISL_1047882, EPI_ISL_1047883, EPI_ISL_1047884, EPI_ISL_1047888, EPI_ISL_1047889, EPI_ISL_1047890, EPI_ISL_1047891, EPI_ISL_1047892, EPI_ISL_1047893, EPI_ISL_1047894, EPI_ISL_1047895, EPI_ISL_1047899, EPI_ISL_1047900, EPI_ISL_1047901, EPI_ISL_1047902, EPI_ISL_1047903, EPI_ISL_1047904, EPI_ISL_1047905, EPI_ISL_1047906, EPI_ISL_1047907, EPI_ISL_1047908, EPI_ISL_1047909, EPI_ISL_1047910, EPI_ISL_1047912, EPI_ISL_1047913			
see above	Virology Department, Royal Infirmary of Edinburgh, NHS Lothian / School of Biological Sciences, University of Edinburgh	COVID-19 Genomics UK (COG-UK) Consortium	McHugh M, Dewar R, Cotton S, Rooke S, O'Toole Á, Scher E, Hill V, McCrone JT, Colquhoun R, Yu X, Jackson B, Rambaut A, Templeton K
EPI_ISL_1050174, EPI_ISL_1050175, EPI_ISL_1050176, EPI_ISL_1050177, EPI_ISL_1050178, EPI_ISL_1050179, EPI_ISL_1050180, EPI_ISL_1050181, EPI_ISL_1050182, EPI_ISL_1050183, EPI_ISL_1050184, EPI_ISL_1050185, EPI_ISL_1050186, EPI_ISL_1050187, EPI_ISL_1050188, EPI_ISL_1050189, EPI_ISL_1050190, EPI_ISL_1050191, EPI_ISL_1050192, EPI_ISL_1050193, EPI_ISL_1050194, EPI_ISL_1050195, EPI_ISL_1050196, EPI_ISL_1050197, EPI_ISL_1050198, EPI_ISL_1050199, EPI_ISL_1050200, EPI_ISL_1050201, EPI_ISL_1050202, EPI_ISL_1050203, EPI_ISL_1050204, EPI_ISL_1050205, EPI_ISL_1050206, EPI_ISL_1050207, EPI_ISL_1050209, EPI_ISL_1050210, EPI_ISL_1050211, EPI_ISL_1050212, EPI_ISL_1050213, EPI_ISL_1050214, EPI_ISL_1050215, EPI_ISL_1050216, EPI_ISL_1050218, EPI_ISL_1050219, EPI_ISL_1050220, EPI_ISL_1050221, EPI_ISL_1050222			
see above	West of Scotland Specialist Virology Centre, NHSGGC / MRC-University of Glasgow Centre for Virus Research	COVID-19 Genomics UK (COG-UK) Consortium	Ana da Silva Filipe, Natasha Johnson, Kathy Smollett, Daniel Mair, Stephen Carmichael, Alice Broos, Lily Tong, Jenna Nichols, Kyriaki Nomikou; Sarah McDonald; Richard Orton, Joseph Hughes, Sreenu Vattipally, David L Robertson; Alasdair MacLean, Rory Gunson; Sharif Shaaban, Matthew Holden; Rachel Blacow, Guy Mollett, Kathy Li, James Shepherd, Antonia Ho, Emma Thomson
EPI_ISL_1050223, EPI_ISL_1050225, EPI_ISL_1050226, EPI_ISL_1050227, EPI_ISL_1050228, EPI_ISL_1050229, EPI_ISL_1050230, EPI_ISL_1050231, EPI_ISL_1050232	Lighthouse Lab in Glasgow / MRC-University of Glasgow Centre for Virus Research	COVID-19 Genomics UK (COG-UK) Consortium	Ana da Silva Filipe, Natasha Johnson, Kathy Smollett, Daniel Mair, Stephen Carmichael, Alice Broos, Lily Tong, Jenna Nichols, Kyriaki Nomikou; Sarah McDonald; Harper VanSteenhouse, Yumi Kasai, David Gray, Carol Clugston, Anna Dominiczak; Alasdair MacLean, Rory Gunson; Richard Orton, Joseph Hughes, Sreenu Vattipally, David L Robertson; Sharif Shaaban, Matthew Holden; Kathy Li, James Shepherd, Antonia Ho, Emma Thomson
EPI_ISL_1050234, EPI_ISL_1050235, EPI_ISL_1050236, EPI_ISL_1050237, EPI_ISL_1050238	West of Scotland Specialist Virology Centre, NHSGGC / MRC-University of Glasgow Centre for Virus Research	COVID-19 Genomics UK (COG-UK) Consortium	Ana da Silva Filipe, Natasha Johnson, Kathy Smollett, Daniel Mair, Stephen Carmichael, Alice Broos, Lily Tong, Jenna Nichols, Kyriaki Nomikou; Sarah McDonald; Richard Orton, Joseph Hughes, Sreenu Vattipally, David L Robertson; Alasdair MacLean, Rory Gunson; Sharif Shaaban, Matthew Holden; Rachel Blacow, Guy Mollett, Kathy Li, James Shepherd, Antonia Ho, Emma Thomson
EPI_ISL_1050239	Lighthouse Lab in Glasgow / MRC-University of Glasgow Centre for Virus Research	COVID-19 Genomics UK (COG-UK) Consortium	Ana da Silva Filipe, Natasha Johnson, Kathy Smollett, Daniel Mair, Stephen Carmichael, Alice Broos, Lily Tong, Jenna Nichols, Kyriaki Nomikou; Sarah McDonald; Harper VanSteenhouse, Yumi Kasai, David Gray, Carol Clugston, Anna Dominiczak; Alasdair MacLean, Rory Gunson; Richard Orton, Joseph Hughes, Sreenu Vattipally, David L Robertson; Sharif Shaaban, Matthew Holden; Kathy Li, James Shepherd, Antonia Ho, Emma Thomson
EPI_ISL_1050240, EPI_ISL_1050241	West of Scotland Specialist Virology Centre, NHSGGC / MRC-University of Glasgow Centre for Virus Research	COVID-19 Genomics UK (COG-UK) Consortium	Ana da Silva Filipe, Natasha Johnson, Kathy Smollett, Daniel Mair, Stephen Carmichael, Alice Broos, Lily Tong, Jenna Nichols, Kyriaki Nomikou; Sarah McDonald; Richard Orton, Joseph Hughes, Sreenu Vattipally, David L Robertson; Alasdair MacLean, Rory Gunson; Sharif Shaaban, Matthew Holden; Rachel Blacow, Guy Mollett, Kathy Li, James Shepherd, Antonia Ho, Emma Thomson
EPI_ISL_1050243, EPI_ISL_1050244	Virology Department, Royal Infirmary of Edinburgh, NHS	COVID-19 Genomics UK (COG-UK) Consortium	McHugh M, Dewar R, Cotton S, Rooke S, O'Toole Á, Scher E, Hill V, McCrone JT, Colquhoun R, Yu X, Jackson B, Rambaut A, Templeton K

We gratefully acknowledge the following Authors from the Originating laboratories responsible for obtaining the specimens, as well as the Submitting laboratories where the genome data were generated and shared via GISAID, on which this research is based.

All Submitters of data may be contacted directly via www.gisaid.org

Authors are sorted alphabetically.

Accession ID	Originating Laboratory	Submitting Laboratory	Authors
EPI_ISL_1186740, EPI_ISL_1190342, EPI_ISL_1205538, EPI_ISL_1207809, EPI_ISL_1207810, EPI_ISL_1207811, EPI_ISL_1207812, EPI_ISL_1207813, EPI_ISL_1207814, EPI_ISL_1207815, EPI_ISL_1207816, EPI_ISL_1207817, EPI_ISL_1207818, EPI_ISL_1207819, EPI_ISL_1207820, EPI_ISL_1207821, EPI_ISL_1207822, EPI_ISL_1207823, EPI_ISL_1207824, EPI_ISL_1207825, EPI_ISL_1207826, EPI_ISL_1207827, EPI_ISL_1207828, EPI_ISL_1207830, EPI_ISL_1207831, EPI_ISL_1207832, EPI_ISL_1207833, EPI_ISL_1207834, EPI_ISL_1207835, EPI_ISL_1207836, EPI_ISL_1207837, EPI_ISL_1207838, EPI_ISL_1207839, EPI_ISL_1207840, EPI_ISL_1207841, EPI_ISL_1207842, EPI_ISL_1207843, EPI_ISL_1207844, EPI_ISL_1207845, EPI_ISL_1207846, EPI_ISL_1207847, EPI_ISL_1207848, EPI_ISL_1207849, EPI_ISL_1207850, EPI_ISL_1207851, EPI_ISL_1207852, EPI_ISL_1207853, EPI_ISL_1207854, EPI_ISL_1207855, EPI_ISL_1207857, EPI_ISL_1207858, EPI_ISL_1207859, EPI_ISL_1207861, EPI_ISL_1207862, EPI_ISL_1207863, EPI_ISL_1207864, EPI_ISL_1207865, EPI_ISL_1207866, EPI_ISL_1207867, EPI_ISL_1207868, EPI_ISL_1207869, EPI_ISL_1207870, EPI_ISL_1207871, EPI_ISL_1207872, EPI_ISL_1207874, EPI_ISL_1207875, EPI_ISL_1207876, EPI_ISL_1207877, EPI_ISL_1207878, EPI_ISL_1207879, EPI_ISL_1207880, EPI_ISL_1207881, EPI_ISL_1207882, EPI_ISL_1207883, EPI_ISL_1207884, EPI_ISL_1207885, EPI_ISL_1207886, EPI_ISL_1207887, EPI_ISL_1207889, EPI_ISL_1207890, EPI_ISL_1207891, EPI_ISL_1207892, EPI_ISL_1207893, EPI_ISL_1207894, EPI_ISL_1207895, EPI_ISL_1207897, EPI_ISL_1207898, EPI_ISL_1207899, EPI_ISL_1207900, EPI_ISL_1207901, EPI_ISL_1207902, EPI_ISL_1207903, EPI_ISL_1207904, EPI_ISL_1207905, EPI_ISL_1207906, EPI_ISL_1207907, EPI_ISL_1207908, EPI_ISL_1207909, EPI_ISL_1207910, EPI_ISL_1208007, EPI_ISL_1208048, EPI_ISL_1208049, EPI_ISL_1208050, EPI_ISL_1208051, EPI_ISL_1208052, EPI_ISL_1208053, EPI_ISL_1208055, EPI_ISL_1208056, EPI_ISL_1208057, EPI_ISL_1208058, EPI_ISL_1208060, EPI_ISL_1208061, EPI_ISL_1208062, EPI_ISL_1208063, EPI_ISL_1208064, EPI_ISL_1208065, EPI_ISL_1208066, EPI_ISL_1208067, EPI_ISL_1208069, EPI_ISL_1208070, EPI_ISL_1208071, EPI_ISL_1208072, EPI_ISL_1208073, EPI_ISL_1208074, EPI_ISL_1208075, EPI_ISL_1208076, EPI_ISL_1208078, EPI_ISL_1208079, EPI_ISL_1208080, EPI_ISL_1208081, EPI_ISL_1208082, EPI_ISL_1208083, EPI_ISL_1208084, EPI_ISL_1208085, EPI_ISL_1208086, EPI_ISL_1208087, EPI_ISL_1208088, EPI_ISL_1208089, EPI_ISL_1208091, EPI_ISL_1208092, EPI_ISL_1208093, EPI_ISL_1208094, EPI_ISL_1208095, EPI_ISL_1208096, EPI_ISL_1208098, EPI_ISL_1208099, EPI_ISL_1208101, EPI_ISL_1208103, EPI_ISL_1208104, EPI_ISL_1208105, EPI_ISL_1208107, EPI_ISL_1208108, EPI_ISL_1208109, EPI_ISL_1208111, EPI_ISL_1208112, EPI_ISL_1208113, EPI_ISL_1208114, EPI_ISL_1208115, EPI_ISL_1208116, EPI_ISL_1208265, EPI_ISL_1208267, EPI_ISL_1208268, EPI_ISL_1208269, EPI_ISL_1208270, EPI_ISL_1208271, EPI_ISL_1208272, EPI_ISL_1208273, EPI_ISL_1208274, EPI_ISL_1208275, EPI_ISL_1208276, EPI_ISL_1208277, EPI_ISL_1208278, EPI_ISL_1208279, EPI_ISL_1208280, EPI_ISL_1208281, EPI_ISL_1208283, EPI_ISL_1208284, EPI_ISL_1208285, EPI_ISL_1208286, EPI_ISL_1208287, EPI_ISL_1208288, EPI_ISL_1208289, EPI_ISL_1208290, EPI_ISL_1208291, EPI_ISL_1208292, EPI_ISL_1208293, EPI_ISL_1208295, EPI_ISL_1208296, EPI_ISL_1208299, EPI_ISL_1208300, EPI_ISL_1208301, EPI_ISL_1208303, EPI_ISL_1208304, EPI_ISL_1208305, EPI_ISL_1208306, EPI_ISL_1208307, EPI_ISL_1208308, EPI_ISL_1208310, EPI_ISL_1208312, EPI_ISL_1208313, EPI_ISL_1208314, EPI_ISL_1208315, EPI_ISL_1208316, EPI_ISL_1208317, EPI_ISL_1208318, EPI_ISL_1208319, EPI_ISL_1208320, EPI_ISL_1208322, EPI_ISL_1208323, EPI_ISL_1208324, EPI_ISL_1208326, EPI_ISL_1208327, EPI_ISL_1208328, EPI_ISL_1208329, EPI_ISL_1208330, EPI_ISL_1208331, EPI_ISL_1208332, EPI_ISL_1208334, EPI_ISL_1208335, EPI_ISL_1208336, EPI_ISL_1208337, EPI_ISL_1208340, EPI_ISL_1208341, EPI_ISL_1208342, EPI_ISL_1208343, EPI_ISL_1208344, EPI_ISL_1208347, EPI_ISL_1208348, EPI_ISL_1208349, EPI_ISL_1208350, EPI_ISL_1208351, EPI_ISL_1208354, EPI_ISL_1222950, EPI_ISL_1222987, EPI_ISL_1243144, EPI_ISL_1244644, EPI_ISL_1245011, EPI_ISL_1245013, EPI_ISL_1245018, EPI_ISL_1245024, EPI_ISL_1245026, EPI_ISL_1245027, EPI_ISL_1245030, EPI_ISL_1245035, EPI_ISL_1245036, EPI_ISL_1245040, EPI_ISL_1245042, EPI_ISL_1245043, EPI_ISL_1245044, EPI_ISL_1245046, EPI_ISL_1245047, EPI_ISL_1245048, EPI_ISL_1245051, EPI_ISL_1245054, EPI_ISL_1245060, EPI_ISL_1245061, EPI_ISL_1245063, EPI_ISL_1245067, EPI_ISL_1245073, EPI_ISL_1245076, EPI_ISL_1245079, EPI_ISL_1245081, EPI_ISL_1245085, EPI_ISL_1245087, EPI_ISL_1245088, EPI_ISL_1245094, EPI_ISL_1245095, EPI_ISL_1245101, EPI_ISL_1245102, EPI_ISL_1245108, EPI_ISL_1245110, EPI_ISL_1245111, EPI_ISL_1245117, EPI_ISL_1245123, EPI_ISL_1245125, EPI_ISL_1245127, EPI_ISL_1245128, EPI_ISL_1245129, EPI_ISL_1245130, EPI_ISL_1245132, EPI_ISL_1245133, EPI_ISL_1245134, EPI_ISL_1245135, EPI_ISL_1245138, EPI_ISL_1245143, EPI_ISL_1245144, EPI_ISL_1245146, EPI_ISL_1245149, EPI_ISL_1245151, EPI_ISL_1245152, EPI_ISL_1245155, EPI_ISL_1245161, EPI_ISL_1245166, EPI_ISL_1245167, EPI_ISL_1245169, EPI_ISL_1245176, EPI_ISL_1245180, EPI_ISL_1245181, EPI_ISL_1245182, EPI_ISL_1245186, EPI_ISL_1245187, EPI_ISL_1245190, EPI_ISL_1245193, EPI_ISL_1245194, EPI_ISL_1245198, EPI_ISL_1245202, EPI_ISL_1245203, EPI_ISL_1245206, EPI_ISL_1245207, EPI_ISL_1245212, EPI_ISL_1245216, EPI_ISL_1245217, EPI_ISL_1245218, EPI_ISL_1245224, EPI_ISL_1245226, EPI_ISL_1245228, EPI_ISL_1245234, EPI_ISL_1245235, EPI_ISL_1245236, EPI_ISL_1245241, EPI_ISL_1245244, EPI_ISL_1245245, EPI_ISL_1245246, EPI_ISL_1245247, EPI_ISL_1245249, EPI_ISL_1245256, EPI_ISL_1245257, EPI_ISL_1245259, EPI_ISL_1245261, EPI_ISL_1245266, EPI_ISL_1245267, EPI_ISL_1245268, EPI_ISL_1245270, EPI_ISL_1245275, EPI_ISL_1245276, EPI_ISL_1245277, EPI_ISL_1245279, EPI_ISL_1245280, EPI_ISL_1245282, EPI_ISL_1245290, EPI_ISL_1245292, EPI_ISL_1245293, EPI_ISL_1245297, EPI_ISL_1245298, EPI_ISL_1245300, EPI_ISL_1245301, EPI_ISL_1245302, EPI_ISL_1245304, EPI_ISL_1245305, EPI_ISL_1245308, EPI_ISL_1245313, EPI_ISL_1245314, EPI_ISL_1245316, EPI_ISL_1245317, EPI_ISL_1245319, EPI_ISL_1245321, EPI_ISL_1245326, EPI_ISL_1245328, EPI_ISL_1245330, EPI_ISL_1245333, EPI_ISL_1245334, EPI_ISL_1245335, EPI_ISL_1245336, EPI_ISL_1245339, EPI_ISL_1245344, EPI_ISL_1245346, EPI_ISL_1245349, EPI_ISL_1245352, EPI_ISL_1245355, EPI_ISL_1245357			
see above	Lighthouse Lab in Glasgow	Wellcome Sanger Institute for the COVID-19 Genomics UK (COG-UK) Consortium	Harper VanSteenhouse, Yumi Kasai, David Gray, Carol Clugston, Anna Dominiczak and Alex Alderton, Roberto Amato, Jeffrey Barrett, Sonia Goncalves, Ewan Harrison, David K. Jackson, Ian Johnston, Dominic Kwiatkowski, Cordelia Langford, John Sillitoe on behalf of the Wellcome Sanger Institute COVID-19 Surveillance Team
EPI_ISL_1245587	Lighthouse Lab in Milton Keynes	Wellcome Sanger Institute for the COVID-19 Genomics UK (COG-UK) Consortium	The Lighthouse Lab in Milton Keynes and Alex Alderton, Roberto Amato, Jeffrey Barrett, Sonia Goncalves, Ewan Harrison, David K. Jackson, Ian Johnston, Dominic Kwiatkowski, Cordelia Langford, John Sillitoe on behalf of the Wellcome Sanger Institute COVID-19 Surveillance Team
EPI_ISL_1246289, EPI_ISL_1246290, EPI_ISL_1246291, EPI_ISL_1246292, EPI_ISL_1246293, EPI_ISL_1246294, EPI_ISL_1246295, EPI_ISL_1246296, EPI_ISL_1246297, EPI_ISL_1246298, EPI_ISL_1246299, EPI_ISL_1246300, EPI_ISL_1246301, EPI_ISL_1246302, EPI_ISL_1246303, EPI_ISL_1246304, EPI_ISL_1246305, EPI_ISL_1246306, EPI_ISL_1246307, EPI_ISL_1246308, EPI_ISL_1246309, EPI_ISL_1246310, EPI_ISL_1246311, EPI_ISL_1246312, EPI_ISL_1246313, EPI_ISL_1246314, EPI_ISL_1246315, EPI_ISL_1246316, EPI_ISL_1246317, EPI_ISL_1246318, EPI_ISL_1246319, EPI_ISL_1246320, EPI_ISL_1246321, EPI_ISL_1246322, EPI_ISL_1246323, EPI_ISL_1246325, EPI_ISL_1246326, EPI_ISL_1246327, EPI_ISL_1246328, EPI_ISL_1246329, EPI_ISL_1246330, EPI_ISL_1246331, EPI_ISL_1246332, EPI_ISL_1246333, EPI_ISL_1246334, EPI_ISL_1246335, EPI_ISL_1246336, EPI_ISL_1246337, EPI_ISL_1246338, EPI_ISL_1246339, EPI_ISL_1246340, EPI_ISL_1246341, EPI_ISL_1246342, EPI_ISL_1246343, EPI_ISL_1246344, EPI_ISL_1246345, EPI_ISL_1246346, EPI_ISL_1246347, EPI_ISL_1246348, EPI_ISL_1246349, EPI_ISL_1246351, EPI_ISL_1246352, EPI_ISL_1246353, EPI_ISL_1246354, EPI_ISL_1246355, EPI_ISL_1246356, EPI_ISL_1246357, EPI_ISL_1246358, EPI_ISL_1246359, EPI_ISL_1246361, EPI_ISL_1246362, EPI_ISL_1246363, EPI_ISL_1246364, EPI_ISL_1246365, EPI_ISL_1246366, EPI_ISL_1246367, EPI_ISL_1246368, EPI_ISL_1246369, EPI_ISL_1246370, EPI_ISL_1246371, EPI_ISL_1246372, EPI_ISL_1246373, EPI_ISL_1246374, EPI_ISL_1246375, EPI_ISL_1246376, EPI_ISL_1246377, EPI_ISL_1246378, EPI_ISL_1246379, EPI_ISL_1246380, EPI_ISL_1246381, EPI_ISL_1246382, EPI_ISL_1246384, EPI_ISL_1246385, EPI_ISL_1246386, EPI_ISL_1246387, EPI_ISL_1246388, EPI_ISL_1246389, EPI_ISL_1246391, EPI_ISL_1246392, EPI_ISL_1246393, EPI_ISL_1246394, EPI_ISL_1246395, EPI_ISL_1246396, EPI_ISL_1246397, EPI_ISL_1246398, EPI_ISL_1246399, EPI_ISL_1246400, EPI_ISL_1246401, EPI_ISL_1246402, EPI_ISL_1246403, EPI_ISL_1246404, EPI_ISL_1246406, EPI_ISL_1246407, EPI_ISL_1246408, EPI_ISL_1246409, EPI_ISL_1246410, EPI_ISL_1246411, EPI_ISL_1246413, EPI_ISL_1246414, EPI_ISL_1246415, EPI_ISL_1246416, EPI_ISL_1246418, EPI_ISL_1246420, EPI_ISL_1246422, EPI_ISL_1246423, EPI_ISL_1246424, EPI_ISL_1246425, EPI_ISL_1246426, EPI_ISL_1246427, EPI_ISL_1246428, EPI_ISL_1246429, EPI_ISL_1246430, EPI_ISL_1246431, EPI_ISL_1246432, EPI_ISL_1246433, EPI_ISL_1246434, EPI_ISL_1246435, EPI_ISL_1246436, EPI_ISL_1246437, EPI_ISL_1246438, EPI_ISL_1246439, EPI_ISL_1246440, EPI_ISL_1246441, EPI_ISL_1246442, EPI_ISL_1246443, EPI_ISL_1246444, EPI_ISL_1246445, EPI_ISL_1246446, EPI_ISL_1246447, EPI_ISL_1246449, EPI_ISL_1246451, EPI_ISL_1246452, EPI_ISL_1246453, EPI_ISL_1246455, EPI_ISL_1246456, EPI_ISL_1246457, EPI_ISL_1246458, EPI_ISL_1246459, EPI_ISL_1246460, EPI_ISL_1246461, EPI_ISL_1246462, EPI_ISL_1246463, EPI_ISL_1246464, EPI_ISL_1246465, EPI_ISL_1246467, EPI_ISL_1246468, EPI_ISL_1246469, EPI_ISL_1246471, EPI_ISL_1246472, EPI_ISL_1246473, EPI_ISL_1246474, EPI_ISL_1246475, EPI_ISL_1246476, EPI_ISL_1246477, EPI_ISL_1246478, EPI_ISL_1246479, EPI_ISL_1246480, EPI_ISL_1246481, EPI_ISL_1246482, EPI_ISL_1246483, EPI_ISL_1246484, EPI_ISL_1246485, EPI_ISL_1246487, EPI_ISL_1246488, EPI_ISL_1246489, EPI_ISL_1246490, EPI_ISL_1246491, EPI_ISL_1246492, EPI_ISL_1246493, EPI_ISL_1246494, EPI_ISL_1246495, EPI_ISL_1246496, EPI_ISL_1246497, EPI_ISL_1246498, EPI_ISL_1246499, EPI_ISL_1246500, EPI_ISL_1246501, EPI_ISL_1246502, EPI_ISL_1246505, EPI_ISL_1246506, EPI_ISL_1246507, EPI_ISL_1246508, EPI_ISL_1246509, EPI_ISL_1246510, EPI_ISL_1246512, EPI_ISL_1246513, EPI_ISL_1246515, EPI_ISL_1246516, EPI_ISL_1246517, EPI_ISL_1246518, EPI_ISL_1246519, EPI_ISL_1246520, EPI_ISL_1246521, EPI_ISL_1246522, EPI_ISL_1246523, EPI_ISL_1246524, EPI_ISL_1246525, EPI_ISL_1246527, EPI_ISL_1246528, EPI_ISL_1246529, EPI_ISL_1246530, EPI_ISL_1246531, EPI_ISL_1246532, EPI_ISL_1246534, EPI_ISL_1246535, EPI_ISL_1246536, EPI_ISL_1246537, EPI_ISL_1246538, EPI_ISL_1246539, EPI_ISL_1246540, EPI_ISL_1246541, EPI_ISL_1246542, EPI_ISL_1246543, EPI_ISL_1246544, EPI_ISL_1246545, EPI_ISL_1246546, EPI_ISL_1246547, EPI_ISL_1246548, EPI_ISL_1246549, EPI_ISL_1246550, EPI_ISL_1246551, EPI_ISL_1246552, EPI_ISL_1246553, EPI_ISL_1246554, EPI_ISL_1246556, EPI_ISL_1246557, EPI_ISL_1246558, EPI_ISL_1246559, EPI_ISL_1246560, EPI_ISL_1246561, EPI_ISL_1246562, EPI_ISL_1246563, EPI_ISL_1246564, EPI_ISL_1246565, EPI_ISL_1246566, EPI_ISL_1246567, EPI_ISL_1246568, EPI_ISL_1246569, EPI_ISL_1246570, EPI_ISL_1246571, EPI_ISL_1246572, EPI_ISL_1246573, EPI_ISL_1246574, EPI_ISL_1246575, EPI_ISL_1246576, EPI_ISL_1246577, EPI_ISL_1246578, EPI_ISL_1246579, EPI_ISL_1246580, EPI_ISL_1246581, EPI_ISL_1246582, EPI_ISL_1246583, EPI_ISL_1246584, EPI_ISL_1246585, EPI_ISL_1246586, EPI_ISL_1246587, EPI_ISL_1246588, EPI_ISL_1246589, EPI_ISL_1246590, EPI_ISL_1246591, EPI_ISL_1246592, EPI_ISL_1246593, EPI_ISL_1246594, EPI_ISL_1246595, EPI_ISL_1246596, EPI_ISL_1246597, EPI_ISL_1246598, EPI_ISL_1246599, EPI_ISL_1246600, EPI_ISL_1246601, EPI_ISL_1246602, EPI_ISL_1246603, EPI_ISL_1246604, EPI_ISL_1246605, EPI_ISL_1246606, EPI_ISL_1246608, EPI_ISL_1246609, EPI_ISL_1246610, EPI_ISL_1246611, EPI_ISL_1246612, EPI_ISL_1246613, EPI_ISL_1246614, EPI_ISL_1246615, EPI_ISL_1246616, EPI_ISL_1246617, EPI_ISL_1246618, EPI_ISL_1246619, EPI_ISL_1246620, EPI_ISL_1246621, EPI_ISL_1246622, EPI_ISL_1246623, EPI_ISL_1246624, EPI_ISL_1246625, EPI_ISL_1246626, EPI_ISL_1246627, EPI_ISL_1246628, EPI_ISL_1246629, EPI_ISL_1246630, EPI_ISL_1246631, EPI_ISL_1246632, EPI_ISL_1246633, EPI_ISL_1246634, EPI_ISL_1246636, EPI_ISL_1246637, EPI_ISL_1246638, EPI_ISL_1246639, EPI_ISL_1246640, EPI_ISL_1246641, EPI_ISL_1246642, EPI_ISL_1246643, EPI_ISL_1246644, EPI_ISL_1246645, EPI_ISL_1246646, EPI_ISL_1246647, EPI_ISL_1246648, EPI_ISL_1246649, EPI_ISL_1246650, EPI_ISL_1246651, EPI_ISL_1246652, EPI_ISL_1246653, EPI_ISL_1246654, EPI_ISL_1246655, EPI_ISL_1246656, EPI_ISL_1246657, EPI_ISL_1246658, EPI_ISL_1246659, EPI_ISL_1246660, EPI_ISL_1246661, EPI_ISL_1246662, EPI_ISL_1246663, EPI_ISL_1246664, EPI_ISL_1246665, EPI_ISL_1246666, EPI_ISL_1246667, EPI_ISL_1246668, EPI_ISL_1246669, EPI_ISL_1246670, EPI_ISL_1246671, EPI_ISL_1246672, EPI_ISL_1246673, EPI_ISL_1246674, EPI_ISL_1246675, EPI_ISL_1246676, EPI_ISL_1246677, EPI_ISL_1246678, EPI_ISL_1246679, EPI_ISL_1246680, EPI_ISL_1246681, EPI_ISL_1246682, EPI_ISL_1246683, EPI_ISL_1246684, EPI_ISL_1246685, EPI_ISL_1246686, EPI_ISL_1246687, EPI_ISL_1246688, EPI_ISL_1246689, EPI_ISL_1246690, EPI_ISL_1246691, EPI_ISL_1246693, EPI_ISL_1246694, EPI_ISL_1246695, EPI_ISL_1246696, EPI_ISL_1246698, EPI_ISL_1246699, EPI_ISL_1246700, EPI_ISL_1246701, EPI_ISL_1246702, EPI_ISL_1246703, EPI_ISL_1246704, EPI_ISL_1246705, EPI_ISL_1246706, EPI_ISL_1246707, EPI_ISL_1246708, EPI_ISL_1246709, EPI_ISL_1246710, EPI_ISL_1246711, EPI_ISL_1246712, EPI_ISL_1246713, EPI_ISL_1246714, EPI_ISL_1246715, EPI_ISL_1246716, EPI_ISL_1246717, EPI_ISL_1246719, EPI_ISL_1246720, EPI_ISL_1246721, EPI_ISL_1246723, EPI_ISL_1246724, EPI_ISL_1246725, EPI_ISL_1246726, EPI_ISL_1246727, EPI_ISL_1246728, EPI_ISL_1246729, EPI_ISL_1246730, EPI_ISL_1246731, EPI_ISL_1246732, EPI_ISL_1246733, EPI_ISL_1246734, EPI_ISL_1246735, EPI_ISL_1246736, EPI_ISL_1246737, EPI_ISL_1246738, EPI_ISL_1246739, EPI_ISL_1246740, EPI_ISL_1246741, EPI_ISL_1246742, EPI_ISL_1246743, EPI_ISL_1246744, EPI_ISL_1246745, EPI_ISL_1246746, EPI_ISL_1246748, EPI_ISL_1246749, EPI_ISL_1246750, EPI_ISL_1246751, EPI_ISL_1246752, EPI_ISL_1246753, EPI_ISL_1246754, EPI_ISL_1246755, EPI_ISL_1246756, EPI_ISL_1246757, EPI_ISL_1246758, EPI_ISL_1246759, EPI_ISL_1246760, EPI_ISL_1246761, EPI_ISL_1246762, EPI_ISL_1246763, EPI_ISL_1246765, EPI_ISL_1246766, EPI_ISL_1246767, EPI_ISL_1246768, EPI_ISL_1246769, EPI_ISL_1246770, EPI_ISL_1246771, EPI_ISL_1246772, EPI_ISL_1246773, EPI_ISL_1246774, EPI_ISL_1246776, EPI_ISL_1246777, EPI_ISL_1246778, EPI_ISL_1246779, EPI_ISL_1246780, EPI_ISL_1246781, EPI_ISL_1246782, EPI_ISL_1246783, EPI_ISL_1246784, EPI_ISL_1246785, EPI_ISL_1246786, EPI_ISL_1246787, EPI_ISL_1246788, EPI_ISL_1246789, EPI_ISL_1246790, EPI_ISL_1246791, EPI_ISL_1246792, EPI_ISL_1246793, EPI_ISL_1246794, EPI_ISL_1246795, EPI_ISL_1246796, EPI_ISL_1246797, EPI_ISL_1246798, EPI_ISL_1246799, EPI_ISL_1246800, EPI_ISL_1246801, EPI_ISL_1246802, EPI_ISL_1246803, EPI_ISL_1246804, EPI_ISL_1246805, EPI_ISL_1246806, EPI_ISL_1246807, EPI_ISL_1246808, EPI_ISL_1246809, EPI_ISL_1246810, EPI_ISL_1246811, EPI_ISL_1246812, EPI_ISL_1246813, EPI_ISL_1246814, EPI_ISL_1246815, EPI_ISL_1246816, EPI_ISL_1246817, EPI_ISL_1246818, EPI_ISL_1246819, EPI_ISL_1246820, EPI_ISL_1246821, EPI_ISL_1246822, EPI_ISL_1246823, EPI_ISL_1246824, EPI_ISL_1246825, EPI_ISL_1246826, EPI_ISL_1246827, EPI_ISL_1246828, EPI_ISL_1246829, EPI_ISL_1246830, EPI_ISL_1246831, EPI_ISL_1246832, EPI_ISL_1246833, EPI_ISL_1246834, EPI_ISL_1246835, EPI_ISL_1246836, EPI_ISL_1246837, EPI_ISL_1246838, EPI_ISL_1246840, EPI_ISL_1246841, EPI_ISL_1246842, EPI_ISL_1246843, EPI_ISL_1246844, EPI_ISL_1246845, EPI_ISL_1246846, EPI_ISL_1246847, EPI_ISL_1246848, EPI_ISL_1246849, EPI_ISL_1246850, EPI_ISL_1246851, EPI_ISL_1246852, EPI_ISL_1246853, EPI_ISL_1246854, EPI_ISL_1246855, EPI_ISL_1246875, EPI_ISL_1246876, EPI_ISL_1246877, EPI_ISL_1246878, EPI_ISL_1246879, EPI_ISL_1246880, EPI_ISL_1246881, EPI_ISL_1246882, EPI_ISL_1246884, EPI_ISL_1246885, EPI_ISL_1246886, EPI_ISL_1246887, EPI_ISL_1246889, EPI_ISL_1246890, EPI_ISL_1246891, EPI_ISL_1246892, EPI_ISL_1246893, EPI_ISL_1246894, EPI_ISL_1246895, EPI_ISL_1246896, EPI_ISL_1246898, EPI_ISL_1246899, EPI_ISL_1246901, EPI_ISL_1246902, EPI_ISL_1246903, EPI_ISL_1246904, EPI_ISL_1246905, EPI_ISL_1246906, EPI_ISL_1246907, EPI_ISL_1246908, EPI_ISL_1246909, EPI_ISL_1246910, EPI_ISL_1246911, EPI_ISL_1246912, EPI_ISL_1246913, EPI_ISL_1246914, EPI_ISL_1246915, EPI_ISL_1246916, EPI_ISL_1246918, EPI_ISL_1246919, EPI_ISL_1246920, EPI_ISL_1246921, EPI_ISL_1246922, EPI_ISL_1246923, EPI_ISL_1246924, EPI_ISL_1246925, EPI_ISL_1246926, EPI_ISL_1246927, EPI_ISL_1246928, EPI_ISL_1246929, EPI_ISL_1246931, EPI_ISL_1246932, EPI_ISL_1246933, EPI_ISL_1246934, EPI_ISL_1246935, EPI_ISL_1246936, EPI_ISL_1246937, EPI_ISL_1246939, EPI_ISL_1246940, EPI_ISL_1246941, EPI_ISL_1246942, EPI_ISL_1246943, EPI_ISL_1246944, EPI_ISL_1246945, EPI_ISL_1246946, EPI_ISL_1246947, EPI_ISL_1246949, EPI_ISL_1246950, EPI_ISL_1246951, EPI_ISL_1246952			
see above	Lighthouse Lab in Glasgow	Wellcome Sanger Institute for the COVID-19 Genomics UK (COG-UK) Consortium	Harper VanSteenhouse, Yumi Kasai, David Gray, Carol Clugston, Anna Dominiczak and Alex Alderton, Roberto Amato, Jeffrey Barrett, Sonia Goncalves, Ewan Harrison, David K. Jackson, Ian Johnston, Dominic Kwiatkowski, Cordelia Langford, John Sillitoe on behalf of the Wellcome Sanger Institute COVID-19 Surveillance Team
EPI_ISL_1247640, EPI_ISL_1247685	West of Scotland Specialist Virology Centre, NHSGGC / MRC-University of Glasgow Centre for Virus Research	COVID-19 Genomics UK (COG-UK) Consortium	Ana da Silva Filipe, Natasha Johnson, Kathy Smollett, Daniel Mair, Stephen Carmichael, Alice Broos, Lily Tong, Jenna Nichols, Kyriaki Nomikou; Sarah McDonald; Richard Orton, Joseph Hughes, Sreenu Vattipally, David L Robertson; Alasdair MacLean, Rory Gunson; Sharif Shaaban, Matthew Holden; Rachel Blacow, Guy Mollett, Kathy Li, James Shepherd, Antonia Ho, Emma Thomson
EPI_ISL_1250124, EPI_ISL_1250126, EPI_ISL_1250132, EPI_ISL_1250133, EPI_ISL_1250137, EPI_ISL_1250141, EPI_ISL_1250144, EPI_ISL_1250148, EPI_ISL_1250152, EPI_ISL_1250155, EPI_ISL_1250164, EPI_ISL_1250167, EPI_ISL_1250171, EPI_ISL_1250172, EPI_ISL_1250181, EPI_ISL_1250185, EPI_ISL_1250187, EPI_ISL_1250191, EPI_ISL_1250192, EPI_ISL_1250199, EPI_ISL_1250203, EPI_ISL_1250206, EPI_ISL_1250207, EPI_ISL_1250210, EPI_ISL_1250216, EPI_ISL_1250217, EPI_ISL_1250218, EPI_ISL_1250219, EPI_ISL_1250221			
see above	Department of Pathology, University of Cambridge	COVID-19 Genomics UK (COG-UK) Consortium	Aminu S. Jahun, Yasmin Chaudhry, Iliana Georgana, Myra Hosmillo, Rhys Izuagbe, William L. Hamilton, Martin D. Curran, Surendra Parmar, Ian

We gratefully acknowledge the following Authors from the Originating laboratories responsible for obtaining the specimens, as well as the Submitting laboratories where the genome data were generated and shared via GISAID, on which this research is based.

All Submitters of data may be contacted directly via www.gisaid.org

Authors are sorted alphabetically.

Accession ID	Originating Laboratory	Submitting Laboratory	Authors
EPI_ISL_1506134, EPI_ISL_1506135, EPI_ISL_1506136, EPI_ISL_1506137, EPI_ISL_1506138, EPI_ISL_1506140, EPI_ISL_1506141, EPI_ISL_1506142, EPI_ISL_1506143, EPI_ISL_1506146, EPI_ISL_1506147, EPI_ISL_1506148, EPI_ISL_1506149, EPI_ISL_1506150, EPI_ISL_1506151, EPI_ISL_1506152, EPI_ISL_1506153, EPI_ISL_1506154, EPI_ISL_1506155, EPI_ISL_1506156, EPI_ISL_1506160, EPI_ISL_1506161, EPI_ISL_1506162, EPI_ISL_1506164, EPI_ISL_1506166, EPI_ISL_1506167, EPI_ISL_1506168, EPI_ISL_1506169, EPI_ISL_1506170			
see above	Lighthouse Lab in Glasgow	Wellcome Sanger Institute for the COVID-19 Genomics UK (COG-UK) Consortium	Harper VanSteenhouse, Yumi Kasai, David Gray, Carol Clugston, Anna Dominiczak and Alex Alderton, Roberto Amato, Jeffrey Barrett, Sonia Goncalves, Ewan Harrison, David K. Jackson, Ian Johnston, Dominic Kwiatkowski, Cordelia Langford, John Sillitoe on behalf of the Wellcome Sanger Institute COVID-19 Surveillance Team
EPI_ISL_1506172	Lighthouse Lab in Cambridge	Wellcome Sanger Institute for the COVID-19 Genomics UK (COG-UK) Consortium	Rob Howes, The Lighthouse Lab in Cambridge and Alex Alderton, Roberto Amato, Jeffrey Barrett, Sonia Goncalves, Ewan Harrison, David K. Jackson, Ian Johnston, Dominic Kwiatkowski, Cordelia Langford, John Sillitoe on behalf of the Wellcome Sanger Institute COVID-19 Surveillance Team
EPI_ISL_1506173, EPI_ISL_1506174, EPI_ISL_1506176, EPI_ISL_1506177, EPI_ISL_1506178, EPI_ISL_1506179, EPI_ISL_1506180, EPI_ISL_1506181, EPI_ISL_1506182, EPI_ISL_1506183, EPI_ISL_1506184, EPI_ISL_1506185, EPI_ISL_1506186, EPI_ISL_1506187, EPI_ISL_1506188, EPI_ISL_1506189, EPI_ISL_1506190, EPI_ISL_1506191, EPI_ISL_1506193, EPI_ISL_1506194, EPI_ISL_1506195, EPI_ISL_1506196, EPI_ISL_1506197, EPI_ISL_1506198, EPI_ISL_1506199, EPI_ISL_1506200, EPI_ISL_1506201, EPI_ISL_1506202, EPI_ISL_1506203, EPI_ISL_1506204, EPI_ISL_1506205, EPI_ISL_1506206, EPI_ISL_1506209, EPI_ISL_1506210, EPI_ISL_1506211, EPI_ISL_1506212, EPI_ISL_1506213, EPI_ISL_1506215, EPI_ISL_1506217, EPI_ISL_1506218, EPI_ISL_1506219, EPI_ISL_1506220, EPI_ISL_1506221, EPI_ISL_1506223, EPI_ISL_1506224, EPI_ISL_1506225, EPI_ISL_1506226, EPI_ISL_1506227, EPI_ISL_1506228, EPI_ISL_1506229, EPI_ISL_1506231, EPI_ISL_1506233, EPI_ISL_1506234, EPI_ISL_1506235, EPI_ISL_1506236, EPI_ISL_1506237, EPI_ISL_1506238, EPI_ISL_1506239, EPI_ISL_1506240, EPI_ISL_1506241, EPI_ISL_1506242, EPI_ISL_1506243, EPI_ISL_1506244, EPI_ISL_1506245, EPI_ISL_1506246, EPI_ISL_1506247, EPI_ISL_1506248, EPI_ISL_1506249, EPI_ISL_1506250, EPI_ISL_1506252, EPI_ISL_1506254, EPI_ISL_1506255, EPI_ISL_1506256, EPI_ISL_1506257, EPI_ISL_1506258, EPI_ISL_1506259, EPI_ISL_1506260, EPI_ISL_1506261, EPI_ISL_1506262, EPI_ISL_1506263, EPI_ISL_1506264, EPI_ISL_1506265, EPI_ISL_1506267, EPI_ISL_1506268, EPI_ISL_1506269, EPI_ISL_1506270, EPI_ISL_1506271, EPI_ISL_1506272, EPI_ISL_1506273, EPI_ISL_1506274, EPI_ISL_1506275, EPI_ISL_1506276, EPI_ISL_1506279, EPI_ISL_1506280, EPI_ISL_1506281, EPI_ISL_1506282, EPI_ISL_1506283, EPI_ISL_1506284, EPI_ISL_1506285, EPI_ISL_1506286, EPI_ISL_1506287, EPI_ISL_1506288, EPI_ISL_1506289, EPI_ISL_1506290, EPI_ISL_1506291, EPI_ISL_1506293, EPI_ISL_1506294, EPI_ISL_1506295, EPI_ISL_1506296, EPI_ISL_1506297, EPI_ISL_1506298, EPI_ISL_1506299, EPI_ISL_1506300, EPI_ISL_1506301, EPI_ISL_1506302, EPI_ISL_1506303, EPI_ISL_1506304, EPI_ISL_1506306, EPI_ISL_1506307, EPI_ISL_1506308, EPI_ISL_1506309, EPI_ISL_1506310, EPI_ISL_1506311, EPI_ISL_1506312, EPI_ISL_1506313, EPI_ISL_1506314, EPI_ISL_1506315, EPI_ISL_1506316, EPI_ISL_1506317, EPI_ISL_1506318, EPI_ISL_1506319, EPI_ISL_1506320, EPI_ISL_1506322, EPI_ISL_1506323, EPI_ISL_1506324, EPI_ISL_1506325, EPI_ISL_1506326, EPI_ISL_1506327, EPI_ISL_1506328, EPI_ISL_1506329, EPI_ISL_1506330, EPI_ISL_1506331, EPI_ISL_1506332, EPI_ISL_1506333, EPI_ISL_1506334, EPI_ISL_1506336, EPI_ISL_1506337, EPI_ISL_1506338, EPI_ISL_1506339, EPI_ISL_1506340, EPI_ISL_1506341, EPI_ISL_1506342, EPI_ISL_1506343, EPI_ISL_1506344, EPI_ISL_1506345, EPI_ISL_1506346, EPI_ISL_1506347, EPI_ISL_1506348, EPI_ISL_1506349, EPI_ISL_1506350, EPI_ISL_1506351, EPI_ISL_1506352, EPI_ISL_1506354, EPI_ISL_1506355, EPI_ISL_1506356, EPI_ISL_1506357, EPI_ISL_1506358, EPI_ISL_1506359, EPI_ISL_1506360, EPI_ISL_1506361, EPI_ISL_1506362, EPI_ISL_1506363, EPI_ISL_1506364, EPI_ISL_1506365, EPI_ISL_1506366, EPI_ISL_1506367, EPI_ISL_1506368, EPI_ISL_1506369, EPI_ISL_1506370, EPI_ISL_1506371, EPI_ISL_1506372, EPI_ISL_1506373, EPI_ISL_1506374, EPI_ISL_1506375, EPI_ISL_1506376, EPI_ISL_1506377, EPI_ISL_1506378, EPI_ISL_1506379, EPI_ISL_1506380, EPI_ISL_1506382, EPI_ISL_1506383, EPI_ISL_1506384, EPI_ISL_1506385, EPI_ISL_1506386, EPI_ISL_1506387, EPI_ISL_1506388, EPI_ISL_1506389, EPI_ISL_1506391, EPI_ISL_1506392, EPI_ISL_1506393, EPI_ISL_1506394, EPI_ISL_1506395, EPI_ISL_1506396, EPI_ISL_1506398, EPI_ISL_1506399, EPI_ISL_1506400, EPI_ISL_1506401, EPI_ISL_1506402, EPI_ISL_1506403, EPI_ISL_1506406, EPI_ISL_1506408, EPI_ISL_1506409, EPI_ISL_1506410, EPI_ISL_1506411, EPI_ISL_1506413, EPI_ISL_1506414, EPI_ISL_1506415, EPI_ISL_1506416, EPI_ISL_1506417, EPI_ISL_1506418, EPI_ISL_1506419, EPI_ISL_1506420, EPI_ISL_1506421, EPI_ISL_1506422, EPI_ISL_1506423, EPI_ISL_1506428, EPI_ISL_1506429, EPI_ISL_1506430, EPI_ISL_1506432, EPI_ISL_1506433, EPI_ISL_1506435, EPI_ISL_1506436, EPI_ISL_1506437, EPI_ISL_1506438, EPI_ISL_1506439, EPI_ISL_1506441, EPI_ISL_1506442, EPI_ISL_1506443, EPI_ISL_1506444, EPI_ISL_1506446, EPI_ISL_1506447, EPI_ISL_1506448, EPI_ISL_1506449, EPI_ISL_1506450, EPI_ISL_1506451, EPI_ISL_1506454, EPI_ISL_1506455, EPI_ISL_1506457, EPI_ISL_1506458, EPI_ISL_1506459, EPI_ISL_1506460, EPI_ISL_1506462, EPI_ISL_1506463, EPI_ISL_1506464, EPI_ISL_1506465, EPI_ISL_1506466, EPI_ISL_1506467, EPI_ISL_1506468, EPI_ISL_1506469, EPI_ISL_1506470, EPI_ISL_1506471, EPI_ISL_1506472, EPI_ISL_1506473, EPI_ISL_1506474, EPI_ISL_1506475, EPI_ISL_1506476, EPI_ISL_1506477, EPI_ISL_1506478, EPI_ISL_1506479, EPI_ISL_1506481, EPI_ISL_1506482, EPI_ISL_1506483, EPI_ISL_1506484, EPI_ISL_1506485, EPI_ISL_1506486, EPI_ISL_1506487, EPI_ISL_1506488, EPI_ISL_1506489, EPI_ISL_1506490, EPI_ISL_1506491, EPI_ISL_1506492, EPI_ISL_1506493, EPI_ISL_1506494, EPI_ISL_1506495, EPI_ISL_1506496, EPI_ISL_1506497, EPI_ISL_1506498, EPI_ISL_1506499, EPI_ISL_1506500, EPI_ISL_1506501, EPI_ISL_1506502, EPI_ISL_1506503, EPI_ISL_1506504, EPI_ISL_1506505, EPI_ISL_1506506, EPI_ISL_1506507, EPI_ISL_1506508, EPI_ISL_1506510, EPI_ISL_1506511, EPI_ISL_1506537, EPI_ISL_1506538, EPI_ISL_1506539, EPI_ISL_1506540, EPI_ISL_1506542, EPI_ISL_1506543, EPI_ISL_1506544, EPI_ISL_1506545, EPI_ISL_1506546, EPI_ISL_1506547, EPI_ISL_1506548, EPI_ISL_1506550, EPI_ISL_1506551, EPI_ISL_1506552, EPI_ISL_1506553, EPI_ISL_1506554, EPI_ISL_1506555, EPI_ISL_1506556, EPI_ISL_1506557, EPI_ISL_1506558, EPI_ISL_1506559, EPI_ISL_1506560, EPI_ISL_1506561, EPI_ISL_1506563, EPI_ISL_1506566, EPI_ISL_1506568, EPI_ISL_1506569, EPI_ISL_1506570, EPI_ISL_1506608, EPI_ISL_1506635, EPI_ISL_1506649, EPI_ISL_1506656, EPI_ISL_1506666, EPI_ISL_1506670, EPI_ISL_1506672, EPI_ISL_1506673, EPI_ISL_1506676, EPI_ISL_1506713, EPI_ISL_1506740, EPI_ISL_1506751, EPI_ISL_1507078, EPI_ISL_1507086, EPI_ISL_1507087, EPI_ISL_1507095, EPI_ISL_1507147, EPI_ISL_1507150, EPI_ISL_1507155, EPI_ISL_1507156, EPI_ISL_1507161, EPI_ISL_1507193, EPI_ISL_1507195, EPI_ISL_1507255, EPI_ISL_1507526, EPI_ISL_1537142, EPI_ISL_1537143, EPI_ISL_1537144, EPI_ISL_1537145, EPI_ISL_1537146, EPI_ISL_1537147, EPI_ISL_1537148, EPI_ISL_1537149, EPI_ISL_1537150, EPI_ISL_1537151, EPI_ISL_1537153, EPI_ISL_1537154, EPI_ISL_1537155, EPI_ISL_1537156, EPI_ISL_1537157, EPI_ISL_1537158, EPI_ISL_1537159, EPI_ISL_1537161, EPI_ISL_1537162, EPI_ISL_1537163, EPI_ISL_1537164, EPI_ISL_1537165, EPI_ISL_1537166, EPI_ISL_1537167, EPI_ISL_1537168, EPI_ISL_1537169, EPI_ISL_1537170, EPI_ISL_1537171, EPI_ISL_1537172, EPI_ISL_1537173, EPI_ISL_1537174, EPI_ISL_1537175, EPI_ISL_1537176, EPI_ISL_1537177, EPI_ISL_1537178, EPI_ISL_1537179, EPI_ISL_1537180, EPI_ISL_1537181, EPI_ISL_1537182, EPI_ISL_1537183, EPI_ISL_1537184, EPI_ISL_1537185, EPI_ISL_1537186, EPI_ISL_1537187, EPI_ISL_1537188, EPI_ISL_1537189, EPI_ISL_1537190, EPI_ISL_1537191, EPI_ISL_1537192, EPI_ISL_1537195, EPI_ISL_1537196, EPI_ISL_1537197, EPI_ISL_1537198, EPI_ISL_1537199, EPI_ISL_1537200, EPI_ISL_1537201, EPI_ISL_1537202, EPI_ISL_1537203, EPI_ISL_1537204, EPI_ISL_1537205, EPI_ISL_1537206, EPI_ISL_1537207, EPI_ISL_1537208, EPI_ISL_1537210, EPI_ISL_1537211, EPI_ISL_1537212, EPI_ISL_1537213, EPI_ISL_1537214, EPI_ISL_1537215, EPI_ISL_1537216, EPI_ISL_1537217, EPI_ISL_1537218, EPI_ISL_1537219, EPI_ISL_1537220, EPI_ISL_1537221, EPI_ISL_1537222, EPI_ISL_1537223, EPI_ISL_1537224, EPI_ISL_1537225, EPI_ISL_1537227, EPI_ISL_1537228, EPI_ISL_1537229, EPI_ISL_1537230, EPI_ISL_1537231, EPI_ISL_1537232, EPI_ISL_1537233, EPI_ISL_1537234, EPI_ISL_1537235, EPI_ISL_1537237, EPI_ISL_1537238, EPI_ISL_1537239, EPI_ISL_1537241, EPI_ISL_1537242, EPI_ISL_1537243, EPI_ISL_1537244, EPI_ISL_1537245, EPI_ISL_1537246, EPI_ISL_1537247, EPI_ISL_1537249, EPI_ISL_1537250, EPI_ISL_1537251, EPI_ISL_1537252, EPI_ISL_1537253, EPI_ISL_1537254, EPI_ISL_1537255, EPI_ISL_1537256, EPI_ISL_1537257, EPI_ISL_1537258, EPI_ISL_1537259, EPI_ISL_1537260, EPI_ISL_1537261, EPI_ISL_1537262, EPI_ISL_1537263, EPI_ISL_1537264, EPI_ISL_1537265, EPI_ISL_1537266, EPI_ISL_1537267, EPI_ISL_1537268, EPI_ISL_1537269, EPI_ISL_1537270, EPI_ISL_1537271, EPI_ISL_1537272, EPI_ISL_1537273, EPI_ISL_1537274, EPI_ISL_1537275, EPI_ISL_1537276, EPI_ISL_1537277, EPI_ISL_1537278, EPI_ISL_1537279, EPI_ISL_1537280, EPI_ISL_1537281, EPI_ISL_1537282, EPI_ISL_1537284, EPI_ISL_1537285, EPI_ISL_1537286, EPI_ISL_1537287, EPI_ISL_1537288, EPI_ISL_1537289, EPI_ISL_1537290, EPI_ISL_1537291, EPI_ISL_1537292, EPI_ISL_1537293, EPI_ISL_1537294, EPI_ISL_1537295, EPI_ISL_1537296, EPI_ISL_1537297, EPI_ISL_1537298, EPI_ISL_1537299, EPI_ISL_1537300, EPI_ISL_1537301, EPI_ISL_1537302, EPI_ISL_1537303, EPI_ISL_1537304, EPI_ISL_1537305, EPI_ISL_1537306, EPI_ISL_1537307, EPI_ISL_1537308, EPI_ISL_1537309, EPI_ISL_1537310, EPI_ISL_1537311, EPI_ISL_1537312, EPI_ISL_1537313, EPI_ISL_1537314, EPI_ISL_1537845, EPI_ISL_1537846, EPI_ISL_1537847, EPI_ISL_1537848, EPI_ISL_1537849, EPI_ISL_1537850, EPI_ISL_1537851, EPI_ISL_1537852, EPI_ISL_1537853, EPI_ISL_1537854, EPI_ISL_1537855, EPI_ISL_1537856, EPI_ISL_1537857, EPI_ISL_1537858, EPI_ISL_1537859, EPI_ISL_1537860, EPI_ISL_1537861, EPI_ISL_1537862, EPI_ISL_1537863, EPI_ISL_1537864, EPI_ISL_1537865, EPI_ISL_1537866, EPI_ISL_1537867, EPI_ISL_1537868, EPI_ISL_1537869, EPI_ISL_1537870, EPI_ISL_1537871, EPI_ISL_1537872, EPI_ISL_1537873, EPI_ISL_1537874, EPI_ISL_1537875, EPI_ISL_1537876, EPI_ISL_1537877, EPI_ISL_1537878, EPI_ISL_1537879, EPI_ISL_1537880, EPI_ISL_1537881, EPI_ISL_1537882, EPI_ISL_1537884, EPI_ISL_1537885, EPI_ISL_1537886, EPI_ISL_1537887, EPI_ISL_1537888, EPI_ISL_1537889, EPI_ISL_1537890, EPI_ISL_1537891, EPI_ISL_1537893, EPI_ISL_1537894, EPI_ISL_1537895, EPI_ISL_1537896, EPI_ISL_1537897, EPI_ISL_1537898, EPI_ISL_1537899, EPI_ISL_1537900, EPI_ISL_1537901, EPI_ISL_1537902, EPI_ISL_1537903, EPI_ISL_1537904, EPI_ISL_1537905, EPI_ISL_1537906, EPI_ISL_1537907, EPI_ISL_1537908, EPI_ISL_1537909, EPI_ISL_1537910, EPI_ISL_1537911, EPI_ISL_1537912, EPI_ISL_1537913, EPI_ISL_1537914, EPI_ISL_1537915, EPI_ISL_1537916, EPI_ISL_1537917, EPI_ISL_1537918, EPI_ISL_1537919, EPI_ISL_1537920, EPI_ISL_1537921, EPI_ISL_1537922, EPI_ISL_1537923, EPI_ISL_1537924, EPI_ISL_1537925, EPI_ISL_1537926, EPI_ISL_1537927, EPI_ISL_1537928, EPI_ISL_1537929, EPI_ISL_1537930, EPI_ISL_1537931, EPI_ISL_1537932, EPI_ISL_1537933, EPI_ISL_1537934, EPI_ISL_1537935, EPI_ISL_1537937, EPI_ISL_1537938, EPI_ISL_1537939, EPI_ISL_1537940, EPI_ISL_1537941, EPI_ISL_1537942, EPI_ISL_1537943, EPI_ISL_1537944, EPI_ISL_1537945, EPI_ISL_1537946, EPI_ISL_1537947, EPI_ISL_1537948, EPI_ISL_1537949, EPI_ISL_1537950, EPI_ISL_1537951, EPI_ISL_1537952, EPI_ISL_1537953, EPI_ISL_1537954, EPI_ISL_1537955, EPI_ISL_1537958, EPI_ISL_1537959, EPI_ISL_1537960, EPI_ISL_1537961, EPI_ISL_1537962, EPI_ISL_1537963, EPI_ISL_1537964, EPI_ISL_1537965, EPI_ISL_1537966, EPI_ISL_1537967, EPI_ISL_1537968, EPI_ISL_1537969, EPI_ISL_1537970, EPI_ISL_1537971, EPI_ISL_1537972, EPI_ISL_1537973, EPI_ISL_1537974, EPI_ISL_1537976, EPI_ISL_1537977, EPI_ISL_1537978, EPI_ISL_1537979, EPI_ISL_1537980, EPI_ISL_1537981, EPI_ISL_1537982, EPI_ISL_1537983, EPI_ISL_1537984, EPI_ISL_1537985, EPI_ISL_1537986, EPI_ISL_1537988, EPI_ISL_1537989, EPI_ISL_1537990, EPI_ISL_1537991, EPI_ISL_1537992, EPI_ISL_1537993, EPI_ISL_1537994, EPI_ISL_1537995, EPI_ISL_1537996, EPI_ISL_1537997, EPI_ISL_1537998, EPI_ISL_1537999, EPI_ISL_1538000, EPI_ISL_1538001, EPI_ISL_1538002, EPI_ISL_1538003, EPI_ISL_1538004, EPI_ISL_1538005, EPI_ISL_1538006, EPI_ISL_1538007, EPI_ISL_1538008, EPI_ISL_1538009, EPI_ISL_1538010, EPI_ISL_1538011, EPI_ISL_1538012, EPI_ISL_1538013, EPI_ISL_1538014, EPI_ISL_1538015, EPI_ISL_1538016, EPI_ISL_1538017, EPI_ISL_1538018, EPI_ISL_1538019, EPI_ISL_1538021, EPI_ISL_1538022, EPI_ISL_1538023, EPI_ISL_1538024, EPI_ISL_1538025, EPI_ISL_1538026, EPI_ISL_1538027, EPI_ISL_1538028, EPI_ISL_1538030, EPI_ISL_1538032, EPI_ISL_1538033, EPI_ISL_1538034, EPI_ISL_1538035, EPI_ISL_1538037, EPI_ISL_1538038, EPI_ISL_1538041, EPI_ISL_1538042, EPI_ISL_1538044, EPI_ISL_1538045, EPI_ISL_1538046, EPI_ISL_1538047, EPI_ISL_1538048, EPI_ISL_1538050, EPI_ISL_1538051, EPI_ISL_1538052, EPI_ISL_1538054, EPI_ISL_1538055, EPI_ISL_1538056			
see above	Lighthouse Lab in Glasgow	Wellcome Sanger Institute for the COVID-19 Genomics UK (COG-UK) Consortium	Harper VanSteenhouse, Yumi Kasai, David Gray, Carol Clugston, Anna Dominiczak and Alex Alderton, Roberto Amato, Jeffrey Barrett, Sonia Goncalves, Ewan Harrison, David K. Jackson, Ian Johnston, Dominic Kwiatkowski, Cordelia Langford, John Sillitoe on behalf of the Wellcome Sanger Institute COVID-19 Surveillance Team
EPI_ISL_1538057	Lighthouse Lab in Cambridge	Wellcome Sanger Institute for the COVID-19 Genomics UK (COG-UK) Consortium	Rob Howes, The Lighthouse Lab in Cambridge and Alex Alderton, Roberto Amato, Jeffrey Barrett, Sonia Goncalves, Ewan Harrison, David K. Jackson, Ian Johnston, Dominic Kwiatkowski, Cordelia Langford, John Sillitoe on behalf of the Wellcome Sanger Institute COVID-19 Surveillance Team
EPI_ISL_1538058, EPI_ISL_1538059, EPI_ISL_1538060, EPI_ISL_1538061, EPI_ISL_1538062	Lighthouse Lab in Glasgow	Wellcome Sanger Institute for the COVID-19 Genomics UK (COG-UK) Consortium	Harper VanSteenhouse, Yumi Kasai, David Gray, Carol Clugston, Anna Dominiczak and Alex Alderton, Roberto Amato, Jeffrey Barrett, Sonia Goncalves, Ewan Harrison, David K. Jackson, Ian Johnston, Dominic Kwiatkowski, Cordelia Langford, John Sillitoe on behalf of the Wellcome Sanger Institute COVID-19 Surveillance Team
EPI_ISL_1538063	Lighthouse Lab in Cambridge	Wellcome Sanger Institute for the COVID-19 Genomics UK (COG-UK) Consortium	Rob Howes, The Lighthouse Lab in Cambridge and Alex Alderton, Roberto Amato, Jeffrey Barrett, Sonia Goncalves, Ewan Harrison, David K. Jackson, Ian Johnston, Dominic Kwiatkowski, Cordelia Langford, John Sillitoe on behalf of the Wellcome Sanger Institute COVID-19 Surveillance Team
EPI_ISL_1538064, EPI_ISL_1538065, EPI_ISL_1538066	Lighthouse Lab in Glasgow	Wellcome Sanger Institute for the COVID-19 Genomics UK (COG-UK) Consortium	Harper VanSteenhouse, Yumi Kasai, David Gray, Carol Clugston, Anna Dominiczak and Alex Alderton, Roberto Amato, Jeffrey Barrett, Sonia Goncalves, Ewan Harrison, David K. Jackson, Ian Johnston, Dominic Kwiatkowski, Cordelia Langford, John Sillitoe on behalf of the Wellcome Sanger Institute COVID-19 Surveillance Team
EPI_ISL_1538067	Lighthouse Lab in Cambridge	Wellcome Sanger Institute for the COVID-19 Genomics UK (COG-UK) Consortium	Rob Howes, The Lighthouse Lab in Cambridge and Alex Alderton, Roberto Amato, Jeffrey Barrett, Sonia Goncalves, Ewan Harrison, David K. Jackson, Ian Johnston, Dominic Kwiatkowski, Cordelia Langford, John Sillitoe on behalf of the Wellcome Sanger Institute COVID-19 Surveillance Team
EPI_ISL_1538068	Lighthouse Lab in Glasgow	Wellcome Sanger Institute for the COVID-19 Genomics UK (COG-UK) Consortium	Harper VanSteenhouse, Yumi Kasai, David Gray, Carol Clugston, Anna Dominiczak and Alex Alderton, Roberto Amato, Jeffrey Barrett, Sonia Goncalves, Ewan Harrison, David K. Jackson, Ian Johnston, Dominic Kwiatkowski, Cordelia Langford, John Sillitoe on behalf of the Wellcome Sanger Institute COVID-19 Surveillance Team
EPI_ISL_1538072	Lighthouse Lab in Cambridge	Wellcome Sanger Institute for the COVID-19 Genomics UK (COG-UK) Consortium	Rob Howes, The Lighthouse Lab in Cambridge and Alex Alderton, Roberto Amato, Jeffrey Barrett, Sonia Goncalves, Ewan Harrison, David K. Jackson, Ian Johnston, Dominic Kwiatkowski, Cordelia Langford, John Sillitoe on behalf of the Wellcome Sanger Institute COVID-19 Surveillance Team

Switzerland

We gratefully acknowledge the following Authors from the Originating laboratories responsible for obtaining the specimens, as well as the Submitting laboratories where the genome data were generated and shared via GISAID, on which this research is based.

All Submitters of data may be contacted directly via www.gisaid.org

Authors are sorted alphabetically.

Accession ID	Originating Laboratory	Submitting Laboratory	Authors
EPI_ISL_1002357	Viollier AG	Department of Biosystems Science and Engineering, ETH Zürich	Chaoran Chen, Sarah Nadeau, Catharine Aquino, Ivan Topolsky, Philipp Jablonski, Lara Fuhrmann, David Dreifuss, Katharina Jahn, Andreia Cabral de Gouvea, Maria Domenica Moccia, Simon Grüter, Timothy Sykes, Lennart Opitz, Griffin White, Laura Neff, Doris Popovic, Andrea Patrignani, Jay Tracy, Ralph Schlapbach, Christiane Beckmann, Maurice Redondo, Olivier Kobel, Christoph Noppen, Sophie Seidel, Noemie Santamaria de Souza, Niko Beerenwinkel, Tanja Stadler
EPI_ISL_1004814, EPI_ISL_1004815, EPI_ISL_1004816, EPI_ISL_1004817, EPI_ISL_1004818, EPI_ISL_1004819, EPI_ISL_1004820, EPI_ISL_1004821, EPI_ISL_1004822, EPI_ISL_1004823, EPI_ISL_1004824, EPI_ISL_1004825, EPI_ISL_1004826, EPI_ISL_1004827, EPI_ISL_1004828, EPI_ISL_1004829, EPI_ISL_1004830, EPI_ISL_1004831, EPI_ISL_1004832, EPI_ISL_1004833, EPI_ISL_1004834, EPI_ISL_1004835, EPI_ISL_1004836, EPI_ISL_1004837, EPI_ISL_1004838, EPI_ISL_1004839, EPI_ISL_1004840, EPI_ISL_1004841, EPI_ISL_1004842, EPI_ISL_1004843, EPI_ISL_1004844, EPI_ISL_1004845, EPI_ISL_1004846, EPI_ISL_1004847, EPI_ISL_1004848, EPI_ISL_1004849, EPI_ISL_1004850, EPI_ISL_1004851, EPI_ISL_1004852, EPI_ISL_1004853, EPI_ISL_1004854, EPI_ISL_1004855, EPI_ISL_1004856, EPI_ISL_1004857, EPI_ISL_1004858, EPI_ISL_1004859, EPI_ISL_1004860, EPI_ISL_1004861, EPI_ISL_1004862, EPI_ISL_1004863, EPI_ISL_1004864, EPI_ISL_1004865, EPI_ISL_1004866, EPI_ISL_1004867, EPI_ISL_1004868, EPI_ISL_1004869, EPI_ISL_1004870, EPI_ISL_1004871, EPI_ISL_1004872, EPI_ISL_1004873, EPI_ISL_1004874, EPI_ISL_1004898, EPI_ISL_1004899, EPI_ISL_1004900, EPI_ISL_1004901, EPI_ISL_1004902, EPI_ISL_1004903, EPI_ISL_1004904, EPI_ISL_1004905, EPI_ISL_1004906, EPI_ISL_1004907, EPI_ISL_1004908, EPI_ISL_1004909, EPI_ISL_1004910, EPI_ISL_1004911, EPI_ISL_1004912, EPI_ISL_1004913, EPI_ISL_1004914, EPI_ISL_1004915, EPI_ISL_1004916, EPI_ISL_1004917, EPI_ISL_1004918, EPI_ISL_1004919, EPI_ISL_1004920, EPI_ISL_1004921, EPI_ISL_1004922, EPI_ISL_1004923, EPI_ISL_1004924, EPI_ISL_1004925, EPI_ISL_1004926, EPI_ISL_1004927, EPI_ISL_1004928, EPI_ISL_1004929, EPI_ISL_1004930, EPI_ISL_1004931, EPI_ISL_1004932, EPI_ISL_1004933, EPI_ISL_1004934, EPI_ISL_1004935, EPI_ISL_1004936, EPI_ISL_1004937, EPI_ISL_1004938, EPI_ISL_1004939, EPI_ISL_1004940, EPI_ISL_1004941, EPI_ISL_1004942, EPI_ISL_1004943, EPI_ISL_1004944, EPI_ISL_1004945, EPI_ISL_1004946, EPI_ISL_1004947, EPI_ISL_1004948, EPI_ISL_1004949, EPI_ISL_1004950, EPI_ISL_1004951, EPI_ISL_1004952, EPI_ISL_1004953, EPI_ISL_1004954, EPI_ISL_1004955, EPI_ISL_1004956, EPI_ISL_1004957, EPI_ISL_1004958, EPI_ISL_1004959, EPI_ISL_1004960, EPI_ISL_1004961, EPI_ISL_1004962, EPI_ISL_1004964, EPI_ISL_1004965, EPI_ISL_1004966, EPI_ISL_1004967, EPI_ISL_1004968, EPI_ISL_1004969, EPI_ISL_1004970, EPI_ISL_1004971, EPI_ISL_1004972, EPI_ISL_1004973, EPI_ISL_1004974, EPI_ISL_1004975			
see above	Viollier AG	Department of Biosystems Science and Engineering, ETH Zürich	Chaoran Chen, Sarah Nadeau, Ivan Topolsky, Emmanouil Dermitzakis, Keith Harshman, Ioannis Xenarios, Henri Pegeot, Lorenzo Cerutti, Deborah Penet, Philipp Jablonski, Lara Fuhrmann, David Dreifuss, Katharina Jahn, Christiane Beckmann, Maurice Redondo, Olivier Kobel, Christoph Noppen, Sophie Seidel, Noemie Santamaria de Souza, Niko Beerenwinkel, Tanja Stadler
EPI_ISL_1005007, EPI_ISL_1005008, EPI_ISL_1005009, EPI_ISL_1005010, EPI_ISL_1005011, EPI_ISL_1005038, EPI_ISL_1005049, EPI_ISL_1005050, EPI_ISL_1005051, EPI_ISL_1005052, EPI_ISL_1005053, EPI_ISL_1005071, EPI_ISL_1005072, EPI_ISL_1005073, EPI_ISL_1005074, EPI_ISL_1005075, EPI_ISL_1005097, EPI_ISL_1005106, EPI_ISL_1005107, EPI_ISL_1005108, EPI_ISL_1005109, EPI_ISL_1005110, EPI_ISL_1005167, EPI_ISL_1005179, EPI_ISL_1005180, EPI_ISL_1005181, EPI_ISL_1005182, EPI_ISL_1005183, EPI_ISL_1005196, EPI_ISL_1005197, EPI_ISL_1005198, EPI_ISL_1005202, EPI_ISL_1005213, EPI_ISL_1005214, EPI_ISL_1005215, EPI_ISL_1005216, EPI_ISL_1005217			
see above	Viollier AG	Department of Biosystems Science and Engineering, ETH Zürich	Chaoran Chen, Sarah Nadeau, Catharine Aquino, Ivan Topolsky, Philipp Jablonski, Lara Fuhrmann, David Dreifuss, Katharina Jahn, Andreia Cabral de Gouvea, Maria Domenica Moccia, Simon Grüter, Timothy Sykes, Lennart Opitz, Griffin White, Laura Neff, Doris Popovic, Andrea Patrignani, Jay Tracy, Ralph Schlapbach, Christiane Beckmann, Maurice Redondo, Olivier Kobel, Christoph Noppen, Sophie Seidel, Noemie Santamaria de Souza, Niko Beerenwinkel, Tanja Stadler
EPI_ISL_1007614, EPI_ISL_1007615	CHUV	Laboratory of genomics and metagenomics, Institute of Microbiology, University Hospital Centre and University of Lausanne, Switzerland	Trestan Pillonel, Damien Jacot, Sébastien Aeby, Gilbert Greub, Claire Bertelli
EPI_ISL_1036105, EPI_ISL_1036106, EPI_ISL_1036107, EPI_ISL_1036108, EPI_ISL_1036109, EPI_ISL_1036110, EPI_ISL_1036111, EPI_ISL_1036112, EPI_ISL_1036114, EPI_ISL_1036115, EPI_ISL_1036117, EPI_ISL_1036118, EPI_ISL_1036119, EPI_ISL_1036120, EPI_ISL_1036121, EPI_ISL_1036122, EPI_ISL_1036123, EPI_ISL_1036124, EPI_ISL_1036125, EPI_ISL_1036128, EPI_ISL_1036129, EPI_ISL_1036131, EPI_ISL_1036132, EPI_ISL_1036133, EPI_ISL_1036134, EPI_ISL_1036136, EPI_ISL_1036138, EPI_ISL_1036139			
see above	Labormedizinisches Zentrum Dr Risch	University Hospital Basel, Clinical Bacteriology	Tim Roloff, Madlen Stange, Helena MB Seth-Smith, Alfredo Mari, Karoline Leuzinger, Julia Bielicki, Nadia Wohlwend,Martin Risch, Lorenz Risch, Manuel Battegay, Hans Hirsch, Adrian Egli
EPI_ISL_1119024, EPI_ISL_1119025, EPI_ISL_1119026, EPI_ISL_1119027, EPI_ISL_1119028, EPI_ISL_1119029	Viollier AG	Department of Biosystems Science and Engineering, ETH Zürich	Chaoran Chen, Sarah Nadeau, Catharine Aquino, Ivan Topolsky, Philipp Jablonski, Lara Fuhrmann, David Dreifuss, Katharina Jahn, Andreia Cabral de Gouvea, Maria Domenica Moccia, Simon Grüter, Timothy Sykes, Lennart Opitz, Griffin White, Laura Neff, Doris Popovic, Andrea Patrignani, Jay Tracy, Ralph Schlapbach, Christiane Beckmann, Maurice Redondo, Olivier Kobel, Christoph Noppen, Sophie Seidel, Noemie Santamaria de Souza, Niko Beerenwinkel, Tanja Stadler
EPI_ISL_1119041, EPI_ISL_1119042, EPI_ISL_1119043, EPI_ISL_1119044, EPI_ISL_1119045, EPI_ISL_1119046, EPI_ISL_1119047, EPI_ISL_1119048, EPI_ISL_1119049, EPI_ISL_1119050, EPI_ISL_1119202, EPI_ISL_1119222			
see above	Viollier AG	Department of Biosystems Science and Engineering, ETH Zürich	Chaoran Chen, Sarah Nadeau, Ivan Topolsky, Emmanouil Dermitzakis, Keith Harshman, Ioannis Xenarios, Henri Pegeot, Lorenzo Cerutti, Deborah Penet, Philipp Jablonski, Lara Fuhrmann, David Dreifuss, Katharina Jahn, Christiane Beckmann, Maurice Redondo, Olivier Kobel, Christoph Noppen, Sophie Seidel, Noemie Santamaria de Souza, Niko Beerenwinkel, Tanja Stadler
EPI_ISL_1119234, EPI_ISL_1119235, EPI_ISL_1119236, EPI_ISL_1119237	Viollier AG	Department of Biosystems Science and Engineering, ETH Zürich	Chaoran Chen, Sarah Nadeau, Catharine Aquino, Ivan Topolsky, Philipp Jablonski, Lara Fuhrmann, David Dreifuss, Katharina Jahn, Andreia Cabral de Gouvea, Maria Domenica Moccia, Simon Grüter, Timothy Sykes, Lennart Opitz, Griffin White, Laura Neff, Doris Popovic, Andrea Patrignani, Jay Tracy, Ralph Schlapbach, Christiane Beckmann, Maurice Redondo, Olivier Kobel, Christoph Noppen, Sophie Seidel, Noemie Santamaria de Souza, Niko Beerenwinkel, Tanja Stadler
EPI_ISL_1119238	Viollier AG	Department of Biosystems Science and Engineering, ETH Zürich	Chaoran Chen, Sarah Nadeau, Ivan Topolsky, Emmanouil Dermitzakis, Keith Harshman, Ioannis Xenarios, Henri Pegeot, Lorenzo Cerutti, Deborah Penet, Philipp Jablonski, Lara Fuhrmann, David Dreifuss, Katharina Jahn, Christiane Beckmann, Maurice Redondo, Olivier Kobel, Christoph Noppen, Sophie Seidel, Noemie Santamaria de Souza, Niko Beerenwinkel, Tanja Stadler
EPI_ISL_1119241	Viollier AG	Department of Biosystems Science and Engineering, ETH Zürich	Chaoran Chen, Sarah Nadeau, Catharine Aquino, Ivan Topolsky, Philipp Jablonski, Lara Fuhrmann, David Dreifuss, Katharina Jahn, Andreia Cabral de Gouvea, Maria Domenica Moccia, Simon Grüter, Timothy Sykes, Lennart Opitz, Griffin White, Laura Neff, Doris Popovic, Andrea Patrignani, Jay Tracy, Ralph Schlapbach, Christiane Beckmann, Maurice Redondo, Olivier Kobel, Christoph Noppen, Sophie Seidel, Noemie Santamaria de Souza, Niko Beerenwinkel, Tanja Stadler
EPI_ISL_1119244	Viollier AG	Department of Biosystems Science and Engineering, ETH Zürich	Chaoran Chen, Sarah Nadeau, Ivan Topolsky, Emmanouil Dermitzakis, Keith Harshman, Ioannis Xenarios, Henri Pegeot, Lorenzo Cerutti, Deborah Penet, Philipp Jablonski, Lara Fuhrmann, David Dreifuss, Katharina Jahn, Christiane Beckmann, Maurice Redondo, Olivier Kobel, Christoph Noppen, Sophie Seidel, Noemie Santamaria de Souza, Niko Beerenwinkel, Tanja Stadler
EPI_ISL_1119340, EPI_ISL_1119341, EPI_ISL_1119342, EPI_ISL_1119343, EPI_ISL_1119348, EPI_ISL_1119349, EPI_ISL_1119350	Viollier AG	Department of Biosystems Science and Engineering, ETH Zürich	Chaoran Chen, Sarah Nadeau, Catharine Aquino, Ivan Topolsky, Philipp Jablonski, Lara Fuhrmann, David Dreifuss, Katharina Jahn, Andreia Cabral de Gouvea, Maria Domenica Moccia, Simon Grüter, Timothy Sykes, Lennart Opitz, Griffin White, Laura Neff, Doris Popovic, Andrea Patrignani, Jay Tracy, Ralph Schlapbach, Christiane Beckmann, Maurice Redondo, Olivier Kobel, Christoph Noppen, Sophie Seidel, Noemie Santamaria de Souza, Niko Beerenwinkel, Tanja Stadler
EPI_ISL_1119351	Viollier AG	Department of Biosystems Science and Engineering, ETH Zürich	Chaoran Chen, Sarah Nadeau, Ivan Topolsky, Emmanouil Dermitzakis, Keith Harshman, Ioannis Xenarios, Henri Pegeot, Lorenzo Cerutti, Deborah Penet, Philipp Jablonski, Lara Fuhrmann, David Dreifuss, Katharina Jahn, Christiane Beckmann, Maurice Redondo, Olivier Kobel, Christoph Noppen, Sophie Seidel, Noemie Santamaria de Souza, Niko Beerenwinkel, Tanja Stadler
EPI_ISL_1119367, EPI_ISL_1119369	Viollier AG	Department of Biosystems Science and Engineering, ETH Zürich	Chaoran Chen, Sarah Nadeau, Catharine Aquino, Ivan Topolsky, Philipp Jablonski, Lara Fuhrmann, David Dreifuss, Katharina Jahn, Andreia Cabral de Gouvea, Maria Domenica Moccia, Simon Grüter, Timothy Sykes, Lennart Opitz, Griffin White, Laura Neff, Doris Popovic, Andrea Patrignani, Jay Tracy, Ralph Schlapbach, Christiane Beckmann, Maurice Redondo, Olivier Kobel, Christoph Noppen, Sophie Seidel, Noemie Santamaria de Souza, Niko Beerenwinkel, Tanja Stadler
EPI_ISL_1119370	Viollier AG	Department of Biosystems Science and Engineering, ETH Zürich	Chaoran Chen, Sarah Nadeau, Ivan Topolsky, Emmanouil Dermitzakis, Keith Harshman, Ioannis Xenarios, Henri Pegeot, Lorenzo Cerutti, Deborah Penet, Philipp Jablonski, Lara Fuhrmann, David Dreifuss, Katharina Jahn, Christiane Beckmann, Maurice Redondo, Olivier Kobel, Christoph Noppen, Sophie Seidel, Noemie Santamaria de Souza, Niko Beerenwinkel, Tanja Stadler

We gratefully acknowledge the following Authors from the Originating laboratories responsible for obtaining the specimens, as well as the Submitting laboratories where the genome data were generated and shared via GISAID, on which this research is based.

All Submitters of data may be contacted directly via www.gisaid.org

Authors are sorted alphabetically.

Accession ID	Originating Laboratory	Submitting Laboratory	Authors
EPI_ISL_1001828	Viollier AG	Department of Biosystems Science and Engineering, ETH Zürich	Chaoran Chen, Sarah Nadeau, Catharine Aquino, Ivan Topolsky, Philipp Jablonski, Lara Fuhrmann, David Dreifuss, Katharina Jahn, Andreia Cabral de Gouvea, Maria Domenica Moccia, Simon Grüter, Timothy Sykes, Lennart Opitz, Griffin White, Laura Neff, Doris Popovic, Andrea Patrignani, Jay Tracy, Ralph Schlapbach, Christiane Beckmann, Maurice Redondo, Olivier Kobel, Christoph Noppen, Sophie Seidel, Noemie Santamaria de Souza, Niko Beerenwinkel, Tanja Stadler
EPI_ISL_1001832	Viollier AG	Department of Biosystems Science and Engineering, ETH Zürich	Christian Beisel, Sarah Nadeau, Chaoran Chen, Ivan Topolsky, Philipp Jablonski, Lara Fuhrmann, David Dreifuss, Katharina Jahn, Rebecca Denes, Ina Nissen, Natascha Santacroce, Elodie Burcklen, Christiane Beckmann, Maurice Redondo, Olivier Kobel, Christoph Noppen, Sophie Seidel, Noemie Santamaria de Souza, Niko Beerenwinkel, Tanja Stadler
EPI_ISL_1001833, EPI_ISL_1001834	Viollier AG	Department of Biosystems Science and Engineering, ETH Zürich	Chaoran Chen, Sarah Nadeau, Catharine Aquino, Ivan Topolsky, Philipp Jablonski, Lara Fuhrmann, David Dreifuss, Katharina Jahn, Andreia Cabral de Gouvea, Maria Domenica Moccia, Simon Grüter, Timothy Sykes, Lennart Opitz, Griffin White, Laura Neff, Doris Popovic, Andrea Patrignani, Jay Tracy, Ralph Schlapbach, Christiane Beckmann, Maurice Redondo, Olivier Kobel, Christoph Noppen, Sophie Seidel, Noemie Santamaria de Souza, Niko Beerenwinkel, Tanja Stadler
EPI_ISL_1001835	Viollier AG	Department of Biosystems Science and Engineering, ETH Zürich	Christian Beisel, Sarah Nadeau, Chaoran Chen, Ivan Topolsky, Philipp Jablonski, Lara Fuhrmann, David Dreifuss, Katharina Jahn, Rebecca Denes, Ina Nissen, Natascha Santacroce, Elodie Burcklen, Christiane Beckmann, Maurice Redondo, Olivier Kobel, Christoph Noppen, Sophie Seidel, Noemie Santamaria de Souza, Niko Beerenwinkel, Tanja Stadler
EPI_ISL_1001836, EPI_ISL_1001837, EPI_ISL_1001838, EPI_ISL_1001839, EPI_ISL_1001840, EPI_ISL_1001841, EPI_ISL_1001842, EPI_ISL_1001843, EPI_ISL_1001844, EPI_ISL_1001845, EPI_ISL_1001846, EPI_ISL_1001847, EPI_ISL_1001848, EPI_ISL_1001849, EPI_ISL_1001850			
see above	Viollier AG	Department of Biosystems Science and Engineering, ETH Zürich	Chaoran Chen, Sarah Nadeau, Catharine Aquino, Ivan Topolsky, Philipp Jablonski, Lara Fuhrmann, David Dreifuss, Katharina Jahn, Andreia Cabral de Gouvea, Maria Domenica Moccia, Simon Grüter, Timothy Sykes, Lennart Opitz, Griffin White, Laura Neff, Doris Popovic, Andrea Patrignani, Jay Tracy, Ralph Schlapbach, Christiane Beckmann, Maurice Redondo, Olivier Kobel, Christoph Noppen, Sophie Seidel, Noemie Santamaria de Souza, Niko Beerenwinkel, Tanja Stadler
EPI_ISL_1001851, EPI_ISL_1001852	Viollier AG	Department of Biosystems Science and Engineering, ETH Zürich	Christian Beisel, Sarah Nadeau, Chaoran Chen, Ivan Topolsky, Philipp Jablonski, Lara Fuhrmann, David Dreifuss, Katharina Jahn, Rebecca Denes, Ina Nissen, Natascha Santacroce, Elodie Burcklen, Christiane Beckmann, Maurice Redondo, Olivier Kobel, Christoph Noppen, Sophie Seidel, Noemie Santamaria de Souza, Niko Beerenwinkel, Tanja Stadler
EPI_ISL_1001853, EPI_ISL_1001854, EPI_ISL_1001856, EPI_ISL_1001857, EPI_ISL_1001858, EPI_ISL_1001859, EPI_ISL_1001860, EPI_ISL_1001861	Viollier AG	Department of Biosystems Science and Engineering, ETH Zürich	Chaoran Chen, Sarah Nadeau, Catharine Aquino, Ivan Topolsky, Philipp Jablonski, Lara Fuhrmann, David Dreifuss, Katharina Jahn, Andreia Cabral de Gouvea, Maria Domenica Moccia, Simon Grüter, Timothy Sykes, Lennart Opitz, Griffin White, Laura Neff, Doris Popovic, Andrea Patrignani, Jay Tracy, Ralph Schlapbach, Christiane Beckmann, Maurice Redondo, Olivier Kobel, Christoph Noppen, Sophie Seidel, Noemie Santamaria de Souza, Niko Beerenwinkel, Tanja Stadler
EPI_ISL_1001862	Viollier AG	Department of Biosystems Science and Engineering, ETH Zürich	Christian Beisel, Sarah Nadeau, Chaoran Chen, Ivan Topolsky, Philipp Jablonski, Lara Fuhrmann, David Dreifuss, Katharina Jahn, Rebecca Denes, Ina Nissen, Natascha Santacroce, Elodie Burcklen, Christiane Beckmann, Maurice Redondo, Olivier Kobel, Christoph Noppen, Sophie Seidel, Noemie Santamaria de Souza, Niko Beerenwinkel, Tanja Stadler
EPI_ISL_1001863, EPI_ISL_1001864	Viollier AG	Department of Biosystems Science and Engineering, ETH Zürich	Chaoran Chen, Sarah Nadeau, Catharine Aquino, Ivan Topolsky, Philipp Jablonski, Lara Fuhrmann, David Dreifuss, Katharina Jahn, Andreia Cabral de Gouvea, Maria Domenica Moccia, Simon Grüter, Timothy Sykes, Lennart Opitz, Griffin White, Laura Neff, Doris Popovic, Andrea Patrignani, Jay Tracy, Ralph Schlapbach, Christiane Beckmann, Maurice Redondo, Olivier Kobel, Christoph Noppen, Sophie Seidel, Noemie Santamaria de Souza, Niko Beerenwinkel, Tanja Stadler
EPI_ISL_1001865	Viollier AG	Department of Biosystems Science and Engineering, ETH Zürich	Christian Beisel, Sarah Nadeau, Chaoran Chen, Ivan Topolsky, Philipp Jablonski, Lara Fuhrmann, David Dreifuss, Katharina Jahn, Rebecca Denes, Ina Nissen, Natascha Santacroce, Elodie Burcklen, Christiane Beckmann, Maurice Redondo, Olivier Kobel, Christoph Noppen, Sophie Seidel, Noemie Santamaria de Souza, Niko Beerenwinkel, Tanja Stadler
EPI_ISL_1001866, EPI_ISL_1001867, EPI_ISL_1001868	Viollier AG	Department of Biosystems Science and Engineering, ETH Zürich	Chaoran Chen, Sarah Nadeau, Catharine Aquino, Ivan Topolsky, Philipp Jablonski, Lara Fuhrmann, David Dreifuss, Katharina Jahn, Andreia Cabral de Gouvea, Maria Domenica Moccia, Simon Grüter, Timothy Sykes, Lennart Opitz, Griffin White, Laura Neff, Doris Popovic, Andrea Patrignani, Jay Tracy, Ralph Schlapbach, Christiane Beckmann, Maurice Redondo, Olivier Kobel, Christoph Noppen, Sophie Seidel, Noemie Santamaria de Souza, Niko Beerenwinkel, Tanja Stadler
EPI_ISL_1001869	Viollier AG	Department of Biosystems Science and Engineering, ETH Zürich	Christian Beisel, Sarah Nadeau, Chaoran Chen, Ivan Topolsky, Philipp Jablonski, Lara Fuhrmann, David Dreifuss, Katharina Jahn, Rebecca Denes, Ina Nissen, Natascha Santacroce, Elodie Burcklen, Christiane Beckmann, Maurice Redondo, Olivier Kobel, Christoph Noppen, Sophie Seidel, Noemie Santamaria de Souza, Niko Beerenwinkel, Tanja Stadler
EPI_ISL_1001870, EPI_ISL_1001871, EPI_ISL_1001872, EPI_ISL_1001873, EPI_ISL_1001874, EPI_ISL_1001875, EPI_ISL_1001876, EPI_ISL_1001877, EPI_ISL_1001878, EPI_ISL_1001882, EPI_ISL_1001883, EPI_ISL_1001884, EPI_ISL_1001885, EPI_ISL_1001886, EPI_ISL_1001887, EPI_ISL_1001888, EPI_ISL_1001889, EPI_ISL_1001890, EPI_ISL_1001891, EPI_ISL_1001892, EPI_ISL_1002193, EPI_ISL_1002194, EPI_ISL_1002195, EPI_ISL_1002196, EPI_ISL_1002197, EPI_ISL_1002198, EPI_ISL_1002199			
see above	Viollier AG	Department of Biosystems Science and Engineering, ETH Zürich	Chaoran Chen, Sarah Nadeau, Catharine Aquino, Ivan Topolsky, Philipp Jablonski, Lara Fuhrmann, David Dreifuss, Katharina Jahn, Andreia Cabral de Gouvea, Maria Domenica Moccia, Simon Grüter, Timothy Sykes, Lennart Opitz, Griffin White, Laura Neff, Doris Popovic, Andrea Patrignani, Jay Tracy, Ralph Schlapbach, Christiane Beckmann, Maurice Redondo, Olivier Kobel, Christoph Noppen, Sophie Seidel, Noemie Santamaria de Souza, Niko Beerenwinkel, Tanja Stadler
EPI_ISL_1002200	Viollier AG	Department of Biosystems Science and Engineering, ETH Zürich	Christian Beisel, Sarah Nadeau, Chaoran Chen, Ivan Topolsky, Philipp Jablonski, Lara Fuhrmann, David Dreifuss, Katharina Jahn, Rebecca Denes, Ina Nissen, Natascha Santacroce, Elodie Burcklen, Christiane Beckmann, Maurice Redondo, Olivier Kobel, Christoph Noppen, Sophie Seidel, Noemie Santamaria de Souza, Niko Beerenwinkel, Tanja Stadler
EPI_ISL_1002201, EPI_ISL_1002202, EPI_ISL_1002203, EPI_ISL_1002204, EPI_ISL_1002205, EPI_ISL_1002206, EPI_ISL_1002207	Viollier AG	Department of Biosystems Science and Engineering, ETH Zürich	Chaoran Chen, Sarah Nadeau, Catharine Aquino, Ivan Topolsky, Philipp Jablonski, Lara Fuhrmann, David Dreifuss, Katharina Jahn, Andreia Cabral de Gouvea, Maria Domenica Moccia, Simon Grüter, Timothy Sykes, Lennart Opitz, Griffin White, Laura Neff, Doris Popovic, Andrea Patrignani, Jay Tracy, Ralph Schlapbach, Christiane Beckmann, Maurice Redondo, Olivier Kobel, Christoph Noppen, Sophie Seidel, Noemie Santamaria de Souza, Niko Beerenwinkel, Tanja Stadler
EPI_ISL_1002208, EPI_ISL_1002211, EPI_ISL_1002212, EPI_ISL_1002213, EPI_ISL_1002214, EPI_ISL_1002215, EPI_ISL_1002216	Viollier AG	Department of Biosystems Science and Engineering, ETH Zürich	Christian Beisel, Sarah Nadeau, Chaoran Chen, Ivan Topolsky, Philipp Jablonski, Lara Fuhrmann, David Dreifuss, Katharina Jahn, Rebecca Denes, Ina Nissen, Natascha Santacroce, Elodie Burcklen, Christiane Beckmann, Maurice Redondo, Olivier Kobel, Christoph Noppen, Sophie Seidel, Noemie Santamaria de Souza, Niko Beerenwinkel, Tanja Stadler
EPI_ISL_1002217, EPI_ISL_1002218, EPI_ISL_1002219, EPI_ISL_1002220, EPI_ISL_1002221, EPI_ISL_1002222, EPI_ISL_1002223, EPI_ISL_1002224, EPI_ISL_1002225, EPI_ISL_1002226, EPI_ISL_1002227, EPI_ISL_1002228, EPI_ISL_1002229, EPI_ISL_1002230, EPI_ISL_1002231, EPI_ISL_1002232, EPI_ISL_1002233, EPI_ISL_1002234, EPI_ISL_1002235, EPI_ISL_1002236, EPI_ISL_1002237, EPI_ISL_1002238, EPI_ISL_1002239, EPI_ISL_1002240, EPI_ISL_1002241, EPI_ISL_1002242, EPI_ISL_1002243, EPI_ISL_1002244, EPI_ISL_1002245, EPI_ISL_1002246			
see above	Viollier AG	Department of Biosystems Science and Engineering, ETH Zürich	Chaoran Chen, Sarah Nadeau, Catharine Aquino, Ivan Topolsky, Philipp Jablonski, Lara Fuhrmann, David Dreifuss, Katharina Jahn, Andreia Cabral de Gouvea, Maria Domenica Moccia, Simon Grüter, Timothy Sykes, Lennart Opitz, Griffin White, Laura Neff, Doris Popovic, Andrea Patrignani, Jay Tracy, Ralph Schlapbach, Christiane Beckmann, Maurice Redondo, Olivier Kobel, Christoph Noppen, Sophie Seidel, Noemie Santamaria de Souza, Niko Beerenwinkel, Tanja Stadler

We gratefully acknowledge the following Authors from the Originating laboratories responsible for obtaining the specimens, as well as the Submitting laboratories where the genome data were generated and shared via GISAID, on which this research is based.

All Submitters of data may be contacted directly via www.gisaid.org

Authors are sorted alphabetically.

Accession ID	Originating Laboratory	Submitting Laboratory	Authors
EPI_ISL_1228828, EPI_ISL_1228829, EPI_ISL_1228830, EPI_ISL_1228831, EPI_ISL_1228832, EPI_ISL_1228833, EPI_ISL_1228834, EPI_ISL_1228835, EPI_ISL_1228836, EPI_ISL_1228837, EPI_ISL_1228854, EPI_ISL_1228855, EPI_ISL_1228856, EPI_ISL_1228857, EPI_ISL_1228858, EPI_ISL_1228859, EPI_ISL_1228860, EPI_ISL_1228861, EPI_ISL_1228862, EPI_ISL_1228863, EPI_ISL_1228864, EPI_ISL_1228866, EPI_ISL_1228867, EPI_ISL_1228868, EPI_ISL_1228870, EPI_ISL_1228871, EPI_ISL_1228872, EPI_ISL_1228873, EPI_ISL_1228874, EPI_ISL_1228875, EPI_ISL_1228876, EPI_ISL_1228877, EPI_ISL_1228878, EPI_ISL_1228879, EPI_ISL_1228880, EPI_ISL_1228881, EPI_ISL_1228882, EPI_ISL_1228884, EPI_ISL_1228885, EPI_ISL_1228886, EPI_ISL_1228887, EPI_ISL_1228888, EPI_ISL_1228889, EPI_ISL_1228890, EPI_ISL_1228891, EPI_ISL_1228892, EPI_ISL_1228893, EPI_ISL_1228894, EPI_ISL_1228895, EPI_ISL_1228896, EPI_ISL_1228898, EPI_ISL_1228899, EPI_ISL_1228900, EPI_ISL_1228901, EPI_ISL_1228902, EPI_ISL_1228903, EPI_ISL_1228904, EPI_ISL_1228906, EPI_ISL_1228907			
see above	University Hospitals of Geneva, Laboratory of Virology	HUG, Laboratory of Virology and the Health2030 Genome Center	Samuel Cordey, Ana Rita Goncalves, Laurent Kaiser, Lorenzo Cerutti, Henri Pegeot, Melyssa Elies, Deborah Penet, Keith Harshman, Ioannis Xenarios, Emmanouil Dermitzakis
EPI_ISL_1259448, EPI_ISL_1259610, EPI_ISL_1259613, EPI_ISL_1259622, EPI_ISL_1259626, EPI_ISL_1259641, EPI_ISL_1259643, EPI_ISL_1259644, EPI_ISL_1259658, EPI_ISL_1259659, EPI_ISL_1259670, EPI_ISL_1259675, EPI_ISL_1259678, EPI_ISL_1259706, EPI_ISL_1259712, EPI_ISL_1259723, EPI_ISL_1259730, EPI_ISL_1259731, EPI_ISL_1259732, EPI_ISL_1259733, EPI_ISL_1259739, EPI_ISL_1259743, EPI_ISL_1259744, EPI_ISL_1259759, EPI_ISL_1260010, EPI_ISL_1260092, EPI_ISL_1260390, EPI_ISL_1260396, EPI_ISL_1260402, EPI_ISL_1260412, EPI_ISL_1260448			
see above	Viollier AG	Department of Biosystems Science and Engineering, ETH Zürich	Chaoran Chen, Sarah Nadeau, Ivan Topolsky, Emmanouil Dermitzakis, Keith Harshman, Ioannis Xenarios, Henri Pegeot, Lorenzo Cerutti, Deborah Penet, Philipp Jablonski, Lara Fuhrmann, David Dreifuss, Katharina Jahn, Christiane Beckmann, Maurice Redondo, Olivier Kobel, Christoph Noppen, Sophie Seidel, Noemie Santamaria de Souza, Niko Beerenwinkel, Tanja Stadler
EPI_ISL_1295984, EPI_ISL_1295985, EPI_ISL_1295986, EPI_ISL_1295987, EPI_ISL_1295988, EPI_ISL_1295989, EPI_ISL_1295990, EPI_ISL_1295991, EPI_ISL_1295992, EPI_ISL_1295993, EPI_ISL_1295994, EPI_ISL_1295995, EPI_ISL_1295996, EPI_ISL_1295997, EPI_ISL_1295998, EPI_ISL_1295999, EPI_ISL_1296000, EPI_ISL_1296001, EPI_ISL_1296002, EPI_ISL_1296003, EPI_ISL_1296004, EPI_ISL_1296005, EPI_ISL_1296006, EPI_ISL_1296007, EPI_ISL_1296008, EPI_ISL_1296009, EPI_ISL_1296010, EPI_ISL_1296011, EPI_ISL_1296012, EPI_ISL_1296013, EPI_ISL_1296014, EPI_ISL_1296015, EPI_ISL_1296016, EPI_ISL_1296017, EPI_ISL_1296018, EPI_ISL_1296019, EPI_ISL_1296020, EPI_ISL_1296021, EPI_ISL_1296022, EPI_ISL_1296023, EPI_ISL_1296024, EPI_ISL_1296025, EPI_ISL_1296026, EPI_ISL_1296027, EPI_ISL_1296028, EPI_ISL_1296029, EPI_ISL_1296030, EPI_ISL_1296031, EPI_ISL_1296032, EPI_ISL_1296033, EPI_ISL_1296034, EPI_ISL_1296035, EPI_ISL_1296036, EPI_ISL_1296037, EPI_ISL_1296038, EPI_ISL_1296039, EPI_ISL_1296040, EPI_ISL_1296041, EPI_ISL_1296042, EPI_ISL_1296043, EPI_ISL_1296044, EPI_ISL_1296045, EPI_ISL_1296046, EPI_ISL_1296047, EPI_ISL_1296048, EPI_ISL_1296049, EPI_ISL_1296050, EPI_ISL_1296051, EPI_ISL_1296052, EPI_ISL_1296053, EPI_ISL_1296054, EPI_ISL_1296055, EPI_ISL_1296056, EPI_ISL_1296057, EPI_ISL_1296058, EPI_ISL_1296059, EPI_ISL_1296060, EPI_ISL_1296061, EPI_ISL_1296062, EPI_ISL_1296063, EPI_ISL_1296064, EPI_ISL_1296065, EPI_ISL_1296066, EPI_ISL_1296067, EPI_ISL_1296068, EPI_ISL_1296069, EPI_ISL_1296070, EPI_ISL_1296071, EPI_ISL_1296072, EPI_ISL_1296073, EPI_ISL_1296074, EPI_ISL_1296075, EPI_ISL_1296076, EPI_ISL_1296077, EPI_ISL_1296078, EPI_ISL_1296079, EPI_ISL_1296080, EPI_ISL_1296081, EPI_ISL_1296082, EPI_ISL_1296083, EPI_ISL_1296084, EPI_ISL_1296085, EPI_ISL_1296086, EPI_ISL_1296087, EPI_ISL_1296088, EPI_ISL_1296089, EPI_ISL_1296090, EPI_ISL_1296091, EPI_ISL_1296092, EPI_ISL_1296093, EPI_ISL_1296094, EPI_ISL_1296095, EPI_ISL_1296096, EPI_ISL_1296097, EPI_ISL_1296098, EPI_ISL_1296099, EPI_ISL_1296100, EPI_ISL_1296101, EPI_ISL_1296102, EPI_ISL_1296103, EPI_ISL_1296104, EPI_ISL_1296105, EPI_ISL_1296106, EPI_ISL_1296107, EPI_ISL_1296108, EPI_ISL_1296109, EPI_ISL_1296110, EPI_ISL_1296111, EPI_ISL_1296112, EPI_ISL_1296113, EPI_ISL_1296114, EPI_ISL_1296115, EPI_ISL_1296116, EPI_ISL_1296117, EPI_ISL_1296118, EPI_ISL_1296119, EPI_ISL_1296120, EPI_ISL_1296121, EPI_ISL_1296122, EPI_ISL_1296123, EPI_ISL_1296124, EPI_ISL_1296125, EPI_ISL_1296126, EPI_ISL_1296127, EPI_ISL_1296128, EPI_ISL_1296129, EPI_ISL_1296130, EPI_ISL_1296131, EPI_ISL_1296132, EPI_ISL_1296133, EPI_ISL_1296134, EPI_ISL_1296135, EPI_ISL_1296136, EPI_ISL_1296137, EPI_ISL_1296138, EPI_ISL_1296139, EPI_ISL_1296140, EPI_ISL_1296141, EPI_ISL_1296142, EPI_ISL_1296143, EPI_ISL_1296144, EPI_ISL_1296145, EPI_ISL_1296146, EPI_ISL_1296147, EPI_ISL_1296148, EPI_ISL_1296149, EPI_ISL_1296150, EPI_ISL_1296151, EPI_ISL_1296152, EPI_ISL_1296153, EPI_ISL_1296154, EPI_ISL_1296155, EPI_ISL_1296156, EPI_ISL_1296157, EPI_ISL_1296158, EPI_ISL_1296159, EPI_ISL_1296160, EPI_ISL_1296161, EPI_ISL_1296162, EPI_ISL_1296163, EPI_ISL_1296164, EPI_ISL_1296165, EPI_ISL_1296166, EPI_ISL_1296167, EPI_ISL_1296168, EPI_ISL_1296169, EPI_ISL_1296170, EPI_ISL_1296171, EPI_ISL_1296172, EPI_ISL_1296173, EPI_ISL_1296174, EPI_ISL_1296175, EPI_ISL_1296176, EPI_ISL_1296177, EPI_ISL_1296178, EPI_ISL_1296179, EPI_ISL_1296180, EPI_ISL_1296181, EPI_ISL_1296182, EPI_ISL_1296183, EPI_ISL_1296184, EPI_ISL_1296185, EPI_ISL_1296186, EPI_ISL_1296187, EPI_ISL_1296188, EPI_ISL_1296189, EPI_ISL_1296190, EPI_ISL_1296191, EPI_ISL_1296192, EPI_ISL_1296193, EPI_ISL_1296194, EPI_ISL_1296195, EPI_ISL_1296196, EPI_ISL_1296197, EPI_ISL_1296198, EPI_ISL_1296199, EPI_ISL_1296200, EPI_ISL_1296201, EPI_ISL_1296202, EPI_ISL_1296203, EPI_ISL_1296204, EPI_ISL_1296205, EPI_ISL_1296206, EPI_ISL_1296207, EPI_ISL_1296208, EPI_ISL_1296209, EPI_ISL_1296210, EPI_ISL_1296211, EPI_ISL_1296212, EPI_ISL_1296213, EPI_ISL_1296214, EPI_ISL_1296215, EPI_ISL_1296216, EPI_ISL_1296217, EPI_ISL_1296218, EPI_ISL_1296219, EPI_ISL_1296220, EPI_ISL_1296221, EPI_ISL_1296222, EPI_ISL_1296223, EPI_ISL_1296224, EPI_ISL_1296225, EPI_ISL_1296226, EPI_ISL_1296227, EPI_ISL_1296228, EPI_ISL_1296229, EPI_ISL_1296230, EPI_ISL_1296231, EPI_ISL_1296232, EPI_ISL_1296233			
see above	University Hospitals of Geneva, Laboratory of Virology	HUG, Laboratory of Virology and the Health2030 Genome Center	Samuel Cordey, Ana Rita Goncalves, Laurent Kaiser, Lorenzo Cerutti, Henri Pegeot, Melyssa Elies, Deborah Penet, Keith Harshman, Ioannis Xenarios, Emmanouil Dermitzakis
EPI_ISL_1302990, EPI_ISL_1302992, EPI_ISL_1302993, EPI_ISL_1302994, EPI_ISL_1302995, EPI_ISL_1302996, EPI_ISL_1302997, EPI_ISL_1302998, EPI_ISL_1302999, EPI_ISL_1303000, EPI_ISL_1303001, EPI_ISL_1303002, EPI_ISL_1303003, EPI_ISL_1303004, EPI_ISL_1303005, EPI_ISL_1303006, EPI_ISL_1303007, EPI_ISL_1303008, EPI_ISL_1303009, EPI_ISL_1303010, EPI_ISL_1303011, EPI_ISL_1303012, EPI_ISL_1303013, EPI_ISL_1303014, EPI_ISL_1303015, EPI_ISL_1303016, EPI_ISL_1303017, EPI_ISL_1303018, EPI_ISL_1303019, EPI_ISL_1303020, EPI_ISL_1303021, EPI_ISL_1303022, EPI_ISL_1303023, EPI_ISL_1303024, EPI_ISL_1303025, EPI_ISL_1303026, EPI_ISL_1303027, EPI_ISL_1303028, EPI_ISL_1303029, EPI_ISL_1303030			
see above	ADMED Microbiologie	Genomics and Transcriptomics, Philip Morris International	Reto Lienhard, Marie-Lise Tritten, Emmanuel Guedj, Nicolas Sierro, Rémi Dulize, David Bornand, Mehdi Auberson, Maxime Berthouzoz, Nikolai Ivanov, Manuel Peitsch
EPI_ISL_1360454	Viollier AG	Department of Biosystems Science and Engineering, ETH Zürich	Christian Beisel, Sarah Nadeau, Chaoran Chen, Ivan Topolsky, Philipp Jablonski, Lara Fuhrmann, David Dreifuss, Katharina Jahn, Rebecca Denes, Mirjam Feldkamp, Ina Nissen, Natascha Santacroce, Elodie Burcklen, Christiane Beckmann, Maurice Redondo, Olivier Kobel, Christoph Noppen, Sophie Seidel, Noemie Santamaria de Souza, Niko Beerenwinkel, Tanja Stadler
EPI_ISL_1360456	Viollier AG	Department of Biosystems Science and Engineering, ETH Zürich	Chaoran Chen, Sarah Nadeau, Catharine Aquino, Ivan Topolsky, Philipp Jablonski, Lara Fuhrmann, David Dreifuss, Katharina Jahn, Andreia Cabral de Gouvea, Maria Domenica Moccia, Simon Grüter, Timothy Sykes, Lennart Opitz, Griffin White, Laura Neff, Doris Popovic, Andrea Patrignani, Jay Tracy, Ralph Schlapbach, Christiane Beckmann, Maurice Redondo, Olivier Kobel, Christoph Noppen, Sophie Seidel, Noemie Santamaria de Souza, Niko Beerenwinkel, Tanja Stadler
EPI_ISL_1360458, EPI_ISL_1360461	Viollier AG	Department of Biosystems Science and Engineering, ETH Zürich	Chaoran Chen, Sarah Nadeau, Ivan Topolsky, Emmanouil Dermitzakis, Keith Harshman, Ioannis Xenarios, Henri Pegeot, Lorenzo Cerutti, Deborah Penet, Philipp Jablonski, Lara Fuhrmann, David Dreifuss, Katharina Jahn, Christiane Beckmann, Maurice Redondo, Olivier Kobel, Christoph Noppen, Sophie Seidel, Noemie Santamaria de Souza, Niko Beerenwinkel, Tanja Stadler
EPI_ISL_1360465	Viollier AG	Department of Biosystems Science and Engineering, ETH Zürich	Christian Beisel, Sarah Nadeau, Chaoran Chen, Ivan Topolsky, Philipp Jablonski, Lara Fuhrmann, David Dreifuss, Katharina Jahn, Rebecca Denes, Mirjam Feldkamp, Ina Nissen, Natascha Santacroce, Elodie Burcklen, Christiane Beckmann, Maurice Redondo, Olivier Kobel, Christoph Noppen, Sophie Seidel, Noemie Santamaria de Souza, Niko Beerenwinkel, Tanja Stadler
EPI_ISL_1360466	Viollier AG	Department of Biosystems Science and Engineering, ETH Zürich	Chaoran Chen, Sarah Nadeau, Catharine Aquino, Ivan Topolsky, Philipp Jablonski, Lara Fuhrmann, David Dreifuss, Katharina Jahn, Andreia Cabral de Gouvea, Maria Domenica Moccia, Simon Grüter, Timothy Sykes, Lennart Opitz, Griffin White, Laura Neff, Doris Popovic, Andrea Patrignani, Jay Tracy, Ralph Schlapbach, Christiane Beckmann, Maurice Redondo, Olivier Kobel, Christoph Noppen, Sophie Seidel, Noemie Santamaria de Souza, Niko Beerenwinkel, Tanja Stadler
EPI_ISL_1360467, EPI_ISL_1360472	Viollier AG	Department of Biosystems Science and Engineering, ETH Zürich	Christian Beisel, Sarah Nadeau, Chaoran Chen, Ivan Topolsky, Philipp Jablonski, Lara Fuhrmann, David Dreifuss, Katharina Jahn, Rebecca Denes, Mirjam Feldkamp, Ina Nissen, Natascha Santacroce, Elodie Burcklen, Christiane Beckmann, Maurice Redondo, Olivier Kobel, Christoph Noppen, Sophie Seidel, Noemie Santamaria de Souza, Niko Beerenwinkel, Tanja Stadler
EPI_ISL_1360473	Viollier AG	Department of Biosystems Science and Engineering, ETH Zürich	Chaoran Chen, Sarah Nadeau, Catharine Aquino, Ivan Topolsky, Philipp Jablonski, Lara Fuhrmann, David Dreifuss, Katharina Jahn, Andreia Cabral de Gouvea, Maria Domenica Moccia, Simon Grüter, Timothy Sykes, Lennart Opitz, Griffin White, Laura Neff, Doris Popovic, Andrea Patrignani, Jay Tracy, Ralph Schlapbach, Christiane Beckmann, Maurice Redondo, Olivier Kobel, Christoph Noppen, Sophie Seidel, Noemie Santamaria de Souza, Niko Beerenwinkel, Tanja Stadler
EPI_ISL_1360474	Viollier AG	Department of Biosystems Science and Engineering, ETH Zürich	Chaoran Chen, Sarah Nadeau, Ivan Topolsky, Emmanouil Dermitzakis, Keith Harshman, Ioannis Xenarios, Henri Pegeot, Lorenzo Cerutti, Deborah Penet, Philipp Jablonski, Lara Fuhrmann, David Dreifuss, Katharina Jahn, Christiane Beckmann, Maurice Redondo, Olivier Kobel, Christoph Noppen, Sophie Seidel, Noemie Santamaria de Souza, Niko Beerenwinkel, Tanja Stadler
EPI_ISL_1360477	Viollier AG	Department of Biosystems Science and Engineering, ETH Zürich	Christian Beisel, Sarah Nadeau, Chaoran Chen, Ivan Topolsky, Philipp Jablonski, Lara Fuhrmann, David Dreifuss, Katharina Jahn, Rebecca Denes, Mirjam Feldkamp, Ina Nissen, Natascha Santacroce, Elodie Burcklen, Christiane Beckmann, Maurice Redondo, Olivier Kobel, Christoph Noppen, Sophie Seidel, Noemie Santamaria de Souza, Niko Beerenwinkel, Tanja Stadler
EPI_ISL_1360479	Viollier AG	Department of Biosystems Science and Engineering, ETH Zürich	Chaoran Chen, Sarah Nadeau, Catharine Aquino, Ivan Topolsky, Philipp Jablonski, Lara Fuhrmann, David Dreifuss, Katharina Jahn, Andreia Cabral de Gouvea, Maria Domenica Moccia, Simon Grüter, Timothy Sykes, Lennart Opitz, Griffin White, Laura Neff, Doris Popovic, Andrea Patrignani, Jay Tracy, Ralph Schlapbach, Christiane Beckmann, Maurice Redondo, Olivier Kobel, Christoph Noppen, Sophie Seidel, Noemie Santamaria de Souza, Niko Beerenwinkel, Tanja Stadler

Victoria/AU

We gratefully acknowledge the following Authors from the Originating laboratories responsible for obtaining the specimens, as well as the Submitting laboratories where the genome data were generated and shared via GISAID, on which this research is based.

All Submitters of data may be contacted directly via www.gisaid.org

Authors are sorted alphabetically.

Accession ID	Originating Laboratory	Submitting Laboratory	Authors
EPI_ISL_1033125, EPI_ISL_1033128, EPI_ISL_1033129, EPI_ISL_1033130, EPI_ISL_1033136, EPI_ISL_1033137, EPI_ISL_1033138, EPI_ISL_1033139, EPI_ISL_1055266, EPI_ISL_1055268, EPI_ISL_1055269, EPI_ISL_1055271, EPI_ISL_1055283, EPI_ISL_1055284, EPI_ISL_1055285, EPI_ISL_1055287, EPI_ISL_1055288, EPI_ISL_1055289, EPI_ISL_1055290, EPI_ISL_1055291, EPI_ISL_1055292, EPI_ISL_1055293, EPI_ISL_1055294, EPI_ISL_1055295, EPI_ISL_1055296, EPI_ISL_1055300			
see above	Microbiological Diagnostic Unit - Public Health Laboratory (MDU-PHL)	MDU-PHL	Seemann T., Sait, M.L., Sherry, N.L.
EPI_ISL_1055301, EPI_ISL_1055302	Victorian Infectious Diseases Reference Laboratory (VIDRL)	VIDRL and MDU-PHL	Caly L., Seemann T., Sait, M.L., Druce J., Sherry, N.L.
EPI_ISL_1055303, EPI_ISL_1055304, EPI_ISL_1055305, EPI_ISL_1055306, EPI_ISL_1055308, EPI_ISL_1055310, EPI_ISL_1055311, EPI_ISL_1055312, EPI_ISL_1055313, EPI_ISL_1055314, EPI_ISL_1055315, EPI_ISL_1055316, EPI_ISL_1055317, EPI_ISL_1055319, EPI_ISL_1055320, EPI_ISL_1055321, EPI_ISL_1055322, EPI_ISL_1055323, EPI_ISL_1055324, EPI_ISL_1055325, EPI_ISL_1055326, EPI_ISL_1055327, EPI_ISL_1055329, EPI_ISL_1055330, EPI_ISL_1055332, EPI_ISL_1055334, EPI_ISL_1055335, EPI_ISL_1055336, EPI_ISL_1055337, EPI_ISL_1055338, EPI_ISL_1055339			
see above	Microbiological Diagnostic Unit - Public Health Laboratory (MDU-PHL)	MDU-PHL	Seemann T., Sait, M.L., Sherry, N.L.
EPI_ISL_1055340, EPI_ISL_1055341	Victorian Infectious Diseases Reference Laboratory (VIDRL)	VIDRL and MDU-PHL	Caly L., Seemann T., Sait, M.L., Druce J., Sherry, N.L.
EPI_ISL_1055342, EPI_ISL_1055343, EPI_ISL_1055344, EPI_ISL_1055345, EPI_ISL_1055346, EPI_ISL_1055347	Microbiological Diagnostic Unit - Public Health Laboratory (MDU-PHL)	MDU-PHL	Seemann T., Sait, M.L., Sherry, N.L.
EPI_ISL_1055348	Victorian Infectious Diseases Reference Laboratory (VIDRL)	VIDRL and MDU-PHL	Caly L., Seemann T., Sait, M.L., Druce J., Sherry, N.L.
EPI_ISL_1055352, EPI_ISL_1055354, EPI_ISL_1055357, EPI_ISL_1061399, EPI_ISL_1061400, EPI_ISL_1061401	Microbiological Diagnostic Unit - Public Health Laboratory (MDU-PHL)	MDU-PHL	Seemann T., Sait, M.L., Sherry, N.L.
EPI_ISL_406844	Monash Medical Centre	Collaboration between the University of Melbourne at The Peter Doherty Institute for Infection and Immunity, and the Victorian Infectious Disease Reference Laboratory	Caly,L., Seemann,T., Schultz,M., Druce,J. and Taiaroa,G
EPI_ISL_416410, EPI_ISL_416411, EPI_ISL_416412, EPI_ISL_416413, EPI_ISL_416415	Victorian Infectious Diseases Reference Laboratory (VIDRL)	Victorian Infectious Diseases Reference Laboratory and Microbiological Diagnostic Unit Public Health Laboratory, Doherty Institute	Caly L., Seemann T., Schultz M., Druce J., Taiaroa, G.
EPI_ISL_416514, EPI_ISL_416515, EPI_ISL_416516	Victorian Infectious Diseases Reference Laboratory (VIDRL)	Victorian Infectious Diseases Reference Laboratory and Microbiological Diagnostic Unit Public Health Laboratory, Doherty Institute	Caly L., Seemann T., Schultz M., Taiaroa, G., Druce J.
EPI_ISL_419714, EPI_ISL_419715, EPI_ISL_419716, EPI_ISL_419717, EPI_ISL_419718, EPI_ISL_419719, EPI_ISL_419720, EPI_ISL_419721, EPI_ISL_419722, EPI_ISL_419723, EPI_ISL_419725, EPI_ISL_419726, EPI_ISL_419727, EPI_ISL_419728, EPI_ISL_419729, EPI_ISL_419730, EPI_ISL_419731, EPI_ISL_419732			
see above	Microbiological Diagnostic Unit Public Health Laboratory	Microbiological Diagnostic Unit Public Health Laboratory	Seemann T., Schultz M., Sait, M., Sherry, N.
EPI_ISL_419733, EPI_ISL_419736, EPI_ISL_419737, EPI_ISL_419739, EPI_ISL_419740, EPI_ISL_419741, EPI_ISL_419742, EPI_ISL_419743, EPI_ISL_419744, EPI_ISL_419745, EPI_ISL_419746, EPI_ISL_419747, EPI_ISL_419748, EPI_ISL_419749, EPI_ISL_419750, EPI_ISL_419751, EPI_ISL_419752, EPI_ISL_419753, EPI_ISL_419754, EPI_ISL_419755, EPI_ISL_419756, EPI_ISL_419757, EPI_ISL_419758, EPI_ISL_419759, EPI_ISL_419760, EPI_ISL_419761, EPI_ISL_419762, EPI_ISL_419763, EPI_ISL_419764, EPI_ISL_419765, EPI_ISL_419766, EPI_ISL_419767, EPI_ISL_419768, EPI_ISL_419769, EPI_ISL_419770, EPI_ISL_419771, EPI_ISL_419772, EPI_ISL_419773, EPI_ISL_419774, EPI_ISL_419775, EPI_ISL_419776, EPI_ISL_419777, EPI_ISL_419778, EPI_ISL_419779, EPI_ISL_419780, EPI_ISL_419781, EPI_ISL_419782, EPI_ISL_419783, EPI_ISL_419784, EPI_ISL_419785, EPI_ISL_419786, EPI_ISL_419787, EPI_ISL_419788, EPI_ISL_419789, EPI_ISL_419790, EPI_ISL_419791, EPI_ISL_419792, EPI_ISL_419793, EPI_ISL_419794, EPI_ISL_419795, EPI_ISL_419796, EPI_ISL_419797, EPI_ISL_419798, EPI_ISL_419799, EPI_ISL_419800, EPI_ISL_419801, EPI_ISL_419802, EPI_ISL_419803, EPI_ISL_419804, EPI_ISL_419805, EPI_ISL_419806, EPI_ISL_419807, EPI_ISL_419808, EPI_ISL_419809, EPI_ISL_419810, EPI_ISL_419811, EPI_ISL_419812, EPI_ISL_419813, EPI_ISL_419814, EPI_ISL_419816, EPI_ISL_419817, EPI_ISL_419818, EPI_ISL_419819, EPI_ISL_419820, EPI_ISL_419821, EPI_ISL_419822			
see above	Victorian Infectious Diseases Reference Laboratory (VIDRL)	Victorian Infectious Diseases Reference Laboratory and Microbiological Diagnostic Unit Public Health Laboratory, Doherty Institute	Caly L., Seemann T., Sait, M., Schultz M., Druce J., Sherry, N.
EPI_ISL_419823, EPI_ISL_419824, EPI_ISL_419825	Microbiological Diagnostic Unit Public Health Laboratory	Microbiological Diagnostic Unit Public Health Laboratory	Seemann T., Schultz M., Sait, M., Sherry, N.
EPI_ISL_419826	Victorian Infectious Diseases Reference Laboratory (VIDRL)	Victorian Infectious Diseases Reference Laboratory and Microbiological Diagnostic Unit Public Health Laboratory, Doherty Institute	Caly L., Seemann T., Sait, M., Schultz M., Druce J., Sherry, N.
EPI_ISL_419827, EPI_ISL_419828, EPI_ISL_419829, EPI_ISL_419830	Microbiological Diagnostic Unit Public Health Laboratory	Microbiological Diagnostic Unit Public Health Laboratory	Seemann T., Schultz M., Sait, M., Sherry, N.
EPI_ISL_419834, EPI_ISL_419836, EPI_ISL_419837, EPI_ISL_419838, EPI_ISL_419839, EPI_ISL_419840, EPI_ISL_419841, EPI_ISL_419842, EPI_ISL_419843, EPI_ISL_419844, EPI_ISL_419845, EPI_ISL_419846, EPI_ISL_419847, EPI_ISL_419848, EPI_ISL_419849, EPI_ISL_419850, EPI_ISL_419851, EPI_ISL_419852, EPI_ISL_419853, EPI_ISL_419854, EPI_ISL_419855, EPI_ISL_419856, EPI_ISL_419857, EPI_ISL_419859, EPI_ISL_419860, EPI_ISL_419861, EPI_ISL_419862, EPI_ISL_419863, EPI_ISL_419864, EPI_ISL_419865, EPI_ISL_419866, EPI_ISL_419867, EPI_ISL_419868, EPI_ISL_419869, EPI_ISL_419870, EPI_ISL_419871, EPI_ISL_419872, EPI_ISL_419873, EPI_ISL_419874, EPI_ISL_419875, EPI_ISL_419876, EPI_ISL_419877, EPI_ISL_419878, EPI_ISL_419879, EPI_ISL_419880, EPI_ISL_419881, EPI_ISL_419882, EPI_ISL_419883, EPI_ISL_419884, EPI_ISL_419885, EPI_ISL_419886, EPI_ISL_419887, EPI_ISL_419888, EPI_ISL_419889, EPI_ISL_419890, EPI_ISL_419891, EPI_ISL_419892, EPI_ISL_419893, EPI_ISL_419894, EPI_ISL_419895, EPI_ISL_419896, EPI_ISL_419897, EPI_ISL_419898, EPI_ISL_419899, EPI_ISL_419900, EPI_ISL_419901, EPI_ISL_419902, EPI_ISL_419903, EPI_ISL_419904, EPI_ISL_419905, EPI_ISL_419906, EPI_ISL_419907, EPI_ISL_419908, EPI_ISL_419909, EPI_ISL_419910, EPI_ISL_419911, EPI_ISL_419912, EPI_ISL_419913, EPI_ISL_419914, EPI_ISL_419915, EPI_ISL_419916, EPI_ISL_419917, EPI_ISL_419918, EPI_ISL_419919, EPI_ISL_419920, EPI_ISL_419921, EPI_ISL_419922, EPI_ISL_419923, EPI_ISL_419924, EPI_ISL_419925, EPI_ISL_419926, EPI_ISL_419927, EPI_ISL_419928, EPI_ISL_419929, EPI_ISL_419931, EPI_ISL_419932, EPI_ISL_419933, EPI_ISL_419934, EPI_ISL_419935, EPI_ISL_419936, EPI_ISL_419937, EPI_ISL_419938, EPI_ISL_419939, EPI_ISL_419940, EPI_ISL_419941, EPI_ISL_419942, EPI_ISL_419943, EPI_ISL_419944, EPI_ISL_419945, EPI_ISL_419946, EPI_ISL_419947, EPI_ISL_419948, EPI_ISL_419949, EPI_ISL_419950, EPI_ISL_419951, EPI_ISL_419952, EPI_ISL_419953, EPI_ISL_419954, EPI_ISL_419955, EPI_ISL_419956, EPI_ISL_419957, EPI_ISL_419958, EPI_ISL_419959, EPI_ISL_419960, EPI_ISL_419961, EPI_ISL_419962, EPI_ISL_419963, EPI_ISL_419964, EPI_ISL_419965, EPI_ISL_419966, EPI_ISL_419967, EPI_ISL_419968, EPI_ISL_419969, EPI_ISL_419970, EPI_ISL_419971, EPI_ISL_419972, EPI_ISL_419973, EPI_ISL_419974, EPI_ISL_419975, EPI_ISL_419976, EPI_ISL_419977, EPI_ISL_419978, EPI_ISL_419979			
see above	Victorian Infectious Diseases Reference Laboratory (VIDRL)	Victorian Infectious Diseases Reference Laboratory and Microbiological Diagnostic Unit Public Health Laboratory, Doherty Institute	Caly L., Seemann T., Sait, M., Schultz M., Druce J., Sherry, N.
EPI_ISL_419980	Microbiological Diagnostic Unit Public Health Laboratory	Microbiological Diagnostic Unit Public Health Laboratory	Seemann T., Schultz M., Sait, M., Sherry, N.
EPI_ISL_419982, EPI_ISL_419983, EPI_ISL_419984, EPI_ISL_419985, EPI_ISL_419986, EPI_ISL_419987, EPI_ISL_419988, EPI_ISL_419989, EPI_ISL_419990, EPI_ISL_419991, EPI_ISL_419992, EPI_ISL_419993, EPI_ISL_419994, EPI_ISL_419995, EPI_ISL_419996, EPI_ISL_419997, EPI_ISL_419998			
see above	Victorian Infectious Diseases Reference Laboratory (VIDRL)	Victorian Infectious Diseases Reference Laboratory and Microbiological Diagnostic Unit Public Health Laboratory, Doherty Institute	Caly L., Seemann T., Sait, M., Schultz M., Druce J., Sherry, N.
EPI_ISL_419999, EPI_ISL_420000, EPI_ISL_420001, EPI_ISL_420002, EPI_ISL_420003	Microbiological Diagnostic Unit Public Health Laboratory	Microbiological Diagnostic Unit Public Health Laboratory	Seemann T., Schultz M., Sait, M., Sherry, N.
EPI_ISL_420004, EPI_ISL_420005	Victorian Infectious Diseases Reference Laboratory (VIDRL)	Victorian Infectious Diseases Reference Laboratory and	Caly L., Seemann T., Sait, M., Schultz M., Druce J., Sherry, N.

We gratefully acknowledge the following Authors from the Originating laboratories responsible for obtaining the specimens, as well as the Submitting laboratories where the genome data were generated and shared via GISAID, on which this research is based.

All Submitters of data may be contacted directly via www.gisaid.org

Authors are sorted alphabetically.

Accession ID	Originating Laboratory	Submitting Laboratory	Authors
EPI_ISL_1033126, EPI_ISL_1033127, EPI_ISL_1033131, EPI_ISL_1033132, EPI_ISL_1033133, EPI_ISL_1033134, EPI_ISL_1033141, EPI_ISL_1033144, EPI_ISL_1033145, EPI_ISL_1033146, EPI_ISL_1033147, EPI_ISL_1033148, EPI_ISL_1033149, EPI_ISL_1033150, EPI_ISL_1033151, EPI_ISL_1033152			
see above	Microbiological Diagnostic Unit - Public Health Laboratory (MDU-PHL)	MDU-PHL	Seemann T., Sait, M.L., Sherry, N.L.
EPI_ISL_1033154, EPI_ISL_1033155	Victorian Infectious Diseases Reference Laboratory (VIDRL)	VIDRL and MDU-PHL	Caly L., Seemann T., Sait, M.L., Druce J., Sherry, N.L.
EPI_ISL_1033156, EPI_ISL_1033157, EPI_ISL_1055265, EPI_ISL_1055267, EPI_ISL_1055270, EPI_ISL_1055272, EPI_ISL_1055274, EPI_ISL_1055275, EPI_ISL_1055276, EPI_ISL_1055277	Microbiological Diagnostic Unit - Public Health Laboratory (MDU-PHL)	MDU-PHL	Seemann T., Sait, M.L., Sherry, N.L.
EPI_ISL_1055278	Victorian Infectious Diseases Reference Laboratory (VIDRL)	VIDRL and MDU-PHL	Caly L., Seemann T., Sait, M.L., Druce J., Sherry, N.L.
EPI_ISL_1055279, EPI_ISL_1055280, EPI_ISL_1055281, EPI_ISL_1055282, EPI_ISL_1055286, EPI_ISL_1055297, EPI_ISL_1055298, EPI_ISL_1055299, EPI_ISL_1055307, EPI_ISL_1055309, EPI_ISL_1055318, EPI_ISL_1055331, EPI_ISL_1055333, EPI_ISL_1055349, EPI_ISL_1055350, EPI_ISL_1055353, EPI_ISL_1055355, EPI_ISL_1055356			
see above	Microbiological Diagnostic Unit - Public Health Laboratory (MDU-PHL)	MDU-PHL	Seemann T., Sait, M.L., Sherry, N.L.
EPI_ISL_1055358	Victorian Infectious Diseases Reference Laboratory (VIDRL)	VIDRL and MDU-PHL	Caly L., Seemann T., Sait, M.L., Druce J., Sherry, N.L.
EPI_ISL_1055359, EPI_ISL_1055360, EPI_ISL_1061402, EPI_ISL_1061403, EPI_ISL_1249986, EPI_ISL_1249988, EPI_ISL_1249990	Microbiological Diagnostic Unit - Public Health Laboratory (MDU-PHL)	MDU-PHL	Seemann T., Sait, M.L., Sherry, N.L.
EPI_ISL_1249993	Victorian Infectious Diseases Reference Laboratory (VIDRL)	VIDRL and MDU-PHL	Caly L., Seemann T., Sait, M.L., Druce J., Sherry, N.L.
EPI_ISL_1249999, EPI_ISL_1250005	Microbiological Diagnostic Unit - Public Health Laboratory (MDU-PHL)	MDU-PHL	Seemann T., Sait, M.L., Sherry, N.L.
EPI_ISL_562169, EPI_ISL_562172, EPI_ISL_562173, EPI_ISL_562174, EPI_ISL_562591, EPI_ISL_562592	Microbiological Diagnostic Unit - Public Health Laboratory (MDU-PHL)	MDU-PHL	Seemann, T., Schultz M. B., Sait, M., Sherry, N.
EPI_ISL_562593	Victorian Infectious Diseases Reference Laboratory (VIDRL)	VIDRL and MDU-PHL	Caly, L., Seemann, T., Sait, M., Schultz, M. B., Druce J., Sherry, N.
EPI_ISL_562594	Microbiological Diagnostic Unit - Public Health Laboratory (MDU-PHL)	MDU-PHL	Seemann, T., Schultz M. B., Sait, M., Sherry, N.
EPI_ISL_562595	Victorian Infectious Diseases Reference Laboratory (VIDRL)	VIDRL and MDU-PHL	Caly, L., Seemann, T., Sait, M., Schultz, M. B., Druce J., Sherry, N.
EPI_ISL_562596, EPI_ISL_562597, EPI_ISL_562598, EPI_ISL_562599, EPI_ISL_562600, EPI_ISL_562603, EPI_ISL_562604, EPI_ISL_562605, EPI_ISL_562609, EPI_ISL_562612, EPI_ISL_562613, EPI_ISL_562614, EPI_ISL_562615, EPI_ISL_562616, EPI_ISL_562617, EPI_ISL_562620, EPI_ISL_562621, EPI_ISL_562622, EPI_ISL_562623, EPI_ISL_562624, EPI_ISL_562625, EPI_ISL_562626, EPI_ISL_562627, EPI_ISL_562629, EPI_ISL_562631, EPI_ISL_562632, EPI_ISL_562633, EPI_ISL_562634, EPI_ISL_562635, EPI_ISL_562636, EPI_ISL_562637			
see above	Microbiological Diagnostic Unit - Public Health Laboratory (MDU-PHL)	MDU-PHL	Seemann, T., Schultz M. B., Sait, M., Sherry, N.
EPI_ISL_562640	Victorian Infectious Diseases Reference Laboratory (VIDRL)	VIDRL and MDU-PHL	Caly, L., Seemann, T., Sait, M., Schultz, M. B., Druce J., Sherry, N.
EPI_ISL_562642, EPI_ISL_562643, EPI_ISL_562644	Microbiological Diagnostic Unit - Public Health Laboratory (MDU-PHL)	MDU-PHL	Seemann, T., Schultz M. B., Sait, M., Sherry, N.
EPI_ISL_562645, EPI_ISL_562646	Victorian Infectious Diseases Reference Laboratory (VIDRL)	VIDRL and MDU-PHL	Caly, L., Seemann, T., Sait, M., Schultz, M. B., Druce J., Sherry, N.
EPI_ISL_562648, EPI_ISL_562649, EPI_ISL_562650, EPI_ISL_562651, EPI_ISL_562652, EPI_ISL_562653, EPI_ISL_562654, EPI_ISL_562655, EPI_ISL_562657, EPI_ISL_562661, EPI_ISL_562662, EPI_ISL_562663, EPI_ISL_562664, EPI_ISL_562666, EPI_ISL_562670, EPI_ISL_562673, EPI_ISL_562674, EPI_ISL_562675, EPI_ISL_562676, EPI_ISL_562677, EPI_ISL_562678, EPI_ISL_562679, EPI_ISL_562681, EPI_ISL_562682, EPI_ISL_562683, EPI_ISL_562684, EPI_ISL_562685, EPI_ISL_562686, EPI_ISL_562687, EPI_ISL_562688, EPI_ISL_562691, EPI_ISL_562692, EPI_ISL_562693, EPI_ISL_562694, EPI_ISL_562695, EPI_ISL_562698			
see above	Microbiological Diagnostic Unit - Public Health Laboratory (MDU-PHL)	MDU-PHL	Seemann, T., Schultz M. B., Sait, M., Sherry, N.
EPI_ISL_562700	Victorian Infectious Diseases Reference Laboratory (VIDRL)	VIDRL and MDU-PHL	Caly, L., Seemann, T., Sait, M., Schultz, M. B., Druce J., Sherry, N.
EPI_ISL_562701, EPI_ISL_562702, EPI_ISL_562706, EPI_ISL_562708, EPI_ISL_562709, EPI_ISL_562710, EPI_ISL_562711, EPI_ISL_562712, EPI_ISL_562713, EPI_ISL_562714, EPI_ISL_562715, EPI_ISL_562716, EPI_ISL_562717, EPI_ISL_562718, EPI_ISL_562719, EPI_ISL_562720, EPI_ISL_562721, EPI_ISL_562722, EPI_ISL_562723, EPI_ISL_562724, EPI_ISL_562725, EPI_ISL_562726, EPI_ISL_562727, EPI_ISL_562728, EPI_ISL_562729, EPI_ISL_562730, EPI_ISL_562731, EPI_ISL_562732, EPI_ISL_562733, EPI_ISL_562734, EPI_ISL_562735, EPI_ISL_562736, EPI_ISL_562737, EPI_ISL_562738, EPI_ISL_562739, EPI_ISL_562740, EPI_ISL_562741, EPI_ISL_562742, EPI_ISL_562743, EPI_ISL_562744, EPI_ISL_562745, EPI_ISL_562746, EPI_ISL_562747, EPI_ISL_562748, EPI_ISL_562749, EPI_ISL_562750, EPI_ISL_562751, EPI_ISL_562752, EPI_ISL_562753, EPI_ISL_562756, EPI_ISL_562761, EPI_ISL_562762, EPI_ISL_562763, EPI_ISL_562764, EPI_ISL_562765, EPI_ISL_562766, EPI_ISL_562767, EPI_ISL_562768, EPI_ISL_562769, EPI_ISL_562770, EPI_ISL_562771, EPI_ISL_562772, EPI_ISL_562773, EPI_ISL_562774, EPI_ISL_562775, EPI_ISL_562776, EPI_ISL_562777, EPI_ISL_562778, EPI_ISL_562779, EPI_ISL_562780, EPI_ISL_562781, EPI_ISL_562782, EPI_ISL_562783, EPI_ISL_562784, EPI_ISL_562786, EPI_ISL_562787, EPI_ISL_562788, EPI_ISL_562789			
see above	Microbiological Diagnostic Unit - Public Health Laboratory (MDU-PHL)	MDU-PHL	Seemann, T., Schultz M. B., Sait, M., Sherry, N.
EPI_ISL_562791	Victorian Infectious Diseases Reference Laboratory (VIDRL)	VIDRL and MDU-PHL	Caly, L., Seemann, T., Sait, M., Schultz, M. B., Druce J., Sherry, N.
EPI_ISL_562793, EPI_ISL_562794, EPI_ISL_562796, EPI_ISL_562797, EPI_ISL_562798, EPI_ISL_562799, EPI_ISL_562800	Microbiological Diagnostic Unit - Public Health Laboratory (MDU-PHL)	MDU-PHL	Seemann, T., Schultz M. B., Sait, M., Sherry, N.
EPI_ISL_562803, EPI_ISL_562804, EPI_ISL_562805, EPI_ISL_562806, EPI_ISL_562807, EPI_ISL_562808, EPI_ISL_562809	Victorian Infectious Diseases Reference Laboratory (VIDRL)	VIDRL and MDU-PHL	Caly, L., Seemann, T., Sait, M., Schultz, M. B., Druce J., Sherry, N.
EPI_ISL_562810, EPI_ISL_562811, EPI_ISL_562812, EPI_ISL_562813, EPI_ISL_562814, EPI_ISL_562815, EPI_ISL_562816, EPI_ISL_562817, EPI_ISL_562818, EPI_ISL_562819, EPI_ISL_562820, EPI_ISL_562821, EPI_ISL_562822, EPI_ISL_562823, EPI_ISL_562824, EPI_ISL_562825, EPI_ISL_562826, EPI_ISL_562827, EPI_ISL_562828, EPI_ISL_562829, EPI_ISL_562830, EPI_ISL_562831, EPI_ISL_562832, EPI_ISL_562833, EPI_ISL_562834, EPI_ISL_562835, EPI_ISL_562836, EPI_ISL_562837			
see above	Microbiological Diagnostic Unit - Public Health Laboratory	MDU-PHL	Seemann, T., Schultz M. B., Sait, M., Sherry, N.

Japan

We gratefully acknowledge the following Authors from the Originating laboratories responsible for obtaining the specimens, as well as the Submitting laboratories where the genome data were generated and shared via GISAID, on which this research is based.

All Submitters of data may be contacted directly via www.gisaid.org

Authors are sorted alphabetically.

Accession ID	Originating Laboratory	Submitting Laboratory	Authors
EPI_ISL_408665, EPI_ISL_408666, EPI_ISL_408667, EPI_ISL_408669	Dept. of Virology III, National Institute of Infectious Diseases	Pathogen Genomics Center, National Institute of Infectious Diseases	Tsuyoshi Sekizuka, Shutoku Matsuyama, Naganori Nao, Kazuya Shirato, Makoto Takeda, Makoto Kuroda
EPI_ISL_410531, EPI_ISL_410532	Dept. of Pathology, National Institute of Infectious Diseases	Pathogen Genomics Center, National Institute of Infectious Diseases	Tsuyoshi Sekizuka, Harutaka Katano, Shutoku Matsuyama, Naganori Nao, Kazuya Shirato, Motoi Suzuki, Hideki Hasegawa, Takaji Wakita, Makoto Takeda, Tadaki Suzuki, Makoto Kuroda
EPI_ISL_412968, EPI_ISL_412969	Takayuki Hishiki Kanagawa Prefectural Institute of Public Health	Takayuki Hishiki Kanagawa Prefectural Institute of Public Health	Hishiki,T., Suzuki,R., Sakuragi,J., Usui,K., Tanaka,Y., Kawai,J., Kogo,Y., Matsuki,Y., An,T., Hayashizaki,Y. and Takasaki,T.
EPI_ISL_413459	Department of Pathology, Toshima Hospital	Pathogen Genomics Center, National Institute of Infectious Diseases	Tsuyoshi Sekizuka, Kentaro Itokawa, Takuya Adachi, Masahiro Sano, Jun Yamazaki, Ippei Miyamoto, Haruka Nishioka, Ja-Mun Chong, Noriko Nakajima, Yuko Sato, Minoru Tobiume, Harutaka Katano, Tadaki Suzuki, Makoto Kuroda
EPI_ISL_414511	Department of Microbiology; Ryota Kumagai Tokyo Metropolitan Institute of Public Health	Tokyo Metoropolitan Institute of Public Health	Kumagai,R., Yoshida,I., Nagashima,M., Chiba,T. and Sadamasu,K.
EPI_ISL_416524	Saitama Medical University Hospital	Saitama Medical University	Kazuo Imai
EPI_ISL_416525	Saitama Medical University	Saitama Medical University	Kazuo Imai
EPI_ISL_416565, EPI_ISL_416566, EPI_ISL_416567, EPI_ISL_416569, EPI_ISL_416570, EPI_ISL_416571, EPI_ISL_416572, EPI_ISL_416573, EPI_ISL_416574, EPI_ISL_416575, EPI_ISL_416576, EPI_ISL_416577, EPI_ISL_416578, EPI_ISL_416579, EPI_ISL_416580, EPI_ISL_416581, EPI_ISL_416582, EPI_ISL_416583, EPI_ISL_416584, EPI_ISL_416585, EPI_ISL_416586, EPI_ISL_416587, EPI_ISL_416588, EPI_ISL_416589, EPI_ISL_416590, EPI_ISL_416591, EPI_ISL_416592, EPI_ISL_416593, EPI_ISL_416594, EPI_ISL_416595, EPI_ISL_416596, EPI_ISL_416597, EPI_ISL_416598, EPI_ISL_416599, EPI_ISL_416600, EPI_ISL_416601, EPI_ISL_416602, EPI_ISL_416603, EPI_ISL_416604, EPI_ISL_416605, EPI_ISL_416606, EPI_ISL_416607, EPI_ISL_416608, EPI_ISL_416609, EPI_ISL_416610, EPI_ISL_416611, EPI_ISL_416612, EPI_ISL_416613, EPI_ISL_416614, EPI_ISL_416615, EPI_ISL_416617, EPI_ISL_416618, EPI_ISL_416619, EPI_ISL_416620, EPI_ISL_416621, EPI_ISL_416622, EPI_ISL_416624, EPI_ISL_416625, EPI_ISL_416626, EPI_ISL_416627, EPI_ISL_416628, EPI_ISL_416629, EPI_ISL_416630, EPI_ISL_416631, EPI_ISL_416632, EPI_ISL_416633, EPI_ISL_416634			
see above	Japanese Quarantine Stations	Pathogen Genomics Center, National Institute of Infectious Diseases	Tsuyoshi Sekizuka, Kentaro Itokawa, Rina Tanaka, Masanori Hashino, Tsutomu Kageyama, Shinji Saito, Ikuyo Takayama, Hideki Hasegawa, Takuri Takahashi, Hajime Kamiya, Takuya Yamagishi, Motoi Suzuki, Takaji Wakita, Makoto Kuroda
EPI_ISL_418809	University of Wisconsin - Madison: Influenza Research Institute	University of Wisconsin Madison, AIDS Vaccine Research Laboratories	Katarina Braun, Gage Moreno, Peter Halfmann, et al.
EPI_ISL_419296	Kochi Prefectural Institute of Public Health	Pathogen Genomics Center, National Institute of Infectious Diseases	Tsuyoshi Sekizuka, Akihiko Tokaji, Kentaro Itokawa, Rina Tanaka, Masanori Hashino, Hajime Kamiya, Motoi Suzuki, Makoto Kuroda
EPI_ISL_419297, EPI_ISL_419298	Chiba Prefectural Institute of Public Health	Pathogen Genomics Center, National Institute of Infectious Diseases	Tsuyoshi Sekizuka, Masakatsu Taira, Yushi Hachisu, Kentaro Itokawa, Rina Tanaka, Masanori Hashino, Hajime Kamiya, Motoi Suzuki, Makoto Kuroda
EPI_ISL_419299, EPI_ISL_419300	Ishikawa Prefectural Institute of Public Health and Environmental Science	Pathogen Genomics Center, National Institute of Infectious Diseases	Tsuyoshi Sekizuka, Sanae Kuramoto, Eri Nariai, Kentaro Itokawa, Rina Tanaka, Masanori Hashino, Hajime Kamiya, Motoi Suzuki, Makoto Kuroda
EPI_ISL_419301, EPI_ISL_419302, EPI_ISL_419303, EPI_ISL_419304, EPI_ISL_419305, EPI_ISL_419306, EPI_ISL_419307, EPI_ISL_419308	Saitama Prefectural Institute of Public Health	Pathogen Genomics Center, National Institute of Infectious Diseases	Tsuyoshi Sekizuka, Michiyo Shinohara, Tsuyoshi Kishimoto, Kentaro Itokawa, Rina Tanaka, Masanori Hashino, Hajime Kamiya, Motoi Suzuki, Makoto Kuroda
EPI_ISL_419309, EPI_ISL_419310, EPI_ISL_419311	Chiba Prefectural Institute of Public Health	Pathogen Genomics Center, National Institute of Infectious Diseases	Tsuyoshi Sekizuka, Masakatsu Taira, Yushi Hachisu, Kentaro Itokawa, Rina Tanaka, Masanori Hashino, Hajime Kamiya, Motoi Suzuki, Makoto Kuroda
EPI_ISL_420889, EPI_ISL_420890	Takayuki Hishiki Kanagawa Prefectural Institute of Public Health	Takayuki Hishiki Kanagawa Prefectural Institute of Public Health	Hishiki,T., Suzuki,R., Sakuragi,J., Usui,K., Tanaka,Y., Kawai,J., Kogo,Y., Matsuki,Y., An,T., Hayashizaki,Y. and Takasaki,T.
EPI_ISL_434561, EPI_ISL_434562	Department of Microbiology; Ryota Kumagai Tokyo Metropolitan Institute of Public Health	Department of Microbiology; Ryota Kumagai Tokyo Metropolitan Institute of Public Health	Kumagai,R., Yoshida,I., Asakura,H., Nagashima,M., Chiba,T. and Sadamasu,K.
EPI_ISL_437601	Keio University School of Medicine	Keio University School of Medicine	Kenjiro Kosaki, Yuka Iwasaki, Toshiki Takenouchi, Haruhiko Siomi,
EPI_ISL_438947, EPI_ISL_438948, EPI_ISL_438949, EPI_ISL_438951, EPI_ISL_438952, EPI_ISL_438953, EPI_ISL_438954, EPI_ISL_438955, EPI_ISL_438956, EPI_ISL_438958, EPI_ISL_438959, EPI_ISL_438961, EPI_ISL_438965, EPI_ISL_438966, EPI_ISL_438967, EPI_ISL_438969, EPI_ISL_438970, EPI_ISL_438971, EPI_ISL_438972			
see above	Keio University School of Medicine	Keio University School of Medicine	Kenjiro Kosaki
EPI_ISL_445183	Takayuki Hishiki Kanagawa Prefectural Institute of Public Health	Takayuki Hishiki Kanagawa Prefectural Institute of Public Health	Hishiki,T., Suzuki,R., Sakuragi,J., Usui,K., Tanaka,Y., Kawai,J., Kogo,Y., Matsuki,Y., An,T., Hayashizaki,Y. and Takasaki,T.
EPI_ISL_454749	Japanese Quarantine Stations	Pathogen Genomics Center, National Institute of Infectious Diseases	Tsuyoshi Sekizuka, Kentaro Itokawa, Rina Tanaka, Masanori Hashino, Tsutomu Kageyama, Shinji Saito, Ikuyo Takayama, Hideki Hasegawa, Takuri Takahashi, Hajime Kamiya, Takuya Yamagishi, Motoi Suzuki, Takaji Wakita, Makoto Kuroda
EPI_ISL_462439, EPI_ISL_462440, EPI_ISL_462441, EPI_ISL_462442, EPI_ISL_462443, EPI_ISL_462444, EPI_ISL_462445	unknown	Ryota Kumagai Tokyo Metropolitan Institute of Public Health	Asakura,H., Kumagai,R., Yoshida,I., Nagashima,M., Chiba,T., Sadamasu,K.
EPI_ISL_462446	unknown	Ryota Kumagai Tokyo Metropolitan Institute of Public Health	Kumagai,R., Yoshida,I., Asakura,H., Nagashima,M., Chiba,T., Sadamasu,K.
EPI_ISL_468724, EPI_ISL_468725	unknown	Contact:Ryota Kumagai Tokyo Metropolitan Institute of Public Health	Kumagai,R., Yoshida,I., Asakura,H., Nagashima,M., Chiba,T., Sadamasu,K.
EPI_ISL_469287, EPI_ISL_469288, EPI_ISL_469289, EPI_ISL_469290, EPI_ISL_469291, EPI_ISL_469292, EPI_ISL_469293, EPI_ISL_469294, EPI_ISL_469295, EPI_ISL_469296	Keio University Hospital	Keio University Hospital	Kenjiro Kosaki
EPI_ISL_479676, EPI_ISL_479677, EPI_ISL_479678, EPI_ISL_479679, EPI_ISL_479680, EPI_ISL_479681, EPI_ISL_479682, EPI_ISL_479683, EPI_ISL_479684, EPI_ISL_479685	unknown	Contact:Hiroyuki Asakura Tokyo Metropolitan Institute of Public Health, Department of Microbiology	Asakura,H., Yoshida,I., Kumagai,R., Nagashima,M., Chiba,T., Sadamasu,K.
EPI_ISL_479792, EPI_ISL_479793,	Hokkaido Institute of Public Health	Pathogen Genomics Center, National Institute of Infectious	Tsuyoshi Sekizuka, Rika Komagome, Kentaro Itokawa, Rina Tanaka, Masanori Hashino, Hajime Kamiya, Motoi Suzuki, Makoto Kuroda

We gratefully acknowledge the following Authors from the Originating laboratories responsible for obtaining the specimens, as well as the Submitting laboratories where the genome data were generated and shared via GISAID, on which this research is based.

All Submitters of data may be contacted directly via www.gisaid.org

Authors are sorted alphabetically.

Accession ID	Originating Laboratory	Submitting Laboratory	Authors
EPI_ISL_1020317, EPI_ISL_1020318, EPI_ISL_1020319, EPI_ISL_1020320, EPI_ISL_1020321, EPI_ISL_1020322, EPI_ISL_1020324, EPI_ISL_1020325, EPI_ISL_1020326, EPI_ISL_1020327	Department of Infectious Diseases and Immunology, National Hospital Organization Nagoya Medical Center	Clinical Research Center, National Hospital Organization Nagoya Medical Center	Yoshihiro Nakata, Hirotaka Ode, Mai Kubota, Masakazu Matsuda, Kazuhiro Matsuoka, Miho Nakasuji, Mikiko Mori, Mayumi Imahashi, Yoshiyuki Yokomaku, Yasumasa Iwatani
EPI_ISL_1034196, EPI_ISL_1034197, EPI_ISL_1034198, EPI_ISL_1034199, EPI_ISL_1034200, EPI_ISL_1034201, EPI_ISL_1034202, EPI_ISL_1034203, EPI_ISL_1034204, EPI_ISL_1034205, EPI_ISL_1034206, EPI_ISL_1034207, EPI_ISL_1034208			
see above	Tokyo Metropolitan Institute of Public Health	Tokyo Metropolitan Institute of Public Health	Masaki Hayashi, Takako Yamazaki, Mami Nagashima,Takushi Fujiwara, Takashi Chiba, Kenji Sadamasu
EPI_ISL_1034212, EPI_ISL_1034213, EPI_ISL_1034214, EPI_ISL_1034215, EPI_ISL_1034216, EPI_ISL_1034217, EPI_ISL_1034218, EPI_ISL_1034219, EPI_ISL_1034220, EPI_ISL_1034221, EPI_ISL_1034222, EPI_ISL_1034223, EPI_ISL_1034224, EPI_ISL_1034225, EPI_ISL_1034226			
see above	Tokyo Metropolitan Institute of Public Health	Tokyo Metropolitan Institute of Public Health	Masaki Hayashi, Takako Yamazaki, Mami Nagashima, Takushi Fujiwara, Takashi Chiba, Kenji Sadamasu
EPI_ISL_1041931, EPI_ISL_1041932, EPI_ISL_1041933, EPI_ISL_1041934, EPI_ISL_1041935, EPI_ISL_1041936, EPI_ISL_1041937, EPI_ISL_1041938, EPI_ISL_1041939, EPI_ISL_1041940, EPI_ISL_1041941, EPI_ISL_1041942, EPI_ISL_1041943, EPI_ISL_1041944, EPI_ISL_1041945, EPI_ISL_1041946, EPI_ISL_1041947, EPI_ISL_1041948, EPI_ISL_1041949, EPI_ISL_1041950, EPI_ISL_1041951, EPI_ISL_1041952, EPI_ISL_1041953, EPI_ISL_1041954, EPI_ISL_1041955			
see above	Tokyo Metropolitan Institute of Public Health	Tokyo Metropolitan Institute of Public Health	Masaki Hayashi, Takako Yamazaki, Ayano Hotta, Souichi Yoshikawa, Tomoki Igarashi, Kayo Hagino, Maki Kanda, Kenji Iida, Mami Nagashima,Takushi Fujiwara, Takashi Chiba, Kenji Sadamasu
EPI_ISL_1046761	SARS-CoV-2 testing team, National Institute of Infectious Diseases	Pathogen Genomics Center, National Institute of Infectious Diseases	Tsuyoshi Sekizuka, Kentaro Itokawa, Rina Tanaka, Masanori Hashino, Minoru Nagi, Ken Miyazawa, Takashi Sakudoh, Nozomu Hanaoka, Tsuguto Fujimoto, Makoto Kuroda
EPI_ISL_1046762, EPI_ISL_1046763, EPI_ISL_1046764, EPI_ISL_1046765	SARS-CoV-2 testing team, National Institute of Infectious Diseases	Pathogen Genomics Center, National Institute of Infectious Diseases	Tsuyoshi Sekizuka, Kentaro Itokawa, Rina Tanaka, Masanori Hashino, Koichi Ishikawa, Midori Nakamura-Hoshi, Shigeru Kusagawa, Makoto Kuroda
EPI_ISL_1046766	SARS-CoV-2 testing team, National Institute of Infectious Diseases	Pathogen Genomics Center, National Institute of Infectious Diseases	Tsuyoshi Sekizuka, Kentaro Itokawa, Rina Tanaka, Masanori Hashino, Minoru Nagi, Ken Miyazawa, Takashi Sakudoh, Nozomu Hanaoka, Tsuguto Fujimoto, Makoto Kuroda
EPI_ISL_1046768	SARS-CoV-2 testing team, National Institute of Infectious Diseases	Pathogen Genomics Center, National Institute of Infectious Diseases	Tsuyoshi Sekizuka, Kentaro Itokawa, Rina Tanaka, Masanori Hashino, Takahiro Maeki, Motohiko Ogawa, Chang-Kweng Lim, Makoto Kuroda
EPI_ISL_1046769	SARS-CoV-2 testing team, National Institute of Infectious Diseases	Pathogen Genomics Center, National Institute of Infectious Diseases	Tsuyoshi Sekizuka, Kentaro Itokawa, Rina Tanaka, Masanori Hashino, Koichi Ishikawa, Midori Nakamura-Hoshi, Shigeru Kusagawa, Makoto Kuroda
EPI_ISL_1046774, EPI_ISL_1046775	SARS-CoV-2 testing team, National Institute of Infectious Diseases	Pathogen Genomics Center, National Institute of Infectious Diseases	Tsuyoshi Sekizuka, Kentaro Itokawa, Rina Tanaka, Masanori Hashino, Minoru Nagi, Ken Miyazawa, Takashi Sakudoh, Nozomu Hanaoka, Tsuguto Fujimoto, Makoto Kuroda
EPI_ISL_1046777	SARS-CoV-2 testing team, National Institute of Infectious Diseases	Pathogen Genomics Center, National Institute of Infectious Diseases	Tsuyoshi Sekizuka, Kentaro Itokawa, Rina Tanaka, Masanori Hashino, Takahiro Maeki, Motohiko Ogawa, Chang-Kweng Lim, Makoto Kuroda
EPI_ISL_1046778	SARS-CoV-2 testing team, National Institute of Infectious Diseases	Pathogen Genomics Center, National Institute of Infectious Diseases	Tsuyoshi Sekizuka, Kentaro Itokawa, Rina Tanaka, Masanori Hashino, Eri Nakayama, Shigeru Tajima, Chang-Kweng Lim, Makoto Kuroda
EPI_ISL_1046779, EPI_ISL_1046780, EPI_ISL_1046781, EPI_ISL_1046782	SARS-CoV-2 testing team, National Institute of Infectious Diseases	Pathogen Genomics Center, National Institute of Infectious Diseases	Tsuyoshi Sekizuka, Kentaro Itokawa, Rina Tanaka, Masanori Hashino, Minoru Nagi, Ken Miyazawa, Takashi Sakudoh, Nozomu Hanaoka, Tsuguto Fujimoto, Makoto Kuroda
EPI_ISL_1046783, EPI_ISL_1046784, EPI_ISL_1046785	SARS-CoV-2 testing team, National Institute of Infectious Diseases	Pathogen Genomics Center, National Institute of Infectious Diseases	Tsuyoshi Sekizuka, Kentaro Itokawa, Rina Tanaka, Masanori Hashino, Eri Nakayama, Shigeru Tajima, Chang-Kweng Lim, Makoto Kuroda
EPI_ISL_1046786, EPI_ISL_1046787	SARS-CoV-2 testing team, National Institute of Infectious Diseases	Pathogen Genomics Center, National Institute of Infectious Diseases	Tsuyoshi Sekizuka, Kentaro Itokawa, Rina Tanaka, Masanori Hashino, Takahiro Maeki, Motohiko Ogawa, Chang-Kweng Lim, Makoto Kuroda
EPI_ISL_1046788	SARS-CoV-2 testing team, National Institute of Infectious Diseases	Pathogen Genomics Center, National Institute of Infectious Diseases	Tsuyoshi Sekizuka, Kentaro Itokawa, Rina Tanaka, Masanori Hashino, Tadashi Kikuchi, Lucky Ronald Runtuwene, Shigeru Kusagawa, Makoto Kuroda
EPI_ISL_1046789, EPI_ISL_1046790	SARS-CoV-2 testing team, National Institute of Infectious Diseases	Pathogen Genomics Center, National Institute of Infectious Diseases	Tsuyoshi Sekizuka, Kentaro Itokawa, Rina Tanaka, Masanori Hashino, Koichi Ishikawa, Midori Nakamura-Hoshi, Shigeru Kusagawa, Makoto Kuroda
EPI_ISL_1069157, EPI_ISL_1069158, EPI_ISL_1069159, EPI_ISL_1069160, EPI_ISL_1069161, EPI_ISL_1069162, EPI_ISL_1069163, EPI_ISL_1069164, EPI_ISL_1069165, EPI_ISL_1069166, EPI_ISL_1069167, EPI_ISL_1069168, EPI_ISL_1069171			
see above	Tokyo Metropolitan Institute of Public Health	Tokyo Metropolitan Institute of Public Health	Takako Yamazaki, Masaki Hayashi, Mami Nagashima,Takushi Fujiwara, Takashi Chiba, Kenji Sadamasu
EPI_ISL_1072966, EPI_ISL_1072967, EPI_ISL_1072968, EPI_ISL_1072969, EPI_ISL_1072970, EPI_ISL_1072971, EPI_ISL_1072972, EPI_ISL_1072973, EPI_ISL_1072974, EPI_ISL_1072975, EPI_ISL_1072976, EPI_ISL_1072977, EPI_ISL_1072978, EPI_ISL_1072979, EPI_ISL_1072980, EPI_ISL_1072981, EPI_ISL_1072982, EPI_ISL_1072983, EPI_ISL_1072984			
see above	Tokyo Metropolitan Institute of Public Health	Tokyo Metropolitan Institute of Public Health	Masaki Hayashi, Takako Yamazaki, Mami Nagashima,Takushi Fujiwara, Takashi Chiba, Kenji Sadamasu
EPI_ISL_1078589, EPI_ISL_1078590, EPI_ISL_1078592, EPI_ISL_1078593, EPI_ISL_1078594, EPI_ISL_1078596, EPI_ISL_1078597, EPI_ISL_1078599, EPI_ISL_1078600, EPI_ISL_1078601, EPI_ISL_1078603, EPI_ISL_1078604, EPI_ISL_1078606, EPI_ISL_1078607, EPI_ISL_1078608, EPI_ISL_1078610, EPI_ISL_1078613, EPI_ISL_1078614, EPI_ISL_1078616, EPI_ISL_1078618, EPI_ISL_1078620, EPI_ISL_1078621			
see above	Tokyo Metropolitan Institute of Public Health	Tokyo Metropolitan Institute of Public Health	Masaki Hayashi, Takako Yamazaki, Ayano Hotta, Souichi Yoshikawa, Tomoki Igarashi, Kayo Hagino, Maki Kanda, Kenji Iida, Mami Nagashima,Takushi Fujiwara, Takashi Chiba, Kenji Sadamasu
EPI_ISL_768526, EPI_ISL_768527	Department of Virology I, National Institute of Infectious Diseases	Pathogen Genomics Center, National Institute of Infectious Diseases	Tsuyoshi Sekizuka, Shuetsu Fukushi, Souichi Yamada, Kentaro Itokawa, Rina Tanaka, Masanori Hashino, Makoto Kuroda
EPI_ISL_768634, EPI_ISL_768635, EPI_ISL_768636, EPI_ISL_768637, EPI_ISL_768638, EPI_ISL_768639, EPI_ISL_768640, EPI_ISL_768641, EPI_ISL_768642, EPI_ISL_768643, EPI_ISL_768644, EPI_ISL_768645, EPI_ISL_768646, EPI_ISL_768647, EPI_ISL_768648, EPI_ISL_768649, EPI_ISL_768650, EPI_ISL_768651, EPI_ISL_768652, EPI_ISL_768653, EPI_ISL_768654, EPI_ISL_768655, EPI_ISL_768656, EPI_ISL_768657, EPI_ISL_768658, EPI_ISL_768659, EPI_ISL_768660, EPI_ISL_768661, EPI_ISL_768662, EPI_ISL_768663, EPI_ISL_768664, EPI_ISL_768665, EPI_ISL_768666, EPI_ISL_768667, EPI_ISL_768668, EPI_ISL_768669, EPI_ISL_768670, EPI_ISL_768671, EPI_ISL_768672, EPI_ISL_768673, EPI_ISL_768674, EPI_ISL_768675, EPI_ISL_768676, EPI_ISL_768677, EPI_ISL_768678, EPI_ISL_768679, EPI_ISL_768680, EPI_ISL_768681, EPI_ISL_768682, EPI_ISL_768683, EPI_ISL_768684, EPI_ISL_768685, EPI_ISL_768686, EPI_ISL_768687, EPI_ISL_768688, EPI_ISL_768689, EPI_ISL_768691, EPI_ISL_768692, EPI_ISL_768693, EPI_ISL_768694, EPI_ISL_768695, EPI_ISL_768696, EPI_ISL_768697, EPI_ISL_768698, EPI_ISL_768699, EPI_ISL_768700, EPI_ISL_768701, EPI_ISL_768702, EPI_ISL_768703, EPI_ISL_768704, EPI_ISL_768705, EPI_ISL_768706, EPI_ISL_768707, EPI_ISL_768708, EPI_ISL_768709, EPI_ISL_768710, EPI_ISL_768711, EPI_ISL_768712, EPI_ISL_768713, EPI_ISL_768714, EPI_ISL_768715, EPI_ISL_768716, EPI_ISL_768718, EPI_ISL_768719, EPI_ISL_768720, EPI_ISL_768721, EPI_ISL_768722, EPI_ISL_768723, EPI_ISL_768724, EPI_ISL_779199, EPI_ISL_779200, EPI_ISL_779201, EPI_ISL_779202, EPI_ISL_779203, EPI_ISL_779204, EPI_ISL_779205, EPI_ISL_779206, EPI_ISL_779207, EPI_ISL_779208, EPI_ISL_779209, EPI_ISL_779210, EPI_ISL_779211, EPI_ISL_779212, EPI_ISL_779213, EPI_ISL_779214, EPI_ISL_779215, EPI_ISL_779216, EPI_ISL_779217, EPI_ISL_779218, EPI_ISL_779219, EPI_ISL_779220, EPI_ISL_779222, EPI_ISL_779223, EPI_ISL_779224, EPI_ISL_779225, EPI_ISL_779226, EPI_ISL_779227, EPI_ISL_779228, EPI_ISL_779229, EPI_ISL_779231, EPI_ISL_779232, EPI_ISL_779234, EPI_ISL_779235, EPI_ISL_779236, EPI_ISL_779237, EPI_ISL_779238, EPI_ISL_779239, EPI_ISL_779240, EPI_ISL_779241, EPI_ISL_779242, EPI_ISL_779243, EPI_ISL_779244, EPI_ISL_779245, EPI_ISL_779246, EPI_ISL_779247, EPI_ISL_779248, EPI_ISL_779249, EPI_ISL_779250, EPI_ISL_779251, EPI_ISL_779252, EPI_ISL_779661, EPI_ISL_779662, EPI_ISL_779663, EPI_ISL_779664, EPI_ISL_779665, EPI_ISL_779666, EPI_ISL_779667, EPI_ISL_779668, EPI_ISL_779669, EPI_ISL_779670, EPI_ISL_779671, EPI_ISL_779672, EPI_ISL_779673, EPI_ISL_779674, EPI_ISL_779675, EPI_ISL_779676, EPI_ISL_779677, EPI_ISL_779678, EPI_ISL_779679, EPI_ISL_779680, EPI_ISL_779681, EPI_ISL_779682, EPI_ISL_779683, EPI_ISL_779684, EPI_ISL_779685, EPI_ISL_779686, EPI_ISL_779687, EPI_ISL_779688, EPI_ISL_779689, EPI_ISL_779690, EPI_ISL_779691, EPI_ISL_779693, EPI_ISL_779694, EPI_ISL_779695, EPI_ISL_779696, EPI_ISL_779697, EPI_ISL_779698, EPI_ISL_779699, EPI_ISL_779700, EPI_ISL_779701, EPI_ISL_779702, EPI_ISL_792680, EPI_ISL_792681, EPI_ISL_792682, EPI_ISL_792683			

We gratefully acknowledge the following Authors from the Originating laboratories responsible for obtaining the specimens, as well as the Submitting laboratories where the genome data were generated and shared via GISAID, on which this research is based.

All Submitters of data may be contacted directly via www.gisaid.org

Authors are sorted alphabetically.

Accession ID	Originating Laboratory	Submitting Laboratory	Authors
EPI_ISL_1098660, EPI_ISL_1098661, EPI_ISL_1098662, EPI_ISL_1098663, EPI_ISL_1098664, EPI_ISL_1098665, EPI_ISL_1098666, EPI_ISL_1098667, EPI_ISL_1098668, EPI_ISL_1098669, EPI_ISL_1098670, EPI_ISL_1098671, EPI_ISL_1098672, EPI_ISL_1098673, EPI_ISL_1098674, EPI_ISL_1098675, EPI_ISL_1098676, EPI_ISL_1098677, EPI_ISL_1098678, EPI_ISL_1098679, EPI_ISL_1098680, EPI_ISL_1098681, EPI_ISL_1098682, EPI_ISL_1098683, EPI_ISL_1098684, EPI_ISL_1098685, EPI_ISL_1098686, EPI_ISL_1098687, EPI_ISL_1098688, EPI_ISL_1098689, EPI_ISL_1098690, EPI_ISL_1098691, EPI_ISL_1098692, EPI_ISL_1098693, EPI_ISL_1098694, EPI_ISL_1098695, EPI_ISL_1098696, EPI_ISL_1098697, EPI_ISL_1098698, EPI_ISL_1098699, EPI_ISL_1098700, EPI_ISL_1098701, EPI_ISL_1098702, EPI_ISL_1098703, EPI_ISL_1098704, EPI_ISL_1098705, EPI_ISL_1098706, EPI_ISL_1098707, EPI_ISL_1098708, EPI_ISL_1098709, EPI_ISL_1098710, EPI_ISL_1098711, EPI_ISL_1098712, EPI_ISL_1098713, EPI_ISL_1098714, EPI_ISL_1098715, EPI_ISL_1098716, EPI_ISL_1098717, EPI_ISL_1098718, EPI_ISL_1098719, EPI_ISL_1098720, EPI_ISL_1098721, EPI_ISL_1098722, EPI_ISL_1098723, EPI_ISL_1098724, EPI_ISL_1098725, EPI_ISL_1098726, EPI_ISL_1098727, EPI_ISL_1098728, EPI_ISL_1098729, EPI_ISL_1098730, EPI_ISL_1098731, EPI_ISL_1098732, EPI_ISL_1098733, EPI_ISL_1098734, EPI_ISL_1098735, EPI_ISL_1098736, EPI_ISL_1098737, EPI_ISL_1098738, EPI_ISL_1098739, EPI_ISL_1098740, EPI_ISL_1098741, EPI_ISL_1098742, EPI_ISL_1098743, EPI_ISL_1098744, EPI_ISL_1098745, EPI_ISL_1098746, EPI_ISL_1098747, EPI_ISL_1098748, EPI_ISL_1098749, EPI_ISL_1098750, EPI_ISL_1098751, EPI_ISL_1098752, EPI_ISL_1098753, EPI_ISL_1098754			
see above	Fukuoka Institute of Health and Environmental Sciences	Fukuoka Institute of Health and Environmental Sciences	Asako Nakamura, Yoshiki Etoh, Hiroaki Shigemura, Akira Ohishi, Takayuki Kobayashi, Saori Ueda, Chiharu Katamune, Mitsuhiro Hamasaki, Yuki Ashizuka, Tsuyoshi Sekizuka, Kentaro Itokawa, Rina Tanaka, Masanori Hashino, Makoto Kuroda
EPI_ISL_1121961, EPI_ISL_1121962	Department of Respiratory Medicine, Nagasaki University Hospital, Nagasaki, Japan	Department of Virology, Institute of Tropical Medicine, Nagasaki University, Nagasaki, Japan	Satoshi Irifune, Nobuyuki Ashizawa, Takahiro Takazono, Kenji Ota, Tatsuro Hirayama, Ayumi Fujita, Masato Tashiro, Takeshi Tanaka, Kazuko Yamamoto, Yoshifumi Imamura, Taiga Miyazaki, Kouichi Morita, Katsunori Yanagihara, Koichi Izumikawa, and Hiroshi Mukae
EPI_ISL_1122349, EPI_ISL_1122350, EPI_ISL_1122351, EPI_ISL_1122352, EPI_ISL_1122353, EPI_ISL_1122354, EPI_ISL_1122355, EPI_ISL_1122356	Ibaraki Prefectural Institute of Public Health	Ibaraki Prefectural Institute of Public Health	Keiko Goto, Tsuyoshi Sekizuka, Kentaro Itokawa, Rina Tanaka, Masanori Hashino, Makoto Kuroda
EPI_ISL_1122890, EPI_ISL_1123289	Department of Virology I, National Institute of Infectious Diseases	Department of Veterinary Science, National Institute of Infectious Diseases	Yudai Kuroda, Tsukasa Yamamoto, Keita Ishijima, Souichi Yamada, Shuetsu Fukushi, Ken Maeda
EPI_ISL_1123300, EPI_ISL_1123301, EPI_ISL_1123302, EPI_ISL_1123303, EPI_ISL_1123304, EPI_ISL_1123305, EPI_ISL_1123306, EPI_ISL_1123307, EPI_ISL_1123308, EPI_ISL_1123309, EPI_ISL_1123310, EPI_ISL_1123311, EPI_ISL_1123312, EPI_ISL_1123313, EPI_ISL_1123314, EPI_ISL_1123315, EPI_ISL_1123316, EPI_ISL_1123317, EPI_ISL_1123318, EPI_ISL_1123319, EPI_ISL_1123320, EPI_ISL_1123321, EPI_ISL_1123322, EPI_ISL_1123323, EPI_ISL_1123324, EPI_ISL_1123326, EPI_ISL_1123327, EPI_ISL_1123328, EPI_ISL_1123329, EPI_ISL_1123330, EPI_ISL_1123331, EPI_ISL_1123333, EPI_ISL_1123334, EPI_ISL_1123335, EPI_ISL_1123336, EPI_ISL_1123337, EPI_ISL_1123338, EPI_ISL_1123339, EPI_ISL_1123340, EPI_ISL_1123341, EPI_ISL_1123342, EPI_ISL_1123343			
see above	Tokyo Metropolitan Institute of Public Health	Tokyo Metropolitan Institute of Public Health	Takako Yamazaki, Masaki Hayashi, Mami Nagashima,Takushi Fujiwara, Takashi Chiba, Kenji Sadamasu
EPI_ISL_1123377, EPI_ISL_1123378, EPI_ISL_1123379, EPI_ISL_1123380, EPI_ISL_1123381, EPI_ISL_1123382, EPI_ISL_1123383, EPI_ISL_1123384, EPI_ISL_1123385, EPI_ISL_1123386, EPI_ISL_1123387			
see above	Tokyo Metropolitan Institute of Public Health	Tokyo Metropolitan Institute of Public Health	Masaki Hayashi, Takako Yamazaki, Mami Nagashima,Takushi Fujiwara, Takashi Chiba, Kenji Sadamasu
EPI_ISL_1123389, EPI_ISL_1123390, EPI_ISL_1123391, EPI_ISL_1123392, EPI_ISL_1123393, EPI_ISL_1123394, EPI_ISL_1123395, EPI_ISL_1123396, EPI_ISL_1123397, EPI_ISL_1123398, EPI_ISL_1123399			
see above	Tokyo Metropolitan Institute of Public Health	Tokyo Metropolitan Institute of Public Health	Takako Yamazaki, Masaki Hayashi, Mami Nagashima,Takushi Fujiwara, Takashi Chiba, Kenji Sadamasu
EPI_ISL_1123400, EPI_ISL_1123401, EPI_ISL_1123402, EPI_ISL_1123403, EPI_ISL_1123404, EPI_ISL_1123405, EPI_ISL_1123406, EPI_ISL_1123407, EPI_ISL_1123408, EPI_ISL_1123409, EPI_ISL_1123410, EPI_ISL_1123411, EPI_ISL_1123412			
see above	Tokyo Metropolitan Institute of Public Health	Tokyo Metropolitan Institute of Public Health	Masaki Hayashi, Takako Yamazaki, Mami Nagashima,Takushi Fujiwara, Takashi Chiba, Kenji Sadamasu
EPI_ISL_1123418	Pathogen Genomics Center, National Institute of Infectious Diseases	Pathogen Genomics Center, National Institute of Infectious Diseases	Tsuyoshi Sekizuka, Kentaro Itokawa, Rina Tanaka, Masanori Hashino, Makoto Kuroda
EPI_ISL_1123419	Tokyo Metropolitan Institute of Public Health	Pathogen Genomics Center, National Institute of Infectious Diseases	Tsuyoshi Sekizuka, Kentaro Itokawa, Rina Tanaka, Masanori Hashino, Makoto Kuroda
EPI_ISL_1123420, EPI_ISL_1123421, EPI_ISL_1123422, EPI_ISL_1123423	SARS-CoV-2 testing team, National Institute of Infectious Diseases	Pathogen Genomics Center, National Institute of Infectious Diseases	Tsuyoshi Sekizuka, Kentaro Itokawa, Rina Tanaka, Masanori Hashino, Tadashi Kikuchi, Saori Matsuoka, Shigeru Kusagawa, Makoto Kuroda
EPI_ISL_1123424, EPI_ISL_1123425, EPI_ISL_1123426	SARS-CoV-2 testing team, National Institute of Infectious Diseases	Pathogen Genomics Center, National Institute of Infectious Diseases	Tsuyoshi Sekizuka, Kentaro Itokawa, Rina Tanaka, Masanori Hashino, Yoshihiro Kaku, Yasutaka Hoshino, Chikako Shimokawa, Eunsil Park, Tsuguto Fujimoto, Makoto Kuroda
EPI_ISL_1123427, EPI_ISL_1123428, EPI_ISL_1123429, EPI_ISL_1123430, EPI_ISL_1123431, EPI_ISL_1123432, EPI_ISL_1123433, EPI_ISL_1123434	Pathogen Genomics Center, National Institute of Infectious Diseases	Pathogen Genomics Center, National Institute of Infectious Diseases	Tsuyoshi Sekizuka, Kentaro Itokawa, Rina Tanaka, Masanori Hashino, Makoto Kuroda
EPI_ISL_1123435	Gunma Prefectural Institute of Public Health and Environmental Sciences	Pathogen Genomics Center, National Institute of Infectious Diseases	Tsuyoshi Sekizuka, Kentaro Itokawa, Rina Tanaka, Masanori Hashino, Makoto Kuroda
EPI_ISL_1123436	Pathogen Genomics Center, National Institute of Infectious Diseases	Pathogen Genomics Center, National Institute of Infectious Diseases	Tsuyoshi Sekizuka, Kentaro Itokawa, Rina Tanaka, Masanori Hashino, Makoto Kuroda
EPI_ISL_1123437	Gunma Prefectural Institute of Public Health and Environmental Sciences	Pathogen Genomics Center, National Institute of Infectious Diseases	Tsuyoshi Sekizuka, Kentaro Itokawa, Rina Tanaka, Masanori Hashino, Makoto Kuroda
EPI_ISL_1123447	Pathogen Genomics Center, National Institute of Infectious Diseases	Pathogen Genomics Center, National Institute of Infectious Diseases	Tsuyoshi Sekizuka, Kentaro Itokawa, Rina Tanaka, Masanori Hashino, Makoto Kuroda
EPI_ISL_1123448	SARS-CoV-2 testing team, National Institute of Infectious Diseases	Pathogen Genomics Center, National Institute of Infectious Diseases	Tsuyoshi Sekizuka, Kentaro Itokawa, Rina Tanaka, Masanori Hashino, Yoshihiro Kaku, Yasutaka Hoshino, Chikako Shimokawa, Eunsil Park, Tsuguto Fujimoto, Makoto Kuroda
EPI_ISL_1123449	Pathogen Genomics Center, National Institute of Infectious Diseases	Pathogen Genomics Center, National Institute of Infectious Diseases	Tsuyoshi Sekizuka, Kentaro Itokawa, Rina Tanaka, Masanori Hashino, Makoto Kuroda
EPI_ISL_1123451, EPI_ISL_1123452	Gunma Prefectural Institute of Public Health and Environmental Sciences	Pathogen Genomics Center, National Institute of Infectious Diseases	Tsuyoshi Sekizuka, Kentaro Itokawa, Rina Tanaka, Masanori Hashino, Makoto Kuroda
EPI_ISL_1123453, EPI_ISL_1123454, EPI_ISL_1123455, EPI_ISL_1123456, EPI_ISL_1123457	Pathogen Genomics Center, National Institute of Infectious Diseases	Pathogen Genomics Center, National Institute of Infectious Diseases	Tsuyoshi Sekizuka, Kentaro Itokawa, Rina Tanaka, Masanori Hashino, Makoto Kuroda
EPI_ISL_1123458	SARS-CoV-2 testing team, National Institute of Infectious Diseases	Pathogen Genomics Center, National Institute of Infectious Diseases	Tsuyoshi Sekizuka, Kentaro Itokawa, Rina Tanaka, Masanori Hashino, Yoshihiro Kaku, Yasutaka Hoshino, Chikako Shimokawa, Eunsil Park, Tsuguto Fujimoto, Makoto Kuroda
EPI_ISL_1123459, EPI_ISL_1123460, EPI_ISL_1123461, EPI_ISL_1123462, EPI_ISL_1123463, EPI_ISL_1123464, EPI_ISL_1123465, EPI_ISL_1123466	Pathogen Genomics Center, National Institute of Infectious Diseases	Pathogen Genomics Center, National Institute of Infectious Diseases	Tsuyoshi Sekizuka, Kentaro Itokawa, Rina Tanaka, Masanori Hashino, Makoto Kuroda

We gratefully acknowledge the following Authors from the Originating laboratories responsible for obtaining the specimens, as well as the Submitting laboratories where the genome data were generated and shared via GISAID, on which this research is based.

All Submitters of data may be contacted directly via www.gisaid.org

Authors are sorted alphabetically.

Accession ID	Originating Laboratory	Submitting Laboratory	Authors
EPI_ISL_1425080, EPI_ISL_1425081, EPI_ISL_1425082, EPI_ISL_1425083, EPI_ISL_1425084, EPI_ISL_1425085, EPI_ISL_1425086, EPI_ISL_1425087, EPI_ISL_1425088, EPI_ISL_1425089, EPI_ISL_1425090, EPI_ISL_1425091, EPI_ISL_1425092, EPI_ISL_1425093, EPI_ISL_1425094, EPI_ISL_1425095, EPI_ISL_1425096, EPI_ISL_1425097, EPI_ISL_1425098, EPI_ISL_1425099, EPI_ISL_1425100, EPI_ISL_1425101, EPI_ISL_1425102, EPI_ISL_1425103, EPI_ISL_1425104, EPI_ISL_1425105, EPI_ISL_1425106, EPI_ISL_1425107, EPI_ISL_1425108, EPI_ISL_1425109, EPI_ISL_1425110, EPI_ISL_1425111, EPI_ISL_1425112, EPI_ISL_1425113, EPI_ISL_1425114, EPI_ISL_1425115, EPI_ISL_1425116, EPI_ISL_1425117, EPI_ISL_1425118, EPI_ISL_1425119, EPI_ISL_1425120, EPI_ISL_1425121, EPI_ISL_1425122, EPI_ISL_1425123, EPI_ISL_1425124, EPI_ISL_1425125, EPI_ISL_1425126, EPI_ISL_1425127, EPI_ISL_1425128, EPI_ISL_1425129, EPI_ISL_1425130, EPI_ISL_1425131, EPI_ISL_1425132, EPI_ISL_1425133, EPI_ISL_1425134, EPI_ISL_1425135, EPI_ISL_1425136, EPI_ISL_1425137, EPI_ISL_1425138, EPI_ISL_1425139, EPI_ISL_1425140, EPI_ISL_1425141, EPI_ISL_1425142, EPI_ISL_1425143, EPI_ISL_1425144, EPI_ISL_1425145, EPI_ISL_1425146, EPI_ISL_1425147, EPI_ISL_1425148, EPI_ISL_1425149, EPI_ISL_1425150, EPI_ISL_1425151, EPI_ISL_1425152, EPI_ISL_1425153, EPI_ISL_1425154, EPI_ISL_1425155, EPI_ISL_1425156, EPI_ISL_1425157, EPI_ISL_1425158, EPI_ISL_1425159, EPI_ISL_1425160, EPI_ISL_1425161, EPI_ISL_1425162, EPI_ISL_1425163, EPI_ISL_1425164, EPI_ISL_1425165, EPI_ISL_1425166, EPI_ISL_1425167, EPI_ISL_1425168, EPI_ISL_1425169, EPI_ISL_1425170, EPI_ISL_1425171, EPI_ISL_1425172, EPI_ISL_1425173, EPI_ISL_1425174, EPI_ISL_1425175, EPI_ISL_1425176, EPI_ISL_1425177, EPI_ISL_1425178, EPI_ISL_1425179, EPI_ISL_1425180, EPI_ISL_1425181, EPI_ISL_1425182, EPI_ISL_1425183, EPI_ISL_1425184, EPI_ISL_1425185, EPI_ISL_1425186, EPI_ISL_1425187, EPI_ISL_1425188, EPI_ISL_1425189, EPI_ISL_1425190, EPI_ISL_1425191, EPI_ISL_1425192, EPI_ISL_1425193, EPI_ISL_1425194, EPI_ISL_1425195, EPI_ISL_1425196, EPI_ISL_1425197, EPI_ISL_1425198, EPI_ISL_1425199, EPI_ISL_1425200, EPI_ISL_1425201, EPI_ISL_1425202, EPI_ISL_1425203, EPI_ISL_1425204, EPI_ISL_1425205, EPI_ISL_1425206, EPI_ISL_1425207, EPI_ISL_1425208, EPI_ISL_1425209, EPI_ISL_1425210, EPI_ISL_1425211, EPI_ISL_1425212, EPI_ISL_1425213, EPI_ISL_1425214, EPI_ISL_1425215, EPI_ISL_1425216, EPI_ISL_1425217, EPI_ISL_1425218, EPI_ISL_1425219, EPI_ISL_1425220, EPI_ISL_1425221, EPI_ISL_1425222, EPI_ISL_1425223, EPI_ISL_1425224, EPI_ISL_1425225, EPI_ISL_1425226, EPI_ISL_1425227, EPI_ISL_1425228, EPI_ISL_1425229, EPI_ISL_1425230, EPI_ISL_1425231, EPI_ISL_1425232, EPI_ISL_1425233, EPI_ISL_1425234, EPI_ISL_1425235, EPI_ISL_1425236, EPI_ISL_1425237, EPI_ISL_1425238, EPI_ISL_1425239, EPI_ISL_1425240, EPI_ISL_1425241, EPI_ISL_1425242, EPI_ISL_1425243, EPI_ISL_1425244, EPI_ISL_1425245, EPI_ISL_1425246, EPI_ISL_1425247, EPI_ISL_1425248, EPI_ISL_1425249, EPI_ISL_1425250, EPI_ISL_1425251, EPI_ISL_1425252, EPI_ISL_1425253, EPI_ISL_1425254, EPI_ISL_1425255, EPI_ISL_1425256, EPI_ISL_1425257, EPI_ISL_1425258, EPI_ISL_1425259, EPI_ISL_1425260, EPI_ISL_1425261, EPI_ISL_1425262, EPI_ISL_1425263, EPI_ISL_1425264, EPI_ISL_1425265, EPI_ISL_1425266, EPI_ISL_1425267, EPI_ISL_1425268, EPI_ISL_1425269, EPI_ISL_1425270, EPI_ISL_1425271, EPI_ISL_1425272, EPI_ISL_1425273, EPI_ISL_1425274, EPI_ISL_1425275, EPI_ISL_1425276, EPI_ISL_1425277, EPI_ISL_1425278, EPI_ISL_1425279, EPI_ISL_1425280, EPI_ISL_1425281, EPI_ISL_1425282, EPI_ISL_1425283, EPI_ISL_1425284, EPI_ISL_1425285, EPI_ISL_1425286, EPI_ISL_1425287, EPI_ISL_1425288, EPI_ISL_1425289, EPI_ISL_1425290, EPI_ISL_1425291, EPI_ISL_1425292, EPI_ISL_1425293, EPI_ISL_1425294, EPI_ISL_1425295, EPI_ISL_1425296, EPI_ISL_1425297, EPI_ISL_1425298, EPI_ISL_1425299, EPI_ISL_1425300, EPI_ISL_1425301, EPI_ISL_1425302, EPI_ISL_1425303, EPI_ISL_1425304, EPI_ISL_1425305, EPI_ISL_1425306, EPI_ISL_1425307, EPI_ISL_1425308, EPI_ISL_1425309, EPI_ISL_1425310, EPI_ISL_1425311, EPI_ISL_1425312, EPI_ISL_1425313, EPI_ISL_1425314, EPI_ISL_1425315, EPI_ISL_1425316, EPI_ISL_1425317, EPI_ISL_1425318, EPI_ISL_1425319, EPI_ISL_1425320, EPI_ISL_1425321, EPI_ISL_1425322, EPI_ISL_1425323, EPI_ISL_1425324, EPI_ISL_1425325, EPI_ISL_1425326, EPI_ISL_1425327, EPI_ISL_1425328, EPI_ISL_1425329, EPI_ISL_1425330, EPI_ISL_1425331, EPI_ISL_1425332, EPI_ISL_1425333, EPI_ISL_1425334, EPI_ISL_1425335, EPI_ISL_1425336, EPI_ISL_1425337, EPI_ISL_1425338, EPI_ISL_1425339, EPI_ISL_1425340, EPI_ISL_1425341, EPI_ISL_1425342, EPI_ISL_1425343, EPI_ISL_1425344, EPI_ISL_1425345, EPI_ISL_1425346, EPI_ISL_1425347, EPI_ISL_1425348, EPI_ISL_1425349, EPI_ISL_1425350, EPI_ISL_1425351, EPI_ISL_1425352, EPI_ISL_1425353, EPI_ISL_1425354, EPI_ISL_1425355, EPI_ISL_1425356, EPI_ISL_1425357, EPI_ISL_1425358, EPI_ISL_1425359, EPI_ISL_1425360, EPI_ISL_1425361, EPI_ISL_1425362, EPI_ISL_1425363, EPI_ISL_1425364, EPI_ISL_1425365, EPI_ISL_1425366, EPI_ISL_1425367, EPI_ISL_1425368, EPI_ISL_1425369, EPI_ISL_1425370, EPI_ISL_1425371, EPI_ISL_1425372, EPI_ISL_1425373, EPI_ISL_1425374, EPI_ISL_1425375, EPI_ISL_1425376, EPI_ISL_1425377, EPI_ISL_1425378, EPI_ISL_1425379, EPI_ISL_1425380, EPI_ISL_1425381, EPI_ISL_1425382, EPI_ISL_1425383, EPI_ISL_1425384, EPI_ISL_1425385, EPI_ISL_1425386, EPI_ISL_1425387, EPI_ISL_1425388, EPI_ISL_1425389, EPI_ISL_1425390, EPI_ISL_1425391, EPI_ISL_1425392, EPI_ISL_1425393, EPI_ISL_1425394, EPI_ISL_1425395, EPI_ISL_1425396, EPI_ISL_1425397, EPI_ISL_1425398, EPI_ISL_1425399, EPI_ISL_1425400, EPI_ISL_1425401, EPI_ISL_1425402, EPI_ISL_1425403, EPI_ISL_1425404, EPI_ISL_1425405, EPI_ISL_1425406, EPI_ISL_1425407, EPI_ISL_1425408, EPI_ISL_1425409, EPI_ISL_1425410, EPI_ISL_1425411, EPI_ISL_1425412, EPI_ISL_1425413, EPI_ISL_1425414, EPI_ISL_1425415, EPI_ISL_1425416, EPI_ISL_1425417, EPI_ISL_1425418, EPI_ISL_1425419, EPI_ISL_1425420, EPI_ISL_1425421, EPI_ISL_1425422, EPI_ISL_1425423, EPI_ISL_1425424, EPI_ISL_1425425, EPI_ISL_1425426, EPI_ISL_1425427, EPI_ISL_1425428, EPI_ISL_1425429, EPI_ISL_1425430, EPI_ISL_1425431, EPI_ISL_1425432, EPI_ISL_1425433, EPI_ISL_1425434, EPI_ISL_1425435, EPI_ISL_1425436, EPI_ISL_1425437, EPI_ISL_1425438, EPI_ISL_1425439, EPI_ISL_1425440, EPI_ISL_1425441, EPI_ISL_1425442, EPI_ISL_1425443, EPI_ISL_1425444, EPI_ISL_1425445, EPI_ISL_1425446, EPI_ISL_1425447, EPI_ISL_1425448, EPI_ISL_1425449, EPI_ISL_1425450, EPI_ISL_1425451, EPI_ISL_1425452, EPI_ISL_1425453, EPI_ISL_1425454, EPI_ISL_1425455, EPI_ISL_1425456, EPI_ISL_1425457, EPI_ISL_1425458, EPI_ISL_1425459, EPI_ISL_1425460, EPI_ISL_1425461, EPI_ISL_1425462, EPI_ISL_1425463, EPI_ISL_1425464, EPI_ISL_1425465, EPI_ISL_1425466, EPI_ISL_1425467, EPI_ISL_1425468, EPI_ISL_1425469, EPI_ISL_1425470, EPI_ISL_1425471, EPI_ISL_1425472, EPI_ISL_1425473, EPI_ISL_1425474, EPI_ISL_1425475, EPI_ISL_1425476, EPI_ISL_1425477, EPI_ISL_1425478, EPI_ISL_1425479, EPI_ISL_1425480, EPI_ISL_1425481, EPI_ISL_1425482, EPI_ISL_1425483, EPI_ISL_1425484, EPI_ISL_1425485, EPI_ISL_1425486, EPI_ISL_1425487, EPI_ISL_1425488, EPI_ISL_1425489, EPI_ISL_1425490, EPI_ISL_1425491, EPI_ISL_1425492, EPI_ISL_1425493, EPI_ISL_1425494, EPI_ISL_1425495, EPI_ISL_1425496, EPI_ISL_1425497, EPI_ISL_1425498, EPI_ISL_1425499, EPI_ISL_1425500, EPI_ISL_1425501, EPI_ISL_1425502, EPI_ISL_1425503, EPI_ISL_1425504, EPI_ISL_1425505, EPI_ISL_1425506, EPI_ISL_1425507, EPI_ISL_1425508, EPI_ISL_1425509, EPI_ISL_1425510, EPI_ISL_1425511, EPI_ISL_1425512, EPI_ISL_1425513, EPI_ISL_1425514, EPI_ISL_1425515, EPI_ISL_1425516, EPI_ISL_1425517, EPI_ISL_1425518, EPI_ISL_1425519, EPI_ISL_1425520, EPI_ISL_1425521, EPI_ISL_1425522, EPI_ISL_1425523, EPI_ISL_1425524, EPI_ISL_1425525, EPI_ISL_1425526, EPI_ISL_1425527, EPI_ISL_1425528, EPI_ISL_1425529, EPI_ISL_1425530, EPI_ISL_1425531, EPI_ISL_1425532, EPI_ISL_1425533, EPI_ISL_1425534, EPI_ISL_1425535, EPI_ISL_1425536, EPI_ISL_1425537, EPI_ISL_1425538, EPI_ISL_1425539, EPI_ISL_1425540			
see above	Department of Infectious Diseases, Kobe Institute of Health	Department of Infectious Diseases, Kobe Institute of Health	Ryohei Nomoto, Noriko Nakanishi, Tomotada Iwamoto, Tsuyoshi Sekizuka, Kentaro Itokawa, Rina Tanaka, Masanori Hashino, Makoto Kuroda
EPI_ISL_1425568	Gunma Prefectural Institute of Public Health and Environmental Sciences	Pathogen Genomics Center, National Institute of Infectious Diseases	Tsuyoshi Sekizuka, Kentaro Itokawa, Rina Tanaka, Masanori Hashino, Makoto Kuroda
EPI_ISL_1425573, EPI_ISL_1425574, EPI_ISL_1425575	SARS-CoV-2 testing team, National Institute of Infectious Diseases	Pathogen Genomics Center, National Institute of Infectious Diseases	Tsuyoshi Sekizuka, Kentaro Itokawa, Rina Tanaka, Masanori Hashino, Yoshihiro Kaku, Yasutaka Hoshino, Chikako Shimokawa, Eunsil Park, Tsuguto Fujimoto, Makoto Kuroda
EPI_ISL_1425576	SARS-CoV-2 testing team, National Institute of Infectious Diseases	Pathogen Genomics Center, National Institute of Infectious Diseases	Tsuyoshi Sekizuka, Kentaro Itokawa, Rina Tanaka, Masanori Hashino, Eri Nakayama, Motohiko Ogawa, Takahiro Maeki, Shigeru Tanjima, Chang-Kweng Lim, Makoto Kuroda
EPI_ISL_1425577, EPI_ISL_1425578	SARS-CoV-2 testing team, National Institute of Infectious Diseases	Pathogen Genomics Center, National Institute of Infectious Diseases	Tsuyoshi Sekizuka, Kentaro Itokawa, Rina Tanaka, Masanori Hashino, Chang-Kweng Lim, Shigeru Tanjima, Takahiro Maeki, Motohiko Ogawa, Eri Nakayama , Makoto Kuroda
EPI_ISL_1425579, EPI_ISL_1425580, EPI_ISL_1425581, EPI_ISL_1425582, EPI_ISL_1425583, EPI_ISL_1425584, EPI_ISL_1425585	Saitama Prefectural Institute of Public Health	Pathogen Genomics Center, National Institute of Infectious Diseases	Tsuyoshi Sekizuka, Kentaro Itokawa, Rina Tanaka, Masanori Hashino, Minoru Nagi, Ken Miyazawa, Takashi Sakudoh, Nozomu Hanaoka, Tsuguto Fujimoto, Makoto Kuroda
EPI_ISL_1425586	SARS-CoV-2 testing team, National Institute of Infectious Diseases	Pathogen Genomics Center, National Institute of Infectious Diseases	Tsuyoshi Sekizuka, Kentaro Itokawa, Rina Tanaka, Masanori Hashino, Minoru Nagi, Ken Miyazawa, Takashi Sakudoh, Nozomu Hanaoka, Tsuguto Fujimoto, Makoto Kuroda
EPI_ISL_1425587	Saitama Prefectural Institute of Public Health	Pathogen Genomics Center, National Institute of Infectious Diseases	Tsuyoshi Sekizuka, Kentaro Itokawa, Rina Tanaka, Masanori Hashino, Shigeru Tanjima, Eri Nakayama, Takahiro Maeki, Motohiko Ogawa, Chang-Kweng Lim, Makoto Kuroda
EPI_ISL_1425588, EPI_ISL_1425589	SARS-CoV-2 testing team, National Institute of Infectious Diseases	Pathogen Genomics Center, National Institute of Infectious Diseases	Tsuyoshi Sekizuka, Kentaro Itokawa, Rina Tanaka, Masanori Hashino, Shigeru Tanjima, Eri Nakayama, Takahiro Maeki, Motohiko Ogawa, Chang-Kweng Lim, Makoto Kuroda
EPI_ISL_1425590, EPI_ISL_1425591, EPI_ISL_1425592, EPI_ISL_1425593, EPI_ISL_1425594, EPI_ISL_1425595, EPI_ISL_1425596, EPI_ISL_1425597, EPI_ISL_1425598, EPI_ISL_1425599, EPI_ISL_1425600			
see above	SARS-CoV-2 testing team, National Institute of Infectious Diseases	Pathogen Genomics Center, National Institute of Infectious Diseases	Tsuyoshi Sekizuka, Kentaro Itokawa, Rina Tanaka, Masanori Hashino, Tadashi Kikuchi, Hiroshi Ishii, Shigeru Kusagawa, Makoto Kuroda
EPI_ISL_1425601, EPI_ISL_1425602, EPI_ISL_1425603, EPI_ISL_1425604	Pathogen Genomics Center, National Institute of Infectious Diseases	Pathogen Genomics Center, National Institute of Infectious Diseases	Tsuyoshi Sekizuka, Kentaro Itokawa, Rina Tanaka, Masanori Hashino, Makoto Kuroda
EPI_ISL_1425605	SARS-CoV-2 testing team, National Institute of Infectious Diseases	Pathogen Genomics Center, National Institute of Infectious Diseases	Tsuyoshi Sekizuka, Kentaro Itokawa, Rina Tanaka, Masanori Hashino, Eri Nakayama, Motohiko Ogawa, Takahiro Maeki, Shigeru Tanjima, Chang-Kweng Lim, Makoto Kuroda
EPI_ISL_1425606	SARS-CoV-2 testing team, National Institute of Infectious Diseases	Pathogen Genomics Center, National Institute of Infectious Diseases	Tsuyoshi Sekizuka, Kentaro Itokawa, Rina Tanaka, Masanori Hashino, Yoshihiro Kaku, Yasutaka Hoshino, Chikako Shimokawa, Eunsil Park, Tsuguto Fujimoto, Makoto Kuroda
EPI_ISL_1425607, EPI_ISL_1425608, EPI_ISL_1425609, EPI_ISL_1425610, EPI_ISL_1425611, EPI_ISL_1425612, EPI_ISL_1425613	SARS-CoV-2 testing team, National Institute of Infectious Diseases	Pathogen Genomics Center, National Institute of Infectious Diseases	Tsuyoshi Sekizuka, Kentaro Itokawa, Rina Tanaka, Masanori Hashino, Eri Nakayama, Motohiko Ogawa, Takahiro Maeki, Shigeru Tanjima, Chang-Kweng Lim, Makoto Kuroda
EPI_ISL_1425614	Pathogen Genomics Center, National Institute of Infectious Diseases	Pathogen Genomics Center, National Institute of Infectious Diseases	Tsuyoshi Sekizuka, Kentaro Itokawa, Rina Tanaka, Masanori Hashino, Makoto Kuroda
EPI_ISL_1425615	SARS-CoV-2 testing team, National Institute of Infectious Diseases	Pathogen Genomics Center, National Institute of Infectious Diseases	Tsuyoshi Sekizuka, Kentaro Itokawa, Rina Tanaka, Masanori Hashino, Koichi Ishikawa, Sayuri Seki, Shigeru Kusagawa, Makoto Kuroda
EPI_ISL_1425616	Pathogen Genomics Center, National Institute of Infectious Diseases	Pathogen Genomics Center, National Institute of Infectious Diseases	Tsuyoshi Sekizuka, Kentaro Itokawa, Rina Tanaka, Masanori Hashino, Makoto Kuroda

We gratefully acknowledge the following Authors from the Originating laboratories responsible for obtaining the specimens, as well as the Submitting laboratories where the genome data were generated and shared via GISAID, on which this research is based.

All Submitters of data may be contacted directly via www.gisaid.org

Authors are sorted alphabetically.

Accession ID	Originating Laboratory	Submitting Laboratory	Authors
EPI_ISL_1922003, EPI_ISL_1922004, EPI_ISL_1922005, EPI_ISL_1922006, EPI_ISL_1922007, EPI_ISL_1922008, EPI_ISL_1922009, EPI_ISL_1922010, EPI_ISL_1922011, EPI_ISL_1922012, EPI_ISL_1922013, EPI_ISL_1922014, EPI_ISL_1922015, EPI_ISL_1922016, EPI_ISL_1922017, EPI_ISL_1922018, EPI_ISL_1922019, EPI_ISL_1922020, EPI_ISL_1922021, EPI_ISL_1922022, EPI_ISL_1922023, EPI_ISL_1922024, EPI_ISL_1922025, EPI_ISL_1922026, EPI_ISL_1922027, EPI_ISL_1922028, EPI_ISL_1922029, EPI_ISL_1922030, EPI_ISL_1922031, EPI_ISL_1922032, EPI_ISL_1922033, EPI_ISL_1922034, EPI_ISL_1922035, EPI_ISL_1922036, EPI_ISL_1922037, EPI_ISL_1922038, EPI_ISL_1922039, EPI_ISL_1922040, EPI_ISL_1922041			
see above	Gunma Prefectural Institute of Public Health and Environmental Sciences	Gunma Prefectural Institute of Public Health and Environmental Sciences	Tsuyoshi Sekizuka, Kentaro Itokawa, Rina Tanaka, Masanori Hashino, Makoto Kuroda, Hiroyuki Tsukagoshi, Daisuke Shinoda, Mariko Saito
EPI_ISL_1926888, EPI_ISL_1926890	SARS-CoV-2 testing team, National Institute of Infectious Diseases	Pathogen Genomics Center, National Institute of Infectious Diseases	Tsuyoshi Sekizuka, Kentaro Itokawa, Rina Tanaka, Masanori Hashino, Hidemasa Izumiya, Sunao Iyoda, Shouji Yamamoto, Masatomo Morita, Ken-ichi Lee, Nobuo Koizumi, Makoto Kuroda
EPI_ISL_1926893, EPI_ISL_1926894, EPI_ISL_1926896	SARS-CoV-2 testing team, National Institute of Infectious Diseases	Pathogen Genomics Center, National Institute of Infectious Diseases	Tsuyoshi Sekizuka, Kentaro Itokawa, Rina Tanaka, Masanori Hashino, Ai Kawana-Tachikawa, Midori Nakamura-Hoshi, Shigeru Kusagawa, Makoto Kuroda
EPI_ISL_1926898, EPI_ISL_1926899, EPI_ISL_1926901, EPI_ISL_1926902, EPI_ISL_1926903, EPI_ISL_1926905	SARS-CoV-2 testing team, National Institute of Infectious Diseases	Pathogen Genomics Center, National Institute of Infectious Diseases	Tsuyoshi Sekizuka, Kentaro Itokawa, Rina Tanaka, Masanori Hashino, Koichi Ishikawa, Sayuri Seki, Shigeru Kusagawa, Makoto Kuroda
EPI_ISL_1926906, EPI_ISL_1926908, EPI_ISL_1926909	SARS-CoV-2 testing team, National Institute of Infectious Diseases	Pathogen Genomics Center, National Institute of Infectious Diseases	Tsuyoshi Sekizuka, Kentaro Itokawa, Rina Tanaka, Masanori Hashino, Hidemasa Izumiya, Sunao Iyoda, Ken Shimuta, Nobuo Koizumi, Makoto Kuroda
EPI_ISL_1926911, EPI_ISL_1926912, EPI_ISL_1926914, EPI_ISL_1926915, EPI_ISL_1926917, EPI_ISL_1926918, EPI_ISL_1926919, EPI_ISL_1926921, EPI_ISL_1926922, EPI_ISL_1926924, EPI_ISL_1926925, EPI_ISL_1926927, EPI_ISL_1926929, EPI_ISL_1926930, EPI_ISL_1926931, EPI_ISL_1926933, EPI_ISL_1926934, EPI_ISL_1926936, EPI_ISL_1926937, EPI_ISL_1926939, EPI_ISL_1926941, EPI_ISL_1926942, EPI_ISL_1926944, EPI_ISL_1926945, EPI_ISL_1926946, EPI_ISL_1926948, EPI_ISL_1926949, EPI_ISL_1926951, EPI_ISL_1926952, EPI_ISL_1926954, EPI_ISL_1926955, EPI_ISL_1926957, EPI_ISL_1926958, EPI_ISL_1926960, EPI_ISL_1926961, EPI_ISL_1926962			
see above	SARS-CoV-2 testing team, National Institute of Infectious Diseases	Pathogen Genomics Center, National Institute of Infectious Diseases	Tsuyoshi Sekizuka, Kentaro Itokawa, Rina Tanaka, Masanori Hashino, Nozomu Hanaoka, Masumichi Saito, Naomi Nojiri, Hazuka Y Furihata, Sana Uchikoba, Tsuguto Fujimoto, Makoto Kuroda
EPI_ISL_1926963, EPI_ISL_1926964, EPI_ISL_1926965, EPI_ISL_1926966, EPI_ISL_1926967	SARS-CoV-2 testing team, National Institute of Infectious Diseases	Pathogen Genomics Center, National Institute of Infectious Diseases	Tsuyoshi Sekizuka, Kentaro Itokawa, Rina Tanaka, Masanori Hashino, Hidemasa Izumiya, Sunao Iyoda, Shouji Yamamoto, Masatomo Morita, Ken-ichi Lee, Nobuo Koizumi, Makoto Kuroda
EPI_ISL_1926969	Pathogen Genomics Center, National Institute of Infectious Diseases	Pathogen Genomics Center, National Institute of Infectious Diseases	Tsuyoshi Sekizuka, Kentaro Itokawa, Rina Tanaka, Masanori Hashino, Shigeru Tajima, Takahiro Maeki, Eri Nakayama, Motohiko Ogawa, Chang-Kweng Lim, Makoto Kuroda
EPI_ISL_1926970, EPI_ISL_1926972, EPI_ISL_1926973, EPI_ISL_1926975	Osaka Institute of Public Health, Morinomiya Center	Pathogen Genomics Center, National Institute of Infectious Diseases	Tsuyoshi Sekizuka, Kentaro Itokawa, Rina Tanaka, Masanori Hashino, Shigeru Tajima, Takahiro Maeki, Eri Nakayama, Motohiko Ogawa, Chang-Kweng Lim, Makoto Kuroda
EPI_ISL_1926976, EPI_ISL_1926978	Pathogen Genomics Center, National Institute of Infectious Diseases	Pathogen Genomics Center, National Institute of Infectious Diseases	Tsuyoshi Sekizuka, Kentaro Itokawa, Rina Tanaka, Masanori Hashino, Shigeru Tajima, Takahiro Maeki, Eri Nakayama, Motohiko Ogawa, Chang-Kweng Lim, Makoto Kuroda
EPI_ISL_1926979, EPI_ISL_1926981, EPI_ISL_1926982, EPI_ISL_1926984	Osaka Institute of Public Health, Morinomiya Center	Pathogen Genomics Center, National Institute of Infectious Diseases	Tsuyoshi Sekizuka, Kentaro Itokawa, Rina Tanaka, Masanori Hashino, Shigeru Tajima, Takahiro Maeki, Eri Nakayama, Motohiko Ogawa, Chang-Kweng Lim, Makoto Kuroda
EPI_ISL_1926985	Pathogen Genomics Center, National Institute of Infectious Diseases	Pathogen Genomics Center, National Institute of Infectious Diseases	Tsuyoshi Sekizuka, Kentaro Itokawa, Rina Tanaka, Masanori Hashino, Shigeru Tajima, Takahiro Maeki, Eri Nakayama, Motohiko Ogawa, Chang-Kweng Lim, Makoto Kuroda
EPI_ISL_1926987, EPI_ISL_1926988	Osaka Institute of Public Health, Morinomiya Center	Pathogen Genomics Center, National Institute of Infectious Diseases	Tsuyoshi Sekizuka, Kentaro Itokawa, Rina Tanaka, Masanori Hashino, Shigeru Tajima, Takahiro Maeki, Eri Nakayama, Motohiko Ogawa, Chang-Kweng Lim, Makoto Kuroda
EPI_ISL_1926990, EPI_ISL_1926992, EPI_ISL_1926993, EPI_ISL_1926994, EPI_ISL_1926996, EPI_ISL_1926997	Pathogen Genomics Center, National Institute of Infectious Diseases	Pathogen Genomics Center, National Institute of Infectious Diseases	Tsuyoshi Sekizuka, Kentaro Itokawa, Rina Tanaka, Masanori Hashino, Shigeru Tajima, Takahiro Maeki, Eri Nakayama, Motohiko Ogawa, Chang-Kweng Lim, Makoto Kuroda
EPI_ISL_1926999	Pathogen Genomics Center, National Institute of Infectious Diseases	Pathogen Genomics Center, National Institute of Infectious Diseases	Tsuyoshi Sekizuka, Kentaro Itokawa, Rina Tanaka, Masanori Hashino, Ai Kawana-Tachikawa, Midori Nakamura-Hoshi, Shigeru Kusagawa, Makoto Kuroda
EPI_ISL_1927000, EPI_ISL_1927002, EPI_ISL_1927004	Osaka Institute of Public Health, Morinomiya Center	Pathogen Genomics Center, National Institute of Infectious Diseases	Tsuyoshi Sekizuka, Kentaro Itokawa, Rina Tanaka, Masanori Hashino, Ai Kawana-Tachikawa, Midori Nakamura-Hoshi, Shigeru Kusagawa, Makoto Kuroda
EPI_ISL_1927005, EPI_ISL_1927007, EPI_ISL_1927008, EPI_ISL_1927010, EPI_ISL_1927011, EPI_ISL_1927013, EPI_ISL_1927014, EPI_ISL_1927016, EPI_ISL_1927017, EPI_ISL_1927019, EPI_ISL_1927020, EPI_ISL_1927022			
see above	Pathogen Genomics Center, National Institute of Infectious Diseases	Pathogen Genomics Center, National Institute of Infectious Diseases	Tsuyoshi Sekizuka, Kentaro Itokawa, Rina Tanaka, Masanori Hashino, Ai Kawana-Tachikawa, Midori Nakamura-Hoshi, Shigeru Kusagawa, Makoto Kuroda
EPI_ISL_1927023, EPI_ISL_1927024, EPI_ISL_1927026, EPI_ISL_1927027	Osaka Institute of Public Health, Morinomiya Center	Pathogen Genomics Center, National Institute of Infectious Diseases	Tsuyoshi Sekizuka, Kentaro Itokawa, Rina Tanaka, Masanori Hashino, Ai Kawana-Tachikawa, Midori Nakamura-Hoshi, Shigeru Kusagawa, Makoto Kuroda
EPI_ISL_1927029, EPI_ISL_1927030	Pathogen Genomics Center, National Institute of Infectious Diseases	Pathogen Genomics Center, National Institute of Infectious Diseases	Tsuyoshi Sekizuka, Kentaro Itokawa, Rina Tanaka, Masanori Hashino, Ai Kawana-Tachikawa, Midori Nakamura-Hoshi, Shigeru Kusagawa, Makoto Kuroda
EPI_ISL_1927032, EPI_ISL_1927034, EPI_ISL_1927035	Osaka Institute of Public Health, Morinomiya Center	Pathogen Genomics Center, National Institute of Infectious Diseases	Tsuyoshi Sekizuka, Kentaro Itokawa, Rina Tanaka, Masanori Hashino, Koichi Ishikawa, Sayuri Seki, Shigeru Kusagawa, Makoto Kuroda
EPI_ISL_1927037, EPI_ISL_1927038, EPI_ISL_1927039, EPI_ISL_1927041, EPI_ISL_1927042	Tokyo Metropolitan Institute of Public Health	Pathogen Genomics Center, National Institute of Infectious Diseases	Tsuyoshi Sekizuka, Kentaro Itokawa, Rina Tanaka, Masanori Hashino, Koichi Ishikawa, Sayuri Seki, Shigeru Kusagawa, Makoto Kuroda
EPI_ISL_1927044, EPI_ISL_1927045, EPI_ISL_1927047	Pathogen Genomics Center, National Institute of Infectious Diseases	Pathogen Genomics Center, National Institute of Infectious Diseases	Tsuyoshi Sekizuka, Kentaro Itokawa, Rina Tanaka, Masanori Hashino, Koichi Ishikawa, Sayuri Seki, Shigeru Kusagawa, Makoto Kuroda
EPI_ISL_1927048	Pathogen Genomics Center, National Institute of Infectious Diseases	Pathogen Genomics Center, National Institute of Infectious Diseases	Tsuyoshi Sekizuka, Kentaro Itokawa, Rina Tanaka, Masanori Hashino, Makoto Kuroda
EPI_ISL_1927050, EPI_ISL_1927051, EPI_ISL_1927053, EPI_ISL_1927054, EPI_ISL_1927056, EPI_ISL_1927058, EPI_ISL_1927059, EPI_ISL_1927061, EPI_ISL_1927062, EPI_ISL_1927064, EPI_ISL_1927065, EPI_ISL_1927067, EPI_ISL_1927068			
see above	Pathogen Genomics Center, National Institute of Infectious Diseases	Pathogen Genomics Center, National Institute of Infectious Diseases	Tsuyoshi Sekizuka, Kentaro Itokawa, Rina Tanaka, Masanori Hashino, Koichi Ishikawa, Sayuri Seki, Shigeru Kusagawa, Makoto Kuroda
EPI_ISL_1927069, EPI_ISL_1927071,	Osaka Institute of Public Health, Morinomiya Center	Pathogen Genomics Center, National Institute of Infectious	Tsuyoshi Sekizuka, Kentaro Itokawa, Rina Tanaka, Masanori Hashino, Koichi Ishikawa, Sayuri Seki, Shigeru Kusagawa, Makoto Kuroda

We gratefully acknowledge the following Authors from the Originating laboratories responsible for obtaining the specimens, as well as the Submitting laboratories where the genome data were generated and shared via GISAID, on which this research is based.

All Submitters of data may be contacted directly via www.gisaid.org

Authors are sorted alphabetically.

Accession ID	Originating Laboratory	Submitting Laboratory	Authors
EPI_ISL_2325185, EPI_ISL_2325186, EPI_ISL_2325187, EPI_ISL_2325188, EPI_ISL_2325189, EPI_ISL_2325190, EPI_ISL_2325191, EPI_ISL_2325192, EPI_ISL_2325193, EPI_ISL_2325194, EPI_ISL_2325195, EPI_ISL_2325196, EPI_ISL_2325197, EPI_ISL_2325198, EPI_ISL_2325199, EPI_ISL_2325200, EPI_ISL_2325201, EPI_ISL_2325202, EPI_ISL_2325203, EPI_ISL_2325204, EPI_ISL_2325205, EPI_ISL_2325206, EPI_ISL_2325207, EPI_ISL_2325208, EPI_ISL_2325209, EPI_ISL_2325210, EPI_ISL_2325211, EPI_ISL_2325212, EPI_ISL_2325213, EPI_ISL_2325214, EPI_ISL_2325215, EPI_ISL_2325216, EPI_ISL_2325217, EPI_ISL_2325218, EPI_ISL_2325219, EPI_ISL_2325220, EPI_ISL_2325221, EPI_ISL_2325222, EPI_ISL_2325223, EPI_ISL_2325224, EPI_ISL_2325225, EPI_ISL_2325226, EPI_ISL_2325227, EPI_ISL_2325228, EPI_ISL_2325229, EPI_ISL_2325230, EPI_ISL_2325231, EPI_ISL_2325232, EPI_ISL_2325233, EPI_ISL_2325234, EPI_ISL_2325235, EPI_ISL_2325236, EPI_ISL_2325237, EPI_ISL_2325238, EPI_ISL_2325239, EPI_ISL_2325240, EPI_ISL_2325241, EPI_ISL_2325242, EPI_ISL_2325243, EPI_ISL_2325244, EPI_ISL_2325245, EPI_ISL_2325246, EPI_ISL_2325247, EPI_ISL_2325248, EPI_ISL_2325249, EPI_ISL_2325250, EPI_ISL_2325251, EPI_ISL_2325252, EPI_ISL_2325253, EPI_ISL_2325254, EPI_ISL_2325255, EPI_ISL_2325256, EPI_ISL_2325257, EPI_ISL_2325258, EPI_ISL_2325259, EPI_ISL_2325260, EPI_ISL_2325261, EPI_ISL_2325262, EPI_ISL_2325263, EPI_ISL_2325264, EPI_ISL_2325265, EPI_ISL_2325266, EPI_ISL_2325267, EPI_ISL_2325268, EPI_ISL_2325269, EPI_ISL_2325270, EPI_ISL_2325271, EPI_ISL_2325272, EPI_ISL_2325273, EPI_ISL_2325274, EPI_ISL_2325275, EPI_ISL_2325276, EPI_ISL_2325277, EPI_ISL_2325278, EPI_ISL_2325279, EPI_ISL_2325280, EPI_ISL_2325281, EPI_ISL_2325282, EPI_ISL_2325283, EPI_ISL_2325284, EPI_ISL_2325285, EPI_ISL_2325286, EPI_ISL_2325287, EPI_ISL_2325288, EPI_ISL_2325289, EPI_ISL_2325290, EPI_ISL_2325291, EPI_ISL_2325292, EPI_ISL_2325293, EPI_ISL_2325294, EPI_ISL_2325295, EPI_ISL_2325296, EPI_ISL_2325297, EPI_ISL_2325298, EPI_ISL_2325299, EPI_ISL_2325300, EPI_ISL_2325301, EPI_ISL_2325302, EPI_ISL_2325303, EPI_ISL_2325304, EPI_ISL_2325305, EPI_ISL_2325306, EPI_ISL_2325307, EPI_ISL_2325308, EPI_ISL_2325309, EPI_ISL_2325310, EPI_ISL_2325311, EPI_ISL_2325312, EPI_ISL_2325313, EPI_ISL_2325314, EPI_ISL_2325315, EPI_ISL_2325316, EPI_ISL_2325317, EPI_ISL_2325318, EPI_ISL_2325319, EPI_ISL_2325320, EPI_ISL_2325321, EPI_ISL_2325322, EPI_ISL_2325323, EPI_ISL_2325324, EPI_ISL_2325325, EPI_ISL_2325326, EPI_ISL_2325327, EPI_ISL_2325328, EPI_ISL_2325329, EPI_ISL_2325330, EPI_ISL_2325331, EPI_ISL_2325332, EPI_ISL_2325333, EPI_ISL_2325334, EPI_ISL_2325335, EPI_ISL_2325336, EPI_ISL_2325337, EPI_ISL_2325338, EPI_ISL_2325339, EPI_ISL_2325340, EPI_ISL_2325341, EPI_ISL_2325342, EPI_ISL_2325343, EPI_ISL_2325344, EPI_ISL_2325345, EPI_ISL_2325346, EPI_ISL_2325347, EPI_ISL_2325348, EPI_ISL_2325349, EPI_ISL_2325350, EPI_ISL_2325351, EPI_ISL_2325352, EPI_ISL_2325353, EPI_ISL_2325354, EPI_ISL_2325355, EPI_ISL_2325356, EPI_ISL_2325357, EPI_ISL_2325358, EPI_ISL_2325359, EPI_ISL_2325360, EPI_ISL_2325361, EPI_ISL_2325362, EPI_ISL_2325363, EPI_ISL_2325364, EPI_ISL_2325365, EPI_ISL_2325366, EPI_ISL_2325367, EPI_ISL_2325368, EPI_ISL_2325369, EPI_ISL_2325370, EPI_ISL_2325371, EPI_ISL_2325372, EPI_ISL_2325373, EPI_ISL_2325374, EPI_ISL_2325375, EPI_ISL_2325376, EPI_ISL_2325377, EPI_ISL_2325378, EPI_ISL_2325379, EPI_ISL_2325380, EPI_ISL_2325381, EPI_ISL_2325382, EPI_ISL_2325383, EPI_ISL_2325384, EPI_ISL_2325385, EPI_ISL_2325386, EPI_ISL_2325387, EPI_ISL_2325388, EPI_ISL_2325389, EPI_ISL_2325390, EPI_ISL_2325391, EPI_ISL_2325392, EPI_ISL_2325393, EPI_ISL_2325394, EPI_ISL_2325395, EPI_ISL_2325396, EPI_ISL_2325397, EPI_ISL_2325398, EPI_ISL_2325399, EPI_ISL_2325400, EPI_ISL_2325401, EPI_ISL_2325402, EPI_ISL_2325403, EPI_ISL_2325404, EPI_ISL_2325405, EPI_ISL_2325406, EPI_ISL_2325407, EPI_ISL_2325408, EPI_ISL_2325409, EPI_ISL_2325410, EPI_ISL_2325411, EPI_ISL_2325412, EPI_ISL_2325413, EPI_ISL_2325414, EPI_ISL_2325415, EPI_ISL_2325416, EPI_ISL_2325417, EPI_ISL_2325418, EPI_ISL_2325419, EPI_ISL_2325420, EPI_ISL_2325421, EPI_ISL_2325422, EPI_ISL_2325423, EPI_ISL_2325424, EPI_ISL_2325425, EPI_ISL_2325426, EPI_ISL_2325427, EPI_ISL_2325428, EPI_ISL_2325429, EPI_ISL_2325430, EPI_ISL_2325431, EPI_ISL_2325432, EPI_ISL_2325433, EPI_ISL_2325434, EPI_ISL_2325435, EPI_ISL_2325436, EPI_ISL_2325437, EPI_ISL_2325438, EPI_ISL_2325439, EPI_ISL_2325440, EPI_ISL_2325441, EPI_ISL_2325442, EPI_ISL_2325443, EPI_ISL_2325444, EPI_ISL_2325445, EPI_ISL_2325446, EPI_ISL_2325447, EPI_ISL_2325448, EPI_ISL_2325449, EPI_ISL_2325450, EPI_ISL_2325451, EPI_ISL_2325452, EPI_ISL_2325453, EPI_ISL_2325454, EPI_ISL_2325455, EPI_ISL_2325456, EPI_ISL_2325457, EPI_ISL_2325458, EPI_ISL_2325459, EPI_ISL_2325460, EPI_ISL_2325461, EPI_ISL_2325462, EPI_ISL_2325463, EPI_ISL_2325464, EPI_ISL_2325465, EPI_ISL_2325466, EPI_ISL_2325467, EPI_ISL_2325468, EPI_ISL_2325469, EPI_ISL_2325470, EPI_ISL_2325471, EPI_ISL_2325472, EPI_ISL_2325473, EPI_ISL_2325474, EPI_ISL_2325475, EPI_ISL_2325476, EPI_ISL_2325477, EPI_ISL_2325478, EPI_ISL_2325479, EPI_ISL_2325480, EPI_ISL_2325481, EPI_ISL_2325483, EPI_ISL_2325484, EPI_ISL_2325485, EPI_ISL_2325486, EPI_ISL_2325487, EPI_ISL_2325488, EPI_ISL_2325489, EPI_ISL_2325490, EPI_ISL_2325491, EPI_ISL_2325492, EPI_ISL_2325493, EPI_ISL_2325494, EPI_ISL_2325495, EPI_ISL_2325496, EPI_ISL_2325497, EPI_ISL_2325498, EPI_ISL_2325499, EPI_ISL_2325500, EPI_ISL_2325501, EPI_ISL_2325502, EPI_ISL_2325503, EPI_ISL_2325504, EPI_ISL_2325505, EPI_ISL_2325506, EPI_ISL_2325507, EPI_ISL_2325508, EPI_ISL_2325509, EPI_ISL_2325510, EPI_ISL_2325511, EPI_ISL_2325512, EPI_ISL_2325513, EPI_ISL_2325514, EPI_ISL_2325515, EPI_ISL_2325516, EPI_ISL_2325517, EPI_ISL_2325518, EPI_ISL_2325519, EPI_ISL_2325520, EPI_ISL_2325521, EPI_ISL_2325522, EPI_ISL_2325523, EPI_ISL_2325524, EPI_ISL_2325525, EPI_ISL_2325526, EPI_ISL_2325527, EPI_ISL_2325528, EPI_ISL_2325529, EPI_ISL_2325530, EPI_ISL_2325531, EPI_ISL_2325532, EPI_ISL_2325533, EPI_ISL_2325534, EPI_ISL_2325535, EPI_ISL_2325536, EPI_ISL_2325537, EPI_ISL_2325538, EPI_ISL_2325539, EPI_ISL_2325540, EPI_ISL_2325541, EPI_ISL_2325542, EPI_ISL_2325543, EPI_ISL_2325544, EPI_ISL_2325545, EPI_ISL_2325546, EPI_ISL_2325547, EPI_ISL_2325548, EPI_ISL_2325549, EPI_ISL_2325550, EPI_ISL_2325551, EPI_ISL_2325552, EPI_ISL_2325553, EPI_ISL_2325554, EPI_ISL_2325555, EPI_ISL_2325556, EPI_ISL_2325557, EPI_ISL_2325558, EPI_ISL_2325559, EPI_ISL_2325560, EPI_ISL_2325561, EPI_ISL_2325562, EPI_ISL_2325563, EPI_ISL_2325564, EPI_ISL_2325565, EPI_ISL_2325566, EPI_ISL_2325567, EPI_ISL_2325568, EPI_ISL_2325569, EPI_ISL_2325570, EPI_ISL_2325571, EPI_ISL_2325572, EPI_ISL_2325573, EPI_ISL_2325574, EPI_ISL_2325575, EPI_ISL_2325576, EPI_ISL_2325577, EPI_ISL_2325578, EPI_ISL_2325580, EPI_ISL_2325581, EPI_ISL_2325582, EPI_ISL_2325583, EPI_ISL_2325584, EPI_ISL_2325585, EPI_ISL_2325586, EPI_ISL_2325587, EPI_ISL_2325588, EPI_ISL_2325589, EPI_ISL_2325590, EPI_ISL_2325591, EPI_ISL_2325592, EPI_ISL_2325593, EPI_ISL_2325594, EPI_ISL_2325595, EPI_ISL_2325596, EPI_ISL_2325597, EPI_ISL_2325598, EPI_ISL_2325599, EPI_ISL_2325600, EPI_ISL_2325601, EPI_ISL_2325602, EPI_ISL_2325603, EPI_ISL_2325604, EPI_ISL_2325605, EPI_ISL_2325606, EPI_ISL_2325607, EPI_ISL_2325608, EPI_ISL_2325609, EPI_ISL_2325610, EPI_ISL_2325611, EPI_ISL_2325612, EPI_ISL_2325613, EPI_ISL_2325614, EPI_ISL_2325615, EPI_ISL_2325616, EPI_ISL_2325617			
see above	SARS-CoV-2 testing team, National Institute of Infectious Diseases	Pathogen Genomics Center, National Institute of Infectious Diseases	Tsuyoshi Sekizuka, Kentaro Itokawa, Rina Tanaka, Masanori Hashino, Nozomu Hanaoka, Masumichi Saito, Naomi Nojiri, Hazuka Y Furihata, Sana Uchikoba, Tsuguto Fujimoto, Makoto Kuroda
EPI_ISL_2325618, EPI_ISL_2325619, EPI_ISL_2325620, EPI_ISL_2325621, EPI_ISL_2325622, EPI_ISL_2325623, EPI_ISL_2325624, EPI_ISL_2325625, EPI_ISL_2325626, EPI_ISL_2325627	Pathogen Genomics Center, National Institute of Infectious Diseases	Pathogen Genomics Center, National Institute of Infectious Diseases	Tsuyoshi Sekizuka, Kentaro Itokawa, Rina Tanaka, Masanori Hashino, Makoto Kuroda
EPI_ISL_2325628, EPI_ISL_2325629, EPI_ISL_2325630, EPI_ISL_2325631, EPI_ISL_2325632, EPI_ISL_2325633, EPI_ISL_2325634, EPI_ISL_2325635, EPI_ISL_2325636, EPI_ISL_2325637, EPI_ISL_2325638, EPI_ISL_2325639, EPI_ISL_2325640, EPI_ISL_2325641, EPI_ISL_2325642, EPI_ISL_2325643, EPI_ISL_2325644, EPI_ISL_2325645, EPI_ISL_2325646, EPI_ISL_2325647, EPI_ISL_2325648, EPI_ISL_2325649, EPI_ISL_2325650, EPI_ISL_2325651, EPI_ISL_2325652, EPI_ISL_2325653			
see above	Chiba Prefectural Institute of Public Health	Pathogen Genomics Center, National Institute of Infectious Diseases	Tsuyoshi Sekizuka, Kentaro Itokawa, Rina Tanaka, Masanori Hashino, Makoto Kuroda
EPI_ISL_2325654, EPI_ISL_2325655, EPI_ISL_2325656, EPI_ISL_2325657, EPI_ISL_2325658, EPI_ISL_2325659, EPI_ISL_2325660, EPI_ISL_2325661, EPI_ISL_2325662, EPI_ISL_2325663, EPI_ISL_2325664, EPI_ISL_2325665, EPI_ISL_2325666, EPI_ISL_2325667, EPI_ISL_2325668, EPI_ISL_2325669, EPI_ISL_2325670, EPI_ISL_2325671, EPI_ISL_2325672, EPI_ISL_2325673, EPI_ISL_2325674, EPI_ISL_2325675, EPI_ISL_2325676, EPI_ISL_2325677, EPI_ISL_2325678, EPI_ISL_2325679, EPI_ISL_2325680, EPI_ISL_2325681, EPI_ISL_2325682, EPI_ISL_2325683, EPI_ISL_2325684, EPI_ISL_2325685, EPI_ISL_2325686, EPI_ISL_2325687, EPI_ISL_2325688, EPI_ISL_2325689, EPI_ISL_2325690, EPI_ISL_2325691, EPI_ISL_2325692, EPI_ISL_2325693, EPI_ISL_2325694, EPI_ISL_2325695, EPI_ISL_2325696, EPI_ISL_2325697, EPI_ISL_2325698, EPI_ISL_2325699, EPI_ISL_2325700, EPI_ISL_2325701, EPI_ISL_2325702, EPI_ISL_2325703, EPI_ISL_2325704, EPI_ISL_2325705, EPI_ISL_2325706, EPI_ISL_2325707, EPI_ISL_2325708, EPI_ISL_2325709, EPI_ISL_2325710, EPI_ISL_2325711, EPI_ISL_2325712, EPI_ISL_2325713, EPI_ISL_2325714, EPI_ISL_2325715, EPI_ISL_2325716, EPI_ISL_2325717, EPI_ISL_2325718, EPI_ISL_2325719, EPI_ISL_2325720, EPI_ISL_2325721, EPI_ISL_2325722, EPI_ISL_2325723, EPI_ISL_2325724, EPI_ISL_2325725, EPI_ISL_2325726, EPI_ISL_2325727, EPI_ISL_2325728, EPI_ISL_2325729, EPI_ISL_2325730, EPI_ISL_2325731, EPI_ISL_2325732, EPI_ISL_2325733, EPI_ISL_2325734, EPI_ISL_2325735, EPI_ISL_2325736, EPI_ISL_2325737, EPI_ISL_2325738, EPI_ISL_2325739, EPI_ISL_2325740, EPI_ISL_2325741, EPI_ISL_2325742, EPI_ISL_2325743, EPI_ISL_2325744, EPI_ISL_2325745, EPI_ISL_2325746, EPI_ISL_2325747, EPI_ISL_2325748, EPI_ISL_2325749, EPI_ISL_2325750, EPI_ISL_2325751, EPI_ISL_2325752, EPI_ISL_2325753, EPI_ISL_2325754, EPI_ISL_2325755, EPI_ISL_2325756, EPI_ISL_2325758, EPI_ISL_2325759, EPI_ISL_2325760, EPI_ISL_2325761, EPI_ISL_2325762, EPI_ISL_2325763, EPI_ISL_2325764, EPI_ISL_2325765, EPI_ISL_2325766, EPI_ISL_2325767, EPI_ISL_2325768, EPI_ISL_2325769, EPI_ISL_2325770, EPI_ISL_2325771, EPI_ISL_2325772, EPI_ISL_2325773, EPI_ISL_2325774, EPI_ISL_2325775, EPI_ISL_2325776, EPI_ISL_2325777, EPI_ISL_2325778, EPI_ISL_2325779, EPI_ISL_2325780, EPI_ISL_2325781, EPI_ISL_2325782, EPI_ISL_2325783, EPI_ISL_2325784, EPI_ISL_2325785, EPI_ISL_2325786, EPI_ISL_2325787, EPI_ISL_2325788, EPI_ISL_2325789, EPI_ISL_2325790, EPI_ISL_2325791, EPI_ISL_2325792, EPI_ISL_2325793, EPI_ISL_2325794, EPI_ISL_2325795, EPI_ISL_2325796, EPI_ISL_2325797, EPI_ISL_2325798, EPI_ISL_2325799, EPI_ISL_2325800, EPI_ISL_2325801, EPI_ISL_2325802, EPI_ISL_2325803, EPI_ISL_2325804, EPI_ISL_2325805, EPI_ISL_2325806, EPI_ISL_2325807, EPI_ISL_2325808, EPI_ISL_2325809, EPI_ISL_2325810, EPI_ISL_2325811, EPI_ISL_2325812, EPI_ISL_2325813, EPI_ISL_2325814, EPI_ISL_2325815, EPI_ISL_2325816, EPI_ISL_2325817, EPI_ISL_2325818, EPI_ISL_2325819, EPI_ISL_2325820, EPI_ISL_2325821, EPI_ISL_2325822, EPI_ISL_2325823, EPI_ISL_2325824, EPI_ISL_2325825, EPI_ISL_2325826, EPI_ISL_2325827, EPI_ISL_2325828, EPI_ISL_2325829, EPI_ISL_2325830, EPI_ISL_2325831, EPI_ISL_2325832, EPI_ISL_2325833, EPI_ISL_2325834, EPI_ISL_2325835, EPI_ISL_2325836, EPI_ISL_2325837, EPI_ISL_2325838, EPI_ISL_2325839, EPI_ISL_2325840, EPI_ISL_2325841, EPI_ISL_2325842, EPI_ISL_2325843, EPI_ISL_2325844, EPI_ISL_2325845, EPI_ISL_2325846, EPI_ISL_2325847, EPI_ISL_2325848, EPI_ISL_2325849, EPI_ISL_2325850, EPI_ISL_2325851, EPI_ISL_2325852, EPI_ISL_2325853, EPI_ISL_2325854, EPI_ISL_2325855, EPI_ISL_2325856, EPI_ISL_2325857, EPI_ISL_2325858, EPI_ISL_2325859, EPI_ISL_2325860, EPI_ISL_2325861, EPI_ISL_2325862, EPI_ISL_2325863, EPI_ISL_2325864, EPI_ISL_2325865, EPI_ISL_2325866, EPI_ISL_2325867, EPI_ISL_2325868, EPI_ISL_2325869, EPI_ISL_2325870, EPI_ISL_2325871, EPI_ISL_2325872, EPI_ISL_2325873, EPI_ISL_2325874, EPI_ISL_2325875, EPI_ISL_2325876, EPI_ISL_2325877, EPI_ISL_2325878, EPI_ISL_2325879, EPI_ISL_2325880, EPI_ISL_2325881, EPI_ISL_2325882, EPI_ISL_2325883, EPI_ISL_2325884, EPI_ISL_2325885, EPI_ISL_2325886, EPI_ISL_2325887, EPI_ISL_2325888, EPI_ISL_2325889, EPI_ISL_2325890, EPI_ISL_2325891, EPI_ISL_2325892, EPI_ISL_2325893, EPI_ISL_2325894, EPI_ISL_2325895, EPI_ISL_2325896, EPI_ISL_2325897, EPI_ISL_2325898, EPI_ISL_2325899, EPI_ISL_2325900, EPI_ISL_2325901, EPI_ISL_2325902, EPI_ISL_2325903, EPI_ISL_2325904, EPI_ISL_2325905, EPI_ISL_2325906, EPI_ISL_2325907, EPI_ISL_2325908, EPI_ISL_2325909, EPI_ISL_2325910, EPI_ISL_2325911, EPI_ISL_2325912, EPI_ISL_2325913, EPI_ISL_2325914, EPI_ISL_2325915, EPI_ISL_2325916, EPI_ISL_2325917, EPI_ISL_2325918, EPI_ISL_2325919, EPI_ISL_2325920, EPI_ISL_2325921, EPI_ISL_2325922, EPI_ISL_2325923, EPI_ISL_2325924, EPI_ISL_2325925, EPI_ISL_2325926, EPI_ISL_2325927, EPI_ISL_2325928, EPI_ISL_2325929, EPI_ISL_2325930, EPI_ISL_2325931, EPI_ISL_2325932, EPI_ISL_2325933, EPI_ISL_2325934, EPI_ISL_2325935, EPI_ISL_2325936, EPI_ISL_2325937, EPI_ISL_2325938, EPI_ISL_2325939, EPI_ISL_2325940, EPI_ISL_2325941, EPI_ISL_2325942, EPI_ISL_2325943, EPI_ISL_2325944, EPI_ISL_2325945, EPI_ISL_2325946, EPI_ISL_2325947, EPI_ISL_2325949, EPI_ISL_2325950, EPI_ISL_2325951, EPI_ISL_2325952, EPI_ISL_2325953, EPI_ISL_2325954, EPI_ISL_2325955, EPI_ISL_2325956, EPI_ISL_2325957, EPI_ISL_2325958, EPI_ISL_2325959, EPI_ISL_2325960, EPI_ISL_2325961, EPI_ISL_2325962, EPI_ISL_2325963, EPI_ISL_2325964, EPI_ISL_2325965, EPI_ISL_2325966, EPI_ISL_2325967, EPI_ISL_2325968, EPI_ISL_2325969, EPI_ISL_2325970, EPI_ISL_2325971, EPI_ISL_2325972, EPI_ISL_2325973, EPI_ISL_2325974, EPI_ISL_2325975, EPI_ISL_2325976, EPI_ISL_2325977, EPI_ISL_2325978, EPI_ISL_2325979, EPI_ISL_2325980, EPI_ISL_2325981, EPI_ISL_2325982, EPI_ISL_2325983, EPI_ISL_2325984, EPI_ISL_2325985, EPI_ISL_2325986, EPI_ISL_2325987, EPI_ISL_2325988, EPI_ISL_2325989, EPI_ISL_2325990, EPI_ISL_2325991, EPI_ISL_2325992, EPI_ISL_2325993, EPI_ISL_2325994, EPI_ISL_2325995, EPI_ISL_2325996, EPI_ISL_2325997			
see above	Pathogen Genomics Center, National Institute of Infectious Diseases	Pathogen Genomics Center, National Institute of Infectious Diseases	Tsuyoshi Sekizuka, Kentaro Itokawa, Rina Tanaka, Masanori Hashino, Makoto Kuroda
EPI_ISL_2325998, EPI_ISL_2325999, EPI_ISL_2326000, EPI_ISL_2326001, EPI_ISL_2326002, EPI_ISL_2326003, EPI_ISL_2326004, EPI_ISL_2326005, EPI_ISL_2326006, EPI_ISL_2326007, EPI_ISL_2326008, EPI_ISL_2326009, EPI_ISL_2326010, EPI_ISL_2326011, EPI_ISL_2326012, EPI_ISL_2326013, EPI_ISL_2326014, EPI_ISL_2326015, EPI_ISL_2326016, EPI_ISL_2326017, EPI_ISL_2326018, EPI_ISL_2326019, EPI_ISL_2326020, EPI_ISL_2326021, EPI_ISL_2326022, EPI_ISL_2326023, EPI_ISL_2326024, EPI_ISL_2326025, EPI_ISL_2326026, EPI_ISL_2326027, EPI_ISL_2326028, EPI_ISL_2326029, EPI_ISL_2326030, EPI_ISL_2326031, EPI_ISL_2326032, EPI_ISL_2326033, EPI_ISL_2326034, EPI_ISL_2326035, EPI_ISL_2326036, EPI_ISL_2326037, EPI_ISL_2326038, EPI_ISL_2326039, EPI_ISL_2326040, EPI_ISL_2326041, EPI_ISL_2326042, EPI_ISL_2326043, EPI_ISL_2326044, EPI_ISL_2326045, EPI_ISL_2326046, EPI_ISL_2326047, EPI_ISL_2326048, EPI_ISL_2326049, EPI_ISL_2326050, EPI_ISL_2326051, EPI_ISL_2326052, EPI_ISL_2326053, EPI_ISL_2326054, EPI_ISL_2326055, EPI_ISL_2326056, EPI_ISL_2326057, EPI_ISL_2326058, EPI_ISL_2326059, EPI_ISL_2326060, EPI_ISL_2326061, EPI_ISL_2326062, EPI_ISL_2326063, EPI_ISL_2326064, EPI_ISL_2326065, EPI_ISL_2326066, EPI_ISL_2326067, EPI_ISL_2326068, EPI_ISL_2326069, EPI_ISL_2326070, EPI_ISL_2326071, EPI_ISL_2326072, EPI_ISL_2326073, EPI_ISL_2326074, EPI_ISL_2326075, EPI_ISL_2326076, EPI_ISL_2326077, EPI_ISL_2326078, EPI_ISL_2326079, EPI_ISL_2326080, EPI_ISL_2326081, EPI_ISL_2326082, EPI_ISL_2326083, EPI_ISL_2326084, EPI_ISL_2326085, EPI_ISL_2326086, EPI_ISL_2326087, EPI_ISL_2326088, EPI_ISL_2326089, EPI_ISL_2326090, EPI_ISL_2326091, EPI_ISL_2326092, EPI_ISL_2326093, EPI_ISL_2326094, EPI_ISL_2326095, EPI_ISL_2326096, EPI_ISL_2326097, EPI_ISL_2326098, EPI_ISL_2326099, EPI_ISL_2326100, EPI_ISL_2326101, EPI_ISL_2326102, EPI_ISL_2326103, EPI_ISL_2326104, EPI_ISL_2326105, EPI_ISL_2326106, EPI_ISL_2326107, EPI_ISL_2326108, EPI_ISL_2326109, EPI_ISL_2326110, EPI_ISL_2326111, EPI_ISL_2326112, EPI_ISL_2326113, EPI_ISL_2326114, EPI_ISL_2326115, EPI_ISL_2326116, EPI_ISL_2326117, EPI_ISL_2326118, EPI_ISL_2326119, EPI_ISL_2326120, EPI_ISL_2326121, EPI_ISL_2326122, EPI_ISL_2326123, EPI_ISL_2326124, EPI_ISL_2326125, EPI_ISL_2326126, EPI_ISL_2326127, EPI_ISL_2326128, EPI_ISL_2326129, EPI_ISL_2326130, EPI_ISL_2326131, EPI_ISL_2326132, EPI_ISL_2326133, EPI_ISL_2326134, EPI_ISL_2326135, EPI_ISL_2326136, EPI_ISL_2326137, EPI_ISL_2326138, EPI_ISL_2326139, EPI_ISL_2326140, EPI_ISL_2326141, EPI_ISL_2326142, EPI_ISL_2326143, EPI_ISL_2326144, EPI_ISL_2326145, EPI_ISL_2326146, EPI_ISL_2326147, EPI_ISL_2326148, EPI_ISL_2326149, EPI_ISL_2326150, EPI_ISL_2326151, EPI_ISL_2326152, EPI_ISL_2326153, EPI_ISL_2326154, EPI_ISL_2326155, EPI_ISL_2326156, EPI_ISL_2326157, EPI_ISL_2326158, EPI_ISL_2326159, EPI_ISL_2326160, EPI_ISL_2326161, EPI_ISL_2326162, EPI_ISL_2326163, EPI_ISL_2326164, EPI_ISL_2326165, EPI_ISL_2326166, EPI_ISL_2326167, EPI_ISL_2326168, EPI_ISL_2326169, EPI_ISL_2326170, EPI_ISL_2326171, EPI_ISL_2326172, EPI_ISL_2326173, EPI_ISL_2326174, EPI_ISL_2326175, EPI_ISL_2326176, EPI_ISL_2326177, EPI_ISL_2326178, EPI_ISL_2326179, EPI_ISL_2326180, EPI_ISL_2326181, EPI_ISL_2326182, EPI_ISL_2326183, EPI_ISL_2326184			

We gratefully acknowledge the following Authors from the Originating laboratories responsible for obtaining the specimens, as well as the Submitting laboratories where the genome data were generated and shared via GISAID, on which this research is based.

All Submitters of data may be contacted directly via www.gisaid.org

Authors are sorted alphabetically.

Accession ID	Originating Laboratory	Submitting Laboratory	Authors
EPI_ISL_2349203, EPI_ISL_2349205, EPI_ISL_2349206, EPI_ISL_2349207, EPI_ISL_2349208, EPI_ISL_2349209, EPI_ISL_2349210, EPI_ISL_2349211, EPI_ISL_2349212, EPI_ISL_2349213, EPI_ISL_2349214, EPI_ISL_2349215, EPI_ISL_2349216, EPI_ISL_2349217, EPI_ISL_2349218, EPI_ISL_2349219, EPI_ISL_2349220, EPI_ISL_2349221, EPI_ISL_2349222, EPI_ISL_2349223			
see above	Gunma Prefectural Institute of Public Health and Environmental Sciences	Gunma Prefectural Institute of Public Health and Environmental Sciences	Tsuyoshi Sekizuka, Kentaro Itokawa, Rina Tanaka, Masanori Hashino, Makoto Kuroda
EPI_ISL_2349224, EPI_ISL_2349225, EPI_ISL_2349226, EPI_ISL_2349227, EPI_ISL_2349228, EPI_ISL_2349229, EPI_ISL_2349230, EPI_ISL_2349231, EPI_ISL_2349232, EPI_ISL_2349233, EPI_ISL_2349234, EPI_ISL_2349235, EPI_ISL_2349236, EPI_ISL_2349237, EPI_ISL_2349238, EPI_ISL_2349239, EPI_ISL_2349240, EPI_ISL_2349241, EPI_ISL_2349242, EPI_ISL_2349243, EPI_ISL_2349244, EPI_ISL_2349245, EPI_ISL_2349246, EPI_ISL_2349247, EPI_ISL_2349248, EPI_ISL_2349250, EPI_ISL_2349251, EPI_ISL_2349252, EPI_ISL_2349253, EPI_ISL_2349254, EPI_ISL_2349255, EPI_ISL_2349256, EPI_ISL_2349257, EPI_ISL_2349258, EPI_ISL_2349259, EPI_ISL_2349260, EPI_ISL_2349261, EPI_ISL_2349262, EPI_ISL_2349263, EPI_ISL_2349264, EPI_ISL_2349265, EPI_ISL_2349266, EPI_ISL_2349267, EPI_ISL_2349268			
see above	Gunma Prefectural Institute of Public Health and Environmental Sciences	Gunma Prefectural Institute of Public Health and Environmental Sciences	Tsuyoshi Sekizuka, Kentaro Itokawa, Rina Tanaka, Masanori Hashino, Makoto Kuroda, Hiroyuki Tsukagoshi, Daisuke Shinoda, Mariko Saito
EPI_ISL_2349693, EPI_ISL_2349694, EPI_ISL_2349696, EPI_ISL_2349697, EPI_ISL_2349698, EPI_ISL_2349699, EPI_ISL_2349700, EPI_ISL_2349701, EPI_ISL_2349702, EPI_ISL_2349704, EPI_ISL_2349705, EPI_ISL_2349706, EPI_ISL_2349707, EPI_ISL_2349708			
see above	Genome Analysis Center, Yamanashi Central Hospital	Genome Analysis Center, Yamanashi Central Hospital	Yosuke Hirotsu
EPI_ISL_2350403, EPI_ISL_2350404, EPI_ISL_2350406, EPI_ISL_2350407, EPI_ISL_2350408, EPI_ISL_2350409, EPI_ISL_2350410, EPI_ISL_2350411, EPI_ISL_2350412, EPI_ISL_2350413, EPI_ISL_2350414, EPI_ISL_2350415, EPI_ISL_2350416, EPI_ISL_2350417, EPI_ISL_2350418, EPI_ISL_2350419, EPI_ISL_2350420, EPI_ISL_2350421, EPI_ISL_2350422, EPI_ISL_2350424, EPI_ISL_2350425, EPI_ISL_2350426, EPI_ISL_2350427, EPI_ISL_2350428, EPI_ISL_2350429, EPI_ISL_2350430, EPI_ISL_2350431, EPI_ISL_2350432, EPI_ISL_2350433, EPI_ISL_2350434, EPI_ISL_2350435, EPI_ISL_2350436, EPI_ISL_2350437, EPI_ISL_2350438, EPI_ISL_2350439, EPI_ISL_2350440, EPI_ISL_2350441, EPI_ISL_2350442, EPI_ISL_2350443, EPI_ISL_2350444, EPI_ISL_2350445, EPI_ISL_2350446, EPI_ISL_2350448, EPI_ISL_2350449, EPI_ISL_2350450, EPI_ISL_2350451, EPI_ISL_2350452, EPI_ISL_2350454, EPI_ISL_2350455, EPI_ISL_2350456, EPI_ISL_2350457, EPI_ISL_2350458, EPI_ISL_2350459, EPI_ISL_2350460, EPI_ISL_2350461, EPI_ISL_2350462, EPI_ISL_2350463, EPI_ISL_2350464, EPI_ISL_2350465, EPI_ISL_2350466, EPI_ISL_2350467, EPI_ISL_2350468, EPI_ISL_2350469, EPI_ISL_2350470, EPI_ISL_2350471, EPI_ISL_2350472, EPI_ISL_2350473, EPI_ISL_2350474, EPI_ISL_2350475, EPI_ISL_2350476, EPI_ISL_2350477, EPI_ISL_2350478, EPI_ISL_2350479, EPI_ISL_2350480, EPI_ISL_2350481, EPI_ISL_2350482, EPI_ISL_2350483, EPI_ISL_2350484			
see above	Saitama Prefectural Institute of Public Health	Saitama Prefectural Institute of Public Health	Tsuyoshi Sekizuka, Kentaro Itokawa, Rina Tanaka, Masanori Hashino, Makoto Kuroda,Satoshi Daisaki,Eri Aonuma,Hayato Ehara
EPI_ISL_2350486, EPI_ISL_2350487, EPI_ISL_2350488, EPI_ISL_2350489, EPI_ISL_2350490, EPI_ISL_2350491, EPI_ISL_2350492, EPI_ISL_2350493, EPI_ISL_2350494, EPI_ISL_2350495, EPI_ISL_2350496, EPI_ISL_2350497, EPI_ISL_2350498, EPI_ISL_2350499, EPI_ISL_2350500, EPI_ISL_2350501, EPI_ISL_2350502, EPI_ISL_2350503, EPI_ISL_2350504, EPI_ISL_2350505, EPI_ISL_2350506, EPI_ISL_2350507, EPI_ISL_2350508, EPI_ISL_2350509, EPI_ISL_2350510, EPI_ISL_2350511, EPI_ISL_2350512, EPI_ISL_2350513, EPI_ISL_2350514, EPI_ISL_2350515, EPI_ISL_2350516, EPI_ISL_2350517, EPI_ISL_2350518, EPI_ISL_2350519, EPI_ISL_2350520, EPI_ISL_2350521, EPI_ISL_2350522, EPI_ISL_2350523, EPI_ISL_2350524, EPI_ISL_2350525, EPI_ISL_2350526, EPI_ISL_2350527, EPI_ISL_2350528, EPI_ISL_2350529, EPI_ISL_2350530, EPI_ISL_2350531, EPI_ISL_2350532, EPI_ISL_2350533, EPI_ISL_2350534, EPI_ISL_2350535, EPI_ISL_2350536, EPI_ISL_2350537, EPI_ISL_2350538, EPI_ISL_2350539, EPI_ISL_2350540, EPI_ISL_2350541, EPI_ISL_2350542, EPI_ISL_2350543, EPI_ISL_2350544, EPI_ISL_2350545, EPI_ISL_2350546, EPI_ISL_2350547, EPI_ISL_2350548, EPI_ISL_2350549, EPI_ISL_2350550, EPI_ISL_2350551, EPI_ISL_2350552, EPI_ISL_2350553, EPI_ISL_2350554, EPI_ISL_2350555, EPI_ISL_2350556, EPI_ISL_2350557, EPI_ISL_2350558, EPI_ISL_2350559, EPI_ISL_2350560, EPI_ISL_2350561, EPI_ISL_2350562, EPI_ISL_2350563, EPI_ISL_2350564, EPI_ISL_2350565, EPI_ISL_2350566, EPI_ISL_2350567, EPI_ISL_2350568, EPI_ISL_2350569, EPI_ISL_2350570, EPI_ISL_2350571, EPI_ISL_2350572, EPI_ISL_2350573, EPI_ISL_2350574, EPI_ISL_2350575, EPI_ISL_2350576, EPI_ISL_2350577, EPI_ISL_2350578, EPI_ISL_2350579, EPI_ISL_2350580, EPI_ISL_2350581, EPI_ISL_2350582, EPI_ISL_2350583, EPI_ISL_2350584, EPI_ISL_2350585, EPI_ISL_2350586, EPI_ISL_2350587, EPI_ISL_2350588, EPI_ISL_2350589, EPI_ISL_2350590, EPI_ISL_2350591, EPI_ISL_2350592, EPI_ISL_2350593, EPI_ISL_2350594, EPI_ISL_2350595, EPI_ISL_2350596, EPI_ISL_2350597, EPI_ISL_2350598, EPI_ISL_2350599, EPI_ISL_2350600, EPI_ISL_2350601, EPI_ISL_2350602, EPI_ISL_2350603, EPI_ISL_2350604, EPI_ISL_2350605, EPI_ISL_2350606, EPI_ISL_2350607, EPI_ISL_2350608, EPI_ISL_2350609, EPI_ISL_2350610, EPI_ISL_2350611, EPI_ISL_2350612, EPI_ISL_2350613, EPI_ISL_2350614, EPI_ISL_2350615, EPI_ISL_2350616, EPI_ISL_2350617, EPI_ISL_2350618, EPI_ISL_2350619, EPI_ISL_2350620, EPI_ISL_2350621, EPI_ISL_2350622, EPI_ISL_2350623, EPI_ISL_2350624, EPI_ISL_2350625, EPI_ISL_2350626, EPI_ISL_2350627, EPI_ISL_2350628, EPI_ISL_2350629, EPI_ISL_2350630, EPI_ISL_2350631, EPI_ISL_2350632, EPI_ISL_2350633, EPI_ISL_2350634, EPI_ISL_2350635, EPI_ISL_2350636, EPI_ISL_2350637, EPI_ISL_2350638, EPI_ISL_2350639, EPI_ISL_2350640, EPI_ISL_2350641, EPI_ISL_2350642, EPI_ISL_2350643, EPI_ISL_2350644, EPI_ISL_2350645, EPI_ISL_2350646, EPI_ISL_2350647, EPI_ISL_2350648, EPI_ISL_2350649, EPI_ISL_2350650, EPI_ISL_2350651, EPI_ISL_2350652, EPI_ISL_2350653, EPI_ISL_2350654, EPI_ISL_2350655, EPI_ISL_2350656, EPI_ISL_2350657, EPI_ISL_2350658, EPI_ISL_2350659, EPI_ISL_2350660, EPI_ISL_2350661, EPI_ISL_2350662, EPI_ISL_2350663, EPI_ISL_2350664, EPI_ISL_2350665, EPI_ISL_2350666, EPI_ISL_2350667, EPI_ISL_2350668, EPI_ISL_2350669, EPI_ISL_2350670, EPI_ISL_2350671, EPI_ISL_2350672, EPI_ISL_2350673, EPI_ISL_2350674, EPI_ISL_2350675, EPI_ISL_2350676, EPI_ISL_2350677, EPI_ISL_2350678, EPI_ISL_2350679, EPI_ISL_2350680, EPI_ISL_2350681, EPI_ISL_2350682, EPI_ISL_2350683, EPI_ISL_2350684, EPI_ISL_2350685, EPI_ISL_2350686, EPI_ISL_2350687, EPI_ISL_2350688, EPI_ISL_2350689, EPI_ISL_2350690, EPI_ISL_2350691, EPI_ISL_2350692, EPI_ISL_2350693, EPI_ISL_2350694, EPI_ISL_2350695, EPI_ISL_2350696, EPI_ISL_2350697, EPI_ISL_2350698, EPI_ISL_2350699, EPI_ISL_2350700, EPI_ISL_2350701, EPI_ISL_2350702, EPI_ISL_2350703, EPI_ISL_2350704, EPI_ISL_2350705, EPI_ISL_2350706, EPI_ISL_2350707, EPI_ISL_2350708, EPI_ISL_2350709, EPI_ISL_2350710, EPI_ISL_2350711, EPI_ISL_2350712, EPI_ISL_2350713, EPI_ISL_2350714, EPI_ISL_2350715, EPI_ISL_2350716, EPI_ISL_2350717, EPI_ISL_2350718, EPI_ISL_2350719, EPI_ISL_2350720, EPI_ISL_2350721, EPI_ISL_2350722, EPI_ISL_2350723, EPI_ISL_2350724, EPI_ISL_2350725, EPI_ISL_2350726, EPI_ISL_2350727, EPI_ISL_2350728, EPI_ISL_2350729, EPI_ISL_2350730, EPI_ISL_2350731, EPI_ISL_2350732, EPI_ISL_2350733, EPI_ISL_2350735, EPI_ISL_2350736, EPI_ISL_2350737, EPI_ISL_2350738, EPI_ISL_2350739, EPI_ISL_2350740, EPI_ISL_2350741, EPI_ISL_2350742, EPI_ISL_2350743, EPI_ISL_2350744, EPI_ISL_2350746, EPI_ISL_2350747, EPI_ISL_2350748, EPI_ISL_2350749, EPI_ISL_2350750, EPI_ISL_2350751, EPI_ISL_2350752, EPI_ISL_2350753, EPI_ISL_2350754, EPI_ISL_2350755, EPI_ISL_2350756, EPI_ISL_2350757, EPI_ISL_2350758, EPI_ISL_2350759, EPI_ISL_2350760, EPI_ISL_2350761, EPI_ISL_2350762, EPI_ISL_2350763, EPI_ISL_2350764, EPI_ISL_2350765, EPI_ISL_2350766, EPI_ISL_2350767, EPI_ISL_2350768, EPI_ISL_2350769, EPI_ISL_2350770, EPI_ISL_2350771, EPI_ISL_2350772, EPI_ISL_2350773, EPI_ISL_2350774, EPI_ISL_2350775, EPI_ISL_2350776, EPI_ISL_2350777, EPI_ISL_2350778, EPI_ISL_2350779, EPI_ISL_2350780, EPI_ISL_2350781, EPI_ISL_2350782, EPI_ISL_2350783			
see above	Ibaraki Prefectural Institute of Public Health	Ibaraki Prefectural Institute of Public Health	Keiko Goto, Tsuyoshi Sekizuka, Kentaro Itokawa, Rina Tanaka, Masanori Hashino, Makoto Kuroda
EPI_ISL_2357237, EPI_ISL_2357238, EPI_ISL_2357239, EPI_ISL_2357240, EPI_ISL_2357241, EPI_ISL_2357242, EPI_ISL_2357243, EPI_ISL_2357244, EPI_ISL_2357245, EPI_ISL_2357246, EPI_ISL_2357247, EPI_ISL_2357248, EPI_ISL_2357249, EPI_ISL_2357250, EPI_ISL_2357251, EPI_ISL_2357252, EPI_ISL_2357253, EPI_ISL_2357254, EPI_ISL_2357255, EPI_ISL_2357256, EPI_ISL_2357257, EPI_ISL_2357258, EPI_ISL_2357259, EPI_ISL_2357260, EPI_ISL_2357261, EPI_ISL_2357262, EPI_ISL_2357263, EPI_ISL_2357264, EPI_ISL_2357265, EPI_ISL_2357266, EPI_ISL_2357267, EPI_ISL_2357268, EPI_ISL_2357269, EPI_ISL_2357270, EPI_ISL_2357271, EPI_ISL_2357272, EPI_ISL_2357273, EPI_ISL_2357274, EPI_ISL_2357275, EPI_ISL_2357276, EPI_ISL_2357277, EPI_ISL_2357278, EPI_ISL_2357279, EPI_ISL_2357280, EPI_ISL_2357281, EPI_ISL_2357282, EPI_ISL_2357283, EPI_ISL_2357284, EPI_ISL_2357285, EPI_ISL_2357286, EPI_ISL_2357287, EPI_ISL_2357288, EPI_ISL_2357289, EPI_ISL_2357290, EPI_ISL_2357291, EPI_ISL_2357292, EPI_ISL_2357293, EPI_ISL_2357294, EPI_ISL_2357295, EPI_ISL_2357296, EPI_ISL_2357297, EPI_ISL_2357298, EPI_ISL_2357299, EPI_ISL_2357300, EPI_ISL_2357301, EPI_ISL_2357302, EPI_ISL_2357303, EPI_ISL_2357304, EPI_ISL_2357305, EPI_ISL_2357306, EPI_ISL_2357307, EPI_ISL_2357308, EPI_ISL_2357309, EPI_ISL_2357310, EPI_ISL_2357311, EPI_ISL_2357312, EPI_ISL_2357313, EPI_ISL_2357314, EPI_ISL_2357315, EPI_ISL_2357316, EPI_ISL_2357317, EPI_ISL_2357318, EPI_ISL_2357319, EPI_ISL_2357320, EPI_ISL_2357321, EPI_ISL_2357322, EPI_ISL_2357323, EPI_ISL_2357324, EPI_ISL_2357325, EPI_ISL_2357326, EPI_ISL_2357327, EPI_ISL_2357328, EPI_ISL_2357329, EPI_ISL_2357330, EPI_ISL_2357331, EPI_ISL_2357332, EPI_ISL_2357333, EPI_ISL_2357334, EPI_ISL_2357335, EPI_ISL_2357336, EPI_ISL_2357337, EPI_ISL_2357338, EPI_ISL_2357339, EPI_ISL_2357340, EPI_ISL_2357341, EPI_ISL_2357343, EPI_ISL_2357344, EPI_ISL_2357345, EPI_ISL_2357346, EPI_ISL_2357347, EPI_ISL_2357348, EPI_ISL_2357349, EPI_ISL_2357350, EPI_ISL_2357351, EPI_ISL_2357352, EPI_ISL_2357353, EPI_ISL_2357354, EPI_ISL_2357355, EPI_ISL_2357356, EPI_ISL_2357357, EPI_ISL_2357358, EPI_ISL_2357359, EPI_ISL_2357360, EPI_ISL_2357361, EPI_ISL_2357362, EPI_ISL_2357363, EPI_ISL_2357364, EPI_ISL_2357365, EPI_ISL_2357366, EPI_ISL_2357367, EPI_ISL_2357368, EPI_ISL_2357369, EPI_ISL_2357370, EPI_ISL_2357371, EPI_ISL_2357372, EPI_ISL_2357373, EPI_ISL_2357374, EPI_ISL_2357375, EPI_ISL_2357376, EPI_ISL_2357377, EPI_ISL_2357378, EPI_ISL_2357379, EPI_ISL_2357380, EPI_ISL_2357381, EPI_ISL_2357382, EPI_ISL_2357383, EPI_ISL_2357384, EPI_ISL_2357385, EPI_ISL_2357386, EPI_ISL_2357387, EPI_ISL_2357388, EPI_ISL_2357389, EPI_ISL_2357390, EPI_ISL_2357391, EPI_ISL_2357392, EPI_ISL_2357393, EPI_ISL_2357394, EPI_ISL_2357395, EPI_ISL_2357396, EPI_ISL_2357397, EPI_ISL_2357398, EPI_ISL_2357399, EPI_ISL_2357400, EPI_ISL_2357401, EPI_ISL_2357402, EPI_ISL_2357403, EPI_ISL_2357404, EPI_ISL_2357405, EPI_ISL_2357406, EPI_ISL_2357407, EPI_ISL_2357408, EPI_ISL_2357409, EPI_ISL_2357410, EPI_ISL_2357411, EPI_ISL_2357412, EPI_ISL_2357413, EPI_ISL_2357414, EPI_ISL_2357415, EPI_ISL_2357416, EPI_ISL_2357417, EPI_ISL_2357418, EPI_ISL_2357419, EPI_ISL_2357420, EPI_ISL_2357421, EPI_ISL_2357422, EPI_ISL_2357423, EPI_ISL_2357424, EPI_ISL_2357425, EPI_ISL_2357426, EPI_ISL_2357427, EPI_ISL_2357428, EPI_ISL_2357429, EPI_ISL_2357430, EPI_ISL_2357431, EPI_ISL_2357432, EPI_ISL_2357433, EPI_ISL_2357434, EPI_ISL_2357435, EPI_ISL_2357436, EPI_ISL_2357437, EPI_ISL_2357438, EPI_ISL_2357439, EPI_ISL_2357440, EPI_ISL_2357441, EPI_ISL_2357442, EPI_ISL_2357443, EPI_ISL_2357444, EPI_ISL_2357445, EPI_ISL_2357446, EPI_ISL_2357447, EPI_ISL_2357448, EPI_ISL_2357449, EPI_ISL_2357450, EPI_ISL_2357451, EPI_ISL_2357452, EPI_ISL_2357453, EPI_ISL_2357454, EPI_ISL_2357455, EPI_ISL_2357456, EPI_ISL_2357457, EPI_ISL_2357458, EPI_ISL_2357459, EPI_ISL_2357460, EPI_ISL_2357461, EPI_ISL_2357462, EPI_ISL_2357463, EPI_ISL_2357464, EPI_ISL_2357465, EPI_ISL_2357466, EPI_ISL_2357467, EPI_ISL_2357468, EPI_ISL_2357469, EPI_ISL_2357470, EPI_ISL_2357471, EPI_ISL_2357472, EPI_ISL_2357473, EPI_ISL_2357474, EPI_ISL_2357475, EPI_ISL_2357476, EPI_ISL_2357477, EPI_ISL_2357478, EPI_ISL_2357479, EPI_ISL_2357480, EPI_ISL_2357481, EPI_ISL_2357482, EPI_ISL_2357483, EPI_ISL_2357484, EPI_ISL_2357485, EPI_ISL_2357486, EPI_ISL_2357487, EPI_ISL_2357488, EPI_ISL_2357489, EPI_ISL_2357490, EPI_ISL_2357491, EPI_ISL_2357492, EPI_ISL_2357493, EPI_ISL_2357494, EPI_ISL_2357495, EPI_ISL_2357496, EPI_ISL_2357497, EPI_ISL_2357498, EPI_ISL_2357499, EPI_ISL_2357500, EPI_ISL_2357501, EPI_ISL_2357502, EPI_ISL_2357503, EPI_ISL_2357504, EPI_ISL_2357505, EPI_ISL_2357506, EPI_ISL_2357507, EPI_ISL_2357508, EPI_ISL_2357509, EPI_ISL_2357510, EPI_ISL_2357511, EPI_ISL_2357512, EPI_ISL_2357513, EPI_ISL_2357514, EPI_ISL_2357515, EPI_ISL_2357516, EPI_ISL_2357517, EPI_ISL_2357518, EPI_ISL_2357519, EPI_ISL_2357520, EPI_ISL_2357521, EPI_ISL_2357522, EPI_ISL_2357523, EPI_ISL_2357524, EPI_ISL_2357525, EPI_ISL_2357526, EPI_ISL_2357527, EPI_ISL_2357528, EPI_ISL_2357529, EPI_ISL_2357530, EPI_ISL_2357531, EPI_ISL_2357532, EPI_ISL_2357533, EPI_ISL_2357534, EPI_ISL_2357535, EPI_ISL_2357536, EPI_ISL_2357537, EPI_ISL_2357538, EPI_ISL_2357539, EPI_ISL_2357540, EPI_ISL_2357541, EPI_ISL_2357542, EPI_ISL_2357543, EPI_ISL_2357544, EPI_ISL_2357545, EPI_ISL_2357546, EPI_ISL_2357547, EPI_ISL_2357548, EPI_ISL_2357549, EPI_ISL_2357550, EPI_ISL_2357551, EPI_ISL_2357552, EPI_ISL_2357553, EPI_ISL_2357554, EPI_ISL_2357555, EPI_ISL_2357556, EPI_ISL_2357557, EPI_ISL_2357558, EPI_ISL_2357559, EPI_ISL_2357560, EPI_ISL_2357561, EPI_ISL_2357562, EPI_ISL_2357563, EPI_ISL_2357564, EPI_ISL_2357565, EPI_ISL_2357566, EPI_ISL_2357567, EPI_ISL_2357568, EPI_ISL_2357569, EPI_ISL_2357570, EPI_ISL_2357571, EPI_ISL_2357572, EPI_ISL_2357573, EPI_ISL_2357574, EPI_ISL_2357575, EPI_ISL_2357576, EPI_ISL_2357577, EPI_ISL_2357578, EPI_ISL_2357579, EPI_ISL_2357580, EPI_ISL_2357581, EPI_ISL_2357582, EPI_ISL_2357583, EPI_ISL_2357584, EPI_ISL_2357585, EPI_ISL_2357586, EPI_ISL_2357587, EPI_ISL_2357588, EPI_ISL_2357589, EPI_ISL_2357590, EPI_ISL_2357591, EPI_ISL_2357592, EPI_ISL_2357593, EPI_ISL_2357594, EPI_ISL_2357595, EPI_ISL_2357596, EPI_ISL_2357597, EPI_ISL_2357598, EPI_ISL_2357599, EPI_ISL_2357600, EPI_ISL_2357601, EPI_ISL_2357602, EPI_ISL_2357603, EPI_ISL_2357604, EPI_ISL_2357605, EPI_ISL_2357606, EPI_ISL_2357607, EPI_ISL_2357608, EPI_ISL_2357609, EPI_ISL_2357610, EPI_ISL_2357611, EPI_ISL_2357612, EPI_ISL_2357613, EPI_ISL_2357614, EPI_ISL_2357615, EPI_ISL_2357617, EPI_ISL_2357618, EPI_ISL_2357619, EPI_ISL_2357620, EPI_ISL_2357621, EPI_ISL_2357622, EPI_ISL_2357623, EPI_ISL_2357624, EPI_ISL_2357625, EPI_ISL_2357626, EPI_ISL_2357627, EPI_ISL_2357628, EPI_ISL_2357629, EPI_ISL_2357630, EPI_ISL_2357631, EPI_ISL_2357632, EPI_ISL_2357633, EPI_ISL_2357634, EPI_ISL_2357635, EPI_ISL_2357636, EPI_ISL_2357637, EPI_ISL_2357638, EPI_ISL_2357639, EPI_ISL_2357640, EPI_ISL_2357641, EPI_ISL_2357642, EPI_ISL_2357643, EPI_ISL_2357644, EPI_ISL_2357645, EPI_ISL_2357646, EPI_ISL_2357647, EPI_ISL_2357648, EPI_ISL_2357649, EPI_ISL_2357650, EPI_ISL_2357651, EPI_ISL_2357652, EPI_ISL_2357653, EPI_ISL_2357654, EPI_ISL_2357655, EPI_ISL_2357656, EPI_ISL_2357657, EPI_ISL_2357658, EPI_ISL_2357659, EPI_ISL_2357660, EPI_ISL_2357661, EPI_ISL_2357662, EPI_ISL_2357663, EPI_ISL_2357664, EPI_ISL_2357665, EPI_ISL_2357666, EPI_ISL_2357667, EPI_ISL_2357668, EPI_ISL_2357669, EPI_ISL_2357670, EPI_ISL_2357671, EPI_ISL_2357672, EPI_ISL_2357673, EPI_ISL_2357674, EPI_ISL_2357675, EPI_ISL_2357676, EPI_ISL_2357677, EPI_ISL_2357678, EPI_ISL_2357679, EPI_ISL_2357680, EPI_ISL_2357681, EPI_ISL_2357682, EPI_ISL_2357683, EPI_ISL_2357684, EPI_ISL_2357685, EPI_ISL_2357686, EPI_ISL_2357687, EPI_ISL_2357688, EPI_ISL_2357689, EPI_ISL_2357690, EPI_ISL_2357691, EPI_ISL_2357692, EPI_ISL_2357693, EPI_ISL_2357694, EPI_ISL_2357695, EPI_ISL_2357696, EPI_ISL_2357697, EPI_ISL_2357698, EPI_ISL_2357699, EPI_ISL_2357700, EPI_ISL_2357701, EPI_ISL_2357702, EPI_ISL_2357703, EPI_ISL_2357704, EPI_ISL_2357705, EPI_ISL_2357706, EPI_ISL_2357707, EPI_ISL_2357708, EPI_ISL_2357709, EPI_ISL_2357710, EPI_ISL_2357711, EPI_ISL_2357712, EPI_ISL_2357713, EPI_ISL_2357714			
see above	Fukuoka Institute of Health and Environmental Sciences	Fukuoka Institute of Health and Environmental Sciences	Asako Nakamura, Yoshiki Etoh, Hiroaki Shigemura, Akira Ohishi, Takayuki Kobayashi, Carl Yuki, Chiharu Katamune, Saori Ueda, Mitsuhiro Hamasaki, Yuki Ashizuka, Tsuyoshi Sekizuka, Kentaro Itokawa, Rina Tanaka, Masanori Hashino, Makoto Kuroda
EPI_ISL_2357899, EPI_ISL_2357900, EPI_ISL_2357901, EPI_ISL_2357902, EPI_ISL_2357903, EPI_ISL_2357904, EPI_ISL_2357905, EPI_ISL_2357906, EPI_ISL_2357907, EPI_ISL_2357908, EPI_ISL_2357909, EPI_ISL_2357910, EPI_ISL_2357911, EPI_ISL_2357912, EPI_ISL_2357913, EPI_ISL_2357914, EPI_ISL_2357915, EPI_ISL_2357916, EPI_ISL_2357917, EPI_ISL_2357918, EPI_ISL_2357919, EPI_ISL_2357920, EPI_ISL_2357921, EPI_ISL_2357922, EPI_ISL_2357923, EPI_ISL_2357924, EPI_ISL_2357925, EPI_ISL_2357926, EPI_ISL_2357927, EPI_ISL_2357928, EPI_ISL_2357929, EPI_ISL_2357930, EPI_ISL_2357931, EPI_ISL_2357932, EPI_ISL_2357933, EPI_ISL_2357934, EPI_ISL_2357935, EPI_ISL_2357936, EPI_ISL_2357937, EPI_ISL_2357938, EPI_ISL_2357939, EPI_ISL_2357940, EPI_ISL_2357941, EPI_ISL_2357942, EPI_ISL_2357943, EPI_ISL_2357944, EPI_ISL_2357945, EPI_ISL_2357946, EPI_ISL_2357947, EPI_ISL_2357949, EPI_ISL_2357950,			

Brazil

We gratefully acknowledge the following Authors from the Originating laboratories responsible for obtaining the specimens, as well as the Submitting laboratories where the genome data were generated and shared via GISAID, on which this research is based.

All Submitters of data may be contacted directly via www.gisaid.org

Authors are sorted alphabetically.

Accession ID	Originating Laboratory	Submitting Laboratory	Authors
EPI_ISL_1000670, EPI_ISL_1000671, EPI_ISL_1000673, EPI_ISL_1000675, EPI_ISL_1000677	Instituto de Biotecnologia - UNESP-Botucatu-SP	Instituto de Biotecnologia - UNESP-Botucatu-SP	Leila Sabrina Ullmann; Fábio Sossai Possebon, Camila Dantas Malossi, Paula Rahal, Paulo Inacio da Costa, João Pessoa Araújo Jr.
EPI_ISL_1023841	Centro de Desenvolvimento Tecnológico em Saúde - CDTS	Centro de Desenvolvimento Tecnológico em Saúde - CDTS	Souza,T.M., Fintelman-Rodrigues,N., De Paula,A.D., Saraiva,F.B., Ferreira,M.A. and Sacramento,C.Q.
EPI_ISL_1034304, EPI_ISL_1034305, EPI_ISL_1034306	Laboratorio de Ecologia de Doencas Transmissiveis na Amazonia, Instituto Leonidas e Maria Deane - Fiocruz Amazonia	Laboratorio de Ecologia de Doencas Transmissiveis na Amazonia, Instituto Leonidas e Maria Deane - Fiocruz Amazonia	Valdinete Nascimento, Victor Souza, André Corado, Fernanda Nascimento, George Silva, Ágatha Costa, Debora Duarte, Karina Pessoa, Matilde Mejía, Luciana Gonçalves, Maria Júlia Brandão, Michele Jesus, Felipe Naveca on behalf of the Fiocruz COVID-19 Genomic Surveillance Network
EPI_ISL_1039691, EPI_ISL_1039692, EPI_ISL_1039693, EPI_ISL_1039694, EPI_ISL_1039695	LACEN do Estado de Goias	Instituto Adolfo Lutz, Interdiciplinary Procedures Center, Strategic Laboratory	Claudio Tavares Sacchi, Claudia Regina Gonçalves, Erica Valessa Ramos Gomes, Karoline Rodrigues Campos
EPI_ISL_1039696	Instituto Adolfo Lutz - Regional de Presidente Prudente	Instituto Adolfo Lutz, Interdiciplinary Procedures Center, Strategic Laboratory	Claudio Tavares Sacchi, Claudia Regina Gonçalves, Erica Valessa Ramos Gomes, Karoline Rodrigues Campos
EPI_ISL_1039697	Instituto Adolfo Lutz Central	Instituto Adolfo Lutz, Interdiciplinary Procedures Center, Strategic Laboratory	Claudio Tavares Sacchi, Claudia Regina Gonçalves, Erica Valessa Ramos Gomes, Karoline Rodrigues Campos
EPI_ISL_1039698	Lab Loc - Itapecerica da Serra	Instituto Adolfo Lutz, Interdiciplinary Procedures Center, Strategic Laboratory	Claudio Tavares Sacchi, Claudia Regina Gonçalves, Erica Valessa Ramos Gomes, Karoline Rodrigues Campos
EPI_ISL_1039699	Instituto Adolfo Lutz - Regional de Taubate	Instituto Adolfo Lutz, Interdiciplinary Procedures Center, Strategic Laboratory	Claudio Tavares Sacchi, Claudia Regina Gonçalves, Erica Valessa Ramos Gomes, Karoline Rodrigues Campos
EPI_ISL_1039700, EPI_ISL_1039701	Instituto Adolfo Lutz Central	Instituto Adolfo Lutz, Interdiciplinary Procedures Center, Strategic Laboratory	Claudio Tavares Sacchi, Claudia Regina Gonçalves, Erica Valessa Ramos Gomes, Karoline Rodrigues Campos
EPI_ISL_1039702	Instituto Adolfo Lutz - Regional de Aracatuba	Instituto Adolfo Lutz, Interdiciplinary Procedures Center, Strategic Laboratory	Claudio Tavares Sacchi, Claudia Regina Gonçalves, Erica Valessa Ramos Gomes, Karoline Rodrigues Campos
EPI_ISL_1039703	Instituto Adolfo Lutz - Regional de Taubate	Instituto Adolfo Lutz, Interdiciplinary Procedures Center, Strategic Laboratory	Claudio Tavares Sacchi, Claudia Regina Gonçalves, Erica Valessa Ramos Gomes, Karoline Rodrigues Campos
EPI_ISL_1039704	Lab Loc - Itapecerica da Serra	Instituto Adolfo Lutz, Interdiciplinary Procedures Center, Strategic Laboratory	Claudio Tavares Sacchi, Claudia Regina Gonçalves, Erica Valessa Ramos Gomes, Karoline Rodrigues Campos
EPI_ISL_1039705, EPI_ISL_1039706, EPI_ISL_1039707, EPI_ISL_1039708, EPI_ISL_1039709, EPI_ISL_1039710	Instituto Adolfo Lutz Central	Instituto Adolfo Lutz, Interdiciplinary Procedures Center, Strategic Laboratory	Claudio Tavares Sacchi, Claudia Regina Gonçalves, Erica Valessa Ramos Gomes, Karoline Rodrigues Campos
EPI_ISL_1040823	Secretaria Municipal de Saude de Piracaia	Instituto Adolfo Lutz, Interdiciplinary Procedures Center, Strategic Laboratory	Claudio Tavares Sacchi, Claudia Regina Gonçalves, Erica Valessa Ramos Gomes, Karoline Rodrigues Campos
EPI_ISL_1040824, EPI_ISL_1040825, EPI_ISL_1040826, EPI_ISL_1040827, EPI_ISL_1040828, EPI_ISL_1040829, EPI_ISL_1040830, EPI_ISL_1040831, EPI_ISL_1040832, EPI_ISL_1040833, EPI_ISL_1040834, EPI_ISL_1040835, EPI_ISL_1040836, EPI_ISL_1040837, EPI_ISL_1040838, EPI_ISL_1040839, EPI_ISL_1040840, EPI_ISL_1040841, EPI_ISL_1040842, EPI_ISL_1040843, EPI_ISL_1040844, EPI_ISL_1040845, EPI_ISL_1040846, EPI_ISL_1040847, EPI_ISL_1040848, EPI_ISL_1040849, EPI_ISL_1040850, EPI_ISL_1040851			
see above	LACEN do Mato Grosso do Sul	Instituto Adolfo Lutz, Interdiciplinary Procedures Center, Strategic Laboratory	Claudio Tavares Sacchi, Claudia Regina Gonçalves, Erica Valessa Ramos Gomes, Karoline Rodrigues Campos
EPI_ISL_1041509	LACEN do Estado de Goias	Instituto Adolfo Lutz, Interdiciplinary Procedures Center, Strategic Laboratory	Claudio Tavares Sacchi, Claudia Regina Gonçalves, Erica Valessa Ramos Gomes, Karoline Rodrigues Campos
EPI_ISL_1061031, EPI_ISL_1061032	CDL Laboratorio Santos e Vidal LTDA.	Instituto de Medicina Tropical de Sao Paulo	Brazil-UK Centre for Arbovirus Discovery Diagnosis Genomics and Epidemiology (CADDE) Genomic Network - Instituto de Medicina Tropical
EPI_ISL_1063789	Evandro Chagas Institute	Evandro Chagas Institute Virology	Santos, M.C.; Silva, A.M.; Junior, W.D.C.; Barbagelata, L.S.; Ferreira, J.A.; Sousa, E.M.A.; da Silva, P.S.; Pinheiro, K.C.; L.C.; Sousa Junior, E.C.
EPI_ISL_1067728	Center for Biotechnology and Cell Therapy, São Rafael Hospital, Salvador, Brazil	Central Public Health Laboratory - LACEN -Bahia, Salvador, Brazil	Stephane Tosta, Luciana Oliveira, Vanessa Nardy,Patrícia Cajado,Marcela Gómez, Breno Dominguez, Jaqueline Gomes, Vagner Fonseca,Marta Giovanetti,Luiz Alcantara, Felicidade Pereira, Arabela Leal
EPI_ISL_1067729, EPI_ISL_1067730, EPI_ISL_1067731	Central Public Health Laboratory - LACEN -Bahia, Salvador, Brazil	Central Public Health Laboratory - LACEN -Bahia, Salvador, Brazil	Stephane Tosta, Luciana Oliveira, Vanessa Nardy,Patrícia Cajado,Marcela Gómez, Breno Dominguez, Jaqueline Gomes, Vagner Fonseca,Marta Giovanetti,Luiz Alcantara, Felicidade Pereira, Arabela Leal
EPI_ISL_1067732	Center for Biotechnology and Cell Therapy, São Rafael Hospital, Salvador, Brazil	Central Public Health Laboratory - LACEN -Bahia, Salvador, Brazil	Stephane Tosta, Luciana Oliveira, Vanessa Nardy,Patrícia Cajado,Marcela Gómez, Breno Dominguez, Jaqueline Gomes, Vagner Fonseca,Marta Giovanetti,Luiz Alcantara, Felicidade Pereira, Arabela Leal
EPI_ISL_1067733, EPI_ISL_1067734, EPI_ISL_1067735	Central Public Health Laboratory - LACEN -Bahia, Salvador, Brazil	Central Public Health Laboratory - LACEN -Bahia, Salvador, Brazil	Stephane Tosta, Luciana Oliveira, Vanessa Nardy,Patrícia Cajado,Marcela Gómez, Breno Dominguez, Jaqueline Gomes, Vagner Fonseca,Marta Giovanetti,Luiz Alcantara, Felicidade Pereira, Arabela Leal
EPI_ISL_1067736	Center for Biotechnology and Cell Therapy, São Rafael Hospital, Salvador, Brazil	Central Public Health Laboratory - LACEN -Bahia, Salvador, Brazil	Stephane Tosta, Luciana Oliveira, Vanessa Nardy,Patrícia Cajado,Marcela Gómez, Breno Dominguez, Jaqueline Gomes, Vagner Fonseca,Marta Giovanetti,Luiz Alcantara, Felicidade Pereira, Arabela Leal
EPI_ISL_1067737, EPI_ISL_1067738	Central Public Health Laboratory - LACEN -Bahia, Salvador, Brazil	Central Public Health Laboratory - LACEN -Bahia, Salvador, Brazil	Stephane Tosta, Luciana Oliveira, Vanessa Nardy,Patrícia Cajado,Marcela Gómez, Breno Dominguez, Jaqueline Gomes, Vagner Fonseca,Marta Giovanetti,Luiz Alcantara, Felicidade Pereira, Arabela Leal
EPI_ISL_1068078, EPI_ISL_1068079, EPI_ISL_1068080, EPI_ISL_1068081, EPI_ISL_1068082, EPI_ISL_1068083, EPI_ISL_1068084, EPI_ISL_1068085, EPI_ISL_1068086, EPI_ISL_1068087, EPI_ISL_1068088, EPI_ISL_1068089, EPI_ISL_1068090, EPI_ISL_1068091, EPI_ISL_1068092, EPI_ISL_1068093, EPI_ISL_1068094, EPI_ISL_1068095, EPI_ISL_1068096, EPI_ISL_1068097, EPI_ISL_1068098, EPI_ISL_1068099, EPI_ISL_1068100, EPI_ISL_1068101, EPI_ISL_1068102, EPI_ISL_1068103, EPI_ISL_1068104, EPI_ISL_1068105, EPI_ISL_1068106, EPI_ISL_1068107, EPI_ISL_1068108, EPI_ISL_1068109, EPI_ISL_1068110, EPI_ISL_1068111, EPI_ISL_1068112, EPI_ISL_1068113, EPI_ISL_1068114, EPI_ISL_1068115, EPI_ISL_1068117, EPI_ISL_1068118, EPI_ISL_1068119, EPI_ISL_1068120, EPI_ISL_1068121, EPI_ISL_1068122, EPI_ISL_1068123, EPI_ISL_1068124, EPI_ISL_1068125, EPI_ISL_1068126, EPI_ISL_1068127, EPI_ISL_1068128, EPI_ISL_1068129, EPI_ISL_1068130, EPI_ISL_1068131, EPI_ISL_1068132, EPI_ISL_1068133, EPI_ISL_1068134, EPI_ISL_1068135, EPI_ISL_1068136, EPI_ISL_1068137, EPI_ISL_1068138, EPI_ISL_1068139, EPI_ISL_1068140, EPI_ISL_1068141, EPI_ISL_1068142, EPI_ISL_1068143, EPI_ISL_1068144, EPI_ISL_1068145, EPI_ISL_1068146, EPI_ISL_1068147, EPI_ISL_1068148, EPI_ISL_1068149, EPI_ISL_1068150, EPI_ISL_1068151, EPI_ISL_1068152, EPI_ISL_1068153, EPI_ISL_1068154, EPI_ISL_1068155, EPI_ISL_1068156, EPI_ISL_1068157, EPI_ISL_1068158, EPI_ISL_1068159, EPI_ISL_1068160, EPI_ISL_1068161, EPI_ISL_1068162, EPI_ISL_1068163, EPI_ISL_1068164, EPI_ISL_1068165, EPI_ISL_1068166, EPI_ISL_1068167, EPI_ISL_1068168, EPI_ISL_1068169, EPI_ISL_1068170, EPI_ISL_1068171, EPI_ISL_1068172, EPI_ISL_1068173, EPI_ISL_1068174, EPI_ISL_1068175, EPI_ISL_1068176, EPI_ISL_1068177, EPI_ISL_1068178, EPI_ISL_1068179, EPI_ISL_1068180, EPI_ISL_1068181, EPI_ISL_1068182, EPI_ISL_1068183, EPI_ISL_1068184, EPI_ISL_1068185, EPI_ISL_1068186, EPI_ISL_1068187, EPI_ISL_1068188, EPI_ISL_1068189, EPI_ISL_1068190, EPI_ISL_1068191, EPI_ISL_1068192, EPI_ISL_1068193, EPI_ISL_1068194, EPI_ISL_1068195, EPI_ISL_1068196, EPI_ISL_1068197, EPI_ISL_1068198, EPI_ISL_1068201, EPI_ISL_1068203, EPI_ISL_1068204, EPI_ISL_1068205, EPI_ISL_1068206, EPI_ISL_1068207, EPI_ISL_1068208, EPI_ISL_1068209, EPI_ISL_1068210, EPI_ISL_1068211, EPI_ISL_1068212, EPI_ISL_1068213, EPI_ISL_1068214, EPI_ISL_1068215, EPI_ISL_1068216, EPI_ISL_1068217, EPI_ISL_1068218, EPI_ISL_1068219, EPI_ISL_1068221, EPI_ISL_1068222, EPI_ISL_1068223, EPI_ISL_1068224, EPI_ISL_1068225, EPI_ISL_1068226, EPI_ISL_1068227, EPI_ISL_1068228, EPI_ISL_1068229, EPI_ISL_1068230, EPI_ISL_1068231, EPI_ISL_1068232, EPI_ISL_1068233, EPI_ISL_1068234, EPI_ISL_1068235, EPI_ISL_1068236, EPI_ISL_1068237, EPI_ISL_1068238, EPI_ISL_1068239, EPI_ISL_1068240, EPI_ISL_1068241, EPI_ISL_1068242, EPI_ISL_1068243, EPI_ISL_1068244, EPI_ISL_1068245, EPI_ISL_1068246, EPI_ISL_1068247, EPI_ISL_1068248, EPI_ISL_1068249, EPI_ISL_1068250, EPI_ISL_1068251, EPI_ISL_1068252,			

We gratefully acknowledge the following Authors from the Originating laboratories responsible for obtaining the specimens, as well as the Submitting laboratories where the genome data were generated and shared via GISAID, on which this research is based.

All Submitters of data may be contacted directly via www.gisaid.org

Authors are sorted alphabetically.

Accession ID	Originating Laboratory	Submitting Laboratory	Authors
EPI_ISL_1578453	Laboratório de Biologia Molecular do Hospital das Clínicas da Faculdade de Medicina de Botucatu/SP	Laboratórios Genômica Funcional (FCA/UNESP) e Biologia Molecular (FMB-HC/UNESP) - Rede de Vigilância Genômica (Vigenômica)/UNESP	Patrícia Akemi Assato; Felipe Allan da Silva da Costa; Bianca Cechetto Carlos; Flavia Hebner Barbosa Trovão; Guilherme Targino Valente; Rejane Maria Tommasini Grotto; Jayme A. Souza-Neto.
EPI_ISL_1578740, EPI_ISL_1579258, EPI_ISL_1580269, EPI_ISL_1580502	Laboratório de Biologia Molecular do Hospital das Clínicas da Faculdade de Medicina de Botucatu/SP	Laboratórios de Genômica Funcional (FCA/UNESP) e Biologia Molecular (FMB-HC/UNESP) - Rede de Vigilância Genômica (Vigenômica)/UNESP	Patrícia Akemi Assato; Felipe Allan da Silva da Costa; Bianca Cechetto Carlos; Flavia Hebner Barbosa Trovão; Guilherme Targino Valente; Rejane Maria Tommasini Grotto; Jayme A. Souza-Neto.
EPI_ISL_1664123, EPI_ISL_1664124, EPI_ISL_1664125, EPI_ISL_1664126, EPI_ISL_1664127, EPI_ISL_1664128, EPI_ISL_1664129, EPI_ISL_1664130, EPI_ISL_1664131, EPI_ISL_1664132, EPI_ISL_1664133, EPI_ISL_1664134, EPI_ISL_1664135, EPI_ISL_1664136, EPI_ISL_1664137, EPI_ISL_1664138, EPI_ISL_1664139, EPI_ISL_1664140, EPI_ISL_1664141, EPI_ISL_1664142, EPI_ISL_1664143, EPI_ISL_1664144, EPI_ISL_1664145, EPI_ISL_1664146, EPI_ISL_1664147, EPI_ISL_1664148, EPI_ISL_1664158, EPI_ISL_1664159, EPI_ISL_1664160, EPI_ISL_1664161, EPI_ISL_1664162, EPI_ISL_1664163, EPI_ISL_1664174, EPI_ISL_1664175, EPI_ISL_1664176, EPI_ISL_1664177, EPI_ISL_1664178, EPI_ISL_1664179, EPI_ISL_1664180, EPI_ISL_1664181, EPI_ISL_1664182, EPI_ISL_1664183, EPI_ISL_1664184, EPI_ISL_1664185, EPI_ISL_1664186, EPI_ISL_1664187, EPI_ISL_1664188, EPI_ISL_1664189, EPI_ISL_1664190, EPI_ISL_1664191, EPI_ISL_1664192, EPI_ISL_1664193, EPI_ISL_1664194, EPI_ISL_1664195, EPI_ISL_1664196, EPI_ISL_1664197, EPI_ISL_1664198, EPI_ISL_1664199, EPI_ISL_1664200, EPI_ISL_1664201, EPI_ISL_1664202			
see above	Laboratorio Central Noel Nutels	Bioinformatics Laboratory / LNCC	Luiz G P de Almeida, Alessandra P Lamarca, Ronaldo da Silva F Jr, Liliane Cavalcante, Alexandra L Gerber, Ana Paula de C Guimarães, Douglas Terra Machado, Cassia Alves, Diana Mariani, Thais Felix Cruz, Mario Sergio Ribeiro, Silvia Carvalho, Flávio Dias da Silva, Marcio Henrique de Oliveira Garcia, Leandro Magalhães de Souza, Cristiane Gomes da Silva, Caio Luiz Pereira Ribeiro, Andréa Cony Cavalcanti, Claudia Maria Braga de Mello, Amilcar Tanuri, Ana Tereza R Vasconcelos
EPI_ISL_1707689, EPI_ISL_1707690, EPI_ISL_1707691, EPI_ISL_1707692	Instituto Adolfo Lutz - Regional de Taubate	Instituto Adolfo Lutz, Interdiciplinary Procedures Center, Strategic Laboratory	Claudio Tavares Sacchi, Claudia Regina Gonçalves, Erica Valessa Ramos Gomes, Karoline Rodrigues Campos, Caio Vinicius Dias Lopes, Leonardo Jose Tadeu de Araujo, Katia Correa de Oliveira Santos
EPI_ISL_1715146, EPI_ISL_1715147	Instituto Adolfo Lutz - Regional de Aracatuba	Instituto Adolfo Lutz, Interdiciplinary Procedures Center, Strategic Laboratory	Claudio Tavares Sacchi, Claudia Regina Gonçalves, Erica Valessa Ramos Gomes, Karoline Rodrigues Campos, Caio Vinicius Dias Lopes, Leonardo Jose Tadeu de Araujo, Katia Correa de Oliveira Santos
EPI_ISL_1715148	Instituto Adolfo Lutz - Regional de Ribeirao Preto	Instituto Adolfo Lutz, Interdiciplinary Procedures Center, Strategic Laboratory	Claudio Tavares Sacchi, Claudia Regina Gonçalves, Erica Valessa Ramos Gomes, Karoline Rodrigues Campos, Caio Vinicius Dias Lopes, Leonardo Jose Tadeu de Araujo, Katia Correa de Oliveira Santos
EPI_ISL_1715149, EPI_ISL_1715150, EPI_ISL_1715151, EPI_ISL_1715152, EPI_ISL_1715153, EPI_ISL_1715154, EPI_ISL_1715155, EPI_ISL_1715156, EPI_ISL_1715157, EPI_ISL_1715158	Instituto Adolfo Lutz Central	Instituto Adolfo Lutz, Interdiciplinary Procedures Center, Strategic Laboratory	Claudio Tavares Sacchi, Claudia Regina Gonçalves, Erica Valessa Ramos Gomes, Karoline Rodrigues Campos, Caio Vinicius Dias Lopes, Leonardo Jose Tadeu de Araujo, Katia Correa de Oliveira Santos
EPI_ISL_1731577	Hospital Municipal Dr Mario Gatti Campinas	Instituto Adolfo Lutz, Interdiciplinary Procedures Center, Strategic Laboratory	Claudio Tavares Sacchi, Claudia Regina Gonçalves, Erica Valessa Ramos Gomes, Karoline Rodrigues Campos, Caio Vinicius Dias Lopes, Leonardo Jose Tadeu de Araujo, Katia Correa de Oliveira Santos
EPI_ISL_1731578, EPI_ISL_1731579, EPI_ISL_1731580	Instituto Adolfo Lutz - Regional de Aracatuba	Instituto Adolfo Lutz, Interdiciplinary Procedures Center, Strategic Laboratory	Claudio Tavares Sacchi, Claudia Regina Gonçalves, Erica Valessa Ramos Gomes, Karoline Rodrigues Campos, Caio Vinicius Dias Lopes, Leonardo Jose Tadeu de Araujo, Katia Correa de Oliveira Santos
EPI_ISL_1731582	Instituto Adolfo Lutz - Regional de Ribeirao Preto	Instituto Adolfo Lutz, Interdiciplinary Procedures Center, Strategic Laboratory	Claudio Tavares Sacchi, Claudia Regina Gonçalves, Erica Valessa Ramos Gomes, Karoline Rodrigues Campos, Caio Vinicius Dias Lopes, Leonardo Jose Tadeu de Araujo, Katia Correa de Oliveira Santos
EPI_ISL_1731583, EPI_ISL_1731584, EPI_ISL_1731585, EPI_ISL_1731586, EPI_ISL_1731587, EPI_ISL_1731588, EPI_ISL_1731589, EPI_ISL_1731590, EPI_ISL_1731591, EPI_ISL_1731592, EPI_ISL_1731593, EPI_ISL_1731594, EPI_ISL_1731595, EPI_ISL_1731596, EPI_ISL_1731597, EPI_ISL_1731604, EPI_ISL_1731605, EPI_ISL_1731606, EPI_ISL_1731607, EPI_ISL_1731608, EPI_ISL_1731609, EPI_ISL_1731610, EPI_ISL_1731611, EPI_ISL_1731612			
see above	Instituto Adolfo Lutz Central	Instituto Adolfo Lutz, Interdiciplinary Procedures Center, Strategic Laboratory	Claudio Tavares Sacchi, Claudia Regina Gonçalves, Erica Valessa Ramos Gomes, Karoline Rodrigues Campos, Caio Vinicius Dias Lopes, Leonardo Jose Tadeu de Araujo, Katia Correa de Oliveira Santos
EPI_ISL_1732275	SECAO CENTRO DE DIAGNOSTICO SECEDI	Instituto Butantan / Mendelics	Instituto Butantan: Alexander Roberto Precioso, Dimas Tadeu Covas, Sandra Coccuzzo Sampaio, Maria Carolina Elias, José Salvatore Leister Patané, Vincent Louis Viala, Antonio Jorge Martins, Ricardo Haddad, Claudia Renata dos Santos Barros, Elaine Cristina Marqueze, Raul Machado Neto, Debora Botequio Moretti. Centro de Genômica Funcional da ESALQ: Luiz Lehmann Coutinho, Ricardo Augusto Brassaloti, Raquel de Lello Rocha Campos Cassano. NGS Soluções Genômicas: Pilar Drummond Sampaio Corrêa Mariani. FZEA-USP Pirassununga: Mirele Daiana Poleti, Jessika Cristina Chagas Lesbon, Elisangela Chicaroni Mattos, Heidge Fukumasu. USP-Botucatu: Rejane Maria Tommasini Grotto, Jayme A. Souza-Neto, Guilherme Targino Valente, Patricia Akemi Assato, Felipe Allan da Silva da Costa, Bianca Cechetto Carlos. Mendelics: Bibiana Santos, João Paulo Kitajima, Erika Freitas, David Schlesinger. Hemocentro Ribeirão Preto: Simone Kashima, Evandra Strazza Rodrigues, Svetoslav Nanev Slavov, Elaine Vieira dos Santos, Rafael dos Santos Bezerra, Luiz Carlos Junior de Alcantara, Marta Giovanetti, Vagner Fonseca, Flavia Aburjaile, Rodrigo Tocantins Calado.
EPI_ISL_1734843, EPI_ISL_1734844, EPI_ISL_1734846, EPI_ISL_1734847, EPI_ISL_1734849, EPI_ISL_1734850, EPI_ISL_1734852, EPI_ISL_1734853, EPI_ISL_1734855, EPI_ISL_1734857, EPI_ISL_1734858, EPI_ISL_1734860, EPI_ISL_1734861, EPI_ISL_1734863, EPI_ISL_1734864, EPI_ISL_1734866, EPI_ISL_1734868, EPI_ISL_1734869, EPI_ISL_1734871, EPI_ISL_1734872, EPI_ISL_1734874, EPI_ISL_1734875, EPI_ISL_1734877, EPI_ISL_1734878, EPI_ISL_1734880, EPI_ISL_1734881, EPI_ISL_1734883, EPI_ISL_1734884			
see above	Instituto de Biotecnologia - UNESP-Botucatu-SP	Instituto de Biotecnologia - UNESP-Botucatu-SP	Fábio Sossai Possebon; Leila Sabrina Ullmann; Cecília Artico Banho; Cíntia Bittar; Guilherme Campos; Helena Lage Ferreira; Jorge A. Petroli Marchesi; Lívia Sacchetto; Maisa C. Pereira Parra; Marília Moraes; Maurício L. Nogueira; Paula Rahal; Paulo Inacio da Costa; João Pessoa Araújo Jr.
EPI_ISL_1752638	Casa de Caridade Sao Vicente de Paulo Cajuru	Instituto Adolfo Lutz, Interdiciplinary Procedures Center, Strategic Laboratory	Claudio Tavares Sacchi, Claudia Regina Gonçalves, Erica Valessa Ramos Gomes, Karoline Rodrigues Campos, Caio Vinicius Dias Lopes, Leonardo Jose Tadeu de Araujo, Katia Correa de Oliveira Santos
EPI_ISL_1752639	UPA Dr Luis Atilio Losi Viana Ribeirao Preto	Instituto Adolfo Lutz, Interdiciplinary Procedures Center, Strategic Laboratory	Claudio Tavares Sacchi, Claudia Regina Gonçalves, Erica Valessa Ramos Gomes, Karoline Rodrigues Campos, Caio Vinicius Dias Lopes, Leonardo Jose Tadeu de Araujo, Katia Correa de Oliveira Santos
EPI_ISL_1752640	Hospital Municipal Dr Mario Gatti Campinas	Instituto Adolfo Lutz, Interdiciplinary Procedures Center, Strategic Laboratory	Claudio Tavares Sacchi, Claudia Regina Gonçalves, Erica Valessa Ramos Gomes, Karoline Rodrigues Campos, Caio Vinicius Dias Lopes, Leonardo Jose Tadeu de Araujo, Katia Correa de Oliveira Santos
EPI_ISL_1752641, EPI_ISL_1752642, EPI_ISL_1752643	Instituto Adolfo Lutz - Regional de Aracatuba	Instituto Adolfo Lutz, Interdiciplinary Procedures Center, Strategic Laboratory	Claudio Tavares Sacchi, Claudia Regina Gonçalves, Erica Valessa Ramos Gomes, Karoline Rodrigues Campos, Caio Vinicius Dias Lopes, Leonardo Jose Tadeu de Araujo, Katia Correa de Oliveira Santos
EPI_ISL_1752644, EPI_ISL_1752645	Instituto Adolfo Lutz - Regional de Ribeirao Preto	Instituto Adolfo Lutz, Interdiciplinary Procedures Center, Strategic Laboratory	Claudio Tavares Sacchi, Claudia Regina Gonçalves, Erica Valessa Ramos Gomes, Karoline Rodrigues Campos, Caio Vinicius Dias Lopes, Leonardo Jose Tadeu de Araujo, Katia Correa de Oliveira Santos
EPI_ISL_1752646, EPI_ISL_1752647	Instituto Adolfo Lutz Central	Instituto Adolfo Lutz, Interdiciplinary Procedures Center, Strategic Laboratory	Claudio Tavares Sacchi, Claudia Regina Gonçalves, Erica Valessa Ramos Gomes, Karoline Rodrigues Campos, Caio Vinicius Dias Lopes, Leonardo Jose Tadeu de Araujo, Katia Correa de Oliveira Santos
EPI_ISL_1795099	ESALQ	Instituto Butantan / ESALQ-Piracicaba	Instituto Butantan: Alexander Roberto Precioso, Dimas Tadeu Covas, Sandra Coccuzzo Sampaio, Maria Carolina Elias, José Salvatore Leister Patané, Vincent Louis Viala, Antonio Jorge Martins, Ricardo Haddad, Claudia Renata dos Santos Barros, Elaine Cristina Marqueze, Raul Machado Neto, Debora Botequio Moretti. Centro de Genômica Funcional da ESALQ: Luiz Lehmann Coutinho, Ricardo Augusto Brassaloti, Raquel de Lello Rocha Campos Cassano. NGS Soluções Genômicas: Pilar Drummond Sampaio Corrêa Mariani. FZEA-USP Pirassununga: Mirele Daiana Poleti, Jessika Cristina Chagas Lesbon, Elisangela Chicaroni Mattos, Heidge Fukumasu. USP-Botucatu: Rejane Maria Tommasini Grotto, Jayme A. Souza-Neto, Guilherme Targino

India

We gratefully acknowledge the following Authors from the Originating laboratories responsible for obtaining the specimens, as well as the Submitting laboratories where the genome data were generated and shared via GISAID, on which this research is based.

All Submitters of data may be contacted directly via www.gisaid.org

Authors are sorted alphabetically.

Accession ID	Originating Laboratory	Submitting Laboratory	Authors
EPI_ISL_1034155, EPI_ISL_1034157, EPI_ISL_1034158, EPI_ISL_1034159, EPI_ISL_1034162, EPI_ISL_1034163, EPI_ISL_1034166, EPI_ISL_1034167, EPI_ISL_1034168, EPI_ISL_1034171, EPI_ISL_1034173, EPI_ISL_1034175, EPI_ISL_1034177, EPI_ISL_1034180, EPI_ISL_1034181, EPI_ISL_1034184, EPI_ISL_1034185, EPI_ISL_1034186, EPI_ISL_1034188, EPI_ISL_1034189			
see above	NIV Influenza	NIV Influenza	Potdar V
EPI_ISL_1039249, EPI_ISL_1055383	Virus Research and Diagnostic Laboratory, Medical College Level, Kasturba Hospital for Infectious Diseases	Indian Council of Medical Research-National Institute of Virology, Microbial Containment Complex	Pragya D. Yadav, Jayanti Shastri
EPI_ISL_1055384, EPI_ISL_1055768, EPI_ISL_1055770, EPI_ISL_1055771, EPI_ISL_1055785, EPI_ISL_1055788, EPI_ISL_1055789	Virus Research and Diagnostic Laboratory, Medical College Level, Kasturba Hospital for Infectious Diseases	National Institute of Virology-Microbial Containment Complex, Indian Council of Medical Research	Pragya D. Yadav, Jayanti Shastri
EPI_ISL_1061417, EPI_ISL_1061426, EPI_ISL_1061428, EPI_ISL_1061433, EPI_ISL_1061434	Indian Council of Medical Research-National Institute of Virology, Microbial Containment Complex	Indian Council of Medical Research-National Institute of Virology, Microbial Containment Complex	Pragya D. Yadav
EPI_ISL_1063400, EPI_ISL_1063401	Virus Research and Diagnostic Laboratory, Medical College Level, Kasturba Hospital for Infectious Diseases	National Institute of Virology-Microbial Containment Complex, Indian Council of Medical Research	Pragya D. Yadav, Jayanti Shastri
EPI_ISL_1063403	PGIMER, Chandigarh	ICMR-NATIONAL INSTITUTE OF VIROLOGY, MICROBIAL CONTAINMENT COMPLEX	Pragya D. Yadav, Rupinder Bakshi
EPI_ISL_1063494, EPI_ISL_1063526, EPI_ISL_1063527	Virus Research and Diagnostic Laboratory, Medical College Level, Kasturba Hospital for Infectious Diseases	National Institute of Virology-Microbial Containment Complex, Indian Council of Medical Research	Pragya D. Yadav, Jayanti Shastri
EPI_ISL_1063528	PGIMER, Chandigarh	ICMR-NATIONAL INSTITUTE OF VIROLOGY, MICROBIAL CONTAINMENT COMPLEX	Pragya D. Yadav, Rupinder Bakshi
EPI_ISL_1063529	Virus Research and Diagnostic Laboratory, Medical College Level, Kasturba Hospital for Infectious Diseases	National Institute of Virology-Microbial Containment Complex, Indian Council of Medical Research	Pragya D. Yadav, Jayanti Shastri
EPI_ISL_1063530	PGIMER, Chandigarh	ICMR-NATIONAL INSTITUTE OF VIROLOGY, MICROBIAL CONTAINMENT COMPLEX	Pragya D. Yadav, Rupinder Bakshi
EPI_ISL_1063531	Virus Research and Diagnostic Laboratory, Medical College Level, Kasturba Hospital for Infectious Diseases	National Institute of Virology-Microbial Containment Complex, Indian Council of Medical Research	Pragya D. Yadav, Jayanti Shastri
EPI_ISL_1063532, EPI_ISL_1063534, EPI_ISL_1063535, EPI_ISL_1063536, EPI_ISL_1063627	PGIMER, Chandigarh	ICMR-NATIONAL INSTITUTE OF VIROLOGY, MICROBIAL CONTAINMENT COMPLEX	Pragya D. Yadav, Rupinder Bakshi
EPI_ISL_1063774, EPI_ISL_1063775	PGIMER, Chandigarh	ICMR-NATIONAL INSTITUTE OF VIROLOGY, MICROBIAL CONTAINMENT COMPLEX	Pragya D. Yadav, Mini Singh
EPI_ISL_1063776	PGIMER, Chandigarh	ICMR-NATIONAL INSTITUTE OF VIROLOGY, MICROBIAL CONTAINMENT COMPLEX	Pragya D. Yadav, Rupinder Bakshi
EPI_ISL_1063777	PGIMER, Chandigarh	ICMR-NATIONAL INSTITUTE OF VIROLOGY, MICROBIAL CONTAINMENT COMPLEX	Pragya D. Yadav, Mini Singh
EPI_ISL_1063778, EPI_ISL_1063779	PGIMER, Chandigarh	ICMR-NATIONAL INSTITUTE OF VIROLOGY, MICROBIAL CONTAINMENT COMPLEX	Pragya D. Yadav, Rupinder Bakshi
EPI_ISL_1063780, EPI_ISL_1063781, EPI_ISL_1063782, EPI_ISL_1063783	PGIMER, Chandigarh	ICMR-NATIONAL INSTITUTE OF VIROLOGY, MICROBIAL CONTAINMENT COMPLEX	Pragya D. Yadav, Mini Singh
EPI_ISL_1063806	Molecular Laboratory, Vikash Multispeciality Hospital, Bargarh	Immunogenomics lab, Institute of Life Sciences, Bhubaneswar	Sunil K. Raghav, Safal Walia, Arup Ghosh, Atimukta Jha, Amol M. Kanampalliwar, Shifu Aggarwal, Kirtal Hansdah, Rupesh Dash, Rajeeb Swain, Punit Prasad, INSACOG Consortium, Ajay Parida
EPI_ISL_1063807	REGIONAL VRDL, ICMR-RMRC BBSR	Immunogenomics lab, Institute of Life Sciences, Bhubaneswar	Sunil K. Raghav, Safal Walia, Arup Ghosh, Atimukta Jha, Amol M. Kanampalliwar, Shifu Aggarwal, Kirtal Hansdah, Rupesh Dash, Rajeeb Swain, Punit Prasad, INSACOG Consortium, Ajay Parida
EPI_ISL_1063808	Immunogenomics lab, Institute of Life Sciences, Bhubaneswar	Immunogenomics lab, Institute of Life Sciences, Bhubaneswar	Sunil K. Raghav, Safal Walia, Arup Ghosh, Atimukta Jha, Amol M. Kanampalliwar, Shifu Aggarwal, Kirtal Hansdah, Rupesh Dash, Rajeeb Swain, Punit Prasad, INSACOG Consortium, Ajay Parida
EPI_ISL_1063809	REGIONAL VRDL, ICMR-RMRC BBSR	Immunogenomics lab, Institute of Life Sciences, Bhubaneswar	Sunil K. Raghav, Safal Walia, Arup Ghosh, Atimukta Jha, Amol M. Kanampalliwar, Shifu Aggarwal, Kirtal Hansdah, Rupesh Dash, Rajeeb Swain, Punit Prasad, INSACOG Consortium, Ajay Parida
EPI_ISL_1063810, EPI_ISL_1063811, EPI_ISL_1063812, EPI_ISL_1063813, EPI_ISL_1063814, EPI_ISL_1063815, EPI_ISL_1063816, EPI_ISL_1063817, EPI_ISL_1063818, EPI_ISL_1063819, EPI_ISL_1063820, EPI_ISL_1063821, EPI_ISL_1063822, EPI_ISL_1063823, EPI_ISL_1063824, EPI_ISL_1063825, EPI_ISL_1063826, EPI_ISL_1063827, EPI_ISL_1063828, EPI_ISL_1063829, EPI_ISL_1063830, EPI_ISL_1063831, EPI_ISL_1063832, EPI_ISL_1063833, EPI_ISL_1063834, EPI_ISL_1063835, EPI_ISL_1063836, EPI_ISL_1063837, EPI_ISL_1063838, EPI_ISL_1063839			
see above	Immunogenomics lab, Institute of Life Sciences, Bhubaneswar	Immunogenomics lab, Institute of Life Sciences, Bhubaneswar	Sunil K. Raghav, Safal Walia, Arup Ghosh, Atimukta Jha, Amol M. Kanampalliwar, Shifu Aggarwal, Kirtal Hansdah, Rupesh Dash, Rajeeb Swain, Punit Prasad, INSACOG Consortium, Ajay Parida
EPI_ISL_1063840, EPI_ISL_1063841, EPI_ISL_1063842, EPI_ISL_1063843, EPI_ISL_1063844, EPI_ISL_1063845, EPI_ISL_1063846, EPI_ISL_1063847, EPI_ISL_1063848, EPI_ISL_1063849, EPI_ISL_1063850, EPI_ISL_1063851			
see above	REGIONAL VRDL, ICMR-RMRC BBSR	Immunogenomics lab, Institute of Life Sciences, Bhubaneswar	Sunil K. Raghav, Safal Walia, Arup Ghosh, Atimukta Jha, Amol M. Kanampalliwar, Shifu Aggarwal, Kirtal Hansdah, Rupesh Dash, Rajeeb Swain, Punit Prasad, INSACOG Consortium, Ajay Parida
EPI_ISL_1063852, EPI_ISL_1063853, EPI_ISL_1063854	inDNA Life Sciences Private Limited, Bhubaneswar	Immunogenomics lab, Institute of Life Sciences, Bhubaneswar	Sunil K. Raghav, Safal Walia, Arup Ghosh, Atimukta Jha, Amol M. Kanampalliwar, Shifu Aggarwal, Kirtal Hansdah, Rupesh Dash, Rajeeb Swain, Punit Prasad, INSACOG Consortium, Ajay Parida
EPI_ISL_1063855, EPI_ISL_1063856, EPI_ISL_1063857, EPI_ISL_1063858, EPI_ISL_1063859, EPI_ISL_1063860, EPI_ISL_1063861	Molecular Laboratory, Vikash Multispeciality Hospital, Bargarh	Immunogenomics lab, Institute of Life Sciences, Bhubaneswar	Sunil K. Raghav, Safal Walia, Arup Ghosh, Atimukta Jha, Amol M. Kanampalliwar, Shifu Aggarwal, Kirtal Hansdah, Rupesh Dash, Rajeeb Swain, Punit Prasad, INSACOG Consortium, Ajay Parida
EPI_ISL_1063862, EPI_ISL_1063863, EPI_ISL_1063864, EPI_ISL_1063865, EPI_ISL_1063866, EPI_ISL_1063867, EPI_ISL_1063868, EPI_ISL_1063869, EPI_ISL_1063870, EPI_ISL_1063871, EPI_ISL_1063872, EPI_ISL_1063873, EPI_ISL_1063874, EPI_ISL_1063875, EPI_ISL_1063876, EPI_ISL_1063877, EPI_ISL_1063878,			

We gratefully acknowledge the following Authors from the Originating laboratories responsible for obtaining the specimens, as well as the Submitting laboratories where the genome data were generated and shared via GISAID, on which this research is based.

All Submitters of data may be contacted directly via www.gisaid.org

Authors are sorted alphabetically.

Accession ID	Originating Laboratory	Submitting Laboratory	Authors
EPI_ISL_1357695, EPI_ISL_1357697, EPI_ISL_1357698, EPI_ISL_1357699, EPI_ISL_1357700, EPI_ISL_1357702, EPI_ISL_1357703, EPI_ISL_1357705, EPI_ISL_1357706	INSACOG-WB	National Institute of Biomedical Genomics	Arindam Maitra, Bhaswati Bandyopadhyay, Nidhan Kumar Biswas, Tamal Ghosh, Sreedhar Chinnaswamy, Ajay Chakraborti, Saumitra Das
EPI_ISL_1360383	CSIR-National Environmental Engineering Research Institute	CSIR-Centre for Cellular and Molecular Biology - INSACOG	Lamuk Zaveri, Ara Sreenivas, Sofia Banu, Onkar Kulkarni, Shreekant Verma, Amareshwar Vodapalli , Viswagithe S L, B Himasri, Sharath Chandra Thota, Karthik Bharadwaj Tallapaka, Krishna Khairnar, Rakesh K Mishra,Divya Tej Sowpati
EPI_ISL_1360384	CSIR-National Environmental Engineering Research Institute	CSIR-Centre for Cellular and Molecular Biology - INSACOG	Lamuk Zaveri, Ara Sreenivas, Onkar Kulkarni, Sofia Banu, Shreekant Verma,Amareshwar Vodapalli, Viswagithe S L, B Himasri, Sharath Chandra Thota, Karthik Bharadwaj Tallapaka, Krishna Khairnar, Rakesh K Mishra,Divya Tej Sowpati
EPI_ISL_1360385	CSIR-National Environmental Engineering Research Institute	CSIR-Centre for Cellular and Molecular Biology - INSACOG	Ara Sreenivas, Onkar Kulkarni, Lamuk Zaveri, Sofia Banu, Shreekant Verma, Amareshwar Vodapalli , Viswagithe S L, B Himasri, Sharath Chandra Thota, Karthik Bharadwaj Tallapaka, Krishna Khairnar, Rakesh K Mishra,Divya Tej Sowpati
EPI_ISL_1360387, EPI_ISL_1360388	CSIR-National Environmental Engineering Research Institute	CSIR-Centre for Cellular and Molecular Biology - INSACOG	Sofia Banu, Lamuk Zaveri, Ara Sreenivas, Shreekant Verma, Onkar Kulkarni, Amareshwar Vodapalli ,Viswagithe S L, B Himasri, Sharath Chandra Thota, Karthik Bharadwaj Tallapaka, Krishna Khairnar, Rakesh K Mishra,Divya Tej Sowpati
EPI_ISL_1372095	Nalanda Medical College Hospital, Patna	Institute of Life Sciences - INSACOG	Sunil K. Raghav, Safal Walia, Arup Ghosh, Atimukta Jha, Amol M. Kanampalliwar, Shifu Aggarwal, Rupesh Dash, Rajeeb Swain, Punit Prasad, INSACOG Consortium, Ajay Parida
EPI_ISL_1419164, EPI_ISL_1419166, EPI_ISL_1419411, EPI_ISL_1419413, EPI_ISL_1419414, EPI_ISL_1419415, EPI_ISL_1419416, EPI_ISL_1419427, EPI_ISL_1419598, EPI_ISL_1419629, EPI_ISL_1419630, EPI_ISL_1419635, EPI_ISL_1419638, EPI_ISL_1419639, EPI_ISL_1419640, EPI_ISL_1419641, EPI_ISL_1419642, EPI_ISL_1419643, EPI_ISL_1419644, EPI_ISL_1419645, EPI_ISL_1419647, EPI_ISL_1419648, EPI_ISL_1419690, EPI_ISL_1419780, EPI_ISL_1419781, EPI_ISL_1419797, EPI_ISL_1419804, EPI_ISL_1419805, EPI_ISL_1419806, EPI_ISL_1419921			
see above	INSACOG-WB	National Institute of Biomedical Genomics - INSACOG	Arindam Maitra, Bhaswati Bandyopadhyay, Nidhan Kumar Biswas, Tamal Ghosh, Sreedhar Chinnaswamy, Ajay Chakraborti, Saumitra Das
EPI_ISL_1540456	National Centre For Cell Science	National Centre For Cell Science - INSACOG	Dhiraj Paul, Mohak P Gujare, Shivang P. Bhanushali, Mitali Inamdar, Sonal Manik Chavan, Manoj Kumar Bhat, Ajay Pillai, INSACOG Consortium team, Yogesh Shouche
EPI_ISL_1544058, EPI_ISL_1544060, EPI_ISL_1544061, EPI_ISL_1544062, EPI_ISL_1544063, EPI_ISL_1544070, EPI_ISL_1544071	National Centre For Cell Science	National Centre For Cell Science - INSACOG	Dhiraj Paul, Mohak P Gujare, Shivang P. Bhanushali, Mitali Inamdar, Sonal Manik Chavan, Manoj Kumar Bhat, Ajay Pillai, INSACOG Consortium team, Yogesh Shouche
EPI_ISL_1544133	GMERS, Medical College, Gandhinagar	Gujarat Biotechnology Research Centre	Gaurishankar Shrimali,Sonal Sharma ,Zuber Saiyed,Ramesh Pandit,Janvi Raval,Zarna Patel,Nitin Savaliya,Dinesh Kumar,Twinkle Soni,Umang Mishra,Nitesh Shah,Chaitanya Joshi,Madhvi Joshi
EPI_ISL_1544134	GMERS, Medical College, Gandhinagar	Gujarat Biotechnology Research Centre	Zuber Saiyed,Ramesh Pandit,Janvi Raval,Zarna Patel,Nitin Savaliya,Dinesh Kumar,Twinkle Soni,Sonal Sharma ,Gaurishankar Shrimali,Umang Mishra,Nitesh Shah,Chaitanya Joshi,Madhvi Joshi
EPI_ISL_1547802, EPI_ISL_1547803, EPI_ISL_1547804, EPI_ISL_1547805	CSIR-National Environmental Engineering Research Institute	CSIR-Centre for Cellular and Molecular Biology - INSACOG	Onkar Kulkarni, Lamuk Zaveri, Ara Sreenivas, Sofia Banu, Shreekant Verma, Amareshwar Vodapalli , Viswagithe S L, B Himasri, Sharath Chandra Thota, Karthik Bharadwaj Tallapaka, Krishna Khairnar, Rakesh K Mishra,Divya Tej Sowpati
EPI_ISL_1589745, EPI_ISL_1589747, EPI_ISL_1589748, EPI_ISL_1589750, EPI_ISL_1589751, EPI_ISL_1589753, EPI_ISL_1589754, EPI_ISL_1589755, EPI_ISL_1589756, EPI_ISL_1589758, EPI_ISL_1589759, EPI_ISL_1589760, EPI_ISL_1589761, EPI_ISL_1589762, EPI_ISL_1589763, EPI_ISL_1589764, EPI_ISL_1589765, EPI_ISL_1589766, EPI_ISL_1589767, EPI_ISL_1589768, EPI_ISL_1589770, EPI_ISL_1589771, EPI_ISL_1589772, EPI_ISL_1589773, EPI_ISL_1589774, EPI_ISL_1589775, EPI_ISL_1589776, EPI_ISL_1589777, EPI_ISL_1589779, EPI_ISL_1589780, EPI_ISL_1589781, EPI_ISL_1589782, EPI_ISL_1589783, EPI_ISL_1589784, EPI_ISL_1589785, EPI_ISL_1589787, EPI_ISL_1589788, EPI_ISL_1589789, EPI_ISL_1589790, EPI_ISL_1589795, EPI_ISL_1589797, EPI_ISL_1589800, EPI_ISL_1589803, EPI_ISL_1589804, EPI_ISL_1589805, EPI_ISL_1589806, EPI_ISL_1589807, EPI_ISL_1589808, EPI_ISL_1589809, EPI_ISL_1589810, EPI_ISL_1589811, EPI_ISL_1589812, EPI_ISL_1589813, EPI_ISL_1589814, EPI_ISL_1589815, EPI_ISL_1589817, EPI_ISL_1589818, EPI_ISL_1589819, EPI_ISL_1589820, EPI_ISL_1589853, EPI_ISL_1589854, EPI_ISL_1589857, EPI_ISL_1589859, EPI_ISL_1589860, EPI_ISL_1589861, EPI_ISL_1589862, EPI_ISL_1589863, EPI_ISL_1589864, EPI_ISL_1589866, EPI_ISL_1589867, EPI_ISL_1589868, EPI_ISL_1589869, EPI_ISL_1589870, EPI_ISL_1589871, EPI_ISL_1589872, EPI_ISL_1589873, EPI_ISL_1589874, EPI_ISL_1589875, EPI_ISL_1589876, EPI_ISL_1589877, EPI_ISL_1589878, EPI_ISL_1589879, EPI_ISL_1589880, EPI_ISL_1589881, EPI_ISL_1589882, EPI_ISL_1589883, EPI_ISL_1589884, EPI_ISL_1589885, EPI_ISL_1589886, EPI_ISL_1589887, EPI_ISL_1589888, EPI_ISL_1589889, EPI_ISL_1589890, EPI_ISL_1589891, EPI_ISL_1589892, EPI_ISL_1589893, EPI_ISL_1589894, EPI_ISL_1589895, EPI_ISL_1589896, EPI_ISL_1589897, EPI_ISL_1589898, EPI_ISL_1589899, EPI_ISL_1589900, EPI_ISL_1589901, EPI_ISL_1589902, EPI_ISL_1589903, EPI_ISL_1589905, EPI_ISL_1589906, EPI_ISL_1589907, EPI_ISL_1589908, EPI_ISL_1589909, EPI_ISL_1589910, EPI_ISL_1589911, EPI_ISL_1589912, EPI_ISL_1589913, EPI_ISL_1589914, EPI_ISL_1589915, EPI_ISL_1589916, EPI_ISL_1589917, EPI_ISL_1589918, EPI_ISL_1589919, EPI_ISL_1589920, EPI_ISL_1589921, EPI_ISL_1589922, EPI_ISL_1589923, EPI_ISL_1589924, EPI_ISL_1589925, EPI_ISL_1589926, EPI_ISL_1589927, EPI_ISL_1589928, EPI_ISL_1589929, EPI_ISL_1589930, EPI_ISL_1589931, EPI_ISL_1589932, EPI_ISL_1589933, EPI_ISL_1589934, EPI_ISL_1589935, EPI_ISL_1589936, EPI_ISL_1589937, EPI_ISL_1589938, EPI_ISL_1589939, EPI_ISL_1589940, EPI_ISL_1589941, EPI_ISL_1589942, EPI_ISL_1589944, EPI_ISL_1589945, EPI_ISL_1589946, EPI_ISL_1589947, EPI_ISL_1589948, EPI_ISL_1589949, EPI_ISL_1589950, EPI_ISL_1589951, EPI_ISL_1589956, EPI_ISL_1589957, EPI_ISL_1589958, EPI_ISL_1589959, EPI_ISL_1589960, EPI_ISL_1589961, EPI_ISL_1589962, EPI_ISL_1589963, EPI_ISL_1589964, EPI_ISL_1589965, EPI_ISL_1589966, EPI_ISL_1589969, EPI_ISL_1589970, EPI_ISL_1589971, EPI_ISL_1589972			
see above	INSACOG-WB	National Institute of Biomedical Genomics - INSACOG	Arindam Maitra, Bhaswati Bandyopadhyay, Nidhan Kumar Biswas, Tamal Ghosh, Sreedhar Chinnaswamy, Ajay Chakraborti, Saumitra Das
EPI_ISL_1595862	BBMP Urban PHC	INSACOG-KA, NIMHANS	Chitra Pattabiraman, Pramada Prasad, Anson Kunjumon George, Ananthapadmanabha Kotambail, Darshan Sreenivas, Chetan G K, Gautham Arunachal Udupi, Anita S Desai, V Ravi
EPI_ISL_1595866	BANGALORE MEDICAL COLLEGE AND RESEARCH INSTITUTE	INSACOG-KA, NIMHANS	Chitra Pattabiraman, Pramada Prasad, Anson Kunjumon George, Ananthapadmanabha Kotambail, Darshan Sreenivas, Chetan G K, Gautham Arunachal Udupi, Anita S Desai, V Ravi
EPI_ISL_1595882	WENLOCK HOSPITAL, MANGALORE	INSACOG-KA, NIMHANS	Chitra Pattabiraman, Pramada Prasad, Anson Kunjumon George, Ananthapadmanabha Kotambail, Darshan Sreenivas, Chetan G K, Gautham Arunachal Udupi, Anita S Desai, V Ravi
EPI_ISL_1595896, EPI_ISL_1595897, EPI_ISL_1595899	KEMPEGOWDA INTERNATIONAL AIRPORT	INSACOG-KA, NIMHANS	Chitra Pattabiraman, Pramada Prasad, Anson Kunjumon George, Ananthapadmanabha Kotambail, Darshan Sreenivas, Chetan G K, Gautham Arunachal Udupi, Anita S Desai, V Ravi
EPI_ISL_1595907, EPI_ISL_1595909, EPI_ISL_1595910, EPI_ISL_1595916	BBMP Urban PHC	INSACOG-KA, NIMHANS	Chitra Pattabiraman, Pramada Prasad, Anson Kunjumon George, Ananthapadmanabha Kotambail, Darshan Sreenivas, Chetan G K, Gautham Arunachal Udupi, Anita S Desai, V Ravi
EPI_ISL_1662277, EPI_ISL_1662278, EPI_ISL_1662279, EPI_ISL_1662280, EPI_ISL_1662281	Nalanda Medical College & Hospital, Patna	Institute of Life Sciences - INSACOG	Sunil K. Raghav, Safal Walia, Arup Ghosh, Atimukta Jha, Amol M. Kanampalliwar, Shifu Aggarwal, Rupesh Dash, Rajeeb Swain, Punit Prasad, INSACOG Consortium, Ajay Parida
EPI_ISL_1662283, EPI_ISL_1662284, EPI_ISL_1662285, EPI_ISL_1662286	Veer Surendra Sai Institute of Medical Sciences and Research, Burla, Sambalpur	Institute of Life Sciences - INSACOG	Sunil K. Raghav, Safal Walia, Arup Ghosh, Atimukta Jha, Amol M. Kanampalliwar, Shifu Aggarwal, Rupesh Dash, Rajeeb Swain, Punit Prasad, INSACOG Consortium, Ajay Parida
EPI_ISL_1662290, EPI_ISL_1662291	Dept. Of Microbiology, Lt.. Baliram Kashyap Memorial Govt. Medical college, Dimrapal, Jagdalpur	Institute of Life Sciences - INSACOG	Sunil K. Raghav, Safal Walia, Arup Ghosh, Atimukta Jha, Amol M. Kanampalliwar, Shifu Aggarwal, Rupesh Dash, Rajeeb Swain, Punit Prasad, INSACOG Consortium, Ajay Parida
EPI_ISL_1662387, EPI_ISL_1662401	BRLSABVM Government Medical College, Rajnandgaon, Chhattisgarh	Institute of Life Sciences - INSACOG	Sunil K. Raghav, Safal Walia, Arup Ghosh, Atimukta Jha, Amol M. Kanampalliwar, Shifu Aggarwal, Rupesh Dash, Rajeeb Swain, Punit Prasad, INSACOG Consortium, Ajay Parida
EPI_ISL_1662422, EPI_ISL_1662423	S.C.B. Medical College and Hospital, Cuttack	Institute of Life Sciences - INSACOG	Sunil K. Raghav, Safal Walia, Arup Ghosh, Atimukta Jha, Amol M. Kanampalliwar, Shifu Aggarwal, Rupesh Dash, Rajeeb Swain, Punit Prasad, INSACOG Consortium, Ajay Parida
EPI_ISL_1662424, EPI_ISL_1662425, EPI_ISL_1662426, EPI_ISL_1662427, EPI_ISL_1662428, EPI_ISL_1662430, EPI_ISL_1662431, EPI_ISL_1662432, EPI_ISL_1662434, EPI_ISL_1662435, EPI_ISL_1662436, EPI_ISL_1662437, EPI_ISL_1662439, EPI_ISL_1662440, EPI_ISL_1662441, EPI_ISL_1662442, EPI_ISL_1662443,			

We gratefully acknowledge the following Authors from the Originating laboratories responsible for obtaining the specimens, as well as the Submitting laboratories where the genome data were generated and shared via GISAID, on which this research is based.

All Submitters of data may be contacted directly via www.gisaid.org

Authors are sorted alphabetically.

Accession ID	Originating Laboratory	Submitting Laboratory	Authors
EPI_ISL_2924258, EPI_ISL_2924259, EPI_ISL_2924260, EPI_ISL_2924261, EPI_ISL_2924262, EPI_ISL_2924263	Osmania Medical College	CDFD	Murali Bashyam, Asmita Gupta, Reelina Basu, Vinay Donipadi, Nagamani Kammili, Divya Vashisht, Ashwin Dalal
EPI_ISL_2924264, EPI_ISL_2924265, EPI_ISL_2924267	Gandhi Hospital	CDFD	Murali Bashyam, Asmita Gupta, Reelina Basu, Vinay Donipadi, Nagamani Kammili, Divya Vashisht, Ashwin Dalal

South Africa

We gratefully acknowledge the following Authors from the Originating laboratories responsible for obtaining the specimens, as well as the Submitting laboratories where the genome data were generated and shared via GISAID, on which this research is based.

All Submitters of data may be contacted directly via www.gisaid.org

Authors are sorted alphabetically.

Accession ID	Originating Laboratory	Submitting Laboratory	Authors
EPI_ISL_1040648, EPI_ISL_1040650, EPI_ISL_1040651, EPI_ISL_1040652, EPI_ISL_1040653, EPI_ISL_1040654	Groote Schuur Hospital wc GSH	NHLS/UCT	Arash Iranzadeh, Deelan Doolabh, Lynn Tyers, Bruna Galvao, Innocent Mudau, Marvin Hsiao, Kruger Marais, Diana Hardie, Stephen Korsman, Carolyn Williamson
EPI_ISL_1040655	Pacaltsdorp Clinic wc PAC	NHLS/UCT	Arash Iranzadeh, Deelan Doolabh, Lynn Tyers, Bruna Galvao, Innocent Mudau, Marvin Hsiao, Kruger Marais, Diana Hardie, Stephen Korsman, Carolyn Williamson
EPI_ISL_1040656, EPI_ISL_1040657, EPI_ISL_1040658, EPI_ISL_1040659, EPI_ISL_1040661	Groote Schuur Hospital wc GSH	NHLS/UCT	Arash Iranzadeh, Deelan Doolabh, Lynn Tyers, Bruna Galvao, Innocent Mudau, Marvin Hsiao, Kruger Marais, Diana Hardie, Stephen Korsman, Carolyn Williamson
EPI_ISL_1040662	Heideveld Emergency Centre	NHLS/UCT	Arash Iranzadeh, Deelan Doolabh, Lynn Tyers, Bruna Galvao, Innocent Mudau, Marvin Hsiao, Kruger Marais, Diana Hardie, Stephen Korsman, Carolyn Williamson
EPI_ISL_1040663	Groote Schuur Hospital wc GSH	NHLS/UCT	Arash Iranzadeh, Deelan Doolabh, Lynn Tyers, Bruna Galvao, Innocent Mudau, Marvin Hsiao, Kruger Marais, Diana Hardie, Stephen Korsman, Carolyn Williamson
EPI_ISL_1040682	False Bay Hospital wc FBH	NHLS/UCT	Arash Iranzadeh, Deelan Doolabh, Lynn Tyers, Bruna Galvao, Innocent Mudau, Marvin Hsiao, Kruger Marais, Diana Hardie, Stephen Korsman, Carolyn Williamson
EPI_ISL_1040683, EPI_ISL_1040685, EPI_ISL_1040686, EPI_ISL_1040687	Groote Schuur Hospital wc GSH	NHLS/UCT	Arash Iranzadeh, Deelan Doolabh, Lynn Tyers, Bruna Galvao, Innocent Mudau, Marvin Hsiao, Kruger Marais, Diana Hardie, Stephen Korsman, Carolyn Williamson
EPI_ISL_1040688	Heideveld Emergency Centre	NHLS/UCT	Arash Iranzadeh, Deelan Doolabh, Lynn Tyers, Bruna Galvao, Innocent Mudau, Marvin Hsiao, Kruger Marais, Diana Hardie, Stephen Korsman, Carolyn Williamson
EPI_ISL_1040689, EPI_ISL_1040690, EPI_ISL_1040691, EPI_ISL_1040692, EPI_ISL_1040693	Groote Schuur Hospital wc GSH	NHLS/UCT	Arash Iranzadeh, Deelan Doolabh, Lynn Tyers, Bruna Galvao, Innocent Mudau, Marvin Hsiao, Kruger Marais, Diana Hardie, Stephen Korsman, Carolyn Williamson
EPI_ISL_1040694, EPI_ISL_1040695	Hanover Park CHC wc HPH	NHLS/UCT	Arash Iranzadeh, Deelan Doolabh, Lynn Tyers, Bruna Galvao, Innocent Mudau, Marvin Hsiao, Kruger Marais, Diana Hardie, Stephen Korsman, Carolyn Williamson
EPI_ISL_1040696, EPI_ISL_1040697, EPI_ISL_1040698, EPI_ISL_1040699, EPI_ISL_1040700	Groote Schuur Hospital wc GSH	NHLS/UCT	Arash Iranzadeh, Deelan Doolabh, Lynn Tyers, Bruna Galvao, Innocent Mudau, Marvin Hsiao, Kruger Marais, Diana Hardie, Stephen Korsman, Carolyn Williamson
EPI_ISL_1040701	Mowbray Maternity Hospital wc MMH	NHLS/UCT	Arash Iranzadeh, Deelan Doolabh, Lynn Tyers, Bruna Galvao, Innocent Mudau, Marvin Hsiao, Kruger Marais, Diana Hardie, Stephen Korsman, Carolyn Williamson
EPI_ISL_1040702	Lentegeur Hospital wc LGH	NHLS/UCT	Arash Iranzadeh, Deelan Doolabh, Lynn Tyers, Bruna Galvao, Innocent Mudau, Marvin Hsiao, Kruger Marais, Diana Hardie, Stephen Korsman, Carolyn Williamson
EPI_ISL_1040703	Nieuveldpark Clinic wc NVP	NHLS/UCT	Arash Iranzadeh, Deelan Doolabh, Lynn Tyers, Bruna Galvao, Innocent Mudau, Marvin Hsiao, Kruger Marais, Diana Hardie, Stephen Korsman, Carolyn Williamson
EPI_ISL_1040704, EPI_ISL_1040705, EPI_ISL_1040706, EPI_ISL_1040707, EPI_ISL_1040708	Groote Schuur Hospital wc GSH	NHLS/UCT	Arash Iranzadeh, Deelan Doolabh, Lynn Tyers, Bruna Galvao, Innocent Mudau, Marvin Hsiao, Kruger Marais, Diana Hardie, Stephen Korsman, Carolyn Williamson
EPI_ISL_1040709	Lentegeur Hospital wc LGH	NHLS/UCT	Arash Iranzadeh, Deelan Doolabh, Lynn Tyers, Bruna Galvao, Innocent Mudau, Marvin Hsiao, Kruger Marais, Diana Hardie, Stephen Korsman, Carolyn Williamson
EPI_ISL_1040710	Guguletu CHC wc GDH	NHLS/UCT	Arash Iranzadeh, Deelan Doolabh, Lynn Tyers, Bruna Galvao, Innocent Mudau, Marvin Hsiao, Kruger Marais, Diana Hardie, Stephen Korsman, Carolyn Williamson
EPI_ISL_1040711	Groote Schuur Hospital wc GSH	NHLS/UCT	Arash Iranzadeh, Deelan Doolabh, Lynn Tyers, Bruna Galvao, Innocent Mudau, Marvin Hsiao, Kruger Marais, Diana Hardie, Stephen Korsman, Carolyn Williamson
EPI_ISL_1040712	Mowbray Maternity Hospital wc MMH	NHLS/UCT	Arash Iranzadeh, Deelan Doolabh, Lynn Tyers, Bruna Galvao, Innocent Mudau, Marvin Hsiao, Kruger Marais, Diana Hardie, Stephen Korsman, Carolyn Williamson
EPI_ISL_1040713	Guguletu CHC wc GDH	NHLS/UCT	Arash Iranzadeh, Deelan Doolabh, Lynn Tyers, Bruna Galvao, Innocent Mudau, Marvin Hsiao, Kruger Marais, Diana Hardie, Stephen Korsman, Carolyn Williamson
EPI_ISL_1040714	Groote Schuur Hospital wc GSH	NHLS/UCT	Arash Iranzadeh, Deelan Doolabh, Lynn Tyers, Bruna Galvao, Innocent Mudau, Marvin Hsiao, Kruger Marais, Diana Hardie, Stephen Korsman, Carolyn Williamson
EPI_ISL_1040715	Guguletu CHC wc GDH	NHLS/UCT	Arash Iranzadeh, Deelan Doolabh, Lynn Tyers, Bruna Galvao, Innocent Mudau, Marvin Hsiao, Kruger Marais, Diana Hardie, Stephen Korsman, Carolyn Williamson
EPI_ISL_1040716	Groote Schuur Hospital wc GSH	NHLS/UCT	Arash Iranzadeh, Deelan Doolabh, Lynn Tyers, Bruna Galvao, Innocent Mudau, Marvin Hsiao, Kruger Marais, Diana Hardie, Stephen Korsman, Carolyn Williamson
EPI_ISL_1040717	Hanover Park CHC wc HPH	NHLS/UCT	Arash Iranzadeh, Deelan Doolabh, Lynn Tyers, Bruna Galvao, Innocent Mudau, Marvin Hsiao, Kruger Marais, Diana Hardie, Stephen Korsman, Carolyn Williamson
EPI_ISL_1040718	Guguletu CHC wc GDH	NHLS/UCT	Arash Iranzadeh, Deelan Doolabh, Lynn Tyers, Bruna Galvao, Innocent Mudau, Marvin Hsiao, Kruger Marais, Diana Hardie, Stephen Korsman, Carolyn Williamson
EPI_ISL_1040719	Heideveld CDC wc HVP	NHLS/UCT	Arash Iranzadeh, Deelan Doolabh, Lynn Tyers, Bruna Galvao, Innocent Mudau, Marvin Hsiao, Kruger Marais, Diana Hardie, Stephen Korsman, Carolyn Williamson
EPI_ISL_1040720	Groote Schuur Hospital wc GSH	NHLS/UCT	Arash Iranzadeh, Deelan Doolabh, Lynn Tyers, Bruna Galvao, Innocent Mudau, Marvin Hsiao, Kruger Marais, Diana Hardie, Stephen Korsman, Carolyn Williamson
EPI_ISL_1040721	Guguletu CHC wc GDH	NHLS/UCT	Arash Iranzadeh, Deelan Doolabh, Lynn Tyers, Bruna Galvao, Innocent Mudau, Marvin Hsiao, Kruger Marais, Diana Hardie, Stephen Korsman, Carolyn Williamson

Bibliography

[1] Motoo Kimura. "Estimation of evolutionary distances between homologous nucleotide sequences". In: *Proceedings of the National Academy of Sciences* 78.1 (1981), pp. 454–458.
